# Multi-scale electronics transport properties in non-ideal CVD graphene sheet

**DOI:** 10.1038/s41598-022-15098-6

**Published:** 2022-07-02

**Authors:** Bhupesh Bishnoi, Marius Buerkle, Hisao Nakamura

**Affiliations:** National Institute of Advanced Industrial Science and Technology (AIST), Research Center for Computational Design of Advanced Functional Materials (CD-FMat), Central 2, Umezono 1-1-1, Tsukuba, Ibaraki 305-8568 Japan

**Keywords:** Electronic properties and devices, Two-dimensional materials

## Abstract

In this work, we benchmark non-idealities and variations in the two-dimensional graphene sheet. We have simulated more than two hundred graphene-based devices structure. We have simulated distorted graphene sheets and have included random, inhomogeneous, asymmetric out-of-plane surface corrugation and in-plane deformation corrugation in the sheet through autocorrelation function in the non-equilibrium Green’s function (NEGF) framework to introduce random distortion in flat graphene. These corrugation effects inevitably appear in the graphene sheet due to background substrate roughness or the passivation encapsulation material morphology in the transfer step. We have examined the variation in density of state, propagating density of transmission modes, electronic band structure, electronic density, and hole density in those device structures. We have observed that the surface corrugation increases the electronic and hole density distribution variation across the device and creates electron-hole charge puddles in the sheet. This redistribution of microscopic charge in the sheet is due to the lattice fields’ quantum fluctuation and symmetry breaking. Furthermore, to understand the impact of scattered charge distribution on the sheet, we simulated various impurity effects within the NEGF framework. The study’s objective is to numerically simulate and benchmark numerous device design morphology with different background materials compositions to elucidate the electrical property of the sheet device.

## Introduction

Since the isolation of single-layer graphene in 2004, graphene has shown promising research results in laboratories across the globe^1^, Furthermore, tremendous progress was made in fabricating and characterization techniques to fabricate large-area graphene sheet devices^2,3^. However, large-scale commercial production and market adoption are still very far from the horizon. The possible utilization of graphene sheets is in various applications from the transparent electrode, electronics, telecommunication equipment, and internet of thing (IoT) sensors. To realize this commercial adoption in the electronics and optoelectronics industry, the main challenge for graphene is in the controllability, homogeneity, and uniformity in the electronic property in the mass roll-to-roll production process. The growth and transfer process, such as temperature variation and gas flow fluctuations in the chemical vapor deposition reactor, give rise to long-range spatial variation in the electronics performance parameter of the large-area graphene sheet. It prevents the seamless stitching of graphene single grain, which gives rise to imperfect lattices and grain boundary interfaces, causing a decrease in conductance^4–8^. Moreover, the geometrical circumstance of the graphene sheet transfer scheme gives rise to spatial variation in the doping density. Therefore, for homogeneous and uniform graphene sheets, defect density is an important measure. However, we must consider the graphene sheet’s average defect density and spatial defect distribution profile to quantify the effect^9^. Moreover, all those issues, as mentioned above, are a challenge to scale up the production. There are various methods proposed to fabricate the graphene sheet on an industrial scale, such as exfoliation, the epitaxial method^10^, chemical vapor deposition (CVD)^11^, Roll-to-Roll plasma-enhanced chemical vapor deposition (PECVD) method^12–15^. However, the synthesis of graphene on metal catalysts by CVD has been the most scalable process until now. Besides, all these process steps have their underline chemistry and influence on the quality of the graphene sheet^16^. Therefore, the electrical quality of CVD/PECVD grown graphene sheets varied broadly from process to process and batch to batch. Besides, the transfer and isolation process are bottlenecks in the production scale and incorporate defects in the sheet and degrade the sheet’s electrical quality^17–20^. These nanoscale atomistic effects during the growth and transfer steps influence the vital electrical properties of the sheet through variation in background carrier density, carrier mobility, and sheet resistivity. Electrical property mapping is critical for the process optimization and quality control of large-area graphene sheets in Roll-to-Roll production. In terms of throughput and quality, all the electrical characterization method available is far behind the Roll-to-Roll production process. In recent times to numerically probe the above-mentioned microscopic variations and non-idealities in the macroscopic device, some notable theoretical and numerical efforts have been made in the community, e.g., Stegmann et al. investigate the current flow paths in the deformed graphene by using modified hopping parameters in a tight-binding approach and connecting it with the current classical trajectories in curved space^21^. Also, the influence of a Gaussian strain on sublattice selectivity of impurities in graphene is investigated by modified tight-binding parameters and investigated the local density of states variation^22–25^. However, unlike our multi-scale study, they investigate a single strain defect of Gaussian curvature in the flat graphene sheet or nanoribbon. In a realistic device scenario, however, many fluctuations exist and are random in nature. Also, local sublattice symmetry breaking and electron-hole puddles formation in graphene is theoretically discussed in some detail^26^, and our numerical results match their prediction. Other than the above work, multi-scale quantum ballistic transport calculations have been performed in the recent past on flat graphene^27,28^. Furthermore, our flat graphene sample calculation matches with them. To the best of the author’s knowledge, very few multi-scale incoherent and phase-coherent quantum transport calculations are performed in the graphene and two-dimensional system. All the scattering mechanisms are left as the residual calculation for future work. In this study, first, we will discuss and summarize the various numerical simulation results for the graphene device’s various aspects, variabilities, and non-idealities in device simulation, to elucidate the nature of variability in the measurand electrical properties for benchmarking purposes. In the subsequent article under preparation, we will also report the effect of each scattering mechanism in multi-scale simulation, their combined effect as self-energy incorporating explicitly in the NEGF loop as self-consistent Dyson equation in graphene sheet devices. Furthermore, The multi-scale non-equilibrium Green’s function formalism framework detail, its incoherent extension, implementation, important assumption, approximation, bottom-up atomistic tight-binding models, and numerical truncation to treat these non-ideal graphene devices are discussed in the method section and great detail at supplementary information section. Next, in the following result and discussion section, we will discuss the multi-scale non-equilibrium Green’s function formalism numerical results and their physical interpretations. Finally, in the last section, we conclude the article by summarizing the results, observations, and future work in the two-dimensional material field.

Next, in the following methods section, we will discuss the multi-scale non-equilibrium Green’s function formalism used to get these results in the aforementioned section.

## Method

At the nano-scale atomic composition, crystal symmetry and the spatial disorder affect the material’s bulk properties as quantum mechanics effects come into the picture and modify the electronic-phononic structure. Therefore, atomistic simulations are more appropriate to model the quantum device’s electronic properties in the entire Brillouin zone^29^. The electronic band structure derives from the tight-binding method. The method is similar to the linear combination of atomic orbitals (LCAO) used to construct molecular orbitals^30,31^. However, in the tight-binding approximation, electron-electron interactions of the orbital are neglected, but it gives an excellent approximation to electronic band structure. Furthermore, for more rigorous treatment, the Hubbard model was employed. The graphene is a two-dimensional sheet of carbon atoms arranged in a hexagonal lattice. $2\mathrm {S}, 2\mathrm {P_{x}}$, and $2\mathrm {P_{y}}$ orbitals of carbon atom in graphene are $\mathrm {S}\mathrm {P}^{2}$ hybridize, resulting in strong $\sigma$ bonds where each carbon atom is bonded to all of its three neighbors carbon atoms. The $\mathrm {P_{z}}$ or $\pi$ orbital of the carbon atoms defines the low-energy electronic bandstructure in graphene.

The primitive lattice vectors in graphene are,

1 $$\begin{aligned} \begin{aligned} \vec {a}_1 = \frac{\sqrt{3}a}{2} \hat{x}+ \frac{a}{2}\hat{y}; \\ \vec {a}_2 = \frac{\sqrt{3}a}{2} \hat{x}- \frac{a}{2}\hat{y} . \end{aligned} \end{aligned}$$

The graphene primitive unit cell comprises two carbon atoms, and each atom has one valence $2\mathrm {P_{z}}$ orbital. At the C1 atoms site valence $\phi _{\text {2p}_{z1}}$ orbital is centered and at the C2 atoms site valence $\phi _{\text {2p}_{z2}}$ orbital is centered, The tight binding wavefunction for graphene is,

2 $$\begin{aligned} \psi _{\vec {k}}\left( \vec {r}\right) =\frac{1}{\sqrt{N}}\sum \limits _{h,j}e^{i\left( h\vec {k}\cdot \vec {a}_1 + j\vec {k}\cdot \vec {a}_2\right) } \left( c_1\phi _{\text {2p}_{z1}}\left( \vec {r}-h\vec {a}_1-j\vec {a}_2\right) + c_2 \phi _{\text {2p}_{z2}}\left( \vec {r}-h\vec {a}_1-j\vec {a}_2\right) \right) . \end{aligned}$$

After multiplying from the left by both of $\mathrm {P_{z}}$ orbitals to time-independent Schrödinger equation and integrating overall space, the dispersion relation is,

3 $$\begin{aligned} \begin{aligned} \langle \phi _{\text {2p}_{z1}}|\hat{H}|\psi _{k}\rangle = E\langle \phi _{\text {2p}_{z1}}|\psi _{k}\rangle ; \\ \langle \phi _{\text {2p}_{z2}}|\hat{H}|\psi _{k}\rangle = E\langle \phi _{\text {2p}_{z2}}|\psi _{k}\rangle . \end{aligned} \end{aligned}$$

In tight-binding approximation, on-site and nearest-neighbor matrix elements are retained, and all the other terms are assumed too small enough to ignore the equation,

4 $$\begin{aligned} \begin{aligned} \varepsilon c_1 -tc_2\left( 1+e^{-i\vec {k}\cdot \vec {a_1}} + e^{-i\vec {k}\cdot \vec {a_2}}\right) = Ec_1; \\ \varepsilon c_2 -tc_1\left( 1+e^{i\vec {k}\cdot \vec {a_1}} + e^{i\vec {k}\cdot \vec {a_2}}\right) = Ec_2 . \end{aligned} \end{aligned}$$

where

$$\begin{aligned} \begin{aligned} \varepsilon&= \big \langle \phi _{\text {2p}_{z1}}\left( \vec {r}\right) |\hat{H}|\phi _{\text {2p}_{z1}}\left( \vec {r}\right) \big \rangle ; \\ \mathrm {and} \;\; t&= - \big \langle \phi _{\text {2p}_{z1}}\left( \vec {r}\right) |\hat{H}|\phi _{\text {2p}_{z2}}\left( \vec {r}\right) \big \rangle = - \big \langle \phi _{\text {2p}_{z1}}\left( \vec {r}\right) |\hat{H}|\phi _{\text {2p}_{z1}}\left( \vec {r}-\frac{a}{\sqrt{3}}\hat{x}\right) \big \rangle . \end{aligned} \end{aligned}$$

Graphene electronic band structure is defined in the tight-binding approximation while considering interactions up to third-nearest neighbors as^32,33^,

5 $$\begin{aligned} \hat{H}=\hat{H}_{1}+\hat{H}_{3}. \end{aligned}$$

where $\hat{H}_{1}$ is the first nearest neighbor Hamiltonian and $\hat{H}_{3}$ third nearest neighbor Hamiltonian. In the second quantization language, Hamiltonians are in real space representation is expressed as creation and annihilation operator acting on the $\pi$ state on each carbon atom site as follow,

6 $$\begin{aligned} \begin{aligned} \hat{H}_{1}&=\sum _{\langle n,l\rangle }t_{n,l}\hat{c}_{n}^{\dagger }\hat{c}_{l};\\ \hat{H}_{3}&=\sum _{\langle n,m\rangle }t_{n,m}\hat{c}_{n}^{\dagger }\hat{c}_{m}. \end{aligned} \end{aligned}$$

where $c^{\dagger }$ creation and *c* annihilation operators, and summation runs over entire *n* lattice point, and *l* is first and *m* is third nearest neighbor site of *n* lattice point. First nearest-neighbor hopping parameter $t_{n,l} = -2.74 \; \mathrm {eV}$ and third nearest neighbor $t_{n,m} =-0.3\; \mathrm {eV}$. We have also passivated the dangling bond at the device edge by hydrogen passivation treatment in the device simulation^34^. In the tight-binding framework dangling bonds at the surface or edge is passivated by primarily two numerical methods. In the first strategy, passivation atoms are implicitly incorporated without distinguishing passivation atom types and add a passivation potential to the dangling bonds’ orbital energies. This method works well with a relatively large system, arbitrary crystal structures, and hybridization symmetries. Furthermore, with appropriate parameters, it is applied to any passivation scenario^35^. The second approach is explicit, including the passivation atoms and their coupling to the surface or edge atoms Hamiltonian matrix and limited to small molecules and systems^36^. In this explicit treatment, ab-initio results for different passivation atoms used to fitting targets. The unsaturated dangling bonds at the edge or surface will result in edge or surface states at the electronic band structure. These unwanted states iron out by coupling the hydrogen passivation atoms to the surface’s or edge’s unsaturated dangling bonds in the device. We have used the $\mathrm {P-D}$ orbital tight-binding model, which represents the edge effects by explicitly including the passivated hydrogen in the Hamiltonian matrix. The carbon atom is represented by three $\mathrm {P_{z}}$, $\mathrm {D_{yz}}$, and $\mathrm {D_{zx}}$ orbitals. The simple single orbital $\mathrm {P_{z}}$ tight-binding model works well for the two-dimensional graphene sheet. More further details about the tight-binding framework address in the article’s supplementary information. In 1960 Keldysh, Kadanoff, and Baym first developed the non-equilibrium Green’s function formalism (NEGF)^37,38^. The adaption of NEGF formalism to semiconductor devices first demonstrated by Lake and Datta^39–42^, in 1992 and later in 2002 by Wacker^43^. Through NEGF formalism, the time evolution of many-body quantum fields in thermodynamic equilibrium and non-equilibrium state can be investigated^44–49^. These quantum fields constitute carriers such as electrons, phonons, spin, and electric-field in semiconductor devices^50–60^.

The theoretical framework of Non-equilibrium Green’s function formalism with detailed reference is given in the supplementary information. Here we briefly summarize the NEGF formalism on a discrete device grid as,

7 $$\begin{aligned} \begin{aligned} EG_{nm}^{<}(\varvec{k}_{\mathrm {t}};E)-\sum _{l}h_{nl}G_{lm}^{<}(\varvec{k}_{\mathrm {t}};E)&= \sum _{l}\Sigma _{nl}^{R}(\varvec{k}_{\mathrm {t}};E)G_{lm}^{<}(\varvec{k}_{\mathrm {t}};E)+\sum _{l}\Sigma _{nl}^{<}(\varvec{k}_{\mathrm {t}};E)G_{lm}^{A}(\varvec{k}_{\mathrm {t}};E); \\ EG_{nm}^{<}(\varvec{k}_{\mathrm {t}};E)-\sum _{l}G_{nl}^{<}(\varvec{k}_{\mathrm {t}};E)h_{lm}&= \sum _{l}G_{nl}^{R}(\varvec{k}_{\mathrm {t}};E)\Sigma _{lm}^{<}(\varvec{k}_{\mathrm {t}};E)+\sum _{l}G_{nl}^{<}(\varvec{k}_{\mathrm {t}};E)\Sigma _{{lm}}^{A}(\varvec{k}_{\mathrm {t}};E); \\ G_{nm}^{<}(\varvec{k}_{\mathrm {t}};E)\,&=\ \sum _{l,v}G_{nl}^{R}(\varvec{k}_{\mathrm {t}};E)\Sigma _{lv}^{<}(\varvec{k}_{\mathrm {t}};E)G_{vm}^{A}(\varvec{k}_{\mathrm {t}};E); \\ G_{nm}^{<}(\varvec{k}_{\mathrm {t}};E)\,&=\ -[G_{mn}^{<}(\varvec{k}_{\mathrm {t}};E)]^{\dagger }; \\ EG_{nm}^{R}(\varvec{k}_{\mathrm {t}};E)-\sum _{l}h_{nl}G_{lm}^{R}(\varvec{k}_{\mathrm {t}};E)&= \delta _{nm}+\sum _{l}\Sigma _{nl}^{R}(\varvec{k}_{\mathrm {t}};E)G_{lm}^{R}(\varvec{k}_{\mathrm {t}};E); \\ G_{nm}^{A}(\varvec{k}_{\mathrm {t}};E)\,&=\ [G_{mn}^{R}(\varvec{k}_{\mathrm {t}};E)]^{\dagger }; \\ G_{mn}^{R}(\varvec{k}_{\mathrm {t}};E)-G_{mn}^{A}(\varvec{k}_{\mathrm {t}};E)\,&=\ G_{mn}^{>}(\varvec{k}_{\mathrm {t}};E)-G_{mn}^{<}(\varvec{k}_{\mathrm {t}};E); \\ A(\varvec{k}_{\mathrm {t}};E)&= i\Big [G_{mn}^{R}(\varvec{k}_{\mathrm {t}};E)-G_{mn}^{A}(\varvec{k}_{\mathrm {t}};E)\Big ]. \end{aligned} \end{aligned}$$

where the bold symbol represent the vector space quantities, $G^{R}$ retarded, $G^{A}$ advanced, $G^{<}$ lesser, $G^{>}$ greater Green’s functions, $\Sigma ^{R}$ retarded, $\Sigma ^{A}$ advanced, $\Sigma ^{<}$ lesser, $\Sigma ^{>}$ greater self-energies of interaction between various quantum field, *A* is spectral function related to the spectral peak of Green’s functions in energy spectrum, $\varvec{k}_{\mathrm {t}}=(k_{x}, k_{y})$ is parallel momentum in vector space, *E* is energy space, $\delta$ is Kronecker delta in discrete energy-momentum space where Green’s functions realized it’s value, system Hamiltonian $h_{nm}=\int \mathrm {d}\varvec{r}\phi _{n}^{*}(\varvec{r})H_{0}(\varvec{r})\phi _{m}(\varvec{r})$ with hermitian $h_{ml}^{*}=h_{lm}$ property, $\phi$ is tight-binding orbital describing the system from Eq. (3), $\varvec{r}$ is position space, and *n*, *m*, *l*, *v* are discrete lattice point of device where Green’s propagator travel in coupled system of Eq. (7). From Eq. (7) the stationary state solution of non-equilibrium Green’s functions, the Density of state $\mathcal {D}(E)$ is defined by taking the trace of lesser $G^{<}$ Green’s functions as,

8 $$\begin{aligned} \mathcal {D}(E) = -\frac{1}{\pi }\mathrm {Tr} \Bigg [\sum _{\varvec{k}_{\mathrm {t}}}\sum _{nm}G_{nm}^{<}(\varvec{k}_{\mathrm {t}};E)\Bigg ]. \end{aligned}$$

The carrier density $\mathfrak {N} (\varvec{r}, t)$ in the non-equilibrium Green’s functions formalism is defined as,

9 $$\begin{aligned} \mathfrak {N} (\varvec{r}, t) = -i\hbar G^{<}(\varvec{r}, t;\varvec{r}, t). \end{aligned}$$

In the stationary solution regime from Eq. (7) of NEGF in the $(\mathrm {z})$ directional transport, eigenfunction expansion as orthonormal eigenstate $\phi _{\varvec{k}_{\mathrm {t}}n}(z)$, $\phi _{\varvec{k}_{\mathrm {t}}m}^{*}(z)$, the carrier density as,

10 $$\begin{aligned} \mathfrak {N}(z) = -\frac{i}{A}\sum _{\varvec{k}_{\mathrm {t}}}\sum _{nm}\int \frac{\mathrm {d}E}{2\pi }G_{nm}^{<} (\varvec{k}_{\mathrm {t}};E)\phi _{\varvec{k}_{\mathrm {t}}n}(z)\phi _{\varvec{k}_{\mathrm {t}}m}^{*}(z). \end{aligned}$$

For the contacted device with an external source and continuous supply of electrons in the non-equilibrium configuration $G_{nm}^{<}(\varvec{k}_{\mathrm {t}}; E)$ is defined at different points in the device. Where *l* belongs to the active part of the device, *m* belongs to any point in the contacts with defined carriers Fermi distribution, and *n* belongs to the interface between both regions. Using the corresponding boundary conditions for $h_{ml, lm}$ and with equilibrium contacts assumption as in bulk contacts, the Fermi distribution is in equilibrium. By using the fluctuation-dissipation theorem, the charge exchange rate $\Gamma _{ll_{1}}^{\mathrm {Contact}}(\varvec{k}_{\mathrm {t}};E)$ with the contact is,

11 $$\begin{aligned} \Gamma _{ll_{1}}^{\mathrm {Contact}}(\varvec{k}_{\mathrm {t}};E) = \sum _{m\le n}\sum _{m_{1}\le n}h_{lm}{A_{mm_{1}}}(\varvec{k}_{\mathrm {t}};E)h_{m_{1}l_{1}} \end{aligned}$$

where $A_{mm_{1}}$ is spectral function correspond to lattice indices point at $mm_{1}$ and $h_{lm}$, $h_{m_{1}l_{1}}$ is corresponding points at surface Hamiltonian in the boundary condition. From Eq. (10), and using the argument of Eq. (11), The Transmission probability $\mathcal {T}$ at $(\varvec{k}_{\mathrm {t}};E)$ is defined as,

12 $$\begin{aligned} \mathcal {T}(\varvec{k}_{\mathrm {t}};E) = \bigg \{\Gamma _{ll_{1}}^{L}G_{l_{1}l_{2}}^{R}\Gamma _{l_{2}l_{3}}^{R}G_{l_{3}l}^{A}\bigg \}(\varvec{k}_{\mathrm {t}};E) \end{aligned}$$

where in the non-interacting active part of the device indices $l, l_{1}, l_{2}, l_{3}$ run covering all the points. And, $\Gamma _{ll_{1}}^{L}$ is connected towards the left contact and $\Gamma _{l_{2}l_{3}}^{R}$ connected through the right contact. From the argument of Eqs. (11) and (12), the total number of the transmissible propagating modes of the wave-function in the device is defined as Density of Modes or Modes Density $\mathcal {M}$ at energy (*E*) is,

13 $$\begin{aligned} \mathcal {M}(E) = \ \frac{1}{ A}\sum _{ll_{1}}\sum _{l_{2}l_{3}}\sum _{\varvec{k}_{\mathrm {t}}}\int \bigg \{\Gamma _{ll_{1}}^{L}G_{l_{1}l_{2}}^{R} \Gamma _{l_{2}l_{3}}^{R}G_{l_{3}l}^{A}\bigg \}(\varvec{k}_{\mathrm {t}};E){\mathrm {d}E}. \end{aligned}$$

Though NEGF formalism is computationally intensive to solve compared to classical drift-diffusion and semi-classical Boltzmann transport approach and has simulation overhead, it is a complete quantum treatment. Therefore, it gives excellent detail of insight into device operation in spatial resolution. We have employed Nano-Electronics Modeling Tools $(\mathrm {NEMO5})$, a multi-scale quantum transport kernel for nano-electronic device modeling^61,62^. Its modular architecture parallelizes in a five-level message passing interface (MPI) in the position, momentum, energy, bias, and random seeding space. Due to the modular architecture of the transport kernel, various physical models add up and extend into it, the detailed discussion provided in the article’s supplementary information.

## Result and discussion

We have employed multi-scale, multi-physics-based bottom-up, non-equilibrium Green’s function mechanism-based quantum transport simulation techniques to investigate various device aspects and deduce the observable and measurable at the final stage in the computationally simulated devices. Moreover, most reported publications in the graphene and two-dimensional material field calculate electrical parameters of coherent transport limit and leave scattering interaction behind as a residual. The scattering nature and origin depend on graphene’s different fabrication process steps, the device orientation and substrate, and surface encapsulation in the device preparation step. The complete stepwise description of the theoretical framework provides in the article’s method section and supplementary information. This industrial research work was envisioned to show the opportunity of improving the electrical characterization of the roll-to-roll CVD graphene production process. Therefore, our results in this work are directed to corrugations and impurities effect in the reasonably clean state-of-art industrial production and transfer process. Here we will discuss the main results from numerical calculation.

### Surface corrugation effect

**Figure 1:**
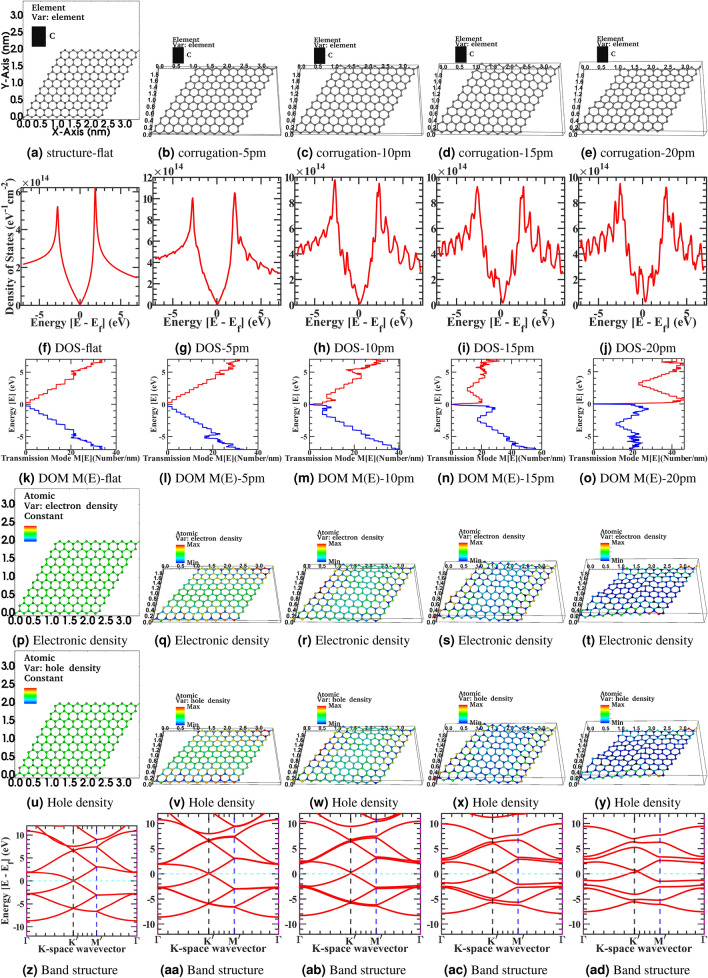
Device structure 10x10 graphene supercell (**a**–**e**) corrugate-5pm to −20pm, Density of state (**f**) flat to (**j**) corrugate-5pm to −20pm, Electronic density of modes M(E) in x-y Direction (**k**) flat to (**o**) corrugate-5pm to -20pm, Electronic density (**p**) flat to (**t**) corrugate-5pm to −20pm, Hole density (**u**) flat to (**y**) corrugate-5pm to −20pm, and Electronic band structure (**z**) flat to (**ad**) corrugate-5pm to −20pm.

**Figure 2:**
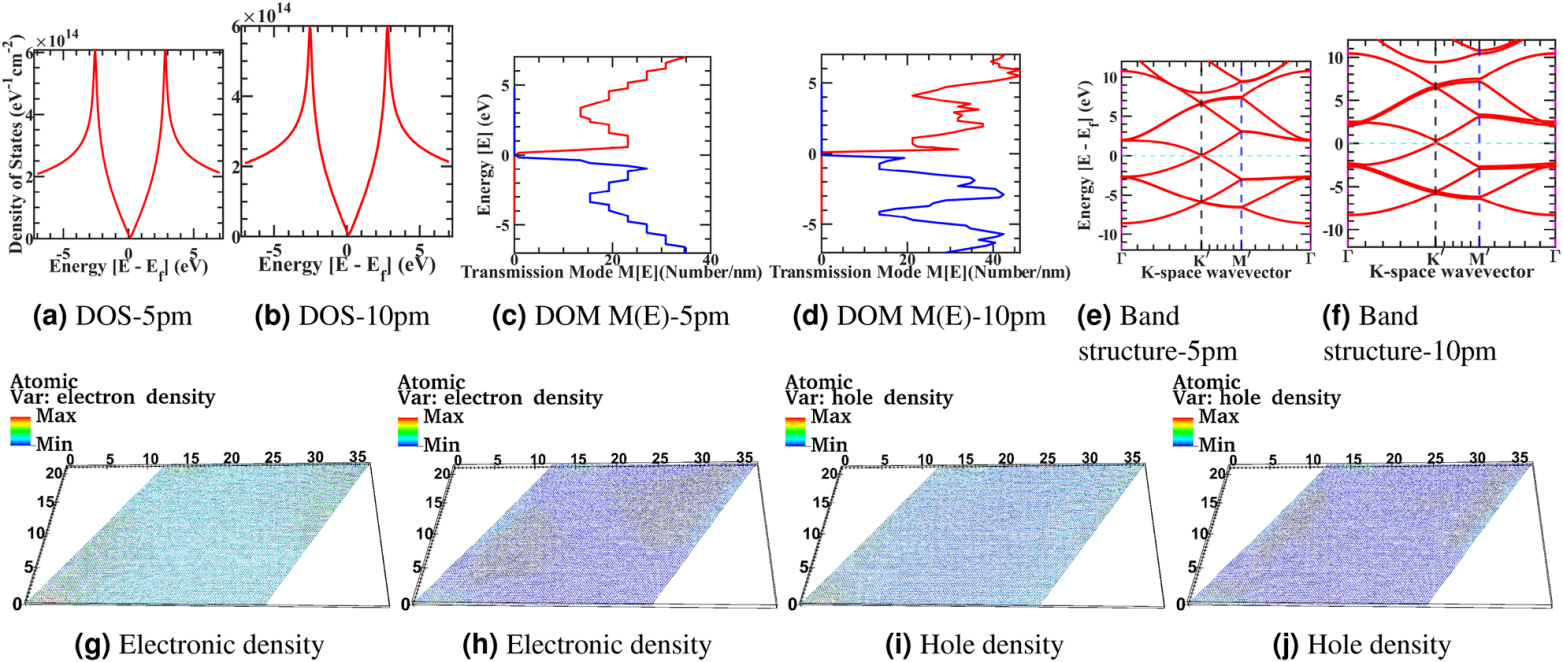
Device structure 100x100 graphene supercell with surface corrugation 5pm and 10pm, and respectively Density of state (**a**, **b**), Electronic density of modes M(E) in x-y Direction (**c**, **d**), Electronic band structure (**e**, **f**), Electronic density (**g**, **h**), and Hole density (**i**, **j**).

**Figure 3:**
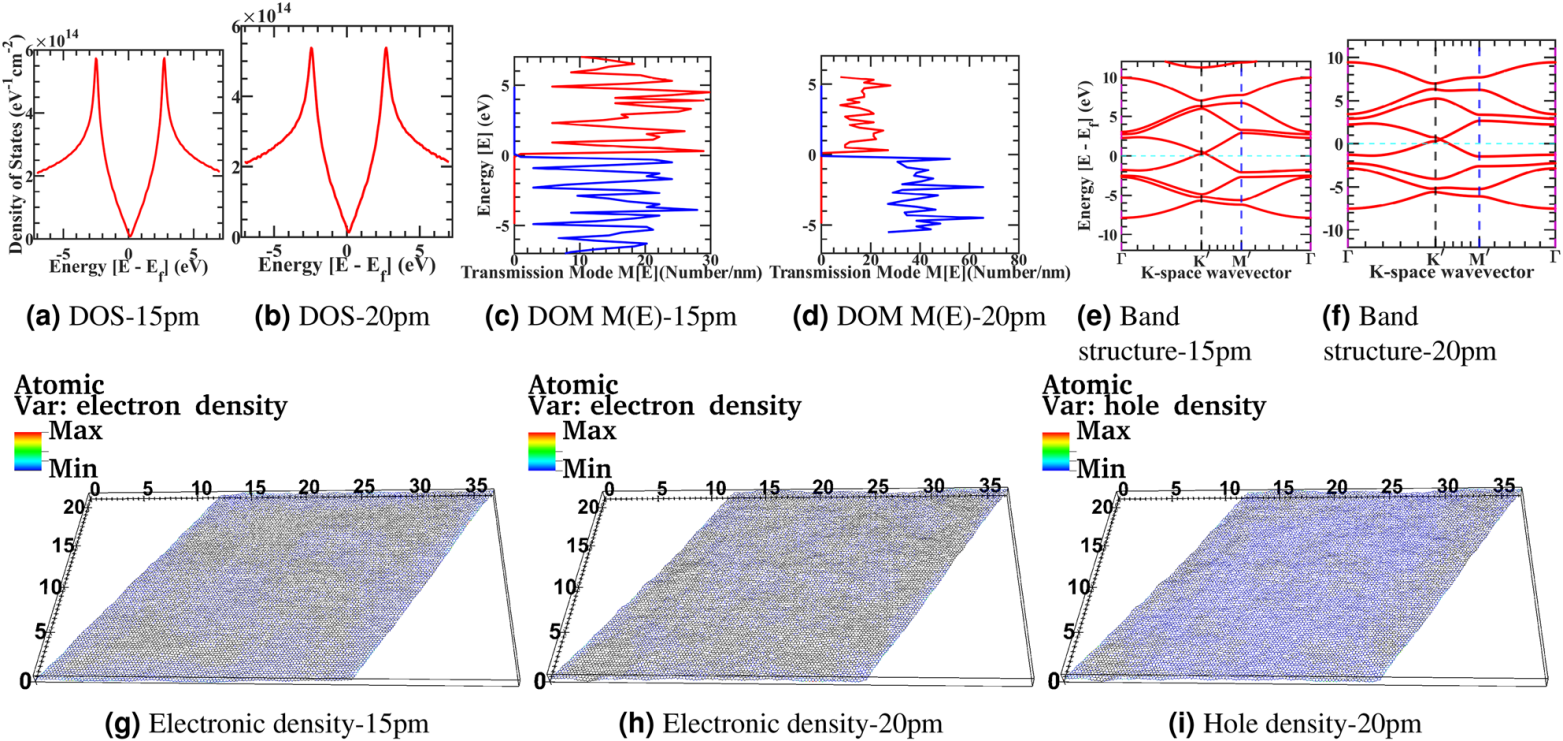
Device structure 100x100 graphene supercell with surface corrugation 15pm and 20pm, and respectively Density of state (**a**, **b**), Electronic density of modes M(E) in x-y Direction (**c**, **d**), Electronic band structure (**e**, **f**), Electronic density (**g**, **h**), and Hole density (**i**) at 20pm.

**Figure 4:**
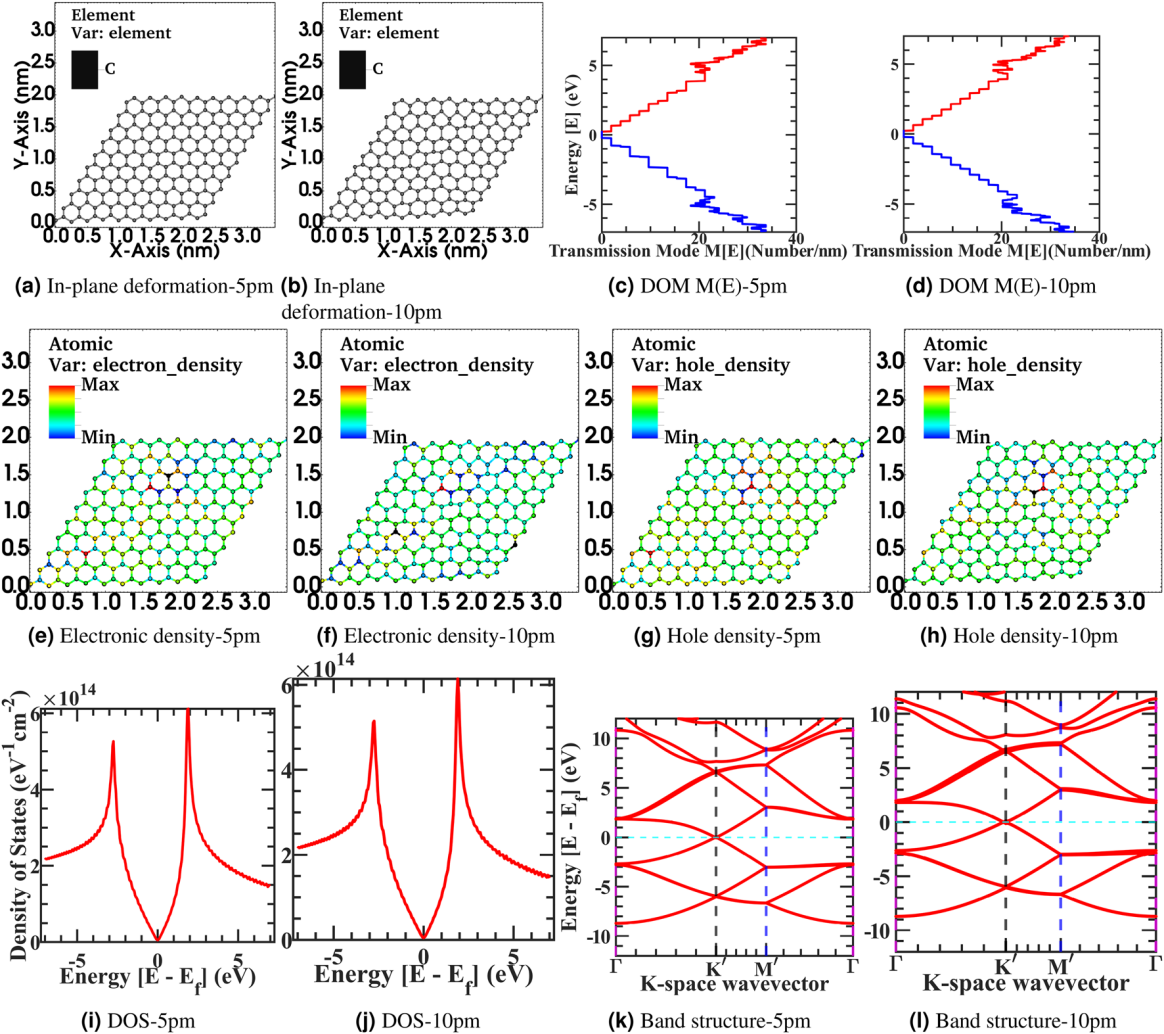
Device structure 10x10 graphene supercell (**a**, **b**) in-plane deformation roughness-5pm to −10pm, Electronic density of modes M(E) in x-y Direction (**c**, **d**) in-plane deformation-5pm to −10pm, Electronic density (**e**, **f**) in-plane deformation-5pm to -10pm, Hole density (**g**, **h**) in-plane deformation-5pm to −10pm, Density of state (**i**, **j**) in-plane deformation-5pm to −10pm, and Electronic band structure (**k**, **l**) in-plane deformation-5pm to −10pm.

**Figure 5:**
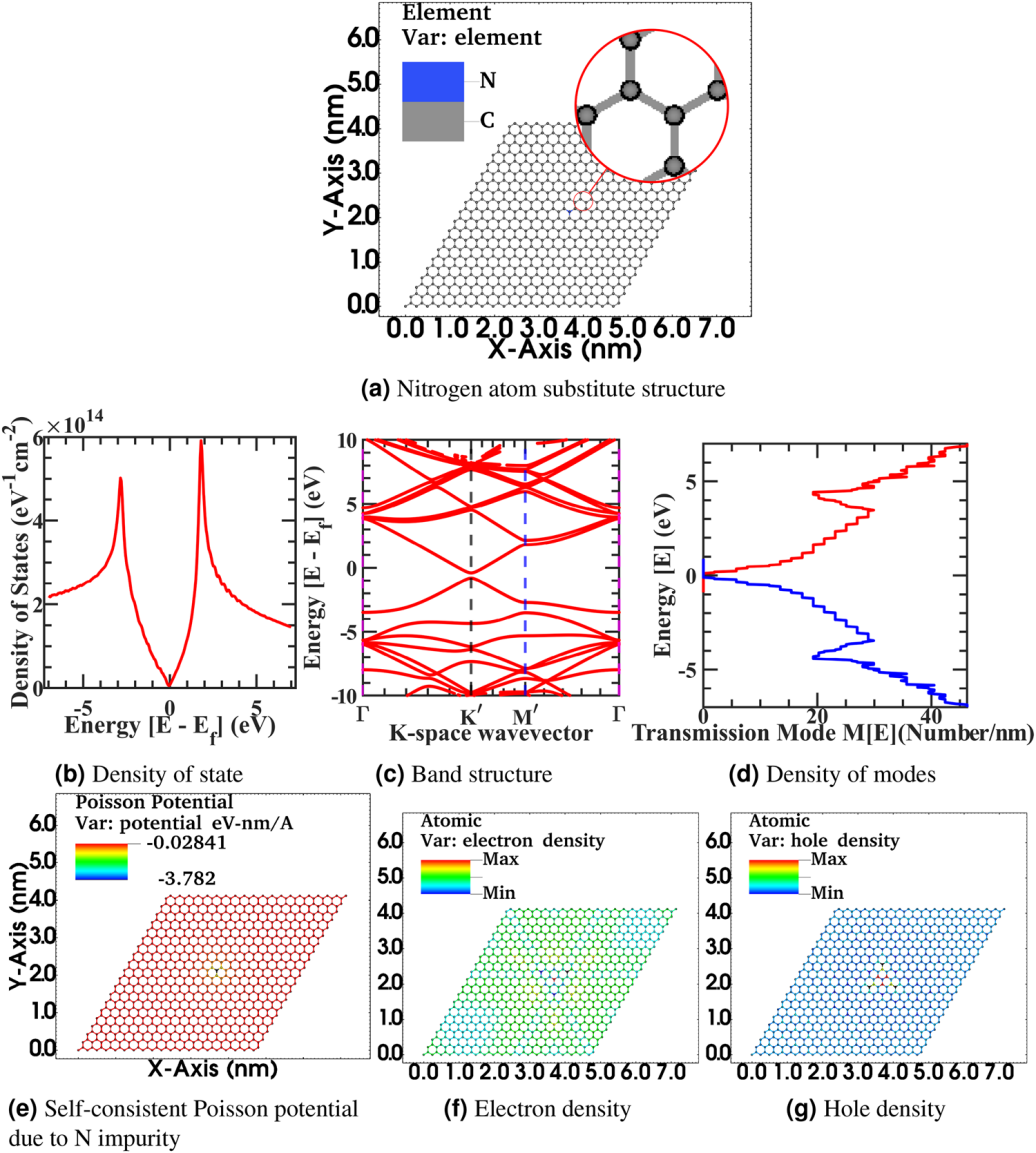
Primitive device structure 20 x 20 graphene supercell with substitutional Nitrogen impurity atom structure (**a**), Density of state (**b**), Electronic band structure (**c**), Density of modes (**d**), Self-consistent Poisson potential due to Nitrogen impurity (**e**), Electronic density (**f**), Hole density (**g**).

**Figure 6:**
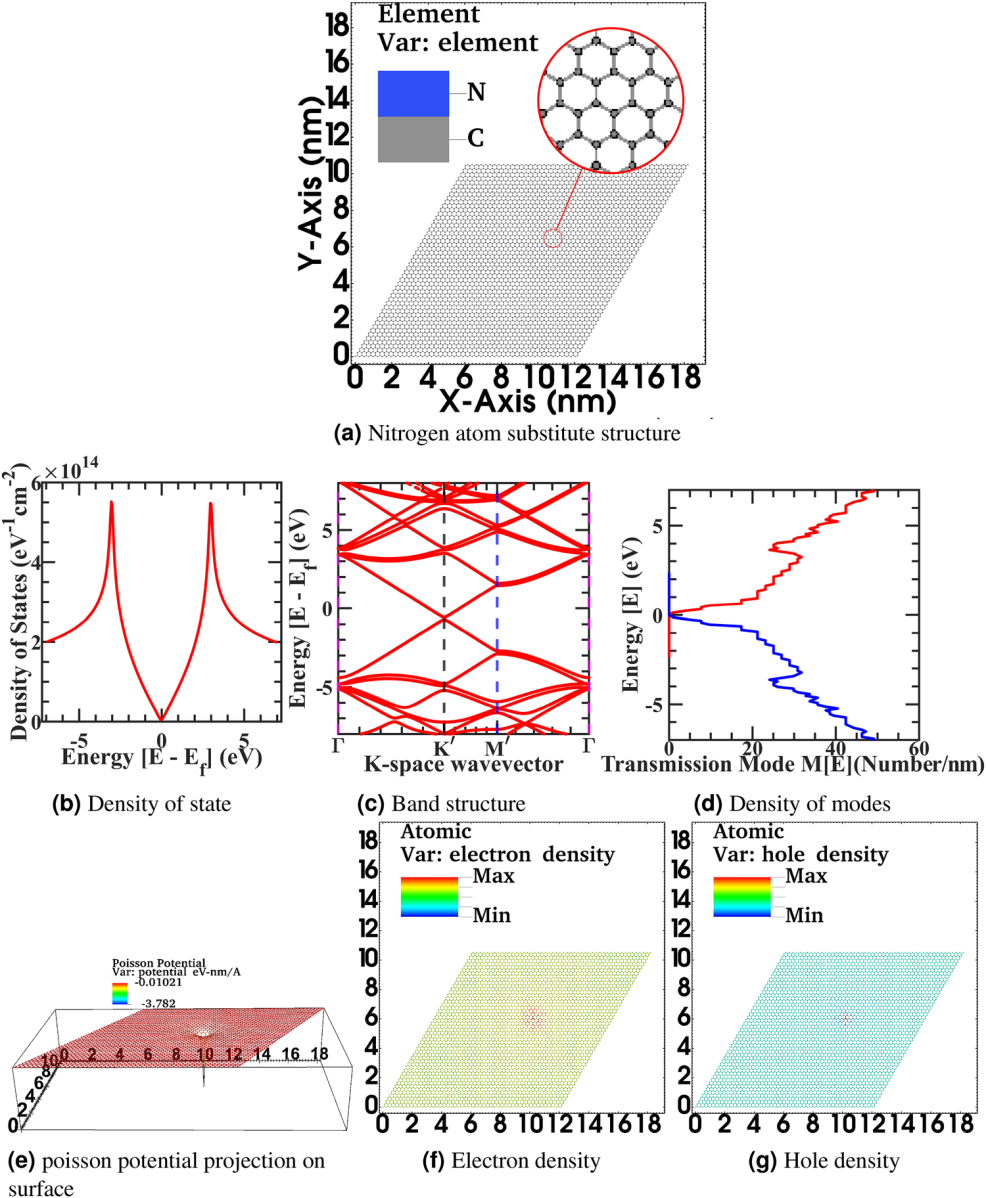
Primitive device structure 50 x 50 graphene supercell with substitutional Nitrogen impurity (**a**), Density of state (**b**), Electronic band structure (**c**), Density of modes (**d**), Self-consistent Poisson potential due to Nitrogen impurity projection on surface (**e**) Electronic density (**f**), Hole density (**g**).

**Figure 7:**
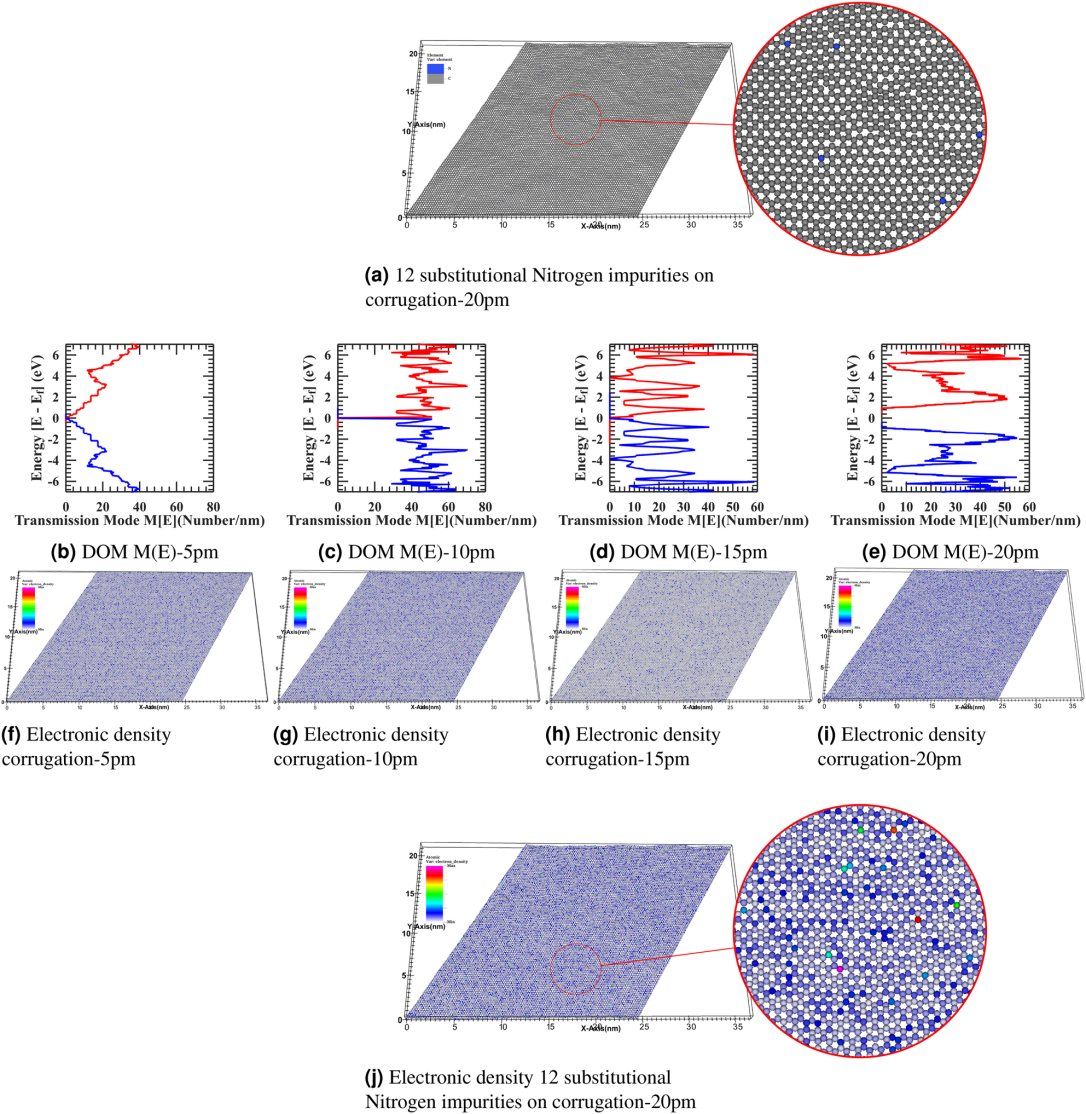
(**a**) Device structure of corrugated 100 x 100 graphene supercell with 12 substitutional Nitrogen impurities for corrugate-5pm to 20pm, Electronic density of modes M(E) in x-y Direction (**b**–**e**) for corrugate-5pm to 20pm, Electronic density (**f**–**i**) for corrugate-5pm to 20pm, and (**j**) close up version of corrugate 20pm.

**Figure 8:**
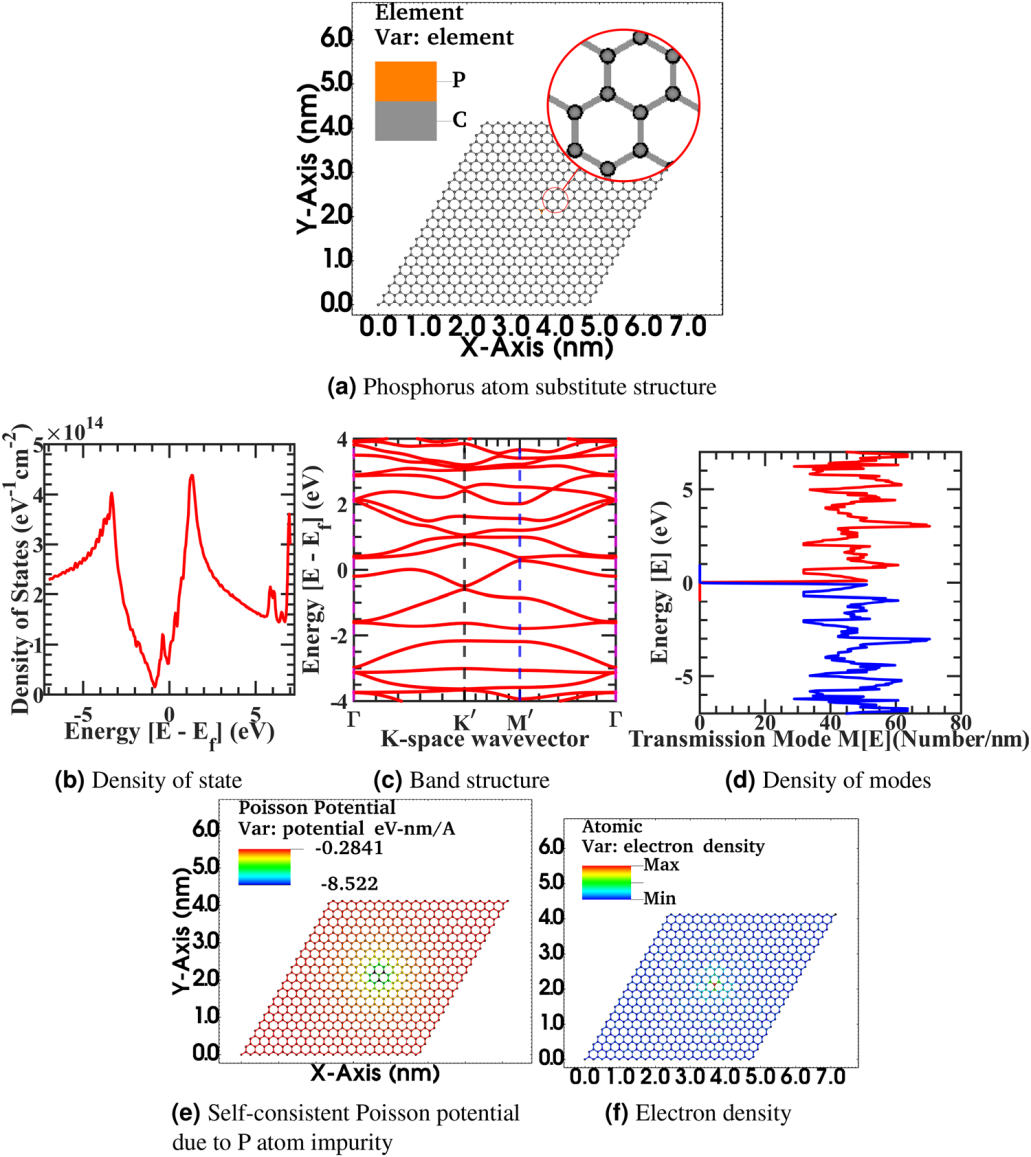
Primitive device structure 20 x 20 graphene supercell with substitutional phosphorus impurity with ten electronic charge (**a**), Density of state (**b**), Electronic band structure (**c**), Density of modes (**d**), Self-consistent Poisson potential due to Phosphorus impurity (**e**), Electronic density (**f**).

**Figure 9:**
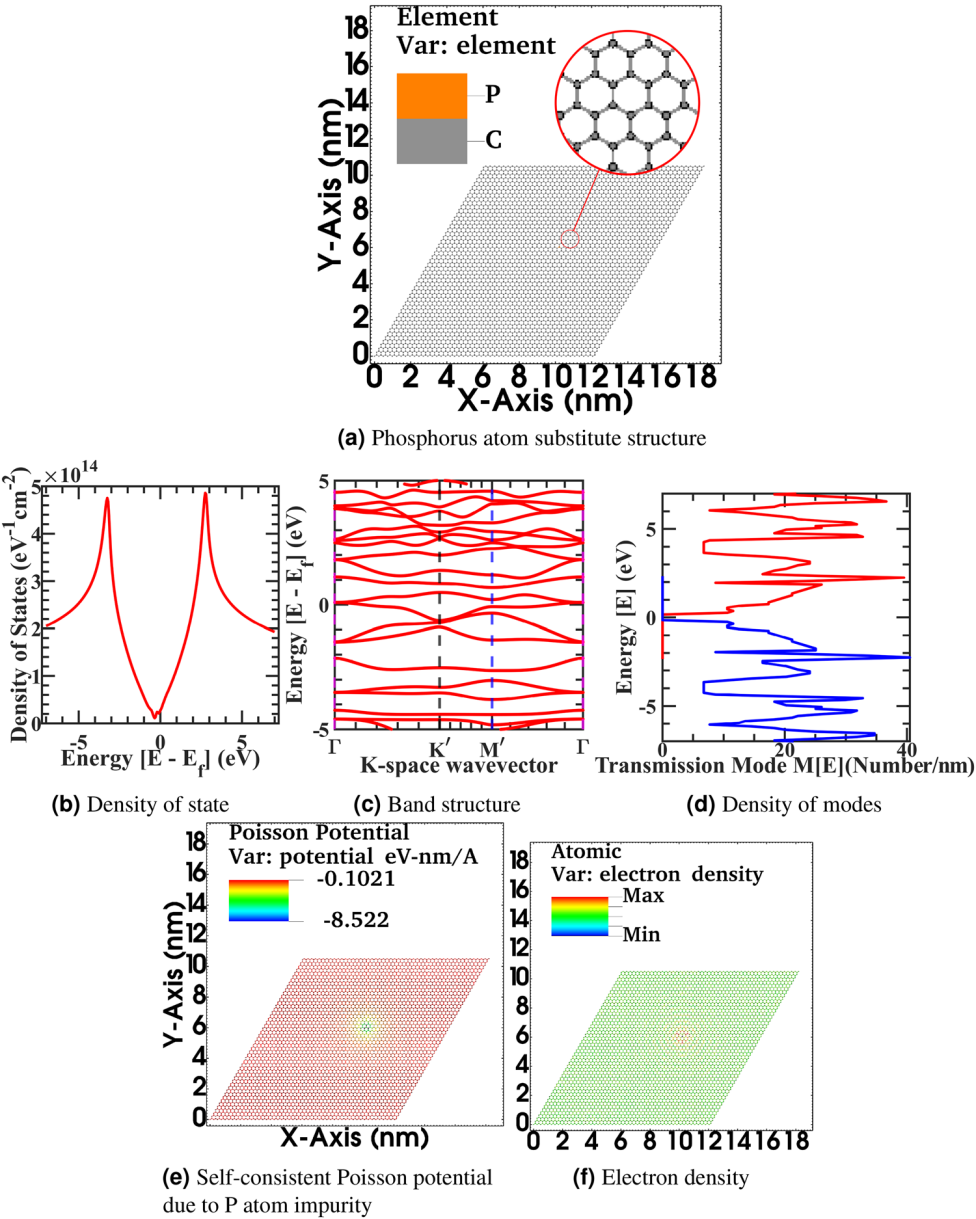
Primitive device structure 50 x 50 graphene supercell with substitutional phosphorus impurity with ten electronic charge (**a**), Density of state (**b**), Electronic band structure (**c**), Density of modes (**d**), Self-consistent Poisson potential due to Phosphorus impurity (**e**), Electronic density (**f**).

We have investigated the out-of-plane random, inhomogeneous, asymmetric structural corrugation effect in the electrically open infinite reservoirs contacted geometry of graphene sheet to examine the large variability across the electric properties of CVD graphene sheet. Graphene is a single atom thin sheet of the carbon atom and needs a substrate to work as a device. The substrate’s effect is manifold, such as corrugation in the graphene sheet by the substrate surface profile and scattering through the substrate’s impurities^63,64^. Due to thermodynamic instability, the corrugation and ripple have been observed in the free-standing and substrate-born graphene sheet devices. There is modest work reported on deformed graphene. Most of the previous work concentrated on a uniform, uniaxial or biaxial strain and centrosymmetric deformations^65–69^. Katsnelson et al. derived the mathematical formulation for microscopic defects and generic random potential by ripples for the Dirac fermions from the semi-classical Boltzmann transport view^70^. The fluctuating interatomic distances and angles between carbon bonds are described by in-plane and out-of-plane atomic displacements deformation tensor in the corrugated graphene. This work tried to elongate their original idea in the non-equilibrium Green’s functions formalism (NEGF) based quantum transport domain. Based on the substrate material type and fabrication process, the corrugation profile has considerable variation from 0.30 $\mathrm {nm}$ to 0.005 $\mathrm {nm}$ when placed on the substrate, graphene follows the underlying substrate’s surface morphology, and these ripples induce stress in the sheet. Therefore, it will influence the electronic property of the graphene sheet. Variation in bonding lengths and angle between the carbon atoms in the corrugated graphene sheet depends on substrate morphology. In tight biding calculation, it modulates the hopping parameters in the hamiltonian. From Harrison’s model, the hopping parameter is inversely proportional to the square of the bond length^71^. Due to corrugation, $p_{z}$-orbitals are also bent, but hopping parameter variation due to bond length variation is more prominent compared to orbital bending^72^. The surface corrugation effect in the graphene sheet modeled through the gaussian auto-correlation function in the transverse *x* and *y*-direction as,

14 $$\begin{aligned} R (x, y)=\delta h^{2}\exp \Bigg (-\frac{x^{2}}{l_{x}^{2}}-\frac{y^{2}}{l_{y}^{2}}\Bigg ). \end{aligned}$$

where $\delta h$ is the root mean square amplitude of fluctuations height. The correlation length $l_{x}$ and $l_{y}$ are in spatial domain morphology repetition length scale. The surface corrugation effect in the lattice is created in simulation by taking the Fourier transform of auto-correlation function from Eq. (14), applying a random phase, and taking an inverse Fourier transformation. In experiment, depending upon the fabrication and transfer process, and underline substrate morphology, the correlation length varies from 2 to 30 $\mathrm {nm}$ and corrugation amplitude 0.30 $\mathrm {nm}$ to 0.005 $\mathrm {nm}$ from the $SiO_2$ to h-BN substrate. To numerically simulate these substate effects and variation, we have calculated the density of state from Eq. (8), electronic density and hole density from Eq. (10), the density of propagating modes M(E) in x-, y-direction from Eq. (13), and electronic band structure from Eq. (7) for a flat and four different corrugation configuration. In corrugated graphene sheet the correlation length is kept at $10\mathrm {nm}$ and corrugation amplitude varying from $0.005$ to $0.020 \mathrm {nm}$ in the corresponding Figs. 1, 2, 3. The x, y, and z-dimension scales in all the figures in this article are in the nanometer scale resolution. Following the quantum chemistry field conventions, the device structures are mapped and indicated as the multiplication of unit cells dimension. Therefore, we omit to show x, y, and z-dimension nanometer scales explicitly to avoid excessive cluttering in the figures from Figs. 1, 2, 3, 4, 5, 6, 7, 8, 9 throughout the article. The Fig. 1a correspond to 10x10 graphene supercell flat structure, and Fig. 1b–e correspond to the corrugate device structure with roughness varying from 5 picometers (pm) to 20 picometers (pm), mimicking the roughness profile of corresponding h-BN and $\mathrm {SiO}_{2}$ substrate. Similarly, Fig. 1f–j represents the corresponding density of state, Fig. 1k–o represents the electronic density of modes M(E) in the x-y direction, Fig. 1p –t represents the electronic density, Fig. 1u–y represents the hole density and Fig. 1z–ad represents the electronic bandstructure of the corresponding device structure. In the simulated device, the primitive unit cell has two atoms per cell, and a finite element mesh simulates a total of 200 atoms with a domain size of 800 finite element mesh grid points. The P-D tight-binding model contains three orbitals, namely carbon $\mathrm {P_{z}}$, and carbon-hydrogen passivated $\mathrm {D_{yz}}$, $\mathrm {D_{xz}}$ orbitals. Therefore total degree of freedom in hamiltonian is 600 variable-sized orbitals. The $K^\prime$ and $M^\prime$ are high symmetric point corresponds to the folded reduce BZ-zone of graphene supercell. In the article’s supplementary information, from Fig. 1a–ad is also rendered in the full-text page resolution in the corresponding Supplementary Figs. 1–30 for the reader’s reference.

We have observed from Fig. 1f–j, the density of states fluctuates from the flat to deformed structure progressively gives rise to additional states to scattered and decreases conductance. Also, in the deformed sample, sharp peaks appear in the density of states at lower energies compared to undeformed flat samples. We have also observed the disorder-induced Dirac point in the density of state shifting from zero energy point towards the right due to a shift in Fermi-energy also visible in from band structure plots Fig. 1z–ad as beforehand announced and reported in Raman spectra of the experimental graphene sheet^73^. Moreover, the sharply peaked Van-Hove singularity point slowly shifts from low energy towards higher energy and slowly smoothing at these higher energies with the increased deformation in the sample. Even a tiny randomized inhomogeneous in-plane or out-of-plane deformation strain will break the local sublattice and inversion symmetry in the one atom thin graphene. It will give rise to an inhomogeneous pseudo magnetic field that produces the additional density of the state’s low-energy peaks and increases the scattering probability with a deteriorating conductance. Moreover, inhomogeneous pseudo field incipient a non-equivalent charge density localization and distribution across the asymmetric sublattices of deformed regions. Furthermore, creating the electron-hole puddles in the graphene sheet can be seen from the numerical calculation from the electronic density Fig. 1p–t and hole density Fig. 1u–y. This will be more visible next for a larger size 100x100 graphene supercell sample simulation for electronic density Figs. 2g–3h and hole density Figs. 2i–3i. In the deformed sheet where sublattice and inversion symmetry is locally broken, energy bandgap opening is not visible from Fig. 1z–ad. However, as discussed in other studies, a sizeable large gaussian deformation can introduce an energy bandgap in the corrugated sheet^74^. From density of modes M(E) Fig. 1k–o, we have observed the first transmission plateau weakly affected for the small deformations sample. However, progressively growing deformations induce a random fluctuation in the higher energy transmission plateau by reducing the plateau’s energy steps size following the density of state trajectories. It will mix up the various transmitting modes and wash out the clearly defined energy step size of the flat ballistic transmission modes. Therefore, it will lead to injected propagating electrons in graphene sheet with clearly defined energy that will not propagate in their own modes but have the further opportunity to interact with another mode through newly available deformed state and gain or release energy and transit between different conducting modes. It will provide them access to the new density of state to scattered further before reaching to drain contact from source contact. This verticle energy coupling will give rise to a phase incoherence in the ballistic regime. Overall electrical conductance in an energy window will degrade with increasing deformed samples from the idealistic flat scenario. First, the extra density of the state gives rise to an additional scattering probability, and second these energies mix up of different transmitting modes in the corresponding window. Moreover, degradation from ideal characteristics and fluctuation in density of state and modes density is more severe in the high-energy windows than in the low-energy region, and this trend is more severe for larger corrugated devices. Phenomenologically corrugated deform graphene sheet can be interpreted as a flat graphene sheet with local defect and scattering center at each site and delocalized spread across the sheet. Deformation will introduce variation in density of state and transmission modes density in the entire scattering wavelength spectrum from the idealized flat graphene characteristic. Therefore, high-energy electronic states with small scattering wavelengths have more variation than low energy states. In near-zero energy fields, there is less distortion for more minor deformed structures. As the deformation grows at lattice size, It also starts distorting the low energy idealize characteristic of flat graphene through a large scattering wavelength. Next, we have simulated the large 100x 100 graphene supercell to observe the above-discussed effect in greater spatial detail. The Figs. 2 and 3 correspond to 100 x 100 graphene supercell structure and correspond to the surface roughness device structure with roughness varying from 5 pm to 20 pm, mimicking the roughness profile of h-BN and $\mathrm {SiO}_{2}$ substrate. Similarly, Figs. 2a–3b represents the corresponding density of state, Figs. 2c–3d represents the electronic density of modes M(E) in the x-y direction, Figs. 2g–3h represents the electronic density, Figs. 2i–3i represents the hole density and Figs. 2e–3f represents the electronic bandstructure of the corresponding device structure. In the article’s supplementary information, from Figs. 2a–3f is also rendered in the full-text page resolution in the corresponding Supplementary Figs. 37–55 for the reader’s reference. In the simulated device, the primitive unit cell has two atoms per cell, and a total of 20000 atoms are simulated by a domain size of 80000 finite element mesh grid points. The total degree of freedom in hamiltonian is 60000 variable-sized orbitals.

The density of state from Figs. 2a–3b plotted for the larger device, and as previously discussed, we have observed density of state fluctuation; however, because of the relatively larger size in comparison to the correlation length of deformation density of state variation relatively smoothen in compare to small size cell, Also, The Van-Hove singularity in the DOS curve is not spiked to very high in the curve due to the numerical resolution of computational calculation. Moreover, the slight shift in the Dirac point in the density of state with the corrugation follows the Fermi level calculation. The effective fermi level value follows the band structure calculation. Also, the density of state shifts towards the right is due to a shift in Fermi-energy, as seen in the band structure plots and reported in Raman spectra of the experimental graphene sheet^75^. However, this shift is minuscule in comparison to a small geometry corrugated structure from Fig. 1. From electronic density Figs. 2g–3h and hole density Figs. 2i–3i we represented a real-space description of confined states concentrating in the region where the pseudo magnetic field acquires its minima or maxima. Furthermore, this will provide inhomogeneous charge redistribution in the deformed lattice. Moreover, these features are independent of the lattice orientation of the graphene sheet and within reach of detailed STM characterization of the CVD graphene sheet for industrial-scale qualitative benchmarking. The emergence of a localized and delocalized state in graphene is due to symmetry breaking in the crystal. We have observed electronic bandstructure, from Figs. 2e –3f and the electronic state gets delocalized with more roughness. However, there is no bandgap opening at the K-point for these deformations values, but it can be possible, as discussed previously. Nonetheless, we have observed that energy splitting started at $\Gamma$ and *M* point in the band structure for these deformation values. From Figs. 2c–3d, as previously discussed, the density of modes M(E) with increasing roughness corrugation states are getting delocalized and mixed up in the energy window from the flat sample. However, the density of the state smoothened for a larger sample. Still, overall transmission is decoherent because energy modes mix up and conductance decreases from the idealistic scenario. The density of modes calculation for the lower energy transmission follows the coherent transport regime. The transmission probability increases with the decreases in $\delta h$ and the scattering rate decreases. The surface corrugation profile becomes smooth with the increase of correlation length and reduces the carrier scattering rate. Fasolino *et al.* perform Metropolis algorithm-based Monte-Carlo simulations for intrinsic ripples in graphene with varying samples size of atoms from N = 240 to 19940^76^. For varying samples size at T = 300K, they found the ripples latent size of 5 to 10 nm long matching with the experiment values^77^. In our work from NEGF calculation from comparing Figs. 2g–j and 3g–i on 25nm X 35nm graphene sheet, we have observed comparable ripples latent size with fluctuating electron-hole charge density puddle in our NEGF calculation.

We have also simulated a two-dimensional orthorhombic device structure to investigate whether a four atom per unit supercell will influence similarly or have different characteristics. This geometrical structure can be further tailored to make an armchair and zigzag nanoribbon configuration based upon confinement direction, boundary condition, and edge topology. In the article’s supplementary information, simulation results are rendered in the full-text page resolution from Supplementary Figs. 56–80 for the reader’s reference. We have observed a similar characteristic in the orthorhombic device structure as in the primitive device. The surface roughness in the corrugated device will shift the Fermi energy level from zero and a shift in the high symmetric points of the Brillouin zone of the ideal graphene sheet as reported in the experimental study of the twisted graphene sheet^75^. The In-plane deformation profile also influences the electronic properties of the narrow-width graphene device and graphene nanoribbon. However, for the wide graphene sheet device on the substrates, the surface corrugation will always impact and fluctuate the device performance characteristics. Furthermore, in the corrugated device structure, the electron density distribution is nonuniform in the sheet implies that scattering rate and carrier conductivity are also spatially varying phenomena in the device. A highly corrugated device will complicate the band diagram with the mixing and split of various branches. In the corrugated device structure, we have observed the density fluctuation with the increase of corrugation compared to the flat configuration. In addition, the local density of state variation suggests that the scattering probability should be in the spatial domain, momentum-dependent, and energy-dependent. Moreover, the current path will be highly disordered and nonlinear in such a device and follow the minimum resistance path. In the corrugated structure, density variations from the average density valuation in the flat configuration arise due to random quantum fluctuation due to variable atomic bond length and bond angle in the microscopic description of the material at the atomistic scale, and inversion symmetry and lattice symmetry breaking. These effects influence the macroscopic electronic and charge transport properties of the sample.

### In-plane deformation roughness effect

We have investigated the in-plane deformation effect on the electronics properties of the graphene sheet. In-plane, inhomogeneous deformation acts as a scattering source and affects the graphene sheet’s transport property^78^. Auto-correlation function is used to statistically model the in-plane deformation effects^79^. In the transport direction of *z*, the graphene sheet has a uniform ideal width of *W*. At position $z_{1}$ and $z_{2}$, the width deviates from the ideal width by $\delta W(z_{1})$ and $\delta W(z_{2})$ respectively. By auto-correlation function the correlation between $\delta W(z_{1})$ and $\delta W(z_{2})$ is defined as,

15 $$\begin{aligned} R(z_{1}, z_{2})=\big \langle \delta W(z_{1})\delta W(z_{2})\big \rangle . \end{aligned}$$

By using the argument of correlation function $R (z_{1},\ z_{2})=R(z_{1}-z_{2})$ and taking the Fourier transform of auto-correlation function the power spectral density is,

16 $$\begin{aligned} R(q)=\int \mathrm {d}zR(z)\exp (-\mathrm {i}qz) . \end{aligned}$$

The graphene sheet in-plane roughness profile is approximated as Gaussian or exponential nature^80^. By using the exponential nature of the auto-correlation function,

17 $$\begin{aligned} R(z)=W_{rms}^{2}\exp \Bigg [-\frac{|z|}{l_c}\Bigg ] \end{aligned}$$

where $W_{rms}$ is fluctuation amplitude root mean square. Smoothness of the edge is measure by roughness correlation length $l_c$ and $z=n\delta z$ is total sampling length divide into $\delta z =a_{cc}/2$ sampling interval, and $a_{cc}$ is carbon-carbon bond length. By using Eq. (17) in the (16) and taking the Fourier transformation, in real space in-plane deformation corrugation defined as the auto-correlation power spectrum is,

18 $$\begin{aligned} R(q)=\frac{W_{rms}^{2}l_c}{1+q^{2}l_c^{2}}. \end{aligned}$$

Finally, the real space roughness is calculated by applying a random phase to Eq. (18) and taking inverse Fourier transformation^81^. Using NEGF formalism, we have created various numerical roughness profiles and evaluated the graphene sheet’s electronic transport properties.

We will discuss the results of 10x10 graphene supercell Fig. 4a–b, correspond to the in-plane deformation roughness device structure with roughness varying from 5 pm to 10 pm, mimicking the in-plane deformation profile of h-BN and $\mathrm {SiO}_{2}$ substrate. Similarly, Fig. 4i–j represents the corresponding density of state, Fig. 4c–d represents the electronic density of modes M(E) in the x-y direction, Fig. 4e–f represents the electronic density, Fig. 4g–h represents the hole density and Fig. 4k–l represents the electronic bandstructure of the corresponding in-plane deformation roughness device structure. In the article’s Supplementary information, from Fig. 4a–l is also rendered in the full-text page resolution in the corresponding Supplementary Figs. 81–92 for the reader’s reference. In all, the in-plane deformation roughness structure correlation is kept constant at 10nm length.

From Fig. 4, we have observed that an increase in the in-plane deformation from Fig. 4i 5 pm to Fig. 4j 10 pm, the density of state fluctuate more from the ideal flat structure of Fig. 1f, however, in comparison to corresponding surface roughness structures distortion in the density of the state is more diminutive. Also, from Fig. 4c–d, we have observed that an increase in the in-plane deformation will mix up more energy modes progressively by reducing the energy plateau and reduce the average transmission probability by providing an extra state to scatter. Furthermore, from Fig. 4e–l we have observed that increasing in-plane deformation will fluctuate electron-hole density in the sample. The correlation length of deformation will also change the localization length scale to mean free path for transport. Furthermore, depending upon the correlation length, more modes can mix up in a specific energy window of transport^82^. Also, the modest increase in deformation amplitude will introduce these localized states, and transmission current through these new states will progress^83^. However, a radical increase in the in-plane deformation will effectively decrease the sheet width available for propagating modes, increase the transport bandgap, and decrease the current value. One of the main contributions of roughness scattering through non-equilibrium Green’s functions formalism (NEGF) transport is that we have shown the corrugations scatterers is a long-range scattering potential, as depicted in Figs. 2g–j and 3g–i on 25nm X 35nm sheet with varying corrugations from 5 pm to 20 pm. Furthermore, with the increasing strength of corrugations, the scatterer’s potential heavily disturbs graphene’s ideal electronic transport properties. In out-of-plane roughness due to the fluctuating scattering potential distribution, it creates electron-hole puddles in the sheet; further in-plane roughness is not so dominant to perturb the ideal graphene’s transport as discussed above. Furthermore, to overcome these effects, graphene’s conductivity increases by intentional doping, but it will lower mobility. Also, conductivity is enhanced by using multiple graphene layers, but it will trade-off with the lower optical transmission in the device, restricting its application as the transparent electrode. In the in-plane deformation roughness effect, we have observed that only the carbon-carbon bond length is modified. However, in the surface roughness effect, both carbon-carbon bond length and bond angle are simultaneously randomized due to corrugation in the substrate. Therefore, the electronic band structure and other transport properties are more sensitive to these surface fluctuations and deviate further from the idealized flat graphene sheet.

### Impurities effect

We have also investigated the effect of unwanted impurities on the electronic properties of the graphene sheet to elucidate the non-idealities that arrive during the fabrication and transfer step in a natural CVD-grown graphene sheet. Nitrogen and Phosphorus impurity atoms numerically substitute on the graphene sheet with corresponding bond radius and excess charge of the impurity atom. In the realistic scenario, numerous methods may arise in spatial charge distribution fluctuation in the graphene sheet. For example, an inhomogeneous electrical field applies at the gate or source/drain contact in the hall device through bias voltages is one such feasibility. Also, an inhomogeneous spatial distribution of impurity atoms in the sheet or below in the substrate or an inhomogeneous strain field due to inherent ripple of flake and topological morphology of underline substrate or overlapping encapsulation may introduce these effects. Ziegler *et al.* mathematically formulated the impurity scattering using linear response theory Kubo formalism. Furthermore, gauged robust minimal conductivity independent of disorder of sample in the Boltzmann conductivity limit by applying mean-field approximation to the Kubo conductivity tensor in a weak scattering regime exploiting the liner Dirac electronic bandstructure of graphene^84^. However, our simulation work is based upon the Landauer formula in the non-equilibrium Green’s functions formalism, which is more generic to handle linear as well as non-equilibrium transport regimes and derived through Keldysh’s generalized perturbation theory for the non-equilibrium system. In the non-equilibrium Green’s functions formalism, the generic interacting current ansatz has been given by Caroli et al.^85–87^ from the fluctuating electronic density between the interacting contact, and it corresponds to equation (5) of the Landauer formula for the current through an interacting electron region^88^. We have numerically incorporated the spatial distribution of impurities atoms in graphene by solving the Poisson equation of spatially fluctuating impurities potential with NEGF formalism. Henceforward, we calculate the spatial charge distribution in terms of density of state, electronic and hole density, and variation in transmitting density of modes. Furthermore, we have investigated the change in electronic properties with the interpretation of impurity density variation. We have varied the number of carbon atoms involving a substitutional impurity atom to consider these effects, effectively changing the device’s doping density. Furthermore, We have changed the impurity atom type, e.g., Nitrogen and Phosphorous atoms, with varying atomic radius. Besides, in the Phosphorous impurity study, we intentionally change the electronic valency of the ion with ten electronic charges. This fictitious super ion configuration is simulated to mimic a larger diameter atomic or molecular defect with the high electronic charge concentrated at a particular lattice site on graphene sheet in the substitutional or adsorbent configuration pattern during the fabrication or transfer step. To circumvent the high computational cost of non-local scattering self-energy term in the NEGF framework. Adsorbed or substrate charged impurities trap are modeled in the graphene sheet as charge impurity center and truncated at the $U_0$ screened Coulomb potential energy superimposed upon the potential energy profile of impurity-free graphene sheet. Furthermore, the potential profile outside the screening length of impurity charges does not vary significantly from the impurity-free device configuration as,

19 $$\begin{aligned}&{\Phi (\varvec{r})=\frac{Q}{4\pi \varepsilon \varvec{r}}\exp ^{-\frac{\varvec{r}-\varvec{r}_0}{\Lambda _D}}\ ;\ \varvec{r}\ne \varvec{r}_0}, \end{aligned}$$

20 $$\begin{aligned}&{\Phi (\varvec{r})=U_0 \ ;\ \varvec{r}=\varvec{r}_0} \end{aligned}$$

where $\varvec{r}$ is the position of the current atom, $\Phi (\varvec{r})$ is the screened Coulomb potential, *Q* is element charge, and $\varvec{r}_0$ is the position of the superimposed impurity atom, $\varepsilon$ is the dielectric constant, $U_0$ onsite core potential modified to adjust impurity atom state energy, and $\Lambda _D$ Debye screening length,

21 $$\begin{aligned} \Lambda _D=\sqrt{\frac{\varepsilon kT}{Q^2N_d}} \end{aligned}$$

where *T* is temperature, *k* is the Boltzmann constant, and $N_d$ is the impurity doping concentration. The charge placement can be done randomly and explicitly at a particular atomic location with a random dopant distribution, sampling, and determining a stochastic model. With the added computation overhead, charge impurities can also be added as a scattering self-energy similar to an explicit way without requiring a sampling step in the NEGF coupled Eq. (7) of motion. However, the convergence achieves fast in the self-consistent born calculation with Poisson’s equation in a stochastic scheme. As the electrons and holes occupy low energy bands, long-range impurity potential interacts more critically with the device’s long-range externally applied electrostatic potential. Therefore from a theoretical perspective in the NEGF framework, a more rigorous model with multiple impurity scattering, impurity-impurity interaction, and interaction with the device’s electrostatic potential should be incorporated for better accuracy and added computational cost. Previously, Chen et al. demonstrated in-situ controlled potassium doping on pristine graphene devices in an ultrahigh vacuum chamber at a low T = 20 K temperature and measured gate-voltage-dependent conductivity, and investigated carrier density-dependent conductivity near Dirac-cone^89^. In our impurities, NEGF calculation similarly minimum conductivity value is observed. However, our calculation scenario is more complex. We calculate the transmission at room temperature and mix up all the propagating modes at thermal energy as it happens in the room temperature measurement in the CVD graphene sheet for any practical application. We have calculated the Density of state from Eqs. (8) and (19), electronic density and hole density from Eqs. (10) and (19), density of propagating modes M(E) in x-, y-direction from Eqs. (13) and (19), Electronic band structure from Eqs. (7) and (19), Spatially resolved electronic state and self-consistent Poisson potential from Eqs. (19) and (21) on a graphene sheet with Nitrogen and Phosphorus atom substitute impurity in the Figs. 5, 6 and 8, 9 respectively for the various supercell size to investigate the effect of impurity density by varying the supercell size. As observed in the CVD-grown graphene samples, the doping density varied batch to batch based on the recipe. To investigate the correlation between the level of impurity effect on the relative size of the graphene sheet, we have used a single impurity with varying sizes of graphene structure. Next in the figure Fig. 5, Fig. 5a correspond to bigger 20x20 graphene supercell flat structure with substitutional Nitrogen impurity, fig. 5b corresponding density of state, Fig. 5c electronic band structure, Fig. 5d density of modes, fig. 5e self-consistent Poisson potential due to Nitrogen impurity, Fig. 5f electronic density, Fig. 5g hole density. In the article’s supplementary information, from Fig. 5a–g is also rendered in the full-text page resolution in the corresponding figures of Figs. 93–99 for the reader’s reference. Also, spatially resolved electronic orbital wave-function amplitude $|\psi |^{2}$ for first seven eigenvalues of stationary solution of Schrödinger equation from $|\psi _{0}|^{2}$ to $|\psi _{6}|^{2}$ in the corresponding graphene supercell is represented in the article’s supplementary information from Supplementary Figs. 100–106. In the simulated device, the primitive unit cell has two atoms per cell, and a total of 800 atoms are simulated with a domain size of 3200 finite element mesh grid points. The total degree of freedom in hamiltonian is 2400 variable-sized orbitals.

In Fig. 5, from Fig. 5b, we have observed that Nitrogen impurity will induce additional state and density of state fluctuation and hence provide more scattering probability to propagating electron in a graphene sheet. Also, from Fig. 5c there is a small energy bandgap opening in the band structure and change in dispersion curvature of Dirac fermion and a dip in Fermi level due to additional Nitrogen electronic charge in sheet. Furthermore, from Fig. 5d there is a more density of modes mixing up in comparison to idealistic characteristics as distinct energy level plateaus are vanishing. Moreover, from Fig. 5f–g electronic and hole density significantly fluctuate from the constant neutral density due to impurity induced self-consistent Poisson potential of 0.3 eV at the impurity site and spread in the vicinity of next to the next nearest neighbor in the lattice as observed in Fig. 5e. Next in the figure Fig. 6, Fig. 6a correspond to biggest 50x50 graphene supercell flat structure with substitutional Nitrogen impurity, Fig. 6b corresponding density of state, Fig. 6c electronic band structure, Fig. 6d density of modes, Fig. 6e self-consistent Poisson potential due to Nitrogen impurity, fig. 6f electronic density, Fig. 6g hole density. In the article’s supplementary information, from Fig. 6a to Fg. 6g is also rendered in the full-text page resolution in the corresponding figures of Figs. 107–113 for the reader’s reference. Also, spatially resolved electronic orbital wave-function amplitude $|\psi |^{2}$ for first seven eigenvalues of stationary solution of Schrödinger equation from $|\psi _{0}|^{2}$ to $|\psi _{6}|^{2}$ in the corresponding graphene supercell is represented in the article’s supplementary information from Supplementary Fig. 114–120. In the simulated device, the primitive unit cell has two atoms per cell, and a total of 5000 atoms are simulated with a domain size of 20000 finite element mesh grid points. The total degree of freedom in hamiltonian is 15000 variable-sized orbitals.

In Fig. 6, from Fig. 6b, we have also observed that Nitrogen impurity will induce additional state and density of state fluctuation and hence provide more scattering probability to propagating electron in a graphene sheet; however, the severity of distortion is less in comparison to the smaller geometry case as discussed previously. Also, from Fig. 6c there is a restoration of the Dirac fermion band structure, and from Fig. 6d However, due to additional availability of state, there is a still density of modes mixing up in comparison to idealistic characteristics as distinct energy level plateau are fading. However, distortion is less in comparison to the smaller geometry case of Fig. 5. Moreover, from Fig. 6f–g electronic and hole density significantly fluctuate from the constant neutral density due to impurity induced self-consistent Poisson potential of 0.3 eV at the impurity site and spread in the vicinity of next to the next nearest neighbor in the lattice as observed in surface projected Poisson potential landscape plot of Fig. 6e. Also, in comparison to Fig. 5 from Fig. 6, we have observed that a single impurity state in the larger device does not affect the energy band edge significantly on the energy scale. Also, it induces insignificant energy broadening compared to contact induces energy broadening as the system is in a non-equilibrium open quantum state. The weak perturbation effect of a single impurity state validates these estimates. Therefore, the device’s electrostatic potential landscape did not further calibrate for this new impurity state induces a potential loop, and overall propagating states are unimpaired by this variation. However, such a numerical truncation situation rapidly changes for a smaller device geometry with a single impurity state or a larger device with multiple impurity states. Therefore, a more detailed self-consistent calculation incorporating an impurity potential loop is required to reach higher accuracy. Next, we have doped the corrugated graphene sheet of 100x100 supercell with substitutional Nitrogen impurities with varying corrugation from 5 pm to 20 pm in the Fig. 7 to investigate the combined effect of impurities atom and out of plane corrugation. We have substituted the 12 carbon atoms with the Nitrogen at the atomic location numbers 2972, 3565, 7129, 7418, 8127, 9101, 9178, 11032, 11692, 11707, 16757, 17155 in a graphene sheet constituting 20000 atoms carved out of 25nm x 40 nm flake. From Fig. 7, we observed that the transmission profile and atomistic electronic density were disturbed significantly by the dual effect from the flat idealistic and uniform charge distribution graphene sheet and the formation of electronic-hole puddle formation in the graphene flake. In the article’s supplementary information, from Fig. 7b–i is also rendered in the full-text page resolution in the corresponding figures of Figs. 121–132 for the reader’s reference. In the Fig. 7 with the progressively increasing corrugation profile with impurities atoms doping in the large 100x100 graphene supercell from corrugate-5pm Fig. 7b to 20pm Fig. 7e, We have observed the electronic density of transmission modes M(E) in x-y direction heavily distorted from the idealistic condition. Moreover, the electronic density for corrugate-5pm Fig. 7f to 20pm Fig. 7i, also fluctuated, as also seen in Fig. 7j view.

The impurity atoms, inhomogeneous impinging applied electrical field, and various strain fields through ripples, corrugation in the graphene sheet increase the energy mixing of propagating density of modes. Furthermore, the extra state density assists in propagating electrons to scatter more, therefore reducing overall conductance. Nevertheless, at the same time, even the slight variation in band structure dispersion gives rise to variation in the effective mass of Dirac fermion from idealistic values, and overall observed mobility and conductivity deteriorate furthermore from the flat characteristic. We have observed the slight kink in the density of state around zero-energy Dirac point due to substitutional impurity and the start of band branch splitting around the high symmetric points of the Brillouin zone, which broadens the original graphene band at these points. These additional band branches will influence the energy landscape in the next few nearest-neighbor carbon atoms. Hence, more accuracy can be achieved in band structure calculation by adopting a higher-order tight-binding model for an impurity-driven sheet in an open boundary device or using an electrically close boundary sheet in the ab-initio calculation with few ten of thousand atoms device with additional computation overhead. Next, we have a doped graphene sheet with substitutional Phosphorus impurity. Although intentionally replace the electronic valency of the ion with ten electronic charges to create a fictitious super ion to mimic a larger diameter atomic or molecular defect with the high electric charge concentrated at a specific lattice site on the graphene sheet. In the figure Fig. 8, Fig. 8a correspond to 20 x 20 graphene supercell flat structure with substitutional Phosphorus impurity, Fig. 8b corresponding density of state, Fig. 8c electronic band structure, Fig. 8d density of modes, Fig. 8e self-consistent Poisson potential due to Phosphorus impurity, Fig. 8f electronic density. In the article’s supplementary information, from fig. 8a–f is also rendered in the full-text page resolution in the corresponding Supplementary Figs. 133–138 for the reader’s reference. Also, spatially resolved electronic orbital wave-function amplitude $|\psi |^{2}$ for first seven eigenvalues of stationary solution of Schrödinger equation from $|\psi _{0}|^{2}$ to $|\psi _{6}|^{2}$ in the corresponding graphene supercell is represented in the article’s supplementary information figures from Figs. 139–145.

In Fig. 8, from Fig. 8b, we have observed that Phosphorus impurity with excessive charge will induce extra state density and hence provide more scattering probability to propagating electrons in a 20x20 graphene sheet. The density of state shifted significantly towards the left due to shifting in Fermi value due to excessive electronic impurity charge doped in this sample; a similar trend is visible in the band structure plot from Fig. 8c. There is a tiny band opening in the band structure, and the dispersion of graphene has heavily deteriorated from Dirac fermion. From Fig. 8d there is a more density of modes mixing up in comparison to idealistic characteristics as distinct energy level plateau dissolved. Moreover, from Fig. 8f electronic density significantly fluctuates from the constant neutral density due to impurity induced self-consistent Poisson potential of 0.85 eV at the impurity site and spread in the vicinity of next to the next nearest neighbor in the lattice as observed in surface projected Poisson potential landscape plot of Fig. 8e. The increase in the Poisson potential in this case as compared to Figs. 5e–6e is due to the excess charge we intentionally doped at a lattice site to investigate the effect of a heavily contaminated site in a CVD Graphene sheet. Next, in the Fig. 9, Fig. 9a correspond to 50 x 50 graphene supercell flat structure with substitutional Phosphorus impurity, Fig. 9b corresponding density of state, Fig. 9c electronic band structure, Fig. 9d density of modes, fig. 9e self-consistent Poisson potential due to Phosphorus impurity, Fig. 9f electronic density. In the article’s supplementary information, from Fig. 9a–f is also rendered in the full-text page resolution in the corresponding figures of Figs. 146–151 for the reader’s reference. Also, spatially resolved electronic orbital wave-function amplitude $|\psi |^{2}$ for first seven eigenvalues of stationary solution of Schrödinger equation from $|\psi _{0}|^{2}$ to $|\psi _{6}|^{2}$ in the corresponding graphene supercell is represented in the article’s supplementary information Supplementary Fig. 152–158.

In Fig. 9, from Fig. 9b, we have observed that Phosphorus impurity with excessive charge will induce extra state density kink and hence provide more scattering probability to propagating electron in a 50x50 graphene sheet. However, again in comparison to Fig. 8b fluctuation in density of state of becoming smooth. The density of state shifted towards the left due to shifting in Fermi value because of excessive electronic impurity charge in the sample. In the band structure plot from Fig. 9c. There is a mixing of states, and the dispersion of graphene has heavily deteriorated. From Fig. 9d there is a more density of modes mixing up in comparison to idealistic characteristics as distinct energy level plateau dissolved. However, the situation improves from the smaller sample calculation with the same number of impurity charges. Moreover, from Fig. 9f electronic density significantly fluctuates from the constant neutral density due to impurity induced self-consistent Poisson potential as observed in Fig. 9e, however again, the fluctuation in electronic density reduce in comparison to small geometry sample as in Fig. 8e. The phosphorus atom, due to its relative atomic size, bond radius, excess orbitals, and additional states compared to the carbon atom, the impurity effect spread in the graphene lattice. Also, these excessive impurity state influences the energy landscape in a couple of next nearest-neighbor carbon atoms. These excess impurity charges are included in numerical simulation by replacing the carbon atom with Nitrogen and Phosphorus atom with an adequate charge. From the above observation, we conclude that the enhancement of conductivity of graphene sheet through the substitutional and adsorbent impurity doping route will come with its tradeoff cost of the enhancement of additional density of states, deterioration of Dirac band dispersion, and fluctuation of transmission and modes density. Therefore reduce mobility values in the sheet. On the other hand, enhancement of conductivity of graphene by depositing multiple sheets in the device will reduce the transparency of the sheet and its possible application in display devices. Moreover, the enhancement of conductivity of graphene sheet through indirect induce doping by gate field will trade off with Fermi level shift and shift in conduction from valance band branch to conduction band branch. Furthermore, giving rise to ambipolar characteristics, and turning off such device by gate filed, especially in transistor application, will be difficult. The combined impact of corrugations and impurities may arise in practical fabrication and transfer processes. The practical impact of various nanoscale atomistic scattering mechanisms in the semiconductor consider by Matthiessen’s rule and total scattering length and time is the summation of all the underline individual effects^53,90–92^, Furthermore, the most prominent mechanisms of these various physical effects determine the final device characteristic. In the NEGF loop, these various mechanisms are adopted as Dyson’s self-energy loop to solve self-consistently. The macroscopic sample’s characterization properties are defined by the microscopic averaging out of competing scattering statistics rather than individual scattering events of the order of the femtosecond process. Finally, we conclude the article with a summary of the results and observations.

## Summary

We have numerically investigated various random, non-idealities, e.g., inhomogeneous out-of-plane surface corrugation, in-plane deformation, and excess atomistic charge impurities in the 2-dimensional graphene sheet. These non-idealities primarily arise in the Roll-to-Roll chemical vapor deposition (CVD) and plasma-enhanced chemical vapor deposition (PECVD) manufacture process. Mitigating these variances is necessary for the practical application success of the graphene industry, and the high fidelity and uniformity require in the graphene production, transfer, benchmark process is the utmost requirement for developing the graphene-based industry. We have employed a multiscale, multi-physics-based non-equilibrium Green’s function framework in a third nearest-neighbor tight-binding configuration. The Tight-binding model is computationally efficient to incorporate atomistic effects on mesoscopic electrical properties. Our detailed study provides an essential understanding for evaluating and benchmarking the electronic properties of graphene sheets. It also oversees the nanoelectronics device design aspect on graphene and 2-D material for practical application in industry. Our calculation has not explicitly treated surface roughness scattering as a self-energy term in the NEGF framework, which is numerically cumbersome, affecting the device’s performance. In future investigations, we will attempt to incorporate that in an equal mathematical footing in the framework. Also, a more sophisticated local density of state-dependent scattering model should be employed for a more detailed investigation of the highly corrugated device. Also, surface passivation treatment can influence the device’s electrical characteristics. Theoretical modeling of capping material effect, doping efficiency, long-range electrostatic screening, interface effect, interlayer phenomena, contact quality, and heat transfer in the low-dimensional material-based device should be examined further for quantitative evaluation. However, scattering is omitted and treated as residue in the most published articles in the 2-D materials domain and ignored without sufficient justification. In addition, the atomistic resolutions are critical for the charge in the self-consist computations. The theoretical treatment of defect situations is even challenging at the multiscale simulation. However, these are challenging transport situations at the nanoscale’s theoretical modeling in the graphene and 2-D material domain.

## Supplementary Information

Supplementary Information.
