# Supplementary material for: Multi-scale electronics transport properties in non-ideal CVD graphene sheet
Source: Sci Rep. 2022 Jul 2;12:11214. doi: 10.1038/s41598-022-15098-6 (PMC9250536; doi:10.1038/s41598-022-15098-6)
Supplement: Supplementary file 1 — Supplementary Information. [file 41598_2022_15098_MOESM1_ESM.pdf]

Supplementary Information

Multi-scale electronics transport properties in non-ideal CVD graphene sheet

Bhupesh Bishnoi[1,†], Marius Buerkle[1,‡], and Hisao Nakamura[1]

[1]National Institute of Advanced Industrial Science and Technology (AIST), Research Center for Computational Design of Advanced Functional Materials (CD-FMat), Central 2, Umezono 1-1-1, Tsukuba, Ibaraki 305-8568, Japan
[†]bishnoi.bhupesh@aist.go.jp
[‡]marius.buerkle@aist.go.jp

ABSTRACT

The complete evolutionary derivation of the theoretical framework of Non-equilibrium Green's function formalism with original reference is presented here, used in the main article for the reader's reference. Also, all the figures of the main article are included here in the full-text page resolution for reference. The x, y, and z-dimension scales in all the figures are in the nanometer scale resolution.

The complete evolutionary derivation of the theoretical framework of Non-equilibrium Green's function formalism with original reference is presented here, used in the main article for the reader's reference.

Non-equilibrium Green's function Formalism

Keldysh, Kadanoff, and Baym developed the non-equilibrium Green's function formalism (NEGF) in 1960.[1,2] The adaption of NEGF formalism to semiconductor devices was first demonstrated by Lake and Datta in 1992[3,4] and later in 2002 by Wacker.[5] Through NEGF formalism, the time evolution of many-body quantum fields in thermodynamic equilibrium and non-equilibrium is investigated.[4,6–10] These quantum fields are constituted by carriers such as electrons, phonons, and spin in semiconductor devices. In the NEGF formalism, The Schrödinger-Poisson equation is solved with the open boundary conditions under the non-equilibrium, Fermi contact potentials with the coupling to the contacts and energy dissipative scattering processes.[4,11–20] By this formalism, various quantum mechanical effects such as quantum mechanical tunneling, quantization of density of states, quantum mechanical transmission, reflections, and resonance states, discretization of energy levels due to spatial confinement, metal-induced gap states, edge states effects investigated. Furthermore, we can also incorporate various scattering mechanisms such as electron-electron scattering, electron-phonon scattering in the formalism. In the Schrödinger representation of the quantum system, the ground state $|\Psi_0\rangle$ of an interacting device is determined by the time-independent H Hamiltonian,

$$H|\Psi_0\rangle = E|\Psi_0\rangle \tag{1}$$

Where Hamilton operator $H = H_0 + V$ constitutes a non-interacting Hamiltonian H_0 part and V an interacting perturbation part. H_0 solved exactly with its eigenvalues and eigenvectors as a solution. The solution of Schrödinger equation gives the time dependence of Schrödinger wave-functions $|\Psi_S(t)\rangle$. The interacting part contains all the many-body effects, e.g., phonon-carrier, ionized dopant, impurity atom, carrier-carrier interaction, interface roughness effects, and evaluated by the respective self-energy calculation. The interaction part V is treated as a perturbation of system H as both wave-functions and operators are time-dependent in the interaction picture. In the alternative Heisenberg representation, the interacting quantum mechanical system is solved using the operator's time-dependent property while the wave functions are now time-independent. The time-dependent Heisenberg $\hat{\psi}_H^\dagger(x',t')$ and $\hat{\psi}_H(x,t)$ evolve as per

$$\hat{\psi}_H(x,t) = e^{iHt/\hbar}\hat{\psi}(x)e^{-iHt/\hbar} \tag{2}$$

Where $\hat{\psi}(x)$ destroy a fermion at place x, In the second quantization language, hamiltonian in the real space representation

presents a relatively intuitive picture

$$\hat{\psi}(x) = \sum_k \phi_k(x) c_k \tag{3}$$

Where $c_k^\dagger$ is the creation and c_k is the annihilation operators of a fermion in orbital state ϕ_k.

Such a quantum interaction picture is described by one-particle Green's functions in an equilibrium system. The causal/time-ordered zero-temperature single-fermion Green's function is defined as,[20,21]

$$G(x,t;x',t') = -\frac{i}{\hbar} \frac{\left\langle \Psi_0 \middle| T^c \{ \hat{\psi}_H(x,t) \hat{\psi}_H^\dagger(x',t') \} \middle| \Psi_0 \right\rangle}{\left\langle \Psi_0 \middle| \Psi_0 \right\rangle} \tag{4}$$

Where a time-ordering operator T^c moves the earlier time-argument to the right, and the resulting expression changes its sign whenever two fermion operators interchanged. By inserting eq. (2) into eq. (4), the Green's functions physical interpretation is defined as,[22]

$$G(x,t;x',t') = -\frac{i}{\hbar} \frac{\left\langle \Psi_0 \middle| T^c \{ e^{iHt/\hbar} \hat{\psi}(x) e^{-iH(t-t')/\hbar} \hat{\psi}^\dagger(x') e^{-iHt'/\hbar} \} \middle| \Psi_0 \right\rangle}{\left\langle \Psi_0 \middle| \Psi_0 \right\rangle} \tag{5}$$

The phenomenological explanation of the above equation interpreted as for the $t > t'$, The probability that a fermion created in the quantum system at time t' and place x' in real space moves to another time t and another place x, is represented by Green's function $G(x,t;x',t')$. $G(x,t;x',t')$ is defined at initial zero-time, quantum system is in ground state $|\Psi_0\rangle$ after that to a time t' system evolves with the factor $e^{-iHt/\hbar}$. At reaching the place x', at that time t', $\hat{\psi}^\dagger(x')$ a fermion is created. Next, from time t' to t, system continuously evolving with a factor $e^{-iH(t-t')/\hbar}$. After reaching at place x, fermion is destroyed by annihilation $\hat{\psi}(x)$ and system return to $\langle\Psi_0|$ initial ground state by evolving $e^{iHt/\hbar}$. For the $t' > t$ contrary process, hold true. The ground state expectation value represents by a bracket $\langle\cdots\rangle$ at the zero temperature. At the finite non-zero temperature, the quantum system is no longer in the ground state. Therefore bracket $\langle\cdots\rangle$ represents the grand canonical ensemble of thermodynamic average. The quantum device is in contact with a reservoir with a temperature T, and with the reservoir, the device might exchange heat and fermions. To represent the zero and finite temperature equilibrium and non-equilibrium quantum system interaction, real-time and imaginary Green's functions and advanced G^A and retarded G^R Green's functions are defined similarly. As well to completely describe the evolution of the system, two additional Green's functions, the greater $G^>$ and the lesser $G^<$ Green's functions are also established as,[1,2,23,24]

$$\begin{aligned} G^R(x,t;x',t') &= -\frac{i}{\hbar}\theta(t-t')\left\langle \left[\hat{\psi}(x,t), \hat{\psi}^\dagger(x',t')\right]_+ \right\rangle \\ G^A(x,t;x',t') &= \frac{i}{\hbar}\theta(t'-t)\left\langle \left[\hat{\psi}(x,t), \hat{\psi}^\dagger(x',t')\right]_+ \right\rangle \end{aligned} \tag{6}$$

$$\begin{aligned} G^<(x,t;x',t') &= \frac{i}{\hbar}\left\langle \hat{\psi}^\dagger(x',t')\hat{\psi}(x,t) \right\rangle \\ G^>(x,t;x',t') &= -\frac{i}{\hbar}\left\langle \hat{\psi}(x,t)\hat{\psi}^\dagger(x',t') \right\rangle \end{aligned} \tag{7}$$

The lesser $G^<$, greater $G^>$, advanced G^A, retarded G^R, and G Green's functions are not uniquely independent but interrelated by following relationships.[1,2,23,24]

$$\begin{aligned} G(x,t;x',t') &= \theta(t-t')G^>(x,t;x',t') + \theta(t'-t)G^<(x,t;x',t') \\ G^{R,A}(x,t;x',t') &= \pm\theta(\pm t \mp t')\left[G^>(x,t;x',t') - G^<(x,t;x',t')\right] \\ G^R(x,t;x',t') - G^A(x,t;x',t') &= G^>(x,t;x',t') - G^<(x,t;x',t') \end{aligned} \tag{8}$$

These equalities hold for both equilibrium and non-equilibrium pictures, though the fluctuation-dissipation theorem linked all these properties in equilibrium. One subtle difference between the equilibrium and non-equilibrium pictures is in the

derivation of the perturbation assumption. In equilibrium, zero-temperature Green's functions scenario system is guaranteed to return its initial state after an asymptotically large time. However, In the non-equilibrium picture, this is not true, as at time t equal to $+\infty$, the final state will be very distinct from the initial state at time t equal to $-\infty$. Consequently, operator expectation values are built through the Feynman diagrams technique for contour integration,[25–27] and by Wick's decomposition theorem[28,29] and linked-graph theorem techniques for non-equilibrium situations.[30–32]

The definition of non-equilibrium Green's function defined as,

$$G(x,t;x',t') = -\frac{i}{\hbar}\left\langle T^c\left\{\hat{\psi}_{\mathscr{H}}(x,t)\hat{\psi}^{\dagger}_{\mathscr{H}}(x',t')\right\}\right\rangle \tag{9}$$

Where now the field operators are expressed in the Heisenberg representation as $\hat{\psi}_{\mathscr{H}}(x,t)$ and $\hat{\psi}^{\dagger}_{\mathscr{H}}(x',t')$ and are correspond to the total Hamiltonian $\mathscr{H}(t)$. By using the eq. (7), which holds for non-equilibrium situation, The non-equilibrium Green's function eq. (9) is,

$$G(x,t;x',t') = \theta(t,t')G^{>}(x,t;x',t') + \theta(t',t)G^{<}(x,t;x',t') \tag{10}$$

Where on the contour the definition of the function $\theta(t,t')$ is,

$$\theta(t,t') = \begin{cases} 0, \text{ if } t \text{ is earlier on a contour than } t' \\ 1, \text{ if } t \text{ is later on a contour than } t' \end{cases} \tag{11}$$

The system is defined by total Hamiltonian $\mathscr{H}(t)$,

$$\mathscr{H}(t) = H + H^{ext}(t) = H_0 + V + H^{ext}(t) \tag{12}$$

Where again non-interacting part of Hamiltonian is H_0, and the external perturbation $H^{ext}(t)$ tries to drive the system out of equilibrium, and carrier-carrier interactions and Poisson potential is contained in V.

The external perturbation $H^{ext}(t)$ defined as,

$$H^{ext}(t) = \int \mathrm{d}x\hat{\psi}^{\dagger}(x)U(x,t)\hat{\psi}(x) \tag{13}$$

Where the external potential due to interactions is $U(x,t)$.

When treating Green's functions in the device domain, changing the variable from time and real space basis to energy and momentum basis is convenient. The $G(x,t;x',t')$ is the functions of the continuous space variables ($\boldsymbol{r}$) and ($\boldsymbol{r'}$). Throughout the work, the bold mathematical symbol is used to represent vector position unless otherwise stated. By using the Fourier transformation, Green's functions are represented in the momentum and energy space. Furthermore, to solve for the real space finite element 2D/3D devices by Green's functions method, the mathematical equations are discretized on the grid of Green's functions $G_{nm}(tt')$. For the z-directional, current transport solution, the potential is homogeneous in the transverse x and y direction. The creation and annihilation operators $\hat{\psi}^{\dagger}(x',t')$ and $\hat{\psi}(x,t)$ from eq. (3) are expanded into a series of eigenfunctions as $G(x,t;x',t')$,[29,33]

$$G(x,t;x',t') = \sum_{n_1,m_1}\sum_{\boldsymbol{k'},\boldsymbol{k''}} \phi_{n_1,\boldsymbol{k'}}(\boldsymbol{r}) \cdot G_{n_1 m_1}(\boldsymbol{k'}\boldsymbol{k''};tt') \cdot \phi^{*}_{m_1,\boldsymbol{k''}}(\boldsymbol{r'}) \tag{14}$$

The wave functions ϕ are factorized in the transport z direction and homogeneous confined in x and y directions.

$$\phi_{\boldsymbol{k}}(\boldsymbol{r}) = \frac{1}{\sqrt{A}}e^{i\boldsymbol{k}_t\cdot\boldsymbol{r}_t}\phi_{\boldsymbol{k}_t}(z) \tag{15}$$

Where xy-surface area is A, $\boldsymbol{r}_t = (x,y)$ and $\boldsymbol{k}_t = (k_x,k_y)$. Due to x and y directions homogeneous condition, $G(x,t;x',t')$ Green's function only depend upon difference $\boldsymbol{r}_t - \boldsymbol{r}'_t$ and $\boldsymbol{k}'_t = \boldsymbol{k}''_t$. The discretized wave-functions ϕ are very localized functions. The atomic orbitals' wave function is tightly bound to the atoms and is usually assumed to be one lattice point in

the grid. In the tight-binding approximation, this is a fundamental assumption. Hence lattice wave functions expanded on the basis of atomic orbitals.[4,34] Therefore Green's function discretized as,

$$G(x,t;x',t') = \frac{1}{A}\sum_{n_1,m_1}\sum_{\boldsymbol{k}'_{\mathrm{t}}}\phi_{n_1,\boldsymbol{k}'_{\mathrm{t}}}(z)\cdot G_{n_1m_1}(\boldsymbol{k}'_{\mathrm{t}};tt')\cdot\phi^*_{m_1,\boldsymbol{k}'_{\mathrm{t}}}(z')\cdot e^{i\boldsymbol{k}'_{\mathrm{t}}\cdot(\boldsymbol{r}_{\mathrm{t}}-\boldsymbol{r}'_{\mathrm{t}})} \tag{16}$$

Self-energies for electron-phonon and other interactions can also discretize by applying similar procedures.

The time evolution of an interacting non-equilibrium quantum system is derived by solving the equations of motion of Green's functions $G(x,t;x',t')$ from the time t to the t', for the evolution of non-equilibrium Green's function $\frac{\mathrm{d}}{\mathrm{d}t'}G(x,t;x',t')$ and $\frac{\mathrm{d}}{\mathrm{d}t}G(x,t;x',t')$ is derived with respect to $G(x,t;x',t')$ as represented by eq. (9), the equation of motion for $G(x,t;x',t')$ at the time t_1 is

$$\begin{aligned}\left[i\hbar\frac{\mathrm{d}}{\mathrm{d}t_1}+\frac{\hbar^2\nabla^2(x_1,t_1)}{2m}-U(x_1,t_1)\right]G(x_1,t_1;x'_1,t'_1) &= \delta(t_1,t'_1)\delta(x_1-x'_1)+\\ &\int_C d(x_3,t_3)\Sigma^{tot}(x_1,t_1;x_3,t_3)G(x_3,t_3;x'_1,t'_1)\end{aligned} \tag{17}$$

Where $\delta(t,t') = \frac{\mathrm{d}}{\mathrm{d}t}\theta(t,t')$ and Σ^{tot} total self-energy.

Σ^{tot} is derived by decomposition of 2-fermion Green's function into single fermion Green's function via variational derivation and Wick's decomposition theorem, and it corresponds to the Dyson equations.[35–38] The computation of the self-energy matrices is based on the treatment of the Dyson equation. The solution of non-equilibrium equations of motion is achieved by dividing the contour integrals over a time-loop $\int_C$ into the time-ordered integrals. To solve these modified equations of motion, all types of $G^{R,A}$ and $G^{<,>}$ Green's functions are required. In the device simulation, the carrier densities distribution $\mathfrak{N}(x,t)$ and $\mathfrak{J}(x,t)$ the current densities are the two most important physical observable quantities and which can also be measured by various experimental technique as discussed in the previous section. By solving the equations of motion, lesser Green's functions $G^<(x,t;x',t')$ are calculated, which is only possible because Green's function $G(x_1,t_1;x_2,t_2)$ and the self-energy $\Sigma(x_1,t_1;x_2,t_2)$ has the same symmetry properties, and that is required for the calculation of carrier densities. Green's function $G(x_1,t_1;x_2,t_2)$ and the self-energy $\Sigma(x_1,t_1;x_2,t_2)$ symmetry properties evaluated by Craig ansatz et.al.[39] and proved by Danielewicz et.al.[40] for the formal solution of NEGF equations. By using the Langreth theorem, and expansion of eigenfunction, for $(t-t')$ time difference by taking the Fourier transform of the Green's functions.[21] The closed set of equations of motion is defined as,

$$\begin{aligned}ih\frac{\mathrm{d}}{\mathrm{d}t}G^<_{nm}(\boldsymbol{k}_{\mathrm{t}};tt')-\sum_l h_{nl}G^<_{lm}(\boldsymbol{k}_{\mathrm{t}};tt') &= \sum_l\int_{t_0}^{\infty}\mathrm{d}t_1\Sigma^R_{nl}(\boldsymbol{k}_{\mathrm{t}};tt_1)G^<_{lm}(\boldsymbol{k}_{\mathrm{t}};t_1t')\\ &+\sum_l\int_{t_0}^{\infty}\mathrm{d}t_1\Sigma^<_{nl}(\boldsymbol{k}_{\mathrm{t}};tt_1)G^A_{lm}(\boldsymbol{k}_{\mathrm{t}};t_1t')\end{aligned} \tag{18}$$

$$\begin{aligned}-ih\frac{\mathrm{d}}{\mathrm{d}t'}G^<_{nm}(\boldsymbol{k}_{\mathrm{t}};tt')-\sum_l G^<_{nl}(\boldsymbol{k}_{\mathrm{t}};tt')h_{lm} &= \sum_l\int_{t_0}^{\infty}\mathrm{d}t_1 G^R_{nl}(\boldsymbol{k}_{\mathrm{t}};tt_1)\Sigma^<_{lm}(\boldsymbol{k}_{\mathrm{t}};t_1t')\\ &+\sum_l\int_{t_0}^{\infty}\mathrm{d}t_1 G^<_{nl}(\boldsymbol{k}_{\mathrm{t}};tt_1)\Sigma^A_{lm}(\boldsymbol{k}_{\mathrm{t}};t_1t')\end{aligned} \tag{19}$$

Where hamiltonian is hermitian $h^*_{ml} = h_{lm}$ and $h_{nm} = \int\mathrm{d}\boldsymbol{r}\phi^*_n(\boldsymbol{r})H_0(\boldsymbol{r})\phi_m(\boldsymbol{r})$. Retarded and advanced Green's function ($G^{R,A}$), as well as lesser self-energy ($\Sigma^<$), and retarded and advanced self-energy ($\Sigma^{R,A}$) is required to solve the close-set of equations of motion eq. (18) and eq. (19). These Green's functions and self-energy are calculated by solving the equation of motion. Similarly, $R's$ replaced by $A's$, and by similarly procedure equations for G^A are obtained.

$$i\hbar\frac{\mathrm{d}}{\mathrm{d}t}G^R_{nm}(\boldsymbol{k}_{\mathrm{t}};tt')-\sum_l h_{nl}G^R_{lm}(\boldsymbol{k}_{\mathrm{t}};tt') = \delta_{nm}(tt')+\sum_l\int_{t_0}^{\infty}\mathrm{d}t_1\Sigma^R_{nl}(\boldsymbol{k}_{\mathrm{t}};tt_1)G^R_{lm}(\boldsymbol{k}_{\mathrm{t}};t_1t') \tag{20}$$

Where $\delta_{nm}(tt')$ is defined as,

$$\delta_{nm}(tt') = \sum_l\int_{t_0}^{\infty}\mathrm{d}t_1[G^R_{nl}(\boldsymbol{k}_{\mathrm{t}};tt_1)]^{-1}G^R_{lm}(\boldsymbol{k}_{\mathrm{t}};t_1t') \tag{21}$$

After simplification by Langreth theorem, The lesser Green's function, the central equation of motion is with the coupling between $G^{<}$ and $G^{R,A}$ as,

$$G^{<}_{nm}(\boldsymbol{k}_{\mathrm{t}};tt') = \sum_{l,v}\int_{t_0}^{\infty}\mathrm{d}t_1\int_{t_0}^{\infty}\mathrm{d}t_2 G^{R}_{nl}(\boldsymbol{k}_{\mathrm{t}};tt_1)\Sigma^{<}_{lv}(\boldsymbol{k}_{\mathrm{t}};t_1t_2)G^{A}_{vm}(\boldsymbol{k}_{\mathrm{t}};t_2t') \tag{22}$$

Green's functions $G_{nm}(\boldsymbol{k}_{\mathrm{t}};tt')$ usually depend upon time (t) and (t'), however once non-equilibrium system reach to a stationary state solution the Green's functions depend on the time difference $(t-t')$. As per the Langreth theorem, the system of evolution, (t) and (t') upon reaching the stationary state, no longer reside on the imaginary time contour. Furthermore, using the advantage of Fourier transform Green's functions for the time difference $(t-t')$ are modified in the energy domain. Comparable relationship holds for $G^{R,A}$ also,

$$G^{<}_{nm}(\boldsymbol{k}_{\mathrm{t}};E) = \int \mathrm{d}(t-t')e^{iE(t-t')/\hbar}G^{<}_{nm}(\boldsymbol{k}_{\mathrm{t}};t-t') \tag{23}$$

Consequently, finally, in the stationary state, equations of motion of the quantum system are simplified as follow in the coupled set of the equations to describe the NEGF formalism,

$$\begin{aligned}
EG^{<}_{nm}(\boldsymbol{k}_{\mathrm{t}};E)-\sum_{l}h_{nl}G^{<}_{lm}(\boldsymbol{k}_{\mathrm{t}};E) &= \sum_{l}\Sigma^{R}_{nl}(\boldsymbol{k}_{\mathrm{t}};E)G^{<}_{lm}(\boldsymbol{k}_{\mathrm{t}};E)+\sum_{l}\Sigma^{<}_{nl}(\boldsymbol{k}_{\mathrm{t}};E)G^{A}_{lm}(\boldsymbol{k}_{\mathrm{t}};E)\\
EG^{<}_{nm}(\boldsymbol{k}_{\mathrm{t}};E)-\sum_{l}G^{<}_{nl}(\boldsymbol{k}_{\mathrm{t}};E)h_{lm} &= \sum_{l}G^{R}_{nl}(\boldsymbol{k}_{\mathrm{t}};E)\Sigma^{<}_{lm}(\boldsymbol{k}_{\mathrm{t}};E)+\sum_{l}G^{<}_{nl}(\boldsymbol{k}_{\mathrm{t}};E)\Sigma^{A}_{lm}(\boldsymbol{k}_{\mathrm{t}};E)\\
G^{<}_{nm}(\boldsymbol{k}_{\mathrm{t}};E) &= \sum_{l,v}G^{R}_{nl}(\boldsymbol{k}_{\mathrm{t}};E)\Sigma^{<}_{lv}(\boldsymbol{k}_{\mathrm{t}};E)G^{A}_{vm}(\boldsymbol{k}_{\mathrm{t}};E)\\
G^{<}_{nm}(\boldsymbol{k}_{\mathrm{t}};E) &= -[G^{<}_{mn}(\boldsymbol{k}_{\mathrm{t}};E)]^{\dagger}\\
EG^{R}_{nm}(\boldsymbol{k}_{\mathrm{t}};E)-\sum_{l}h_{nl}G^{R}_{lm}(\boldsymbol{k}_{\mathrm{t}};E) &= \delta_{nm}+\sum_{l}\Sigma^{R}_{nl}(\boldsymbol{k}_{\mathrm{t}};E)G^{R}_{lm}(\boldsymbol{k}_{\mathrm{t}};E)\\
G^{A}_{nm}(\boldsymbol{k}_{\mathrm{t}};E) &= [G^{R}_{mn}(\boldsymbol{k}_{\mathrm{t}};E)]^{\dagger}\\
G^{R}_{mn}(\boldsymbol{k}_{\mathrm{t}};E)-G^{A}_{mn}(\boldsymbol{k}_{\mathrm{t}};E) &= G^{>}_{mn}(\boldsymbol{k}_{\mathrm{t}};E)-G^{<}_{mn}(\boldsymbol{k}_{\mathrm{t}};E)\\
A(\boldsymbol{k}_{\mathrm{t}};E) &= i\left[G^{R}_{mn}(\boldsymbol{k}_{\mathrm{t}};E)-G^{A}_{mn}(\boldsymbol{k}_{\mathrm{t}};E)\right]
\end{aligned} \tag{24}$$

Where G^{R}, $G^{<}$, and for the relevant different interactions self-energies $\Sigma^{R}_{nm}(\boldsymbol{k}_{\mathrm{t}};E)$, $\Sigma^{<}_{nm}(\boldsymbol{k}_{\mathrm{t}};E)$ calculated in coupled system of eq. (24).[4,5,20,21,41,42]

The couple set of equations is computational intensive to solve, for a device with tight-binding model Hamiltonian h, matrix element is $N_H \times N_H$, if the device contain lattice N_L points, k wavevector N_k points, and energy N_E points. The functions $G^{R}, G^{<}, \Sigma^{R}$, and $\Sigma^{<}$ size to calculate and store is $N_L \times N_L \times N_k \times N_E \times N_H \times N_H$. The size of the matrices is N x N, as N denotes the total number of atoms in the device. The retarded Green's function computed by the Recursive Green's function (RGF) algorithm of complexity $\mathscr{O}(N)$,[43–46] exploiting the property of block tri-diagonal matrix structure with minimal computational resources compared to the massive matrix inversion operation of complexity $\mathscr{O}(N^3)$. The algorithm to calculate the couple set of equation start with an initial value of $G^{<}$ and G^{R}. The system with no interactions is taken as the initial value of free Green's function G^{0R} and $G^{0<}$. For all the lattice, H E, k, points, self-energies $\Sigma^{<}$and Σ^{R} derived by calculating the actual $G^{<}$ and G^{R}. The calculation of new G^{R} and $G^{<}$ is performed by using the self-energies $\Sigma^{<}$ and Σ^{R} values from the previous iteration. This loop continues until Jacobi iterations reach convergence. With this final $G^{<}$, the actual carrier density is obtained and solved in the Poisson equation loop. The carrier-carrier interactions will be approximately treated on the mean-field level with the Hartree self-energies as part of the Poisson potential. The algorithm restarted with the newly calculated Poisson potential and continued to run till convergence was achieved. As calculating carrier density and current density is computationally expensive in Green's function formalism by parallelizing the self energies computation and neglecting some parts of the self-energies, speed up is achieved in the calculation.[4,11–15,47,48]

Result

All the figures of the main article are included here in the full-text page resolution for reference. The x, y, and z-dimension scales in all the figures are in the nanometer scale resolution. Following the quantum chemistry field conventions, the device

structures are mapped and indicated as the multiplication of unit cells dimension. Therefore, similar to the main article, we omit x, y, and z-dimension scales in nanometer to avoid excessive cluttering in the figures throughout in the supplementary information for the corresponding figures from fig. 1 to fig. 158.

Surface Corrugation Effect

10x10 graphene supercell

The fig. 1 correspond to 10x10 graphene supercell flat structure, and fig. 2 to fig. 5 correspond to the corrugate device structure with roughness varying from 5 pm to 20 pm, mimicking the roughness profile of h-BN and SiO_2 substrate. Similarly, fig. 6 to fig. 10 represents the corresponding density of state, fig. 11 to fig. 15 represents the electronic density of mode M(E) in the x-y direction, fig. 16 to fig. 20 represents the electronic density, fig. 21 to fig. 25 represents the hole density and fig. 26 to fig. 30 represents the electronic bandstructure of the corresponding device structure. In all, the corrugate structure correlation is kept constant at 10nm length. In the simulated device, the primitive unit cell has two atoms per cell, and a total of 200 atoms are simulated by a finite element mesh of 800 point Density of Mode size. The P-D tight-binding model contains three orbitals, namely carbon P_z, and carbon-hydrogen passivated D_{yz}, D_{xz} orbitals. Therefore total degree of free density of Mode in hamiltonian is 600 variable-sized. The K' and M' are high symmetric point that corresponds to the folded reduced BZ-zone of graphene supercell.

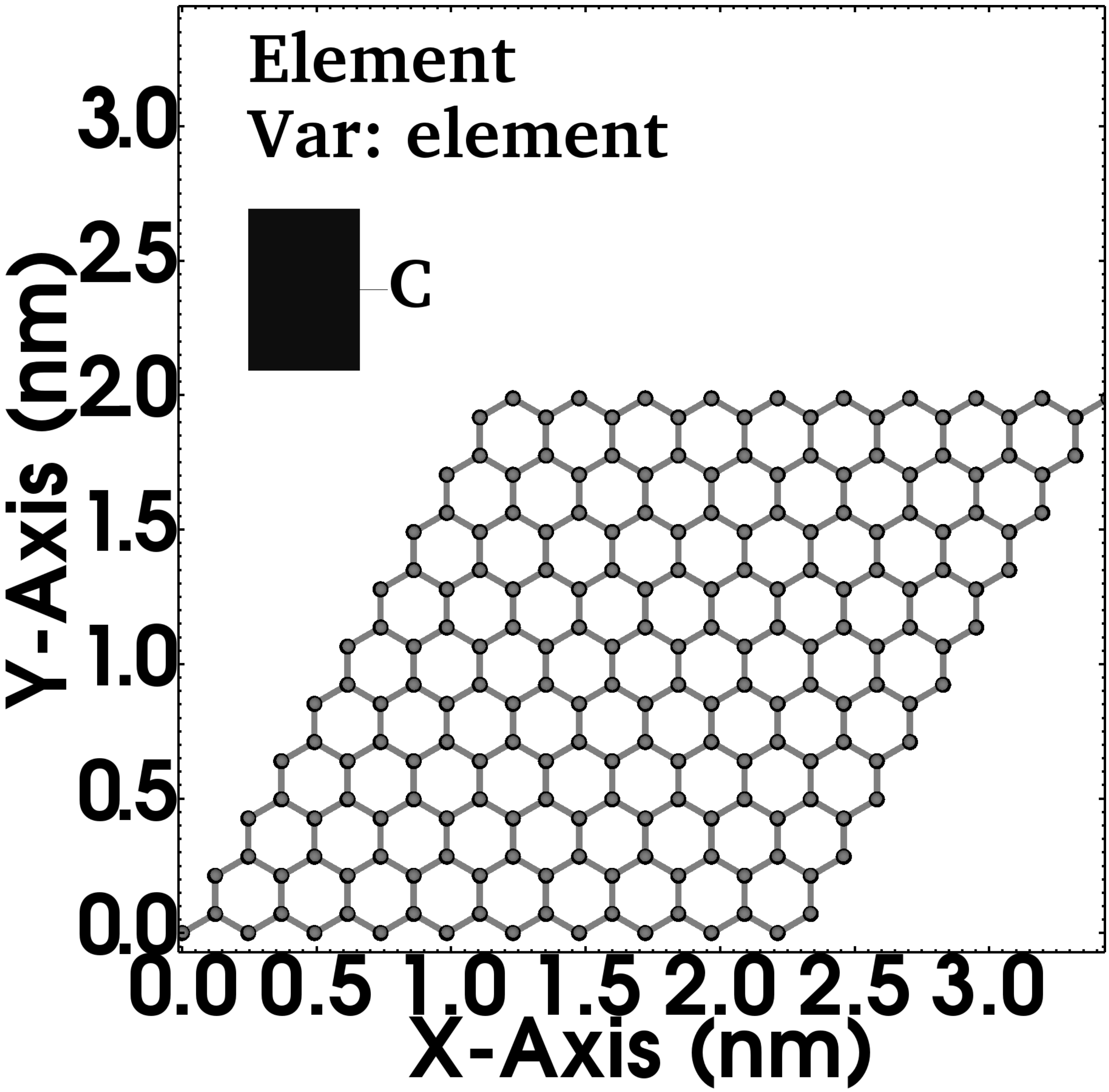

Figure 1. structure-flat

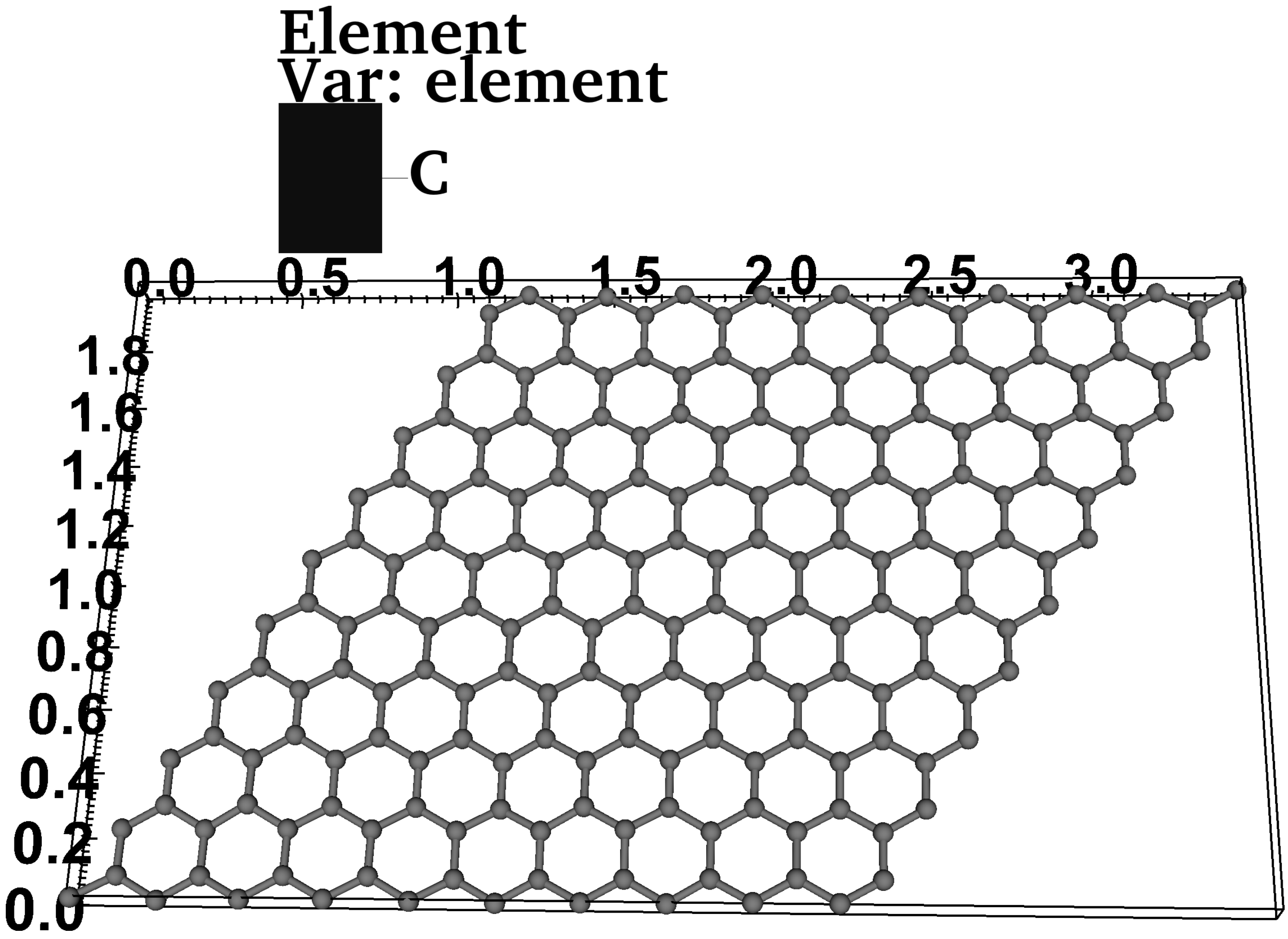

Figure 2. corrugate-5pm

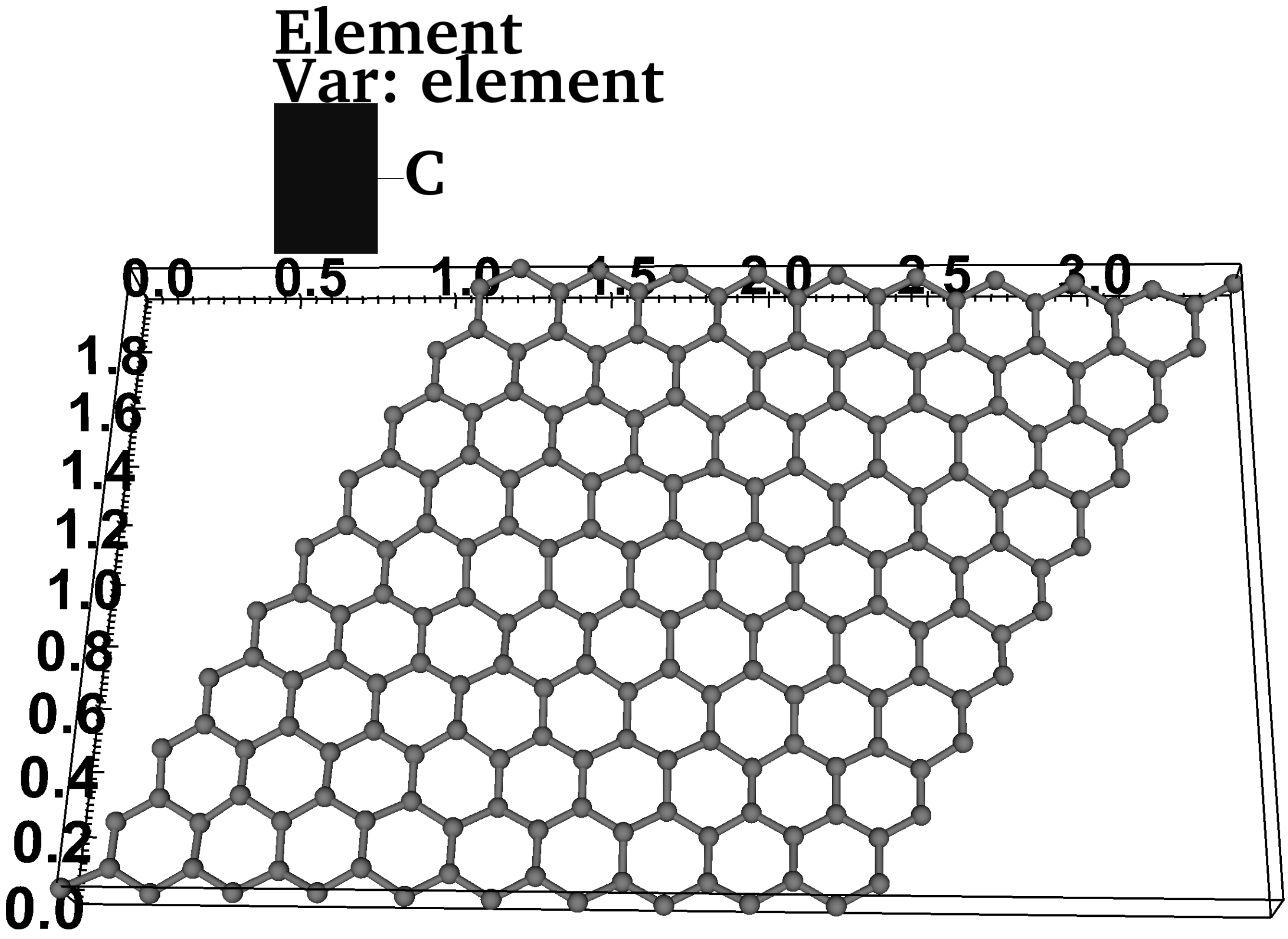

Figure 3. corrugate-10pm

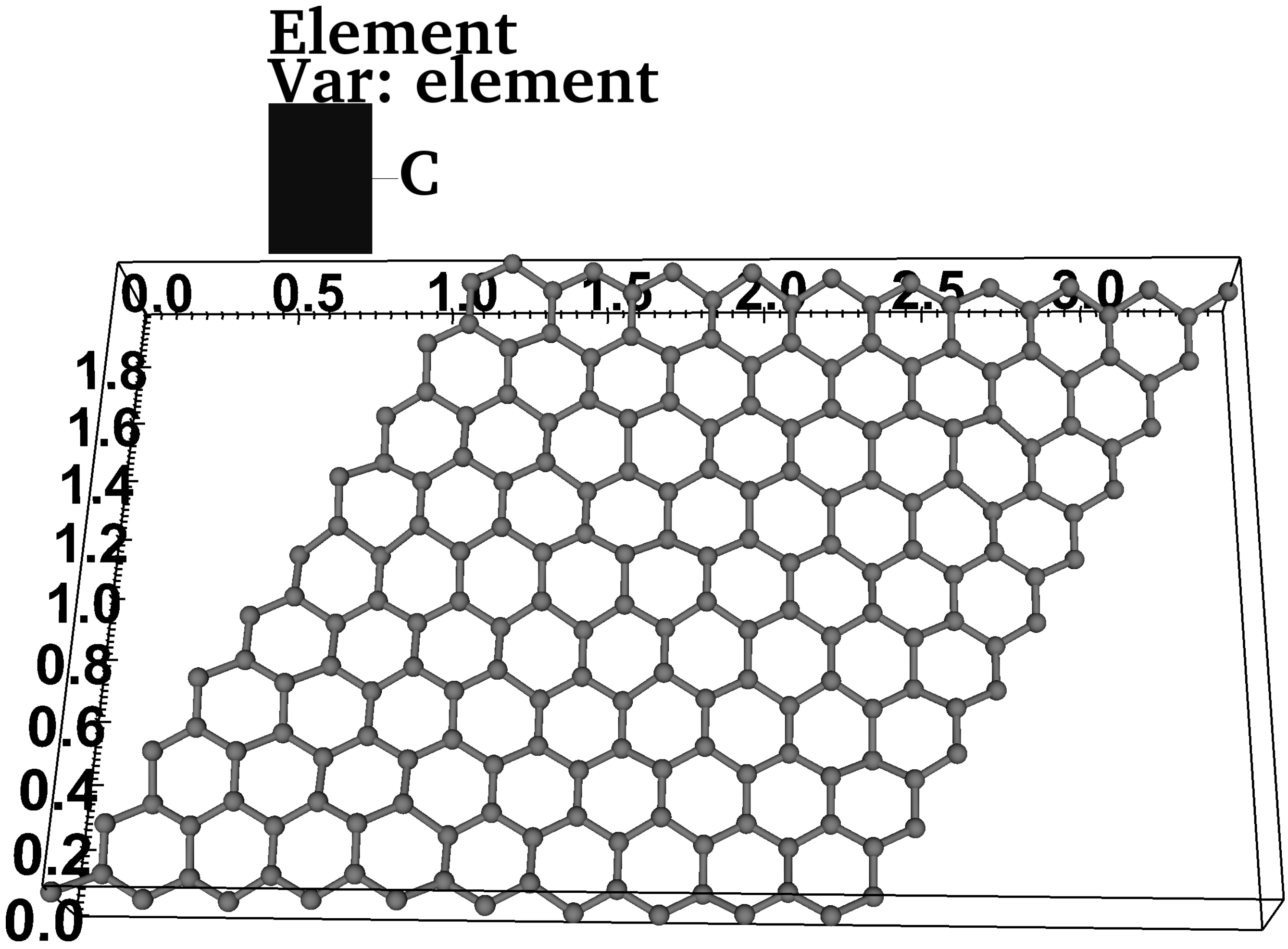

Figure 4. corrugate-15pm

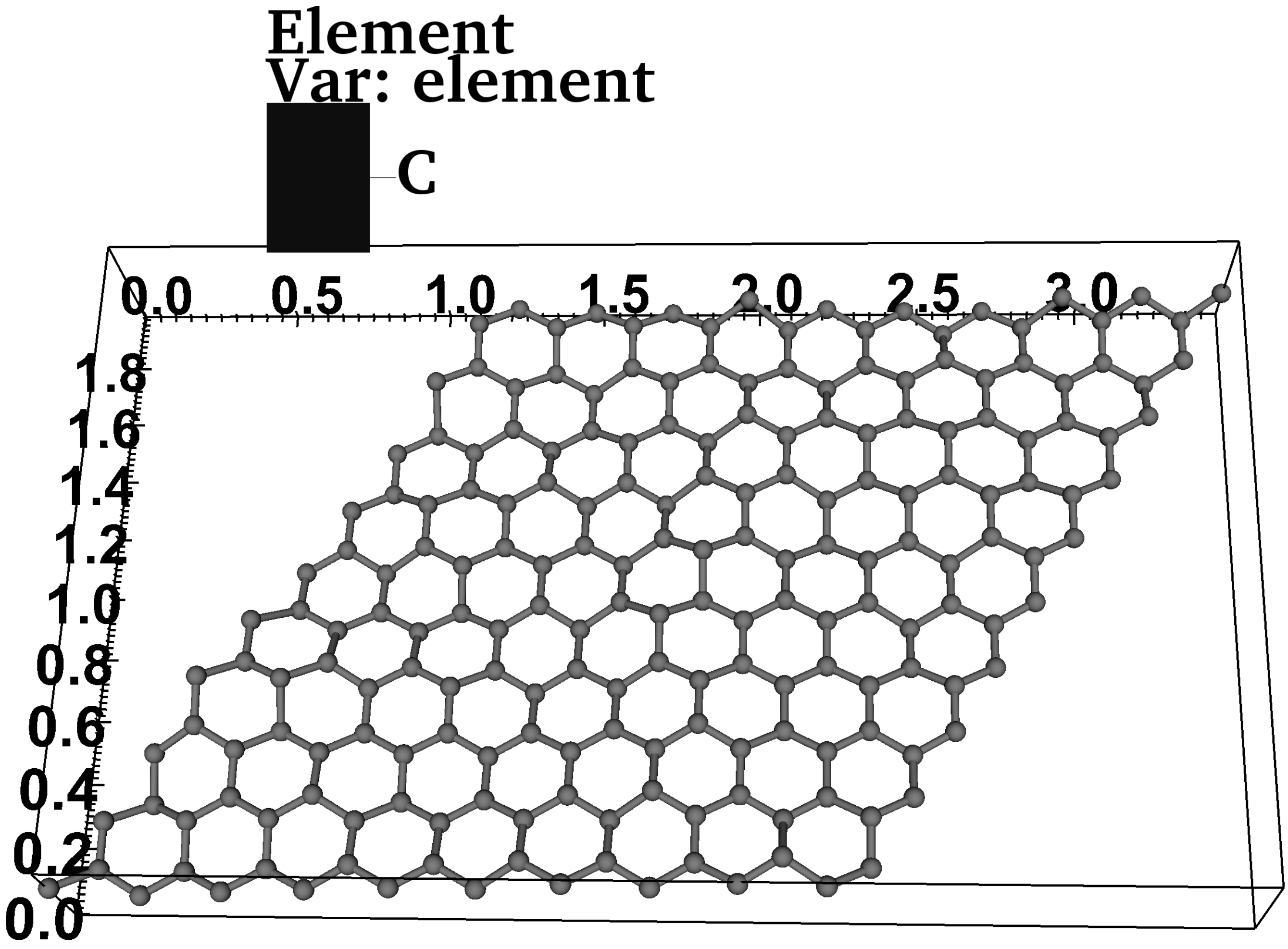

Figure 5. corrugate-20pm

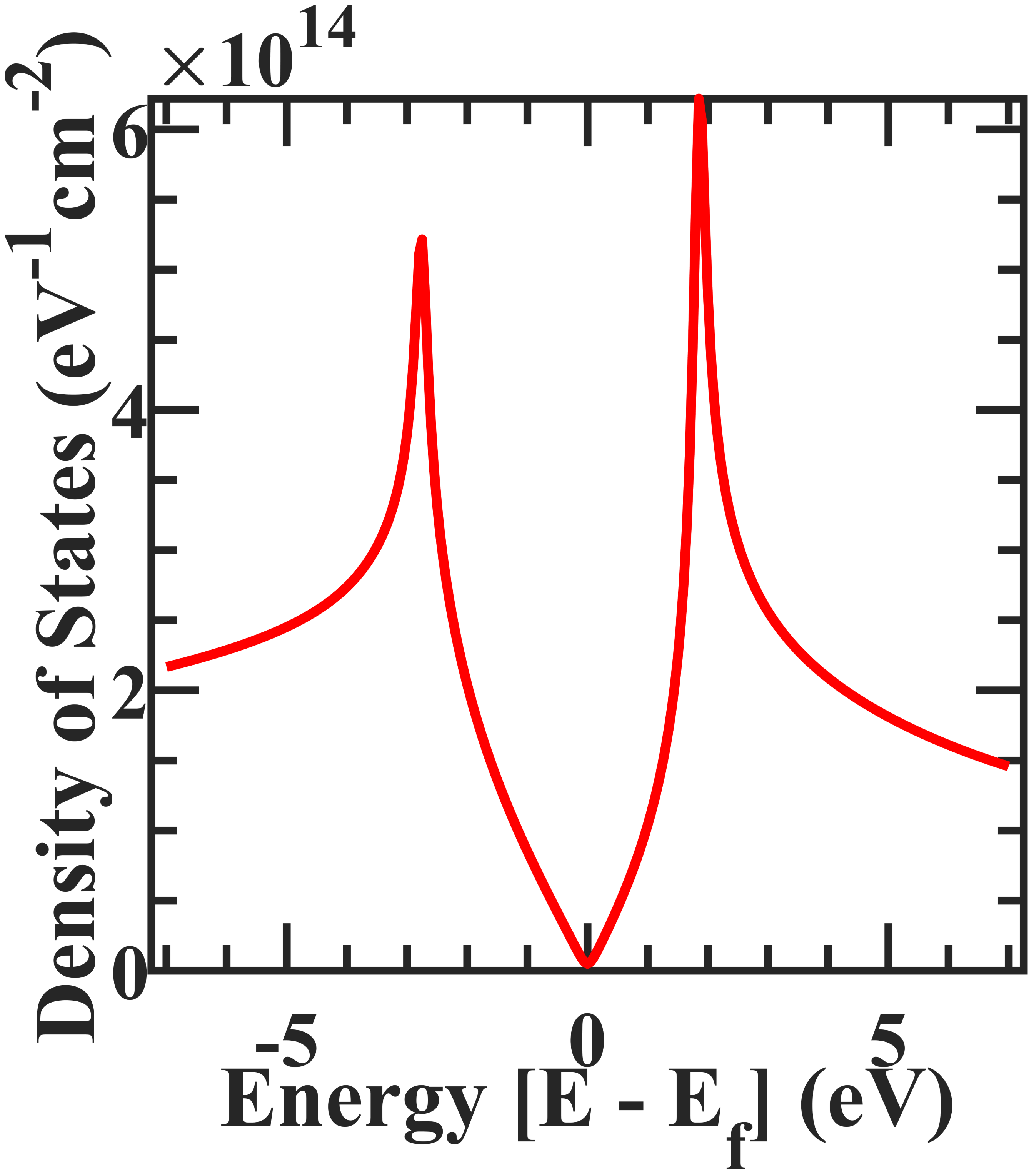

Figure 6. Density of state-flat

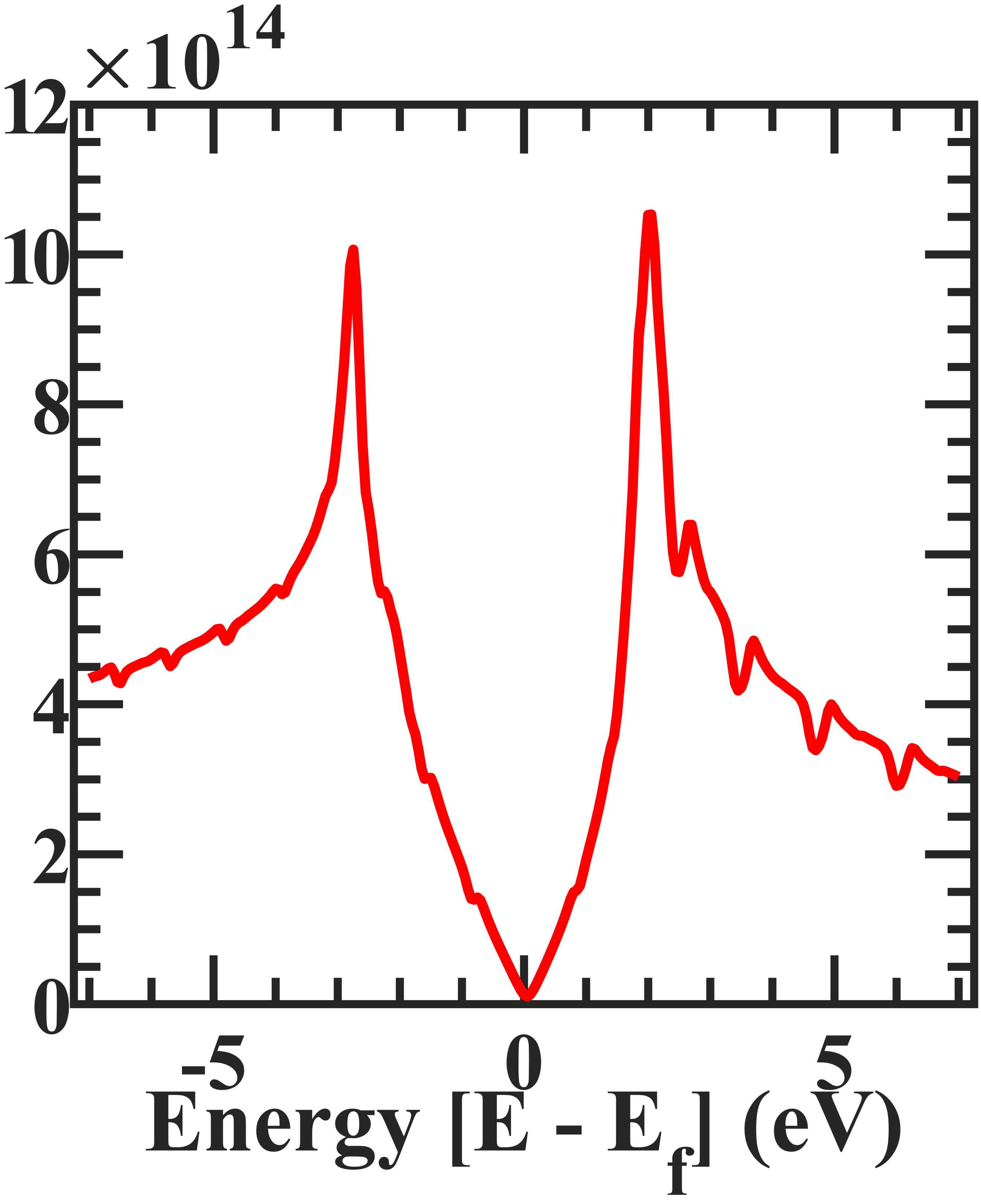

Figure 7. Density of state corrugate-5pm

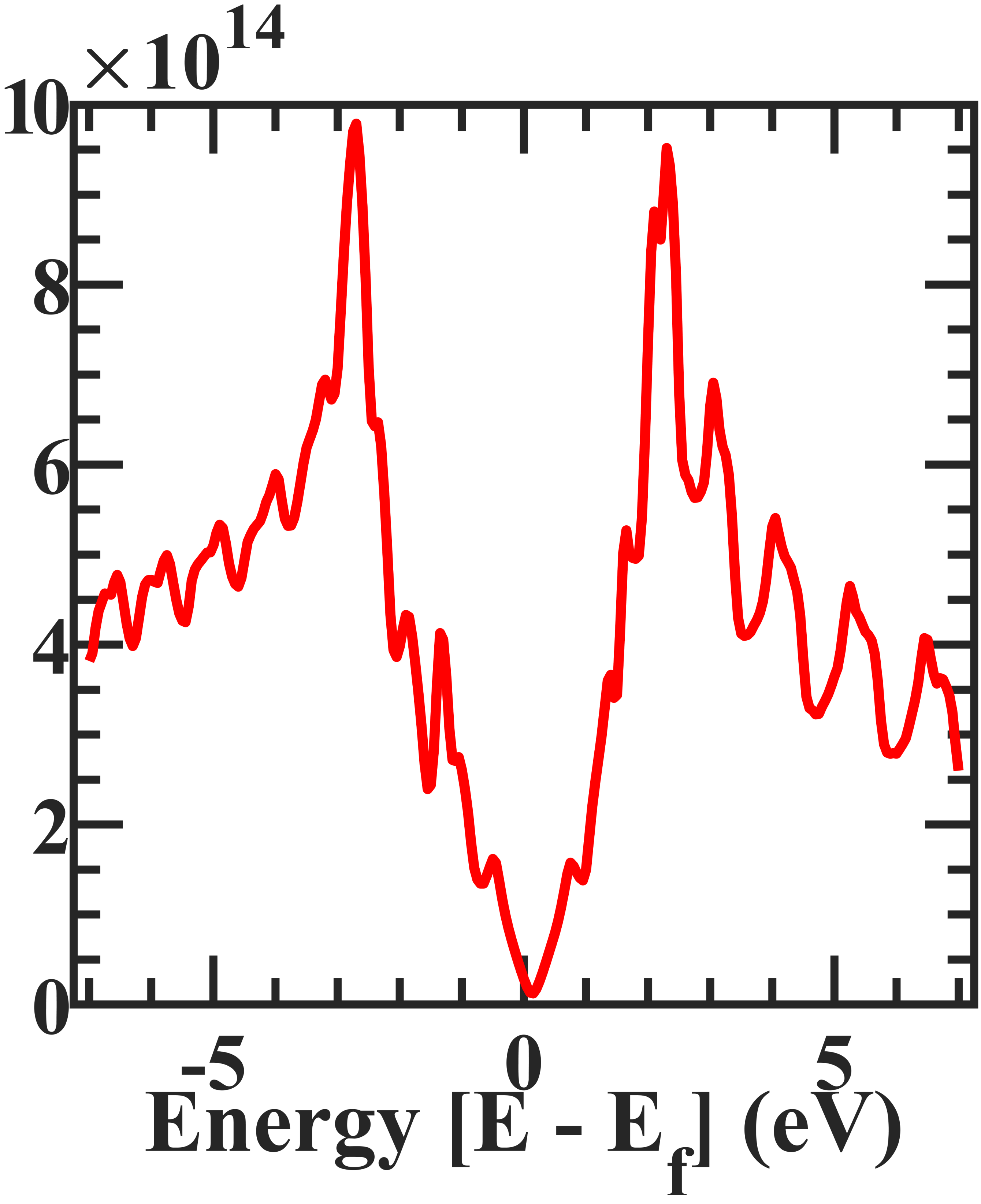

Figure 8. Density of state corrugate-10pm

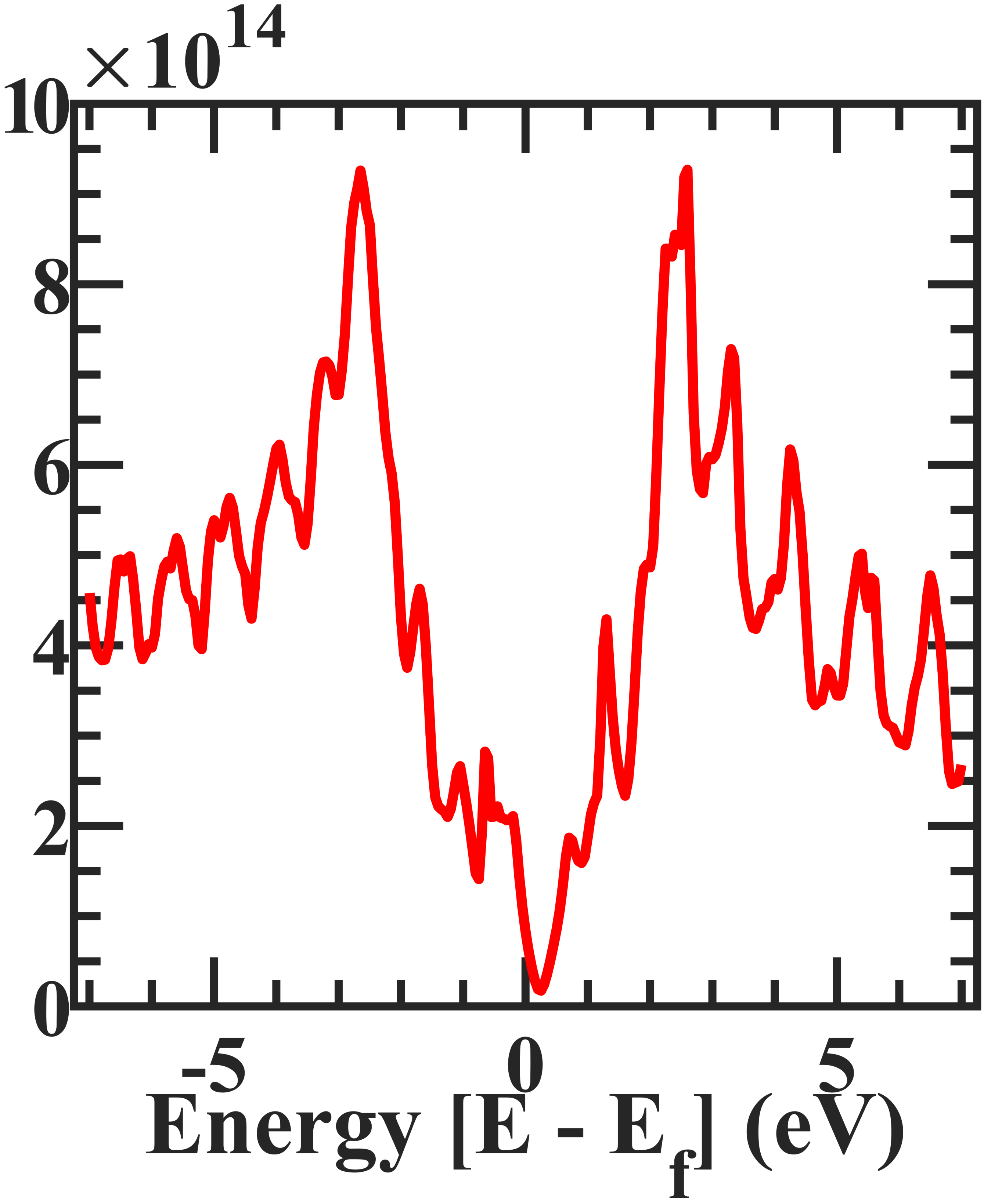

Figure 9. Density of state corrugate-15pm

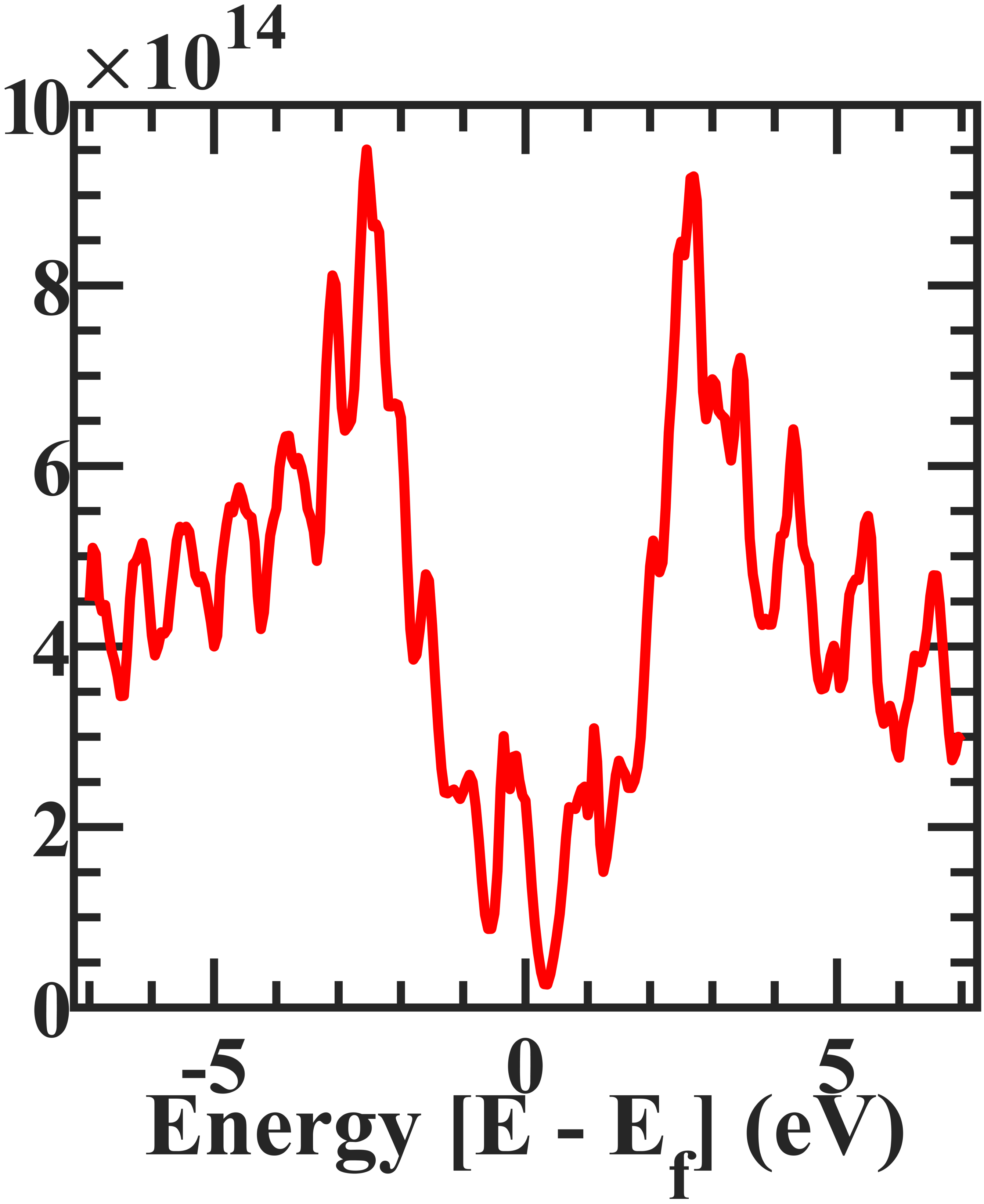

Figure 10. Density of state corrugate-20pm

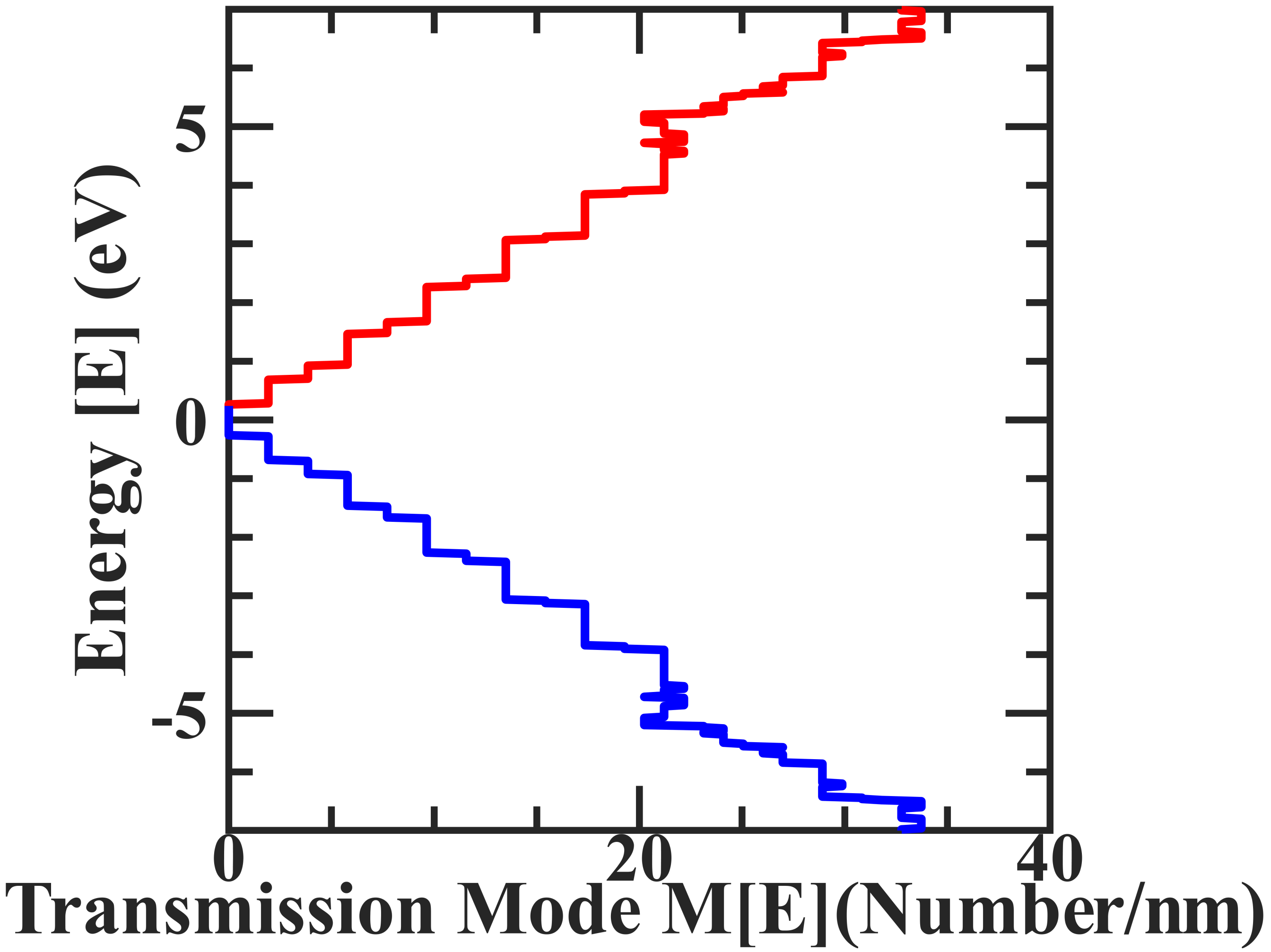

Figure 11. Density of Mode M(E) flat

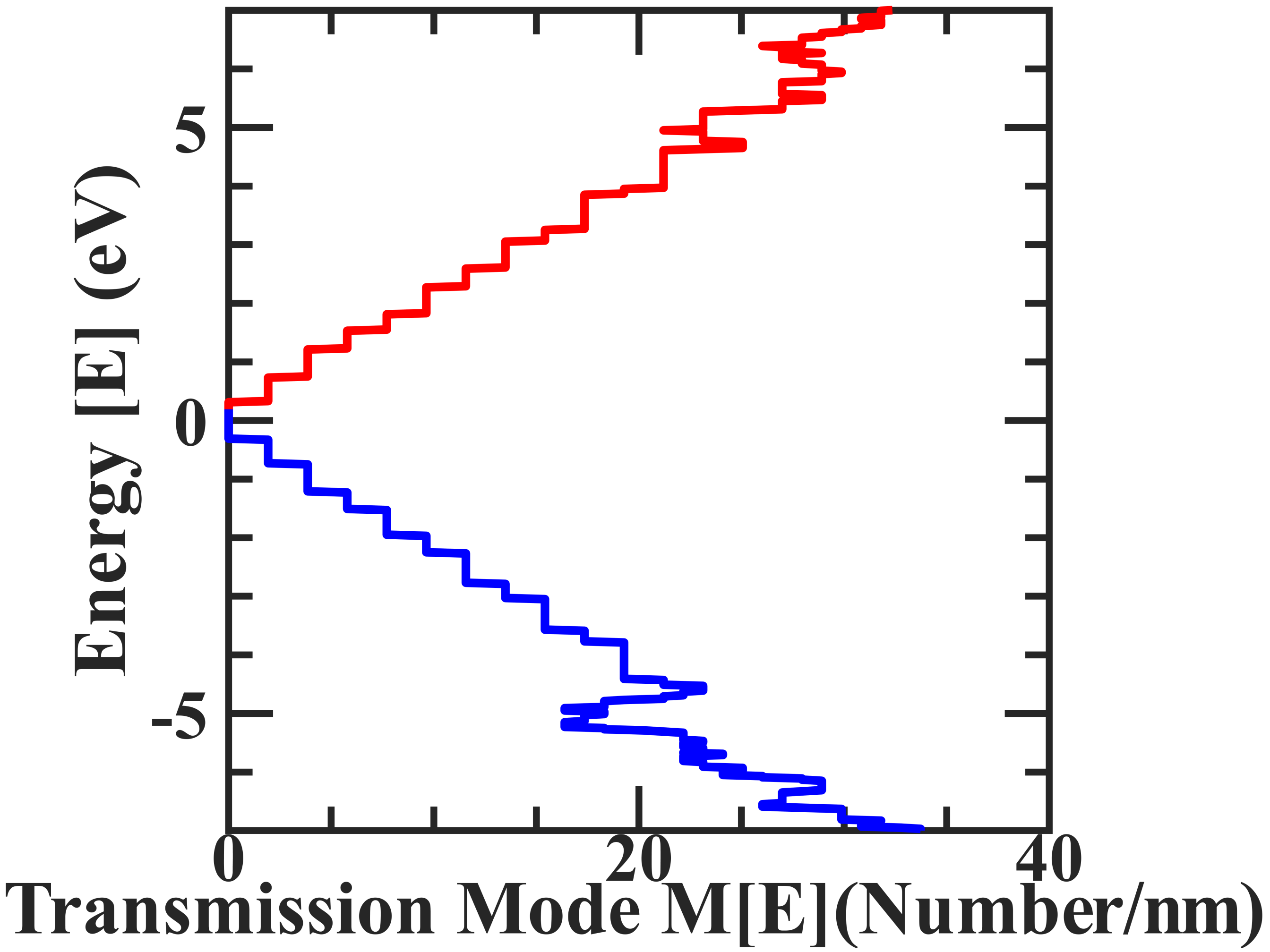

Figure 12. Density of Mode M(E) corrugate-5pm

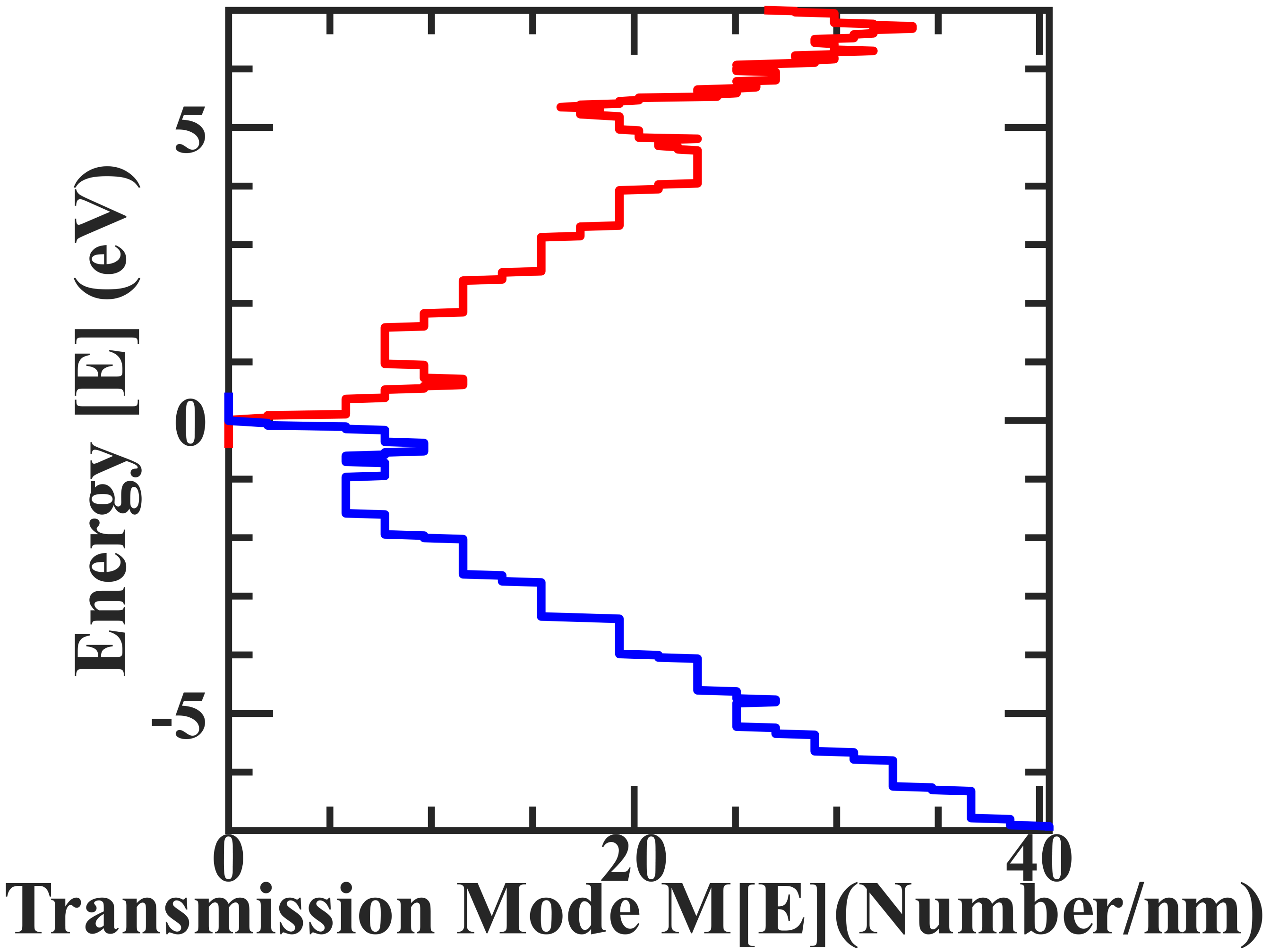

Figure 13. Density of Mode M(E) corrugate-10pm

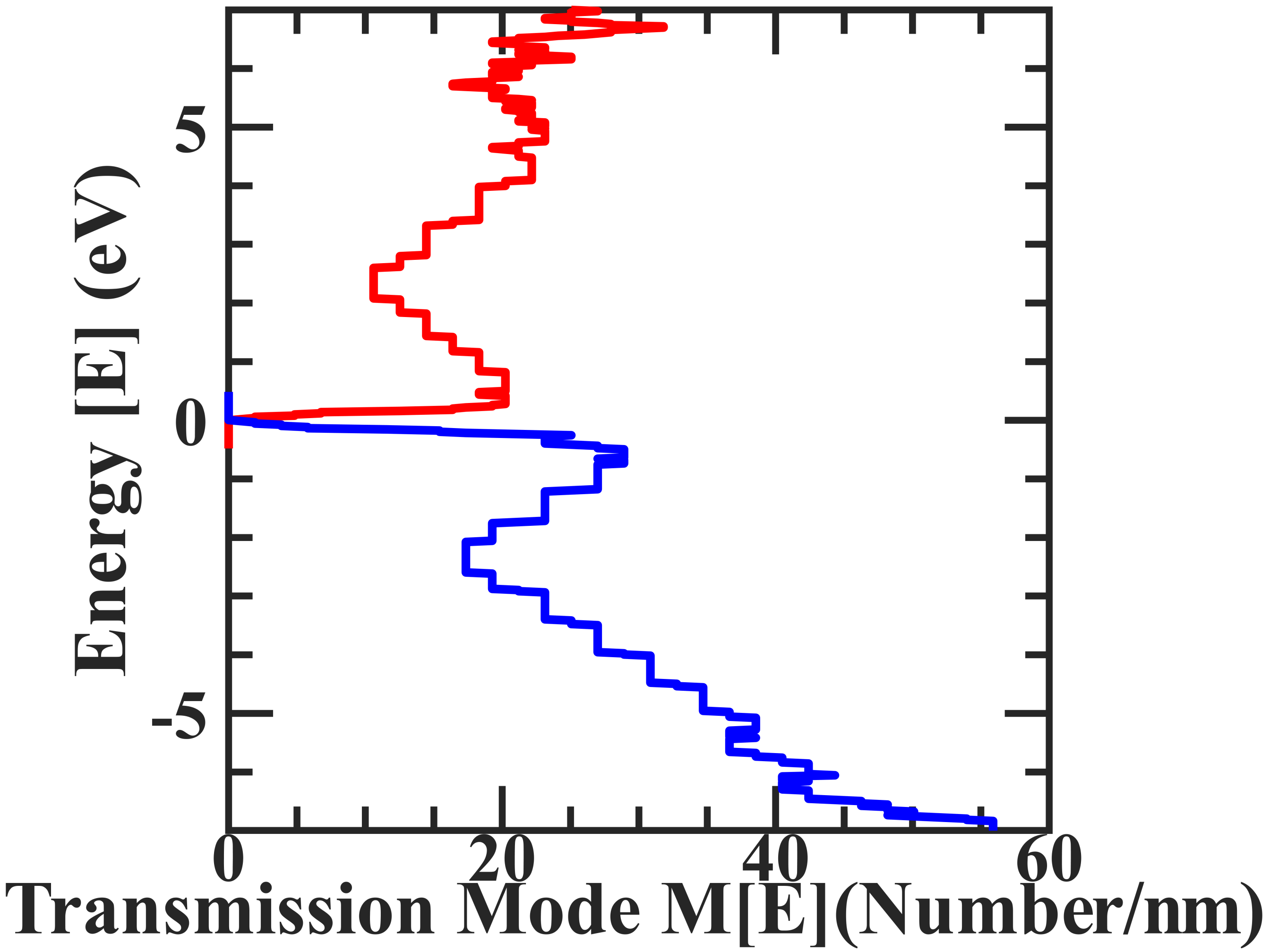

Figure 14. Density of Mode M(E) corrugate-15pm

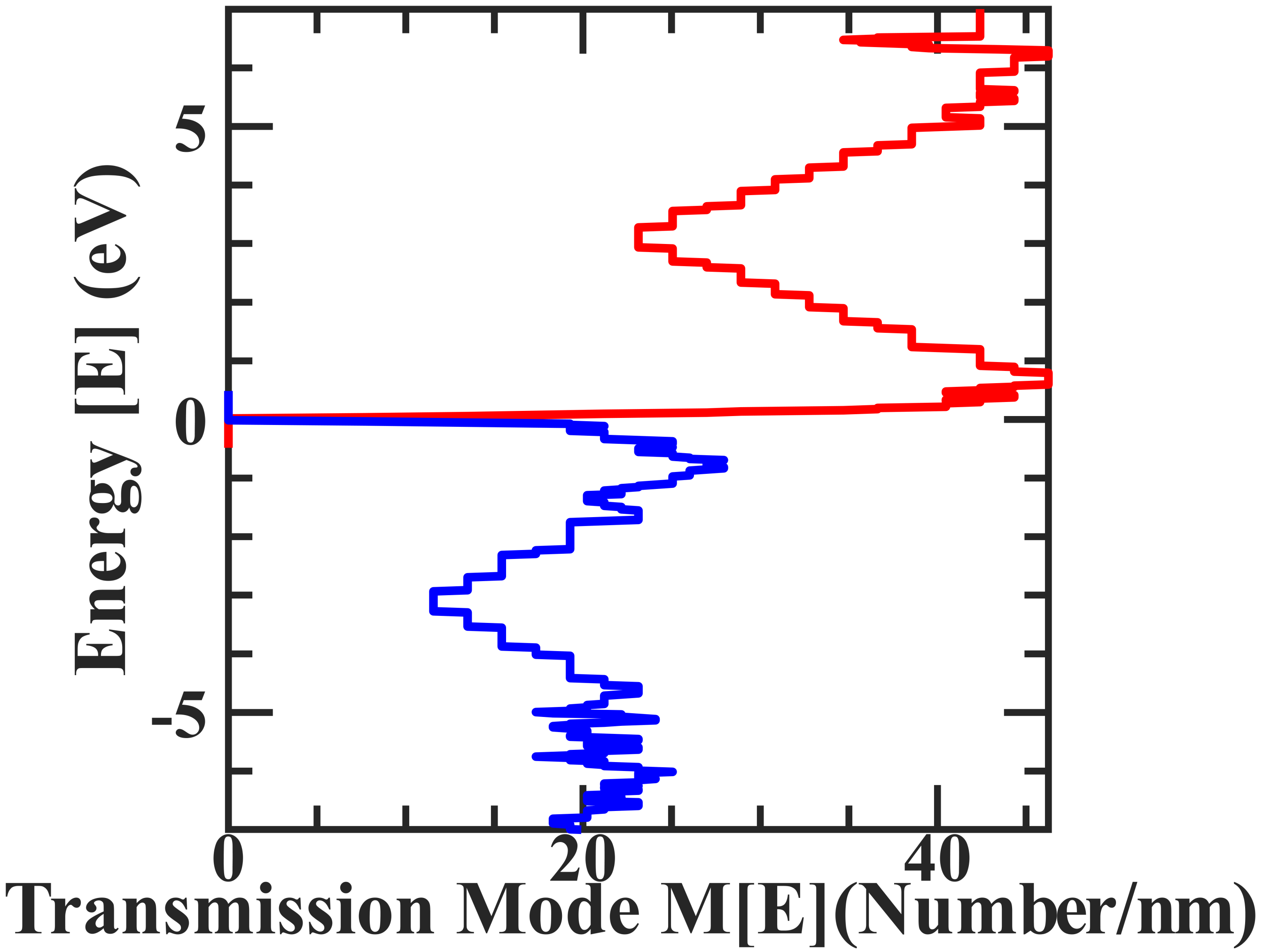

Figure 15. Density of Mode M(E) corrugate-20pm

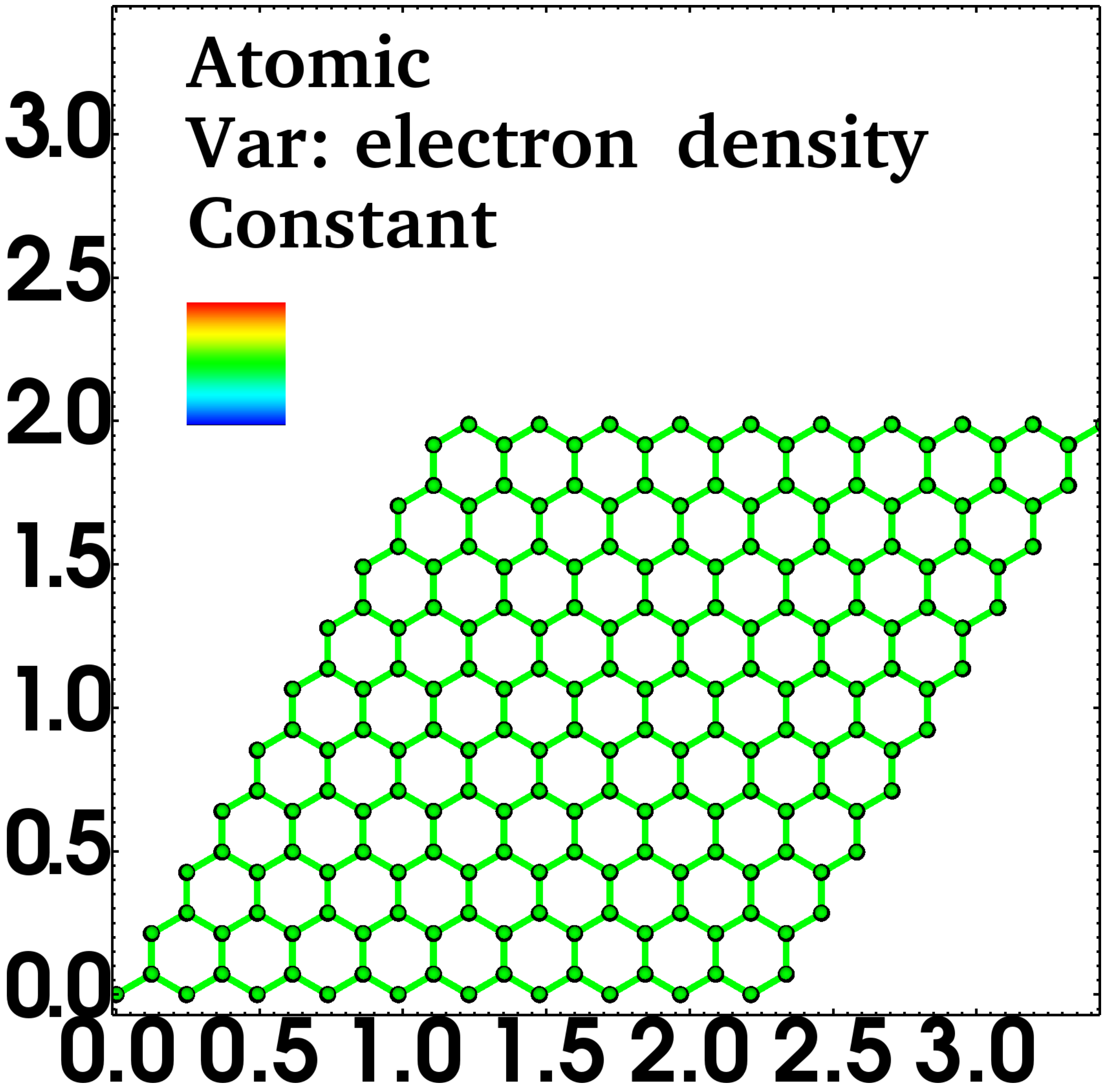

Figure 16. Electron density flat

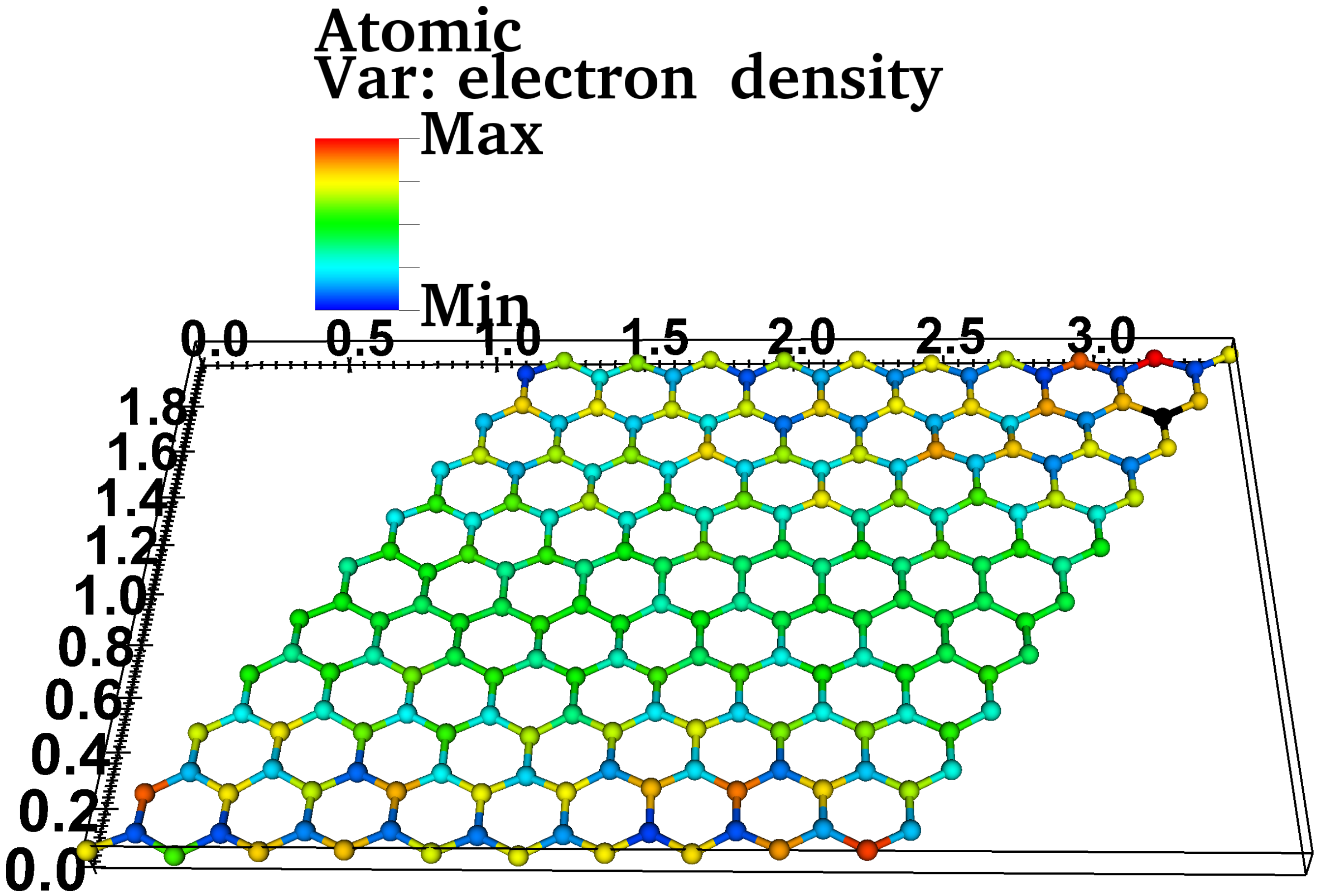

Figure 17. Electron density corrugate-5pm

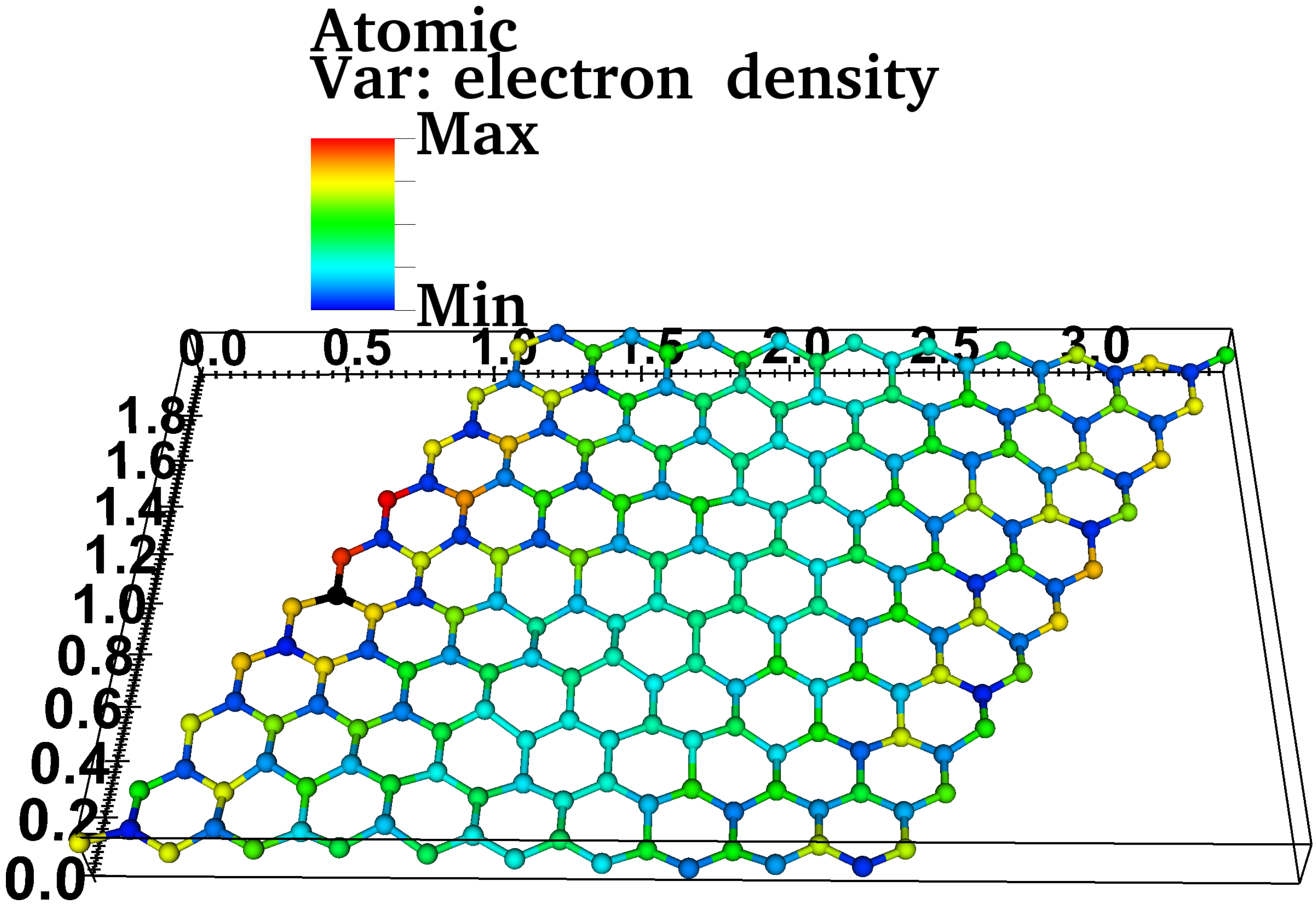

Figure 18. Electron density corrugate-10pm

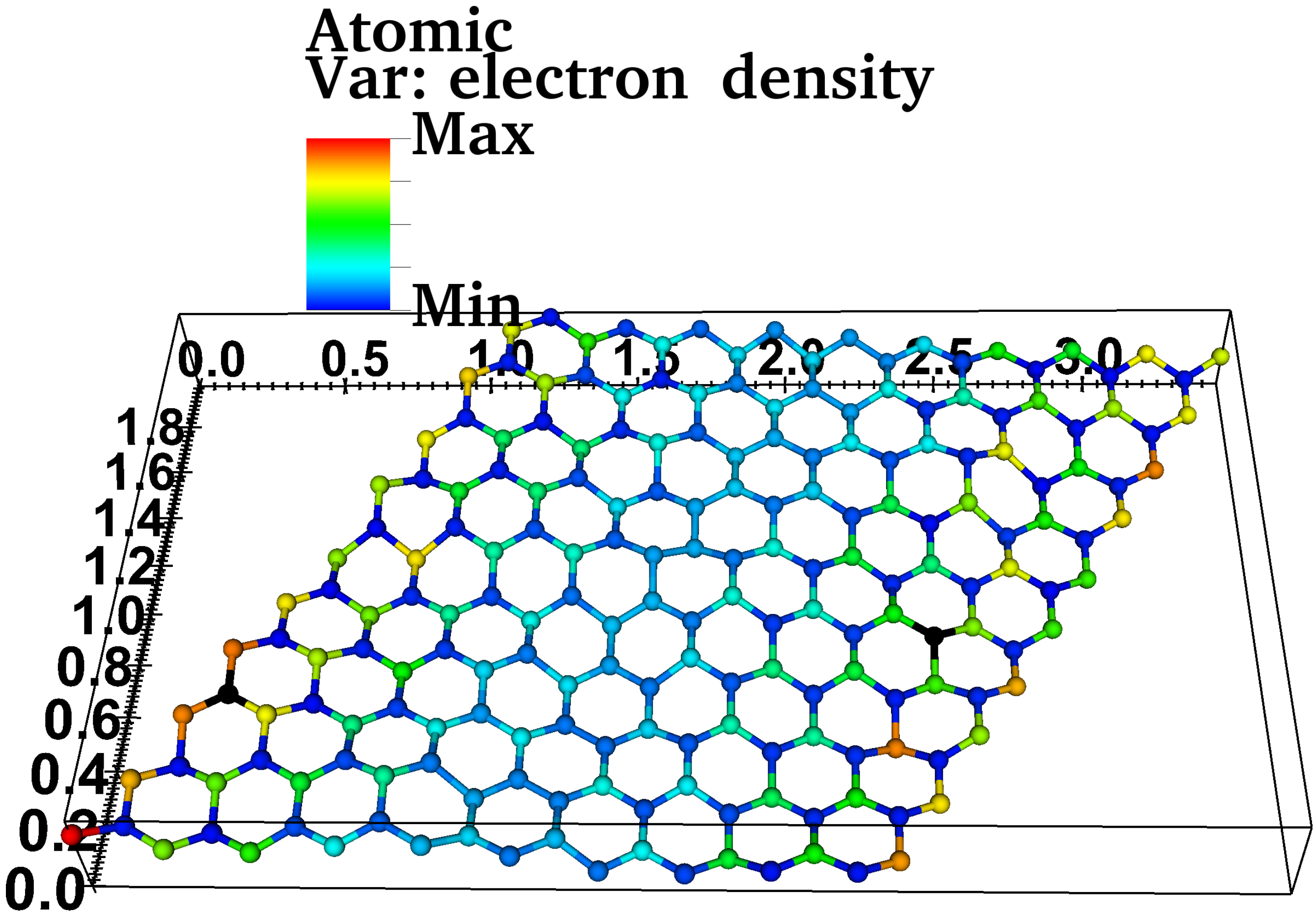

Figure 19. Electron density corrugate-15pm

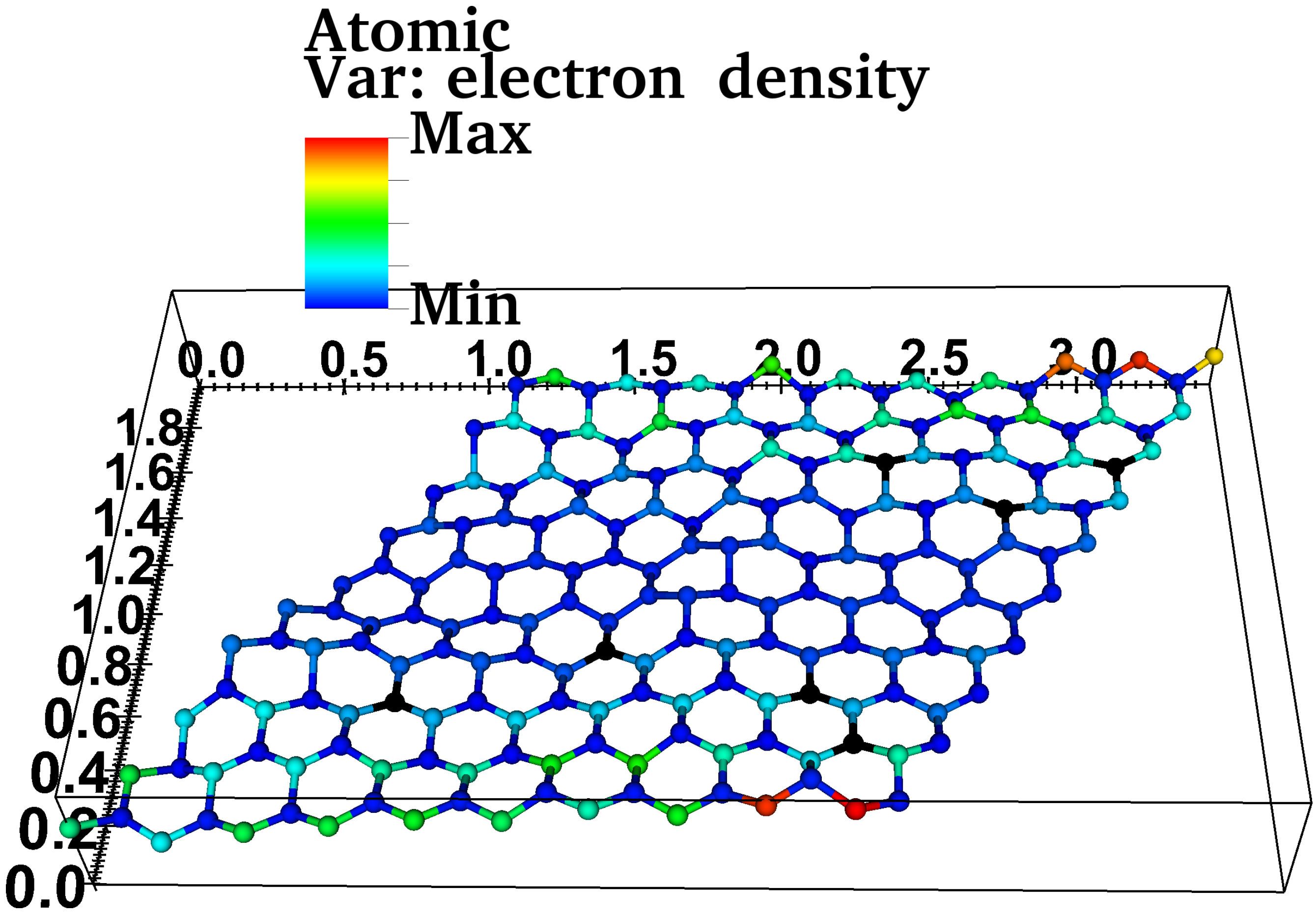

Figure 20. Electron density corrugate-20pm

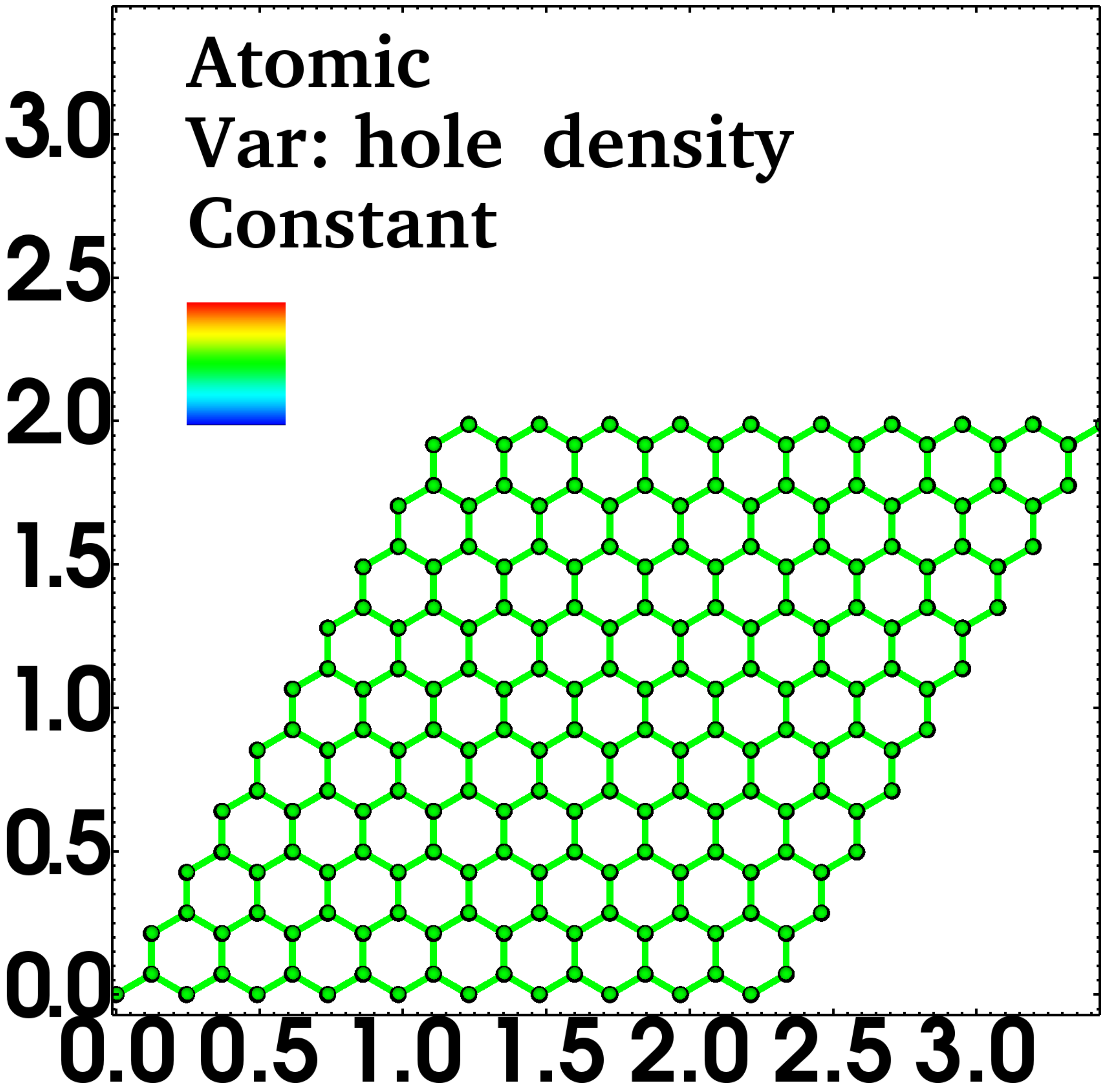

Figure 21. Hole density flat

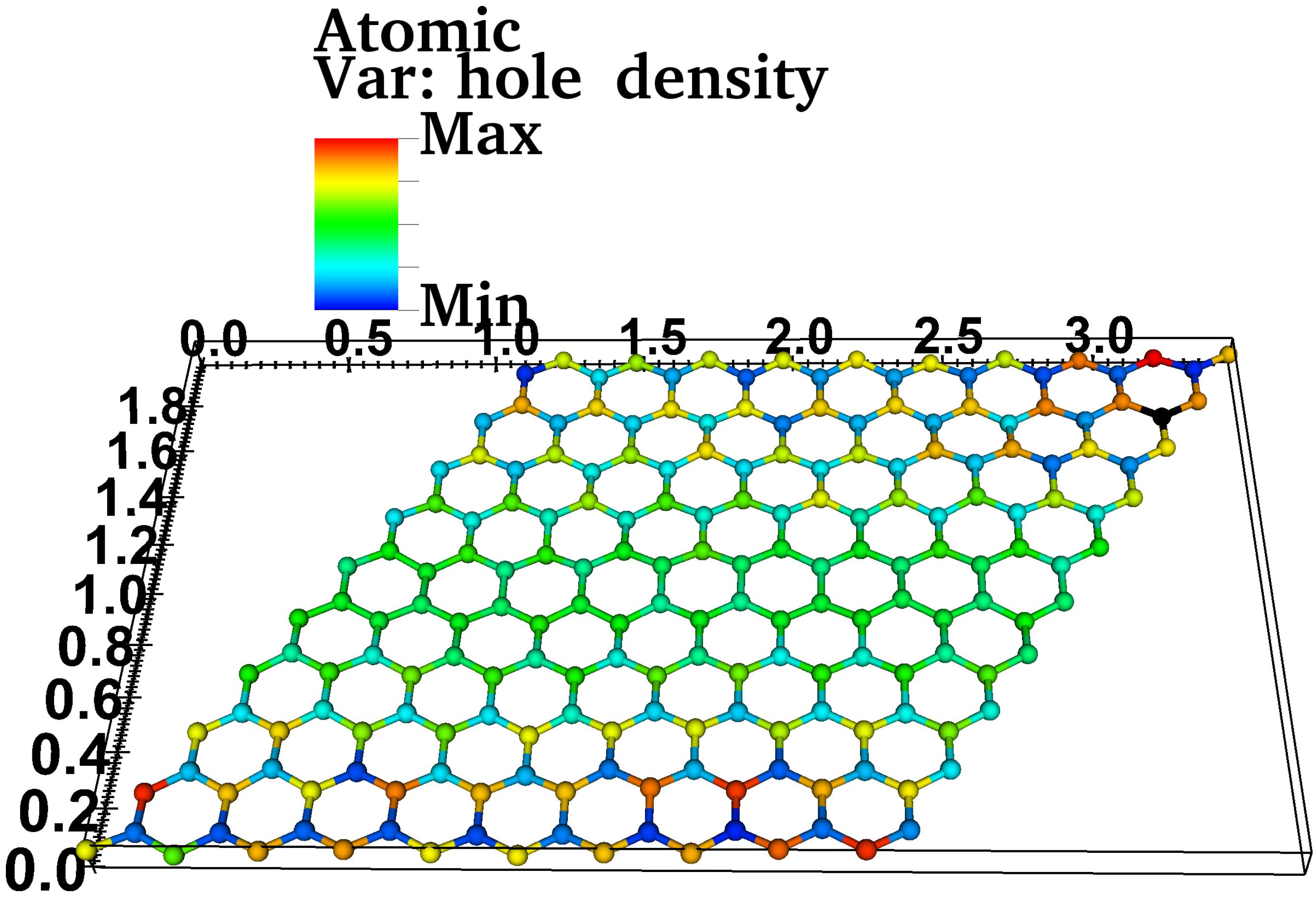

Figure 22. Hole density corrugate-5pm

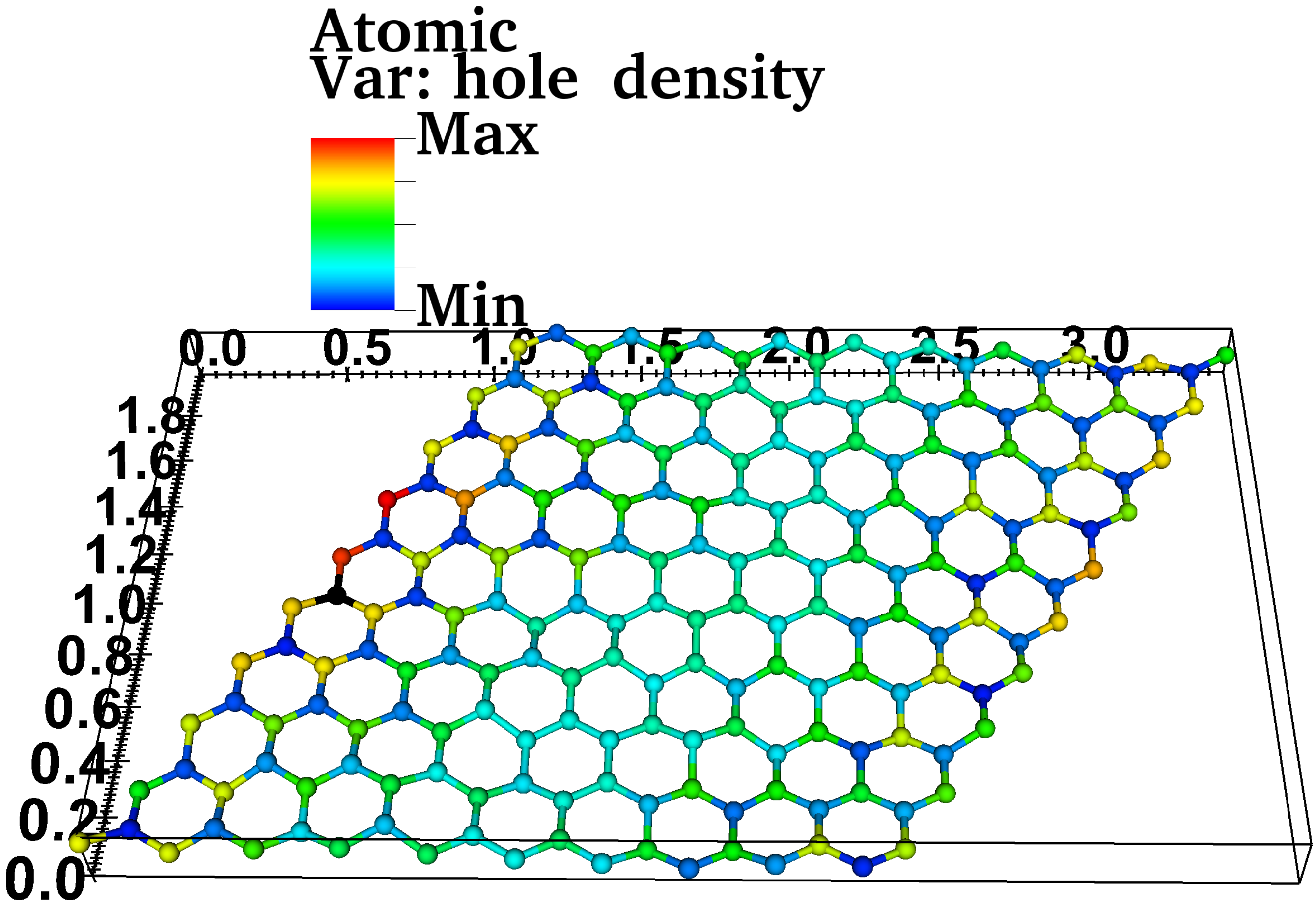

Figure 23. Hole density corrugate-10pm

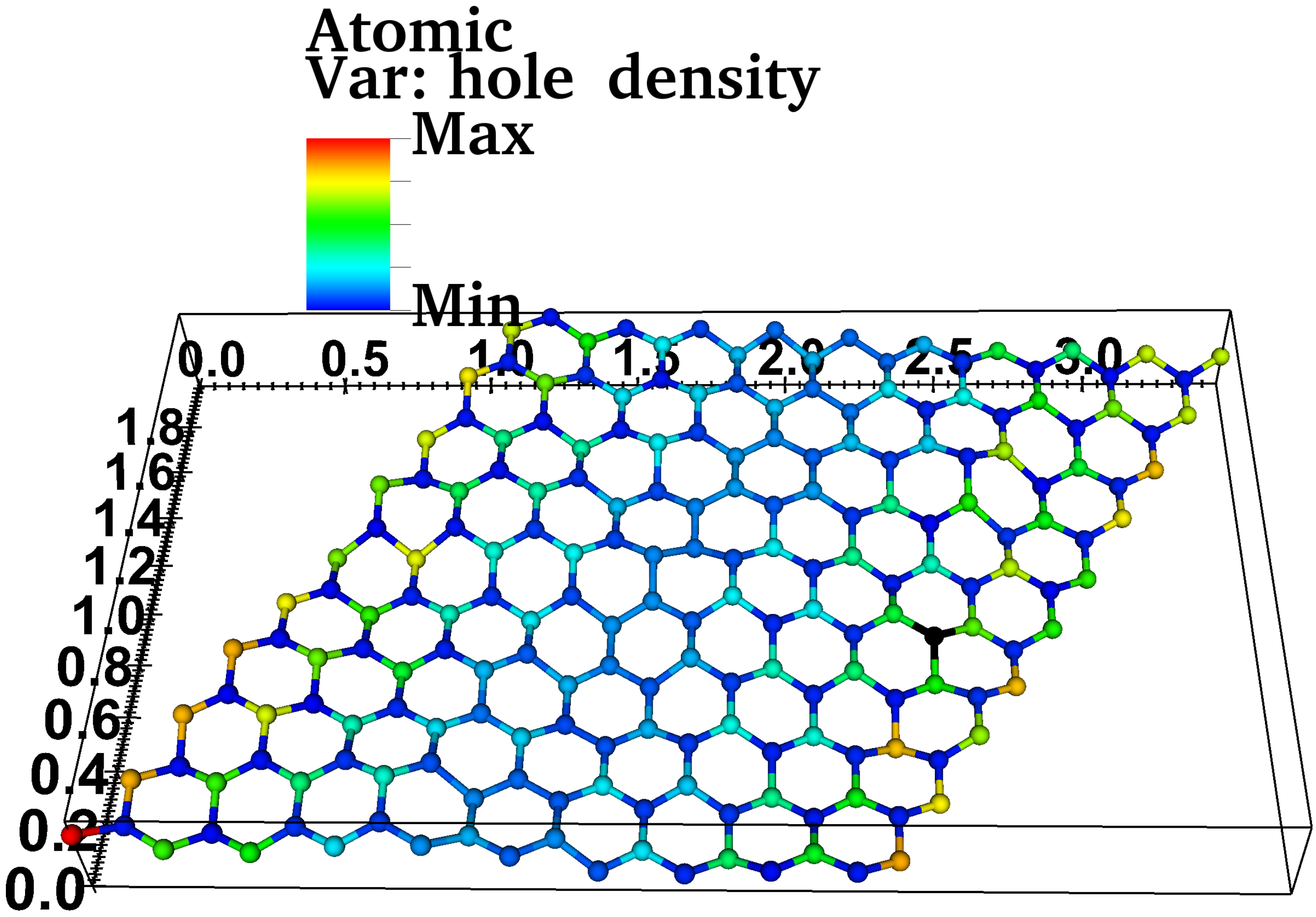

Figure 24. Hole density corrugate-15pm

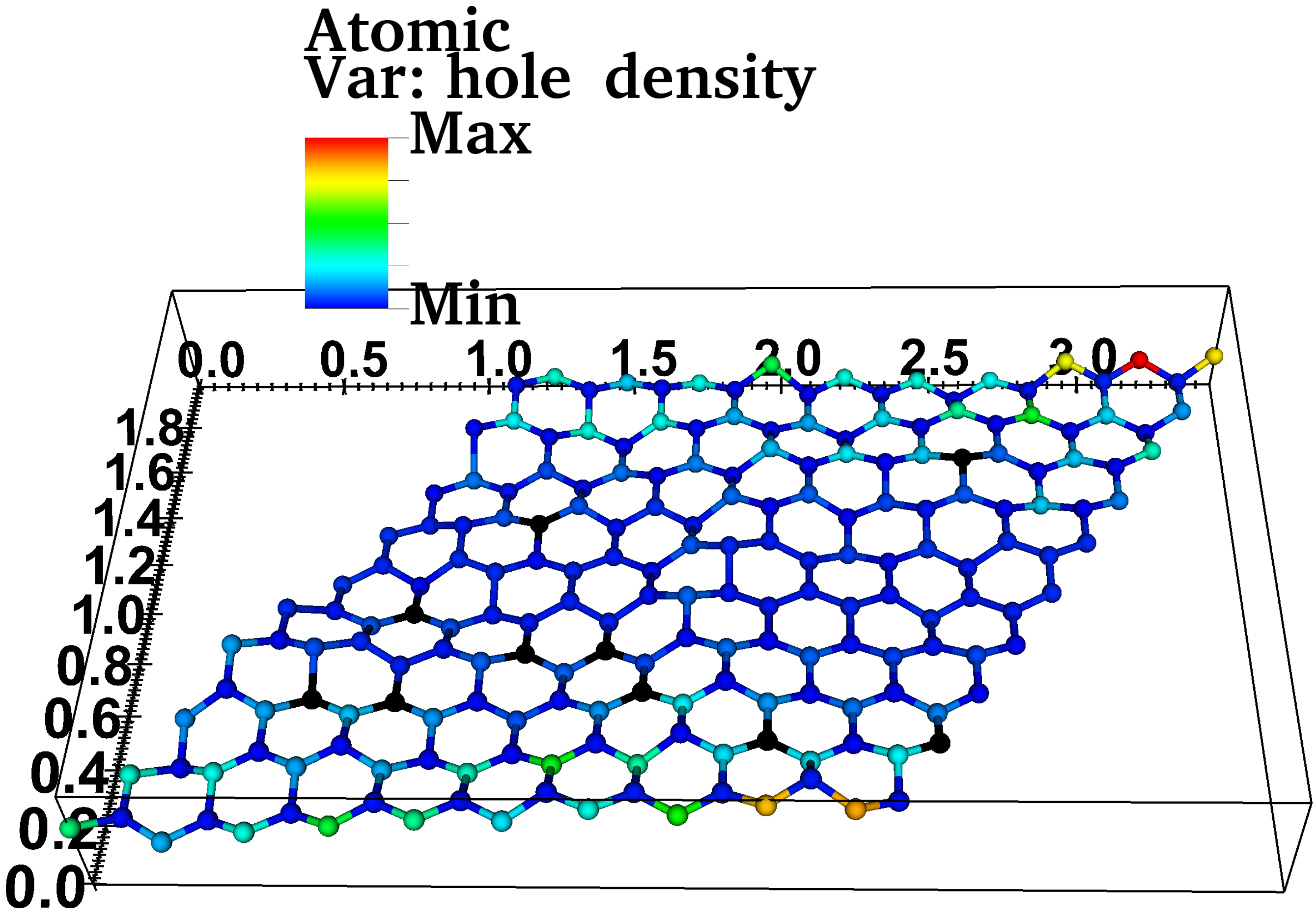

Figure 25. Hole density corrugate-20pm

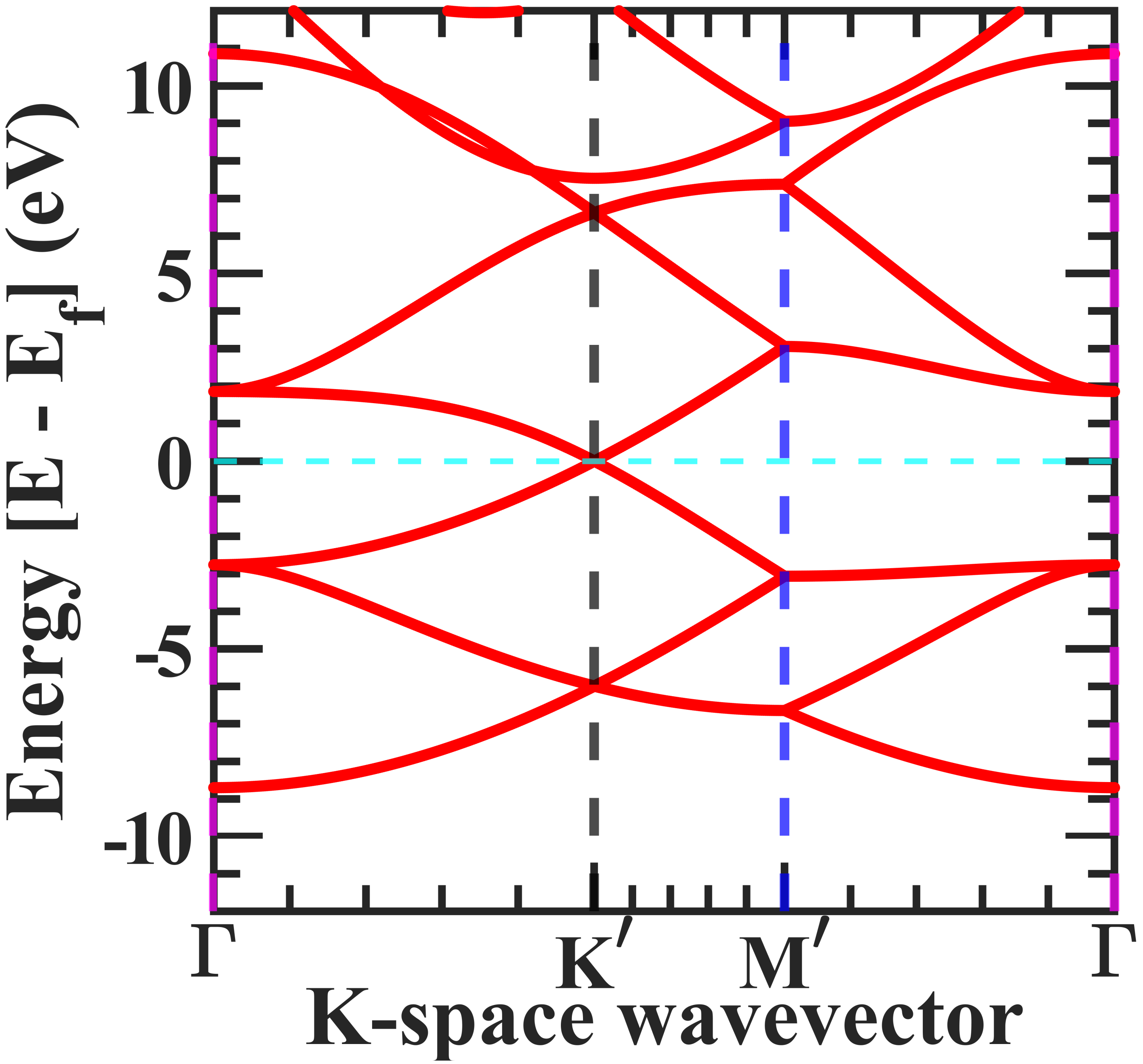

Figure 26. Band structure flat

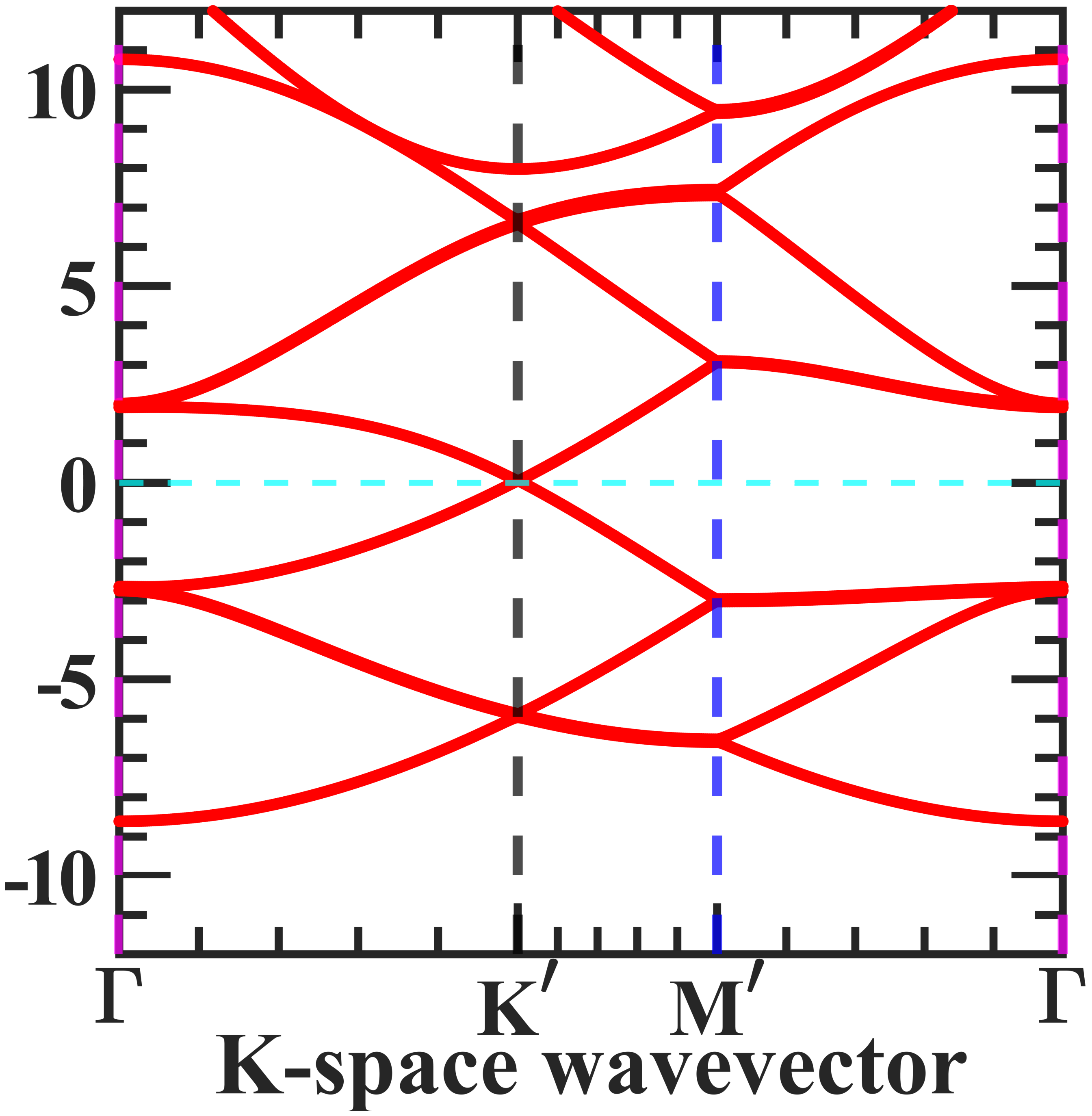

Figure 27. Band structure corrugate-5pm

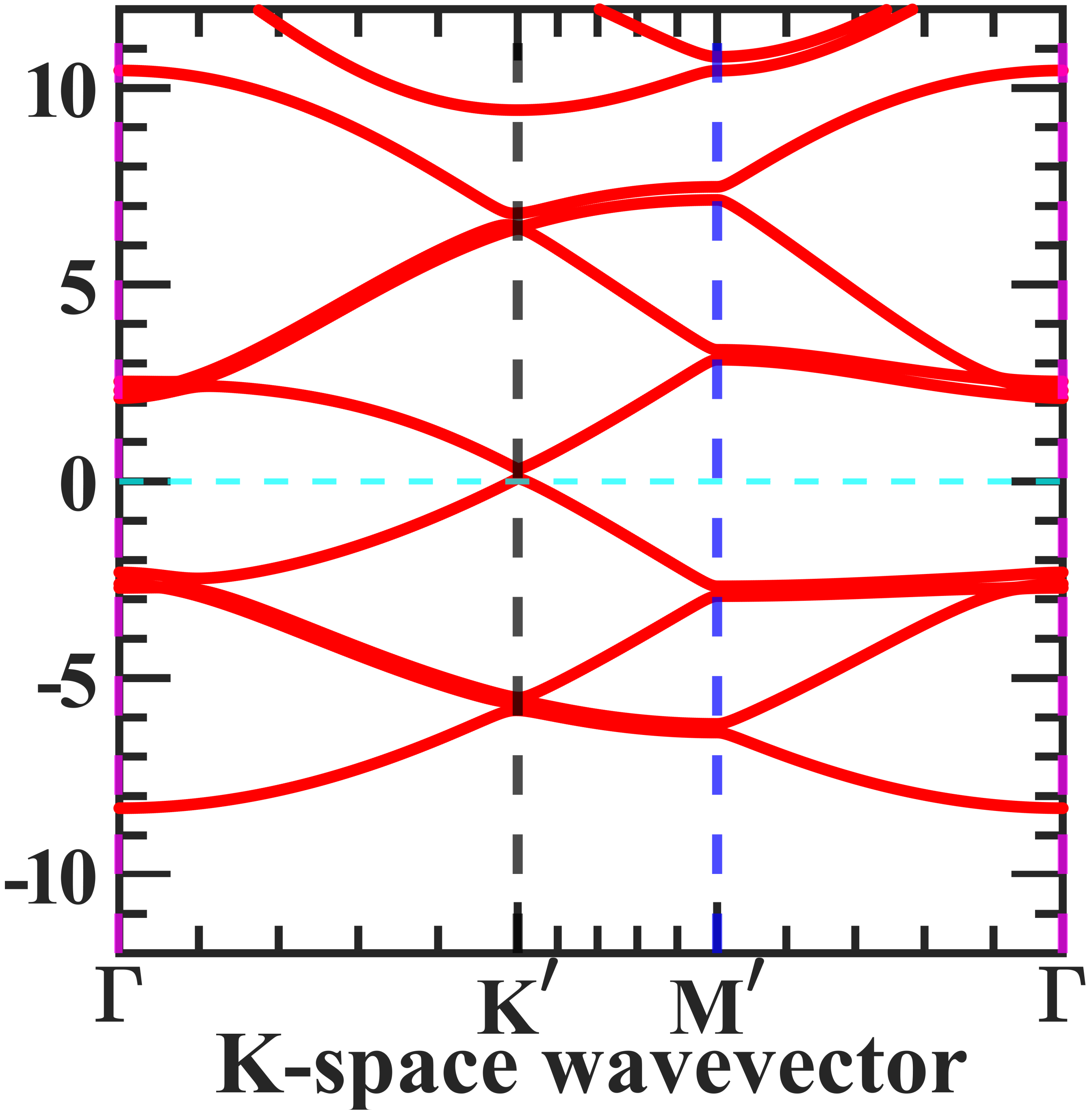

Figure 28. Band structure corrugate-10pm

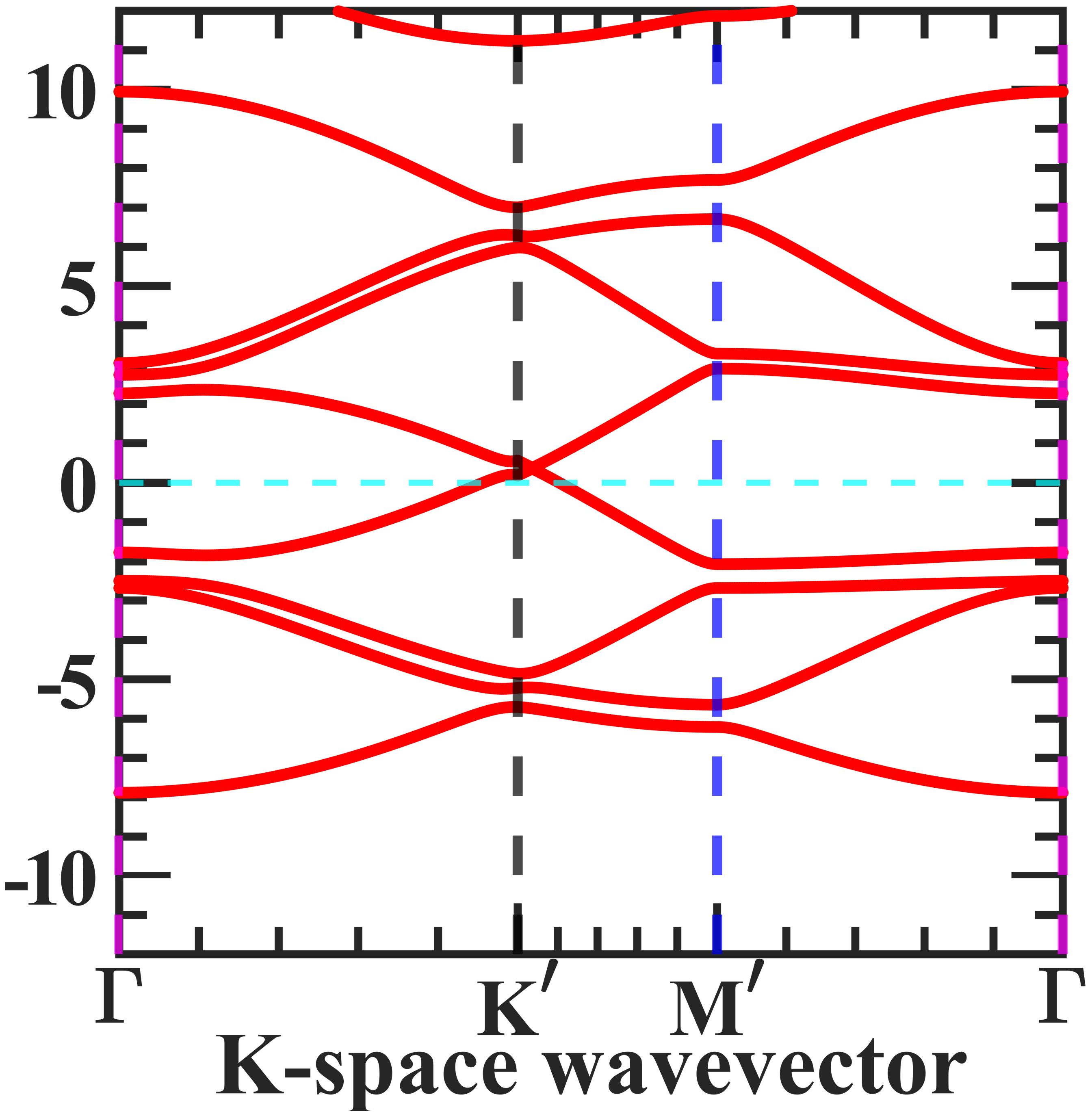

Figure 29. Band structure corrugate-15pm

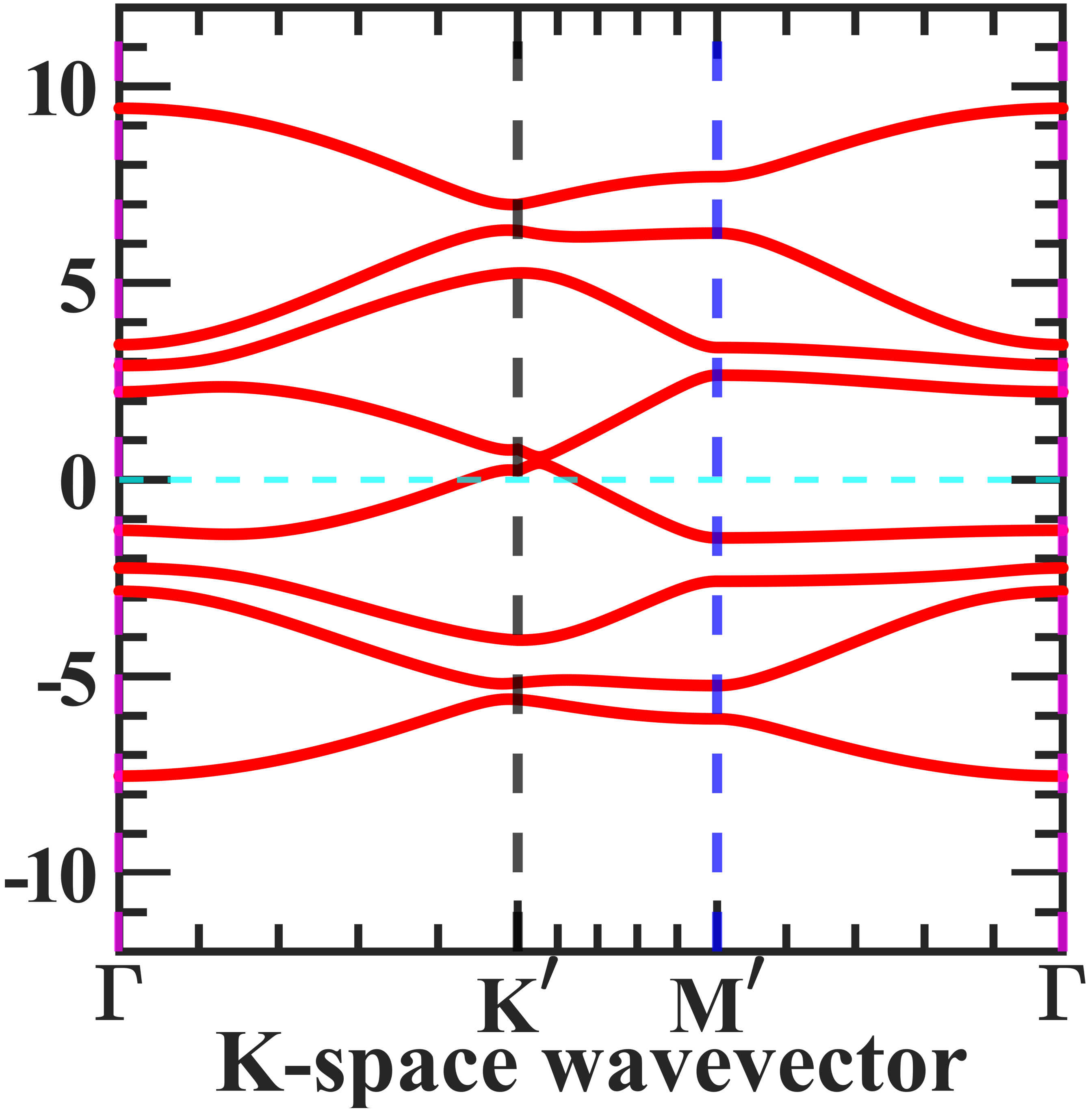

Figure 30. Band structure corrugate-20pm

100x100 graphene supercell

The fig. 31 correspond to 100x100 graphene supercell flat structure, and fig. 32 to fig. 35 correspond to the corrugate device structure with roughness varying from 5 pm to 20 pm, mimicking the roughness profile of h-BN and SiO_2 substrate. Similarly, fig. 36 to fig. 40 represents the corresponding density of state, **??** to fig. 44 represents the electronic density of mode M(E) in the x-y direction, **??** to fig. 48 represents the electronic density, **??** to fig. 51 represents the hole density and **??** to fig. 55 represents the electronic bandstructure of the corresponding device structure. In all, the corrugate structure correlation is kept constant at 10nm length. In the simulated device, the primitive unit cell has two atoms per cell, and a total of 20000 atoms are simulated by a finite element mesh of 80000 point Density of Mode size. The P-D tight-binding model contains three orbitals, namely carbon P_z, and carbon-hydrogen passivated D_{yz}, D_{xz} orbitals. Therefore total degree of free density of Mode in hamiltonian is 60000 variable-sized. The K' and M' are high symmetric point that corresponds to the folded reduced

BZ-zone of graphene supercell.

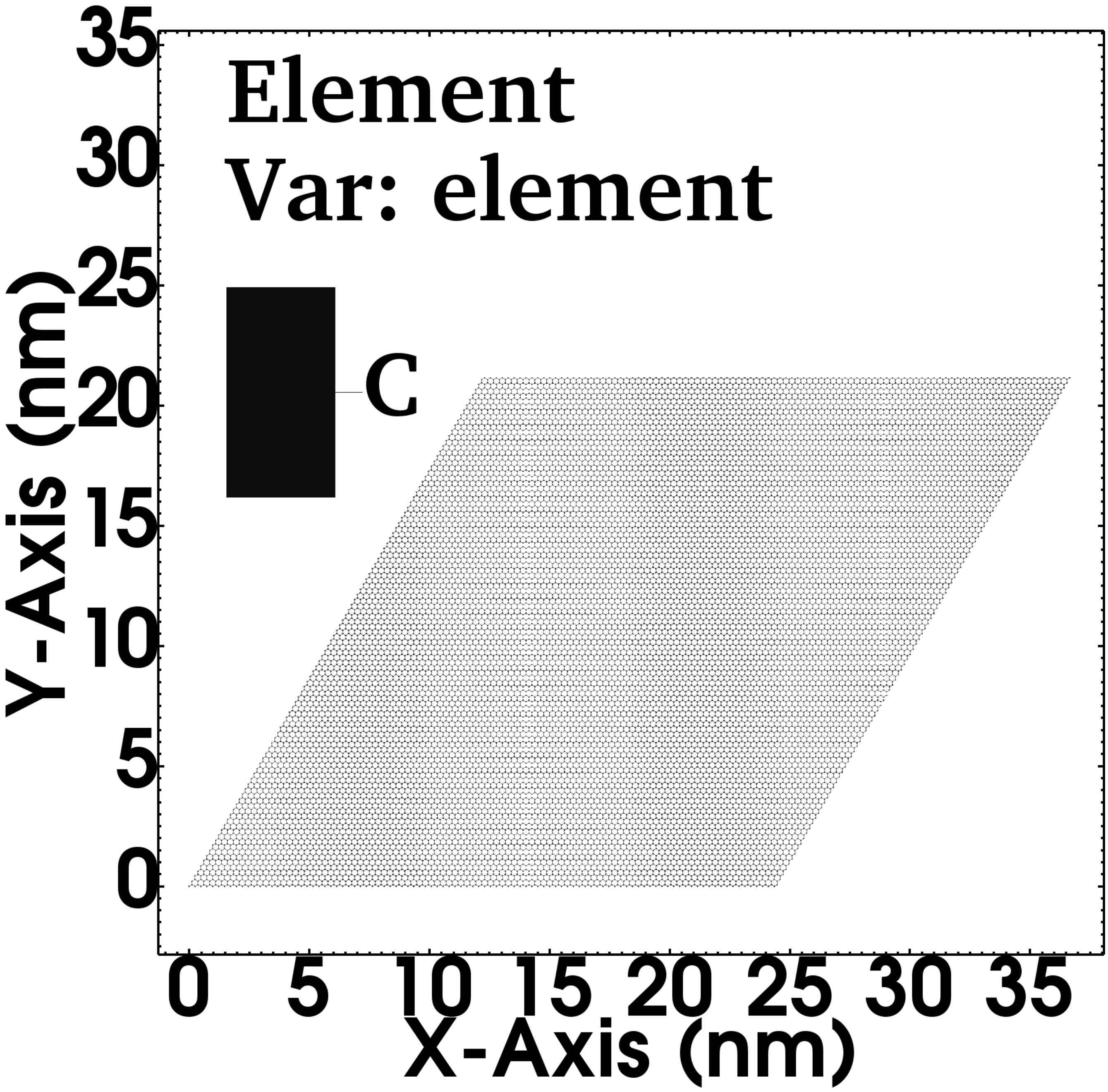

Figure 31. structure-flat

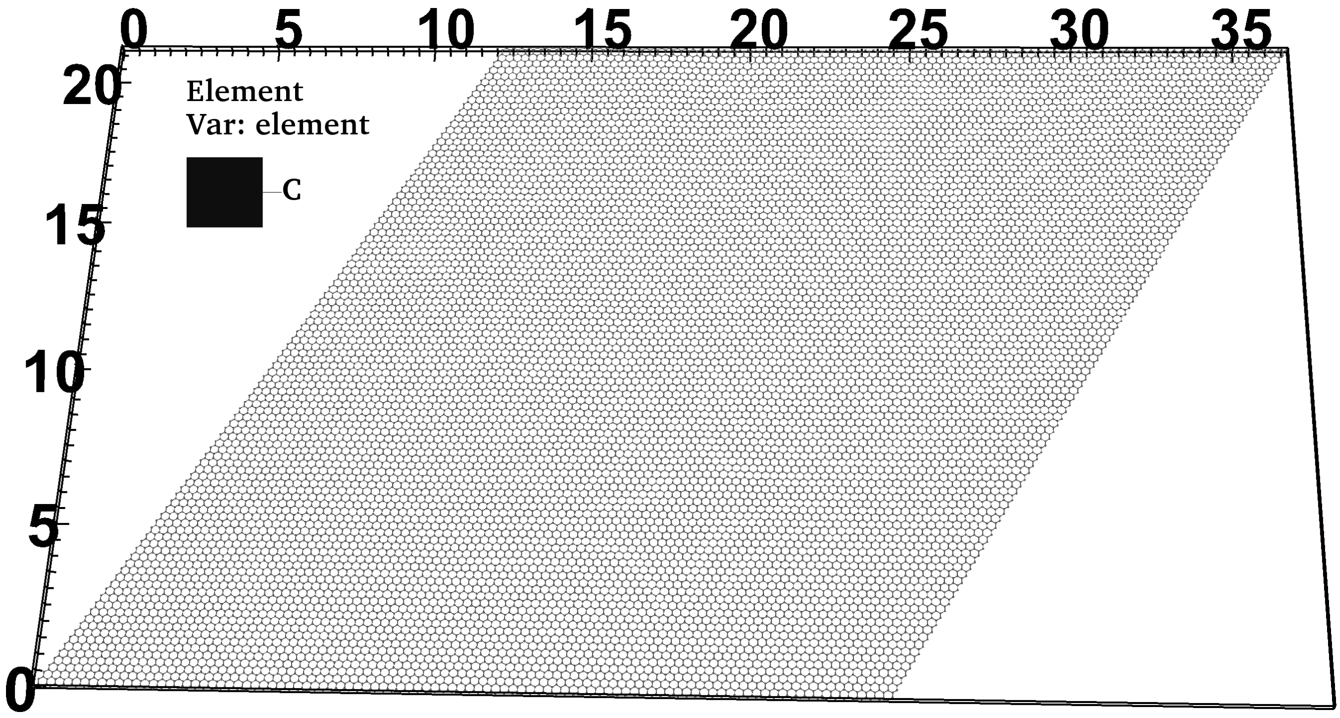

Figure 32. corrugate-5pm

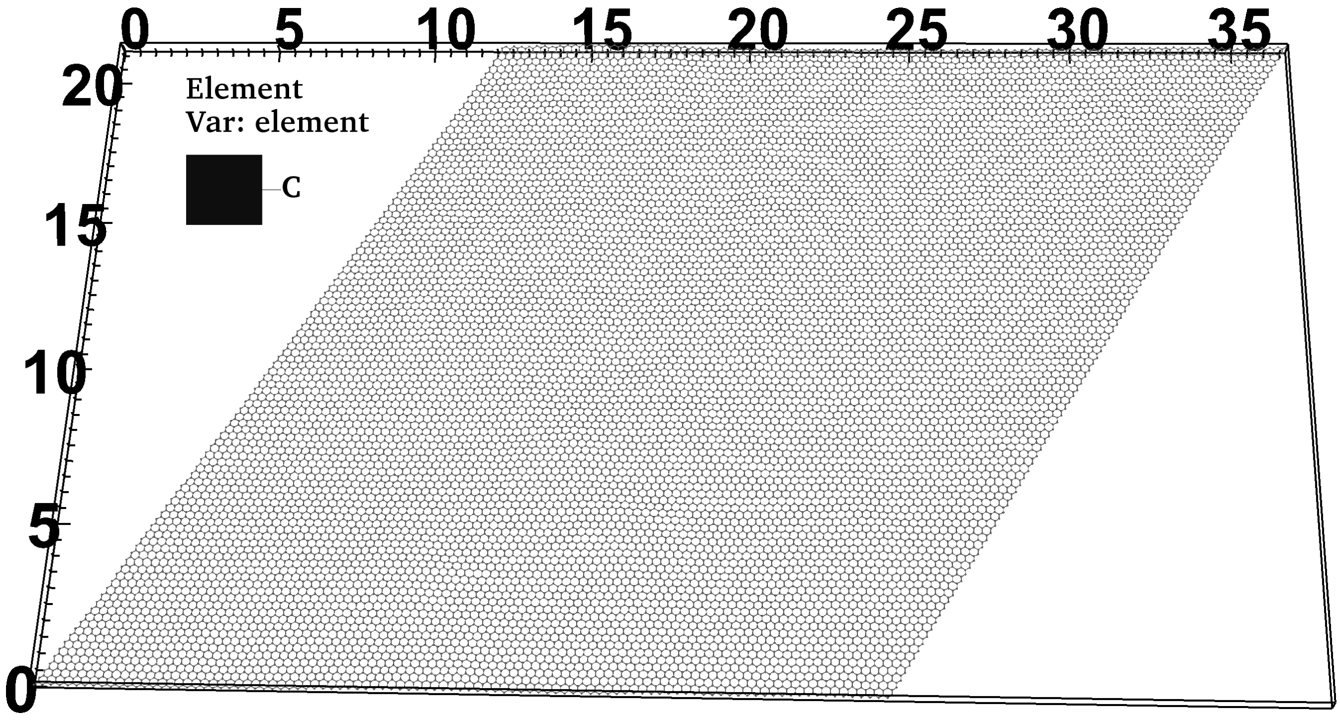

Figure 33. corrugate-10pm

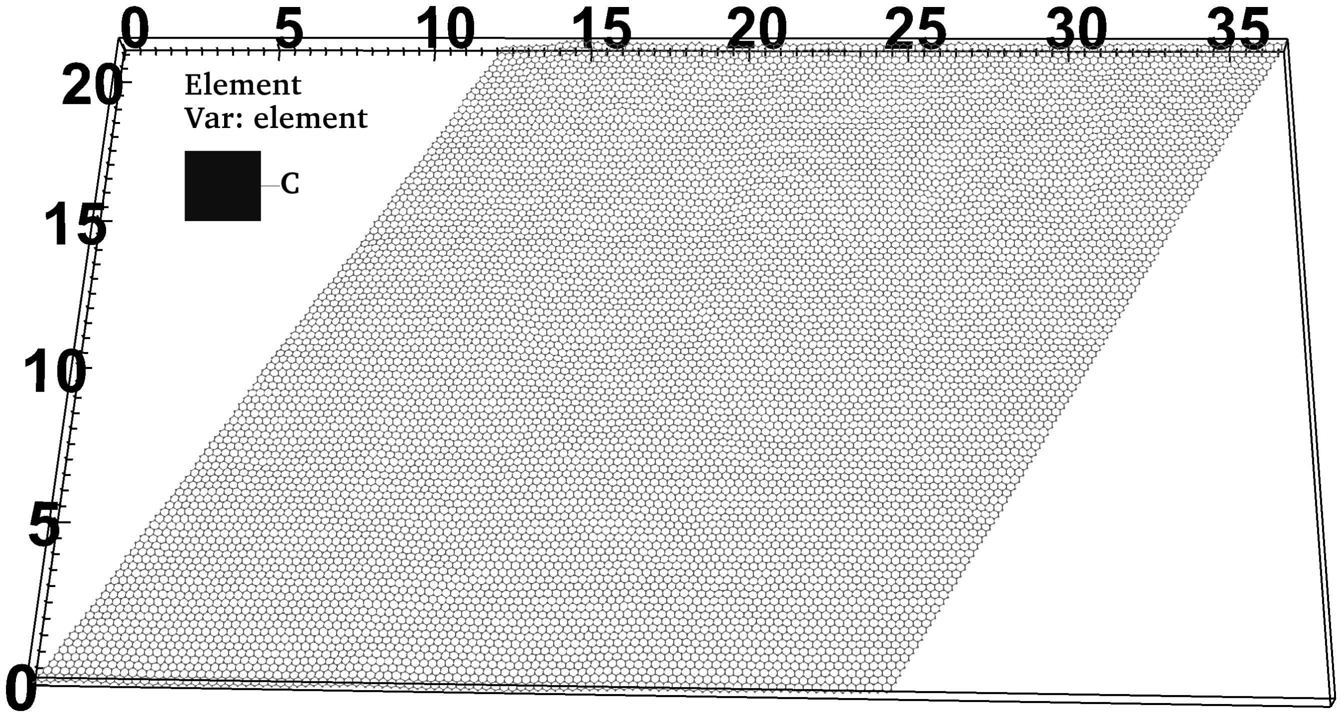

Figure 34. corrugate-15pm

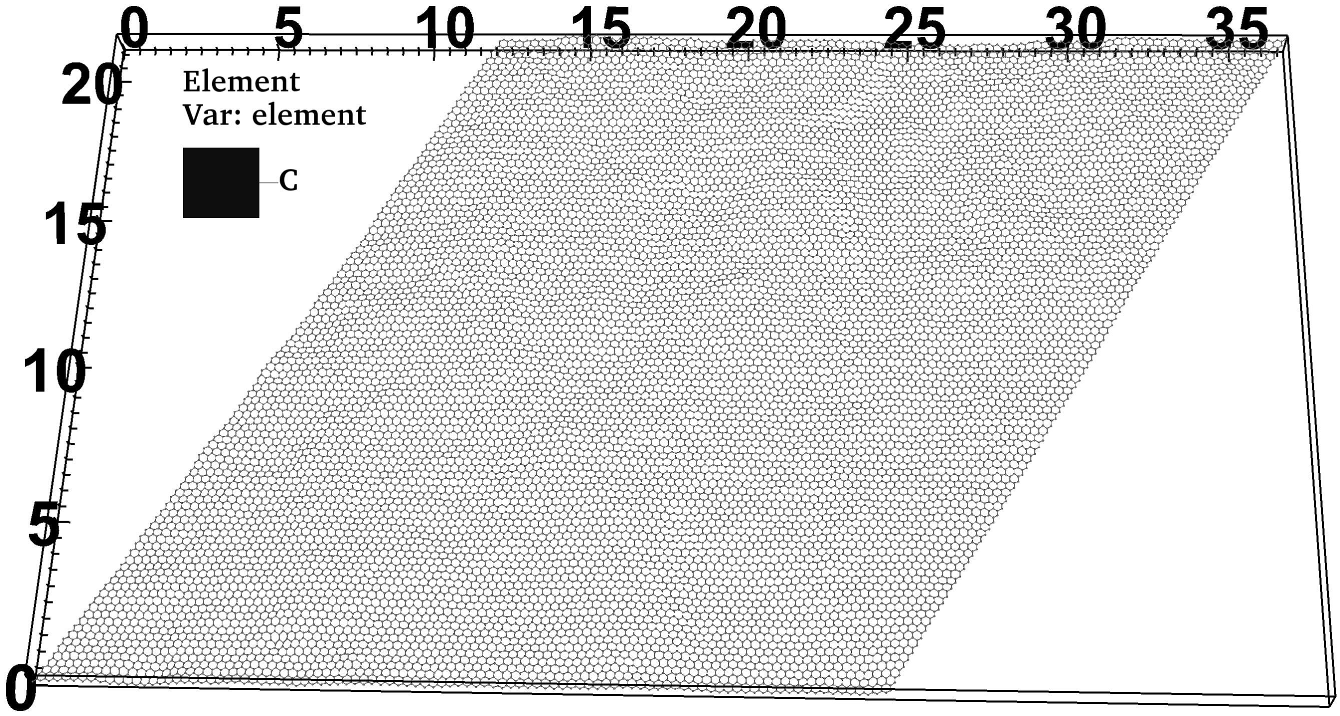

Figure 35. corrugate-20pm

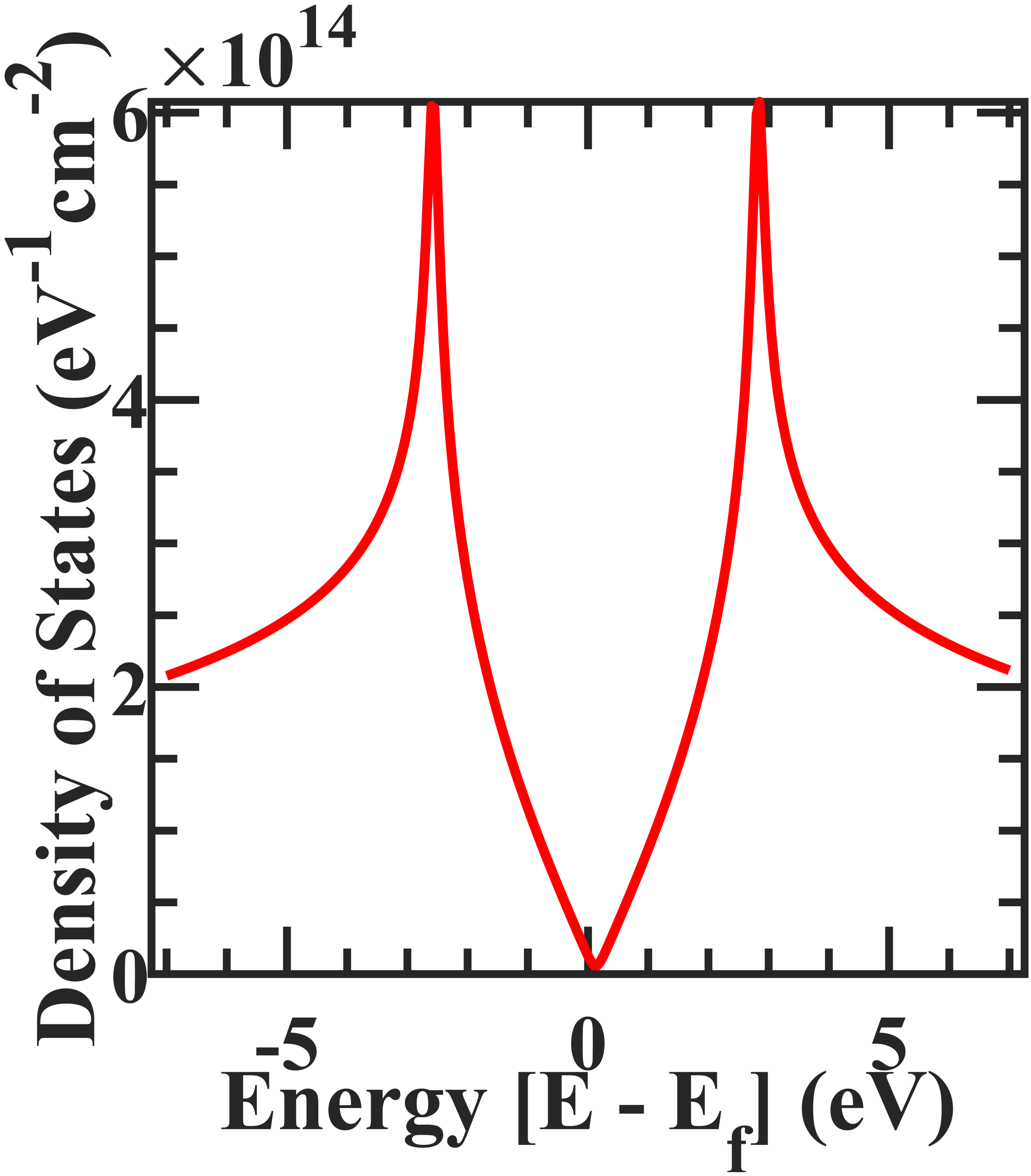

Figure 36. Density of state-flat

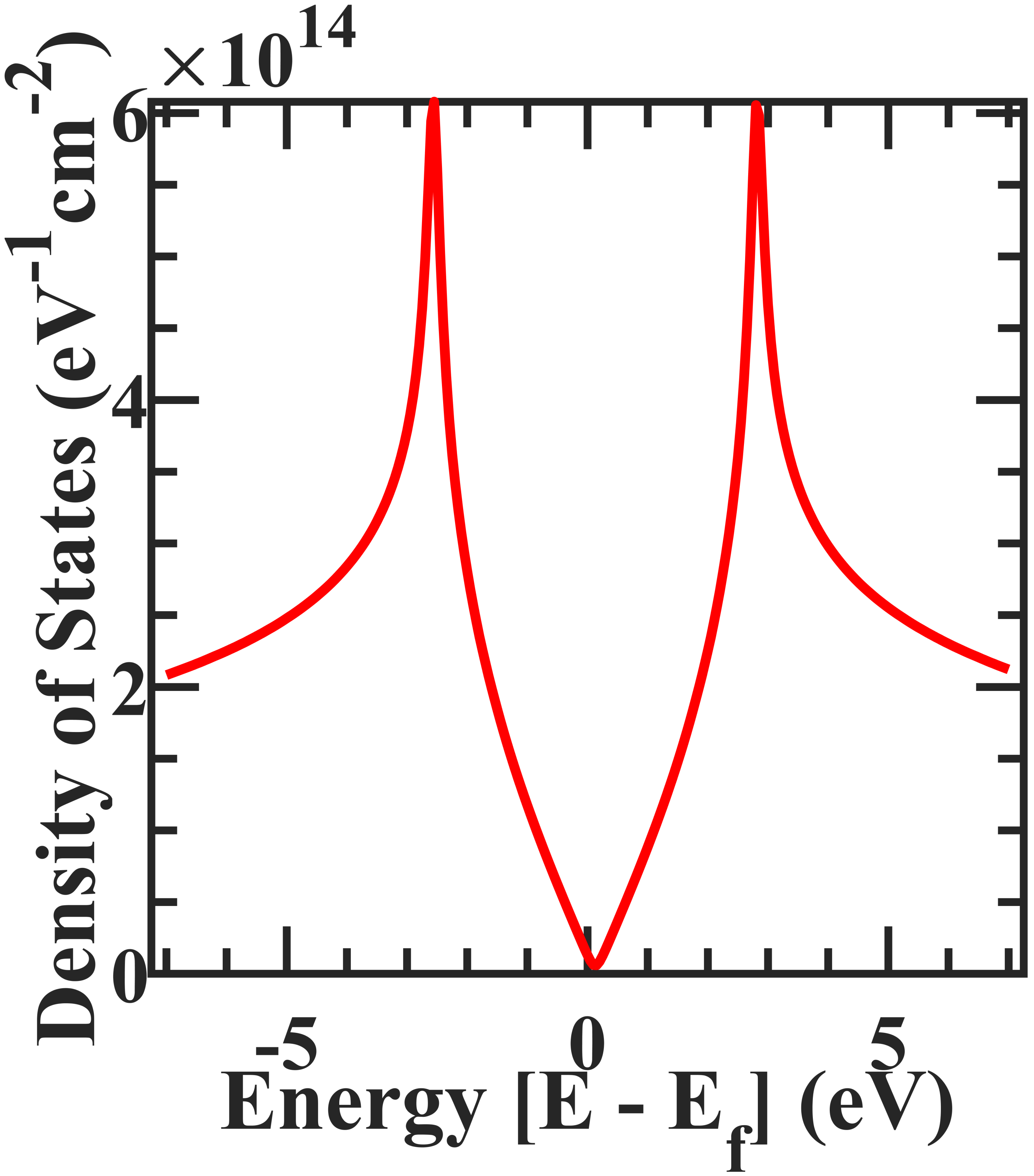

Figure 37. Density of state corrugate-5pm

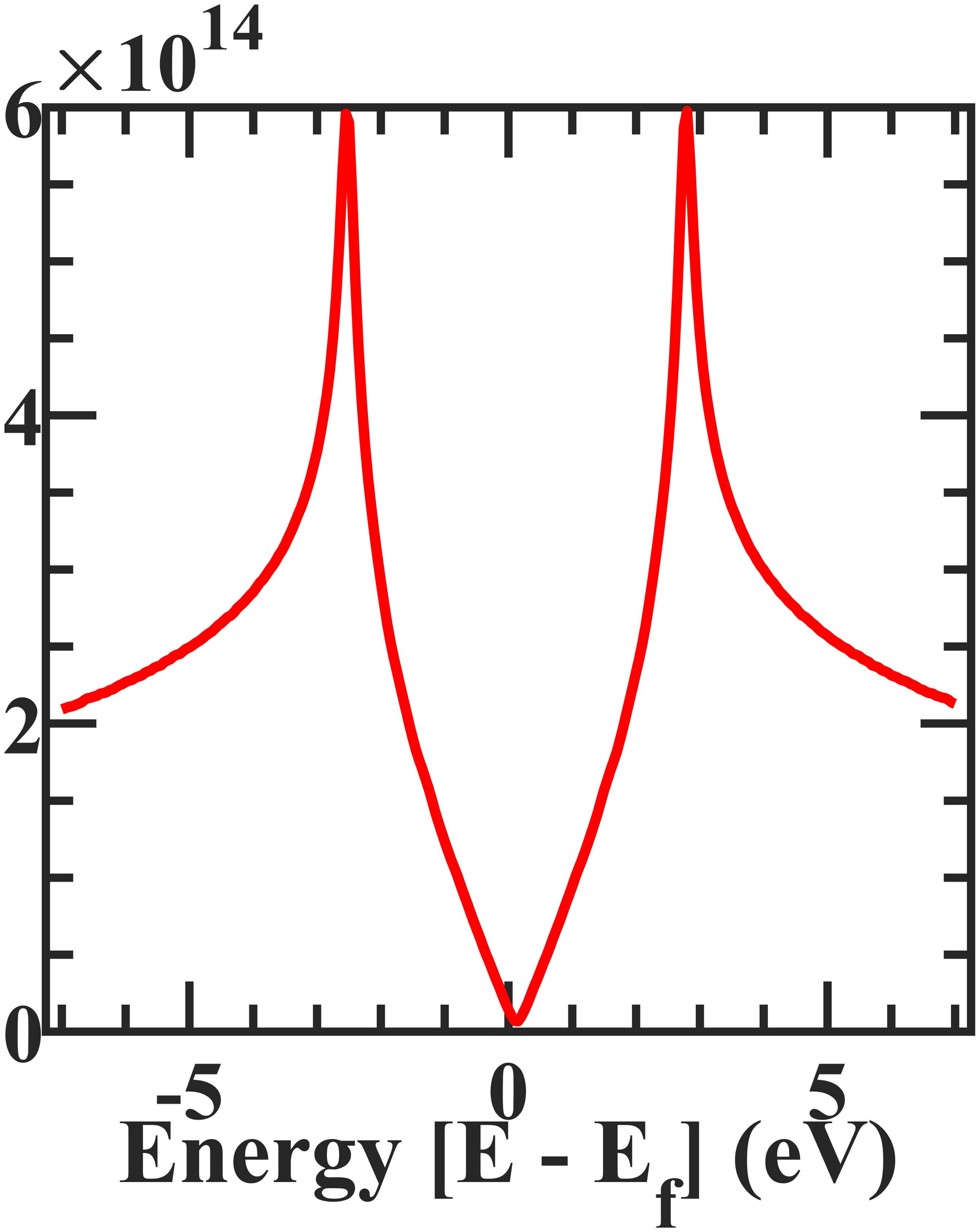

Figure 38. Density of state corrugate-10pm

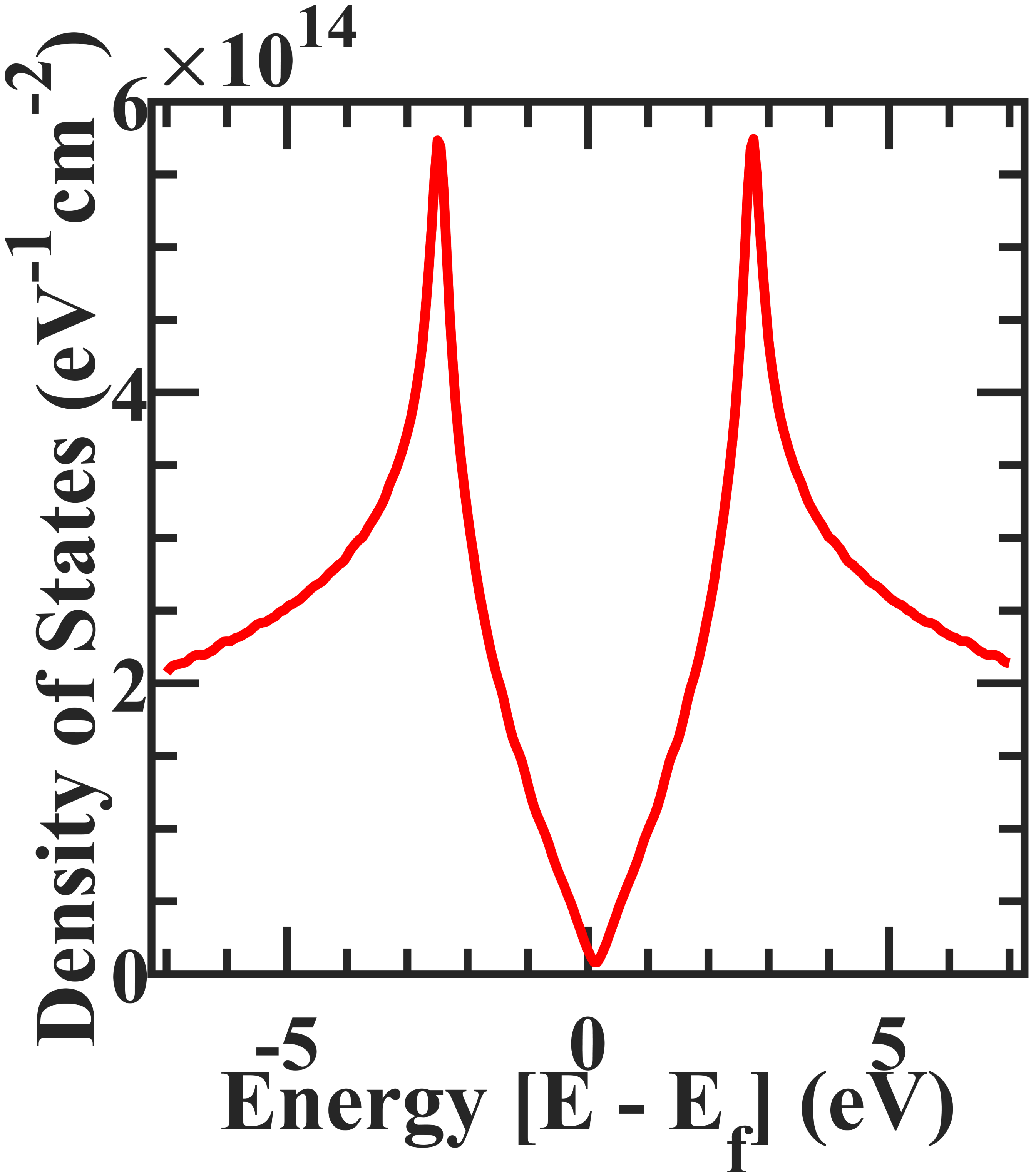

Figure 39. Density of state corrugate-15pm

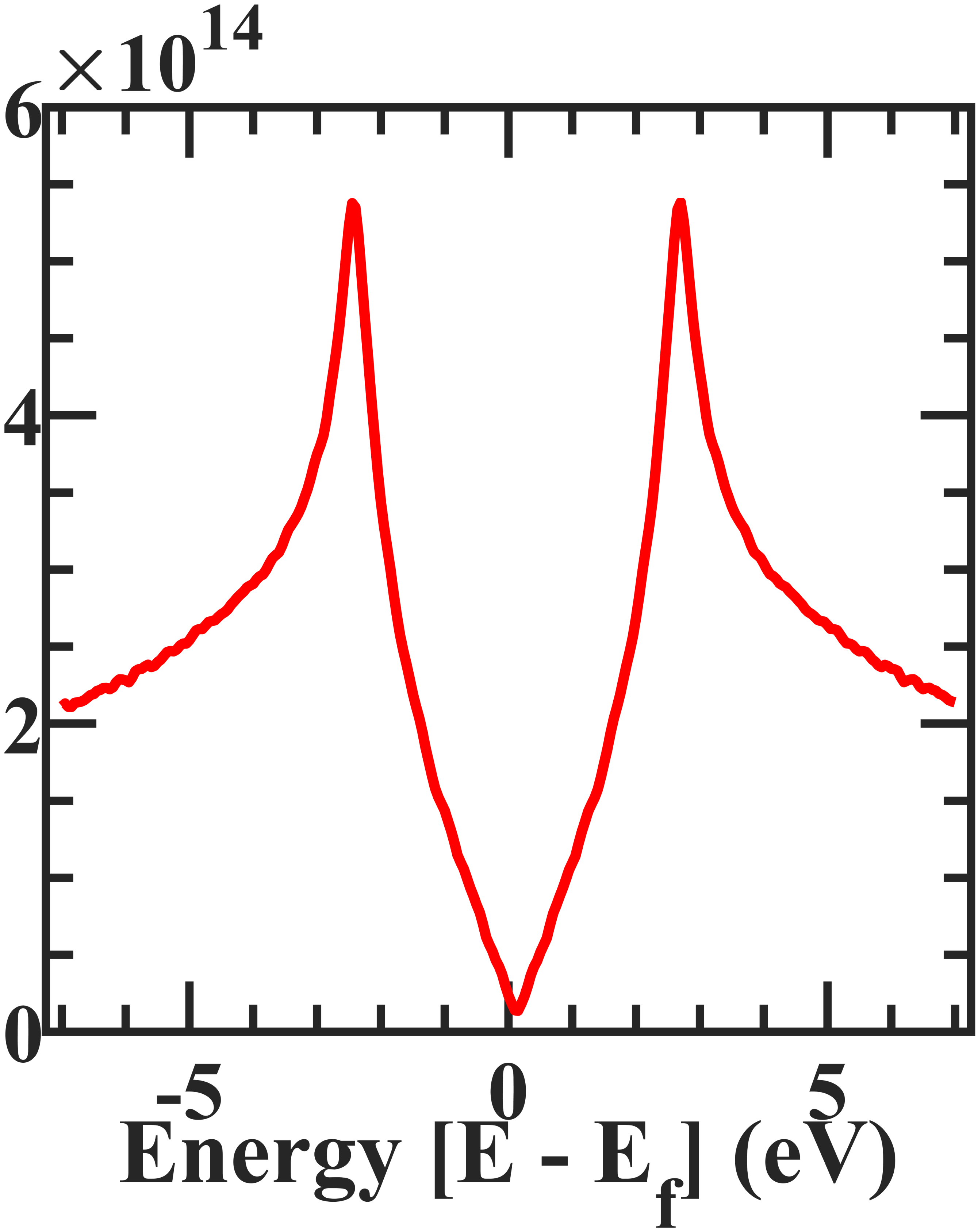

Figure 40. Density of state corrugate-20pm

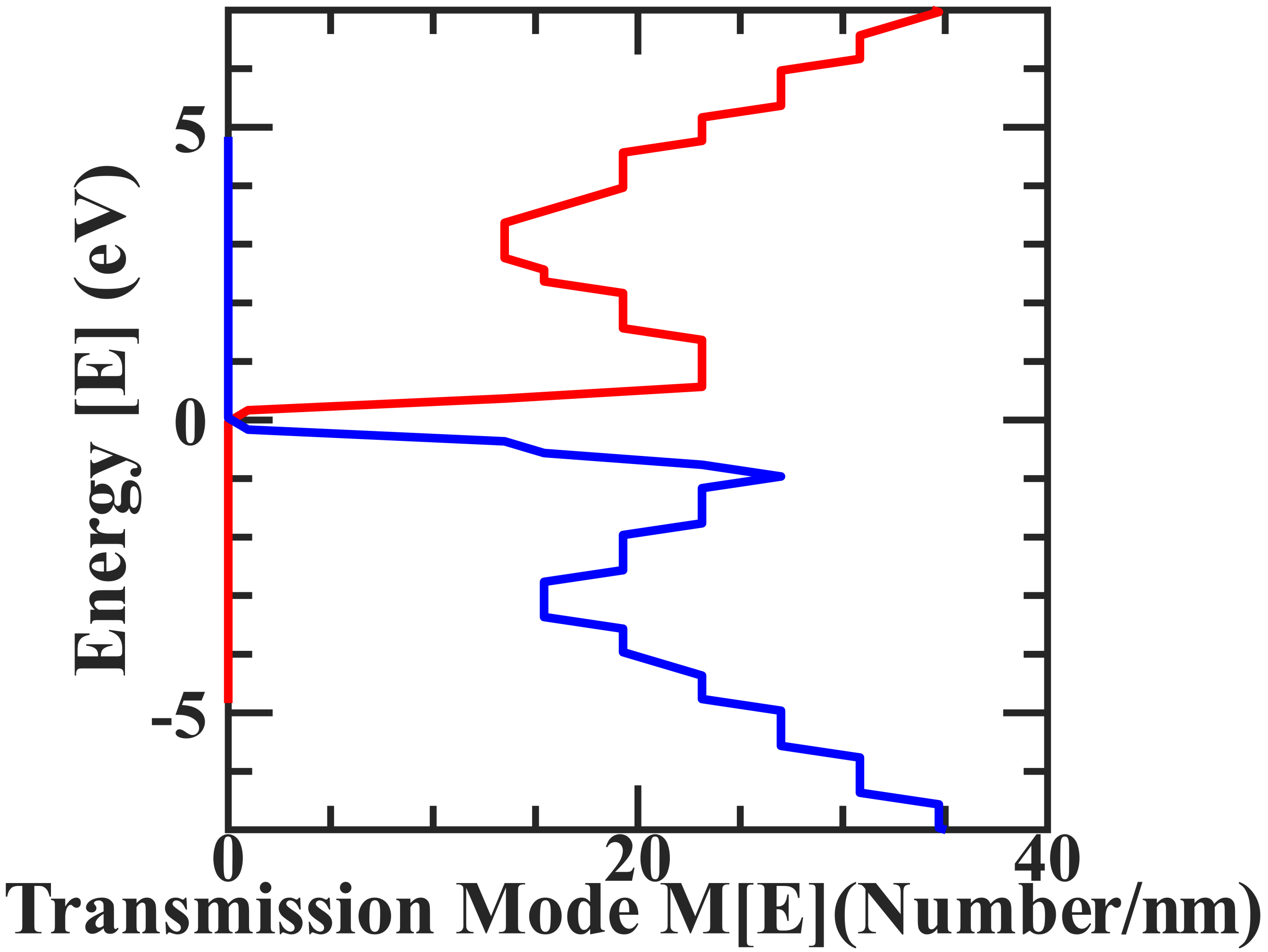

Figure 41. Density of Mode M(E) corrugate-5pm

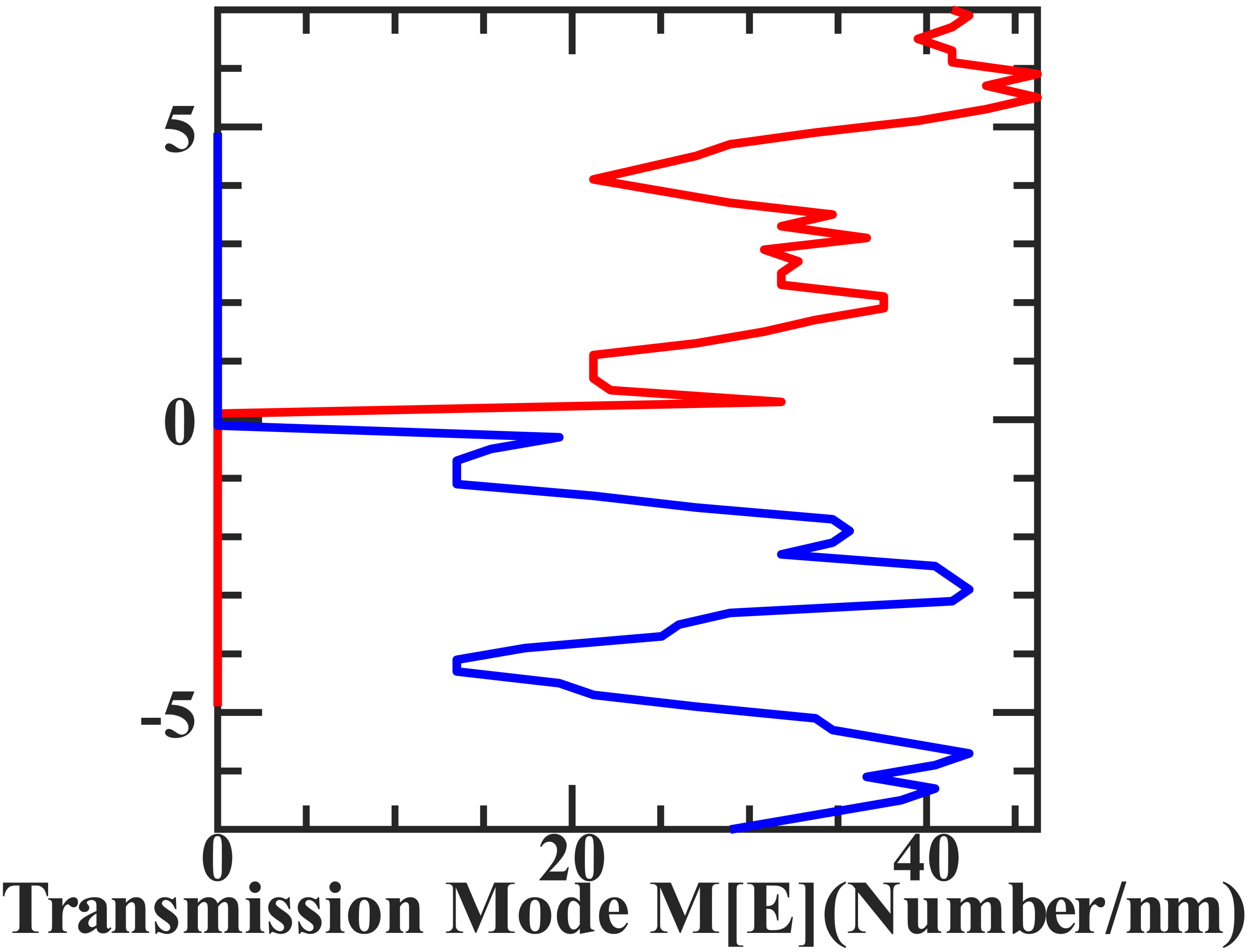

Figure 42. Density of Mode M(E) corrugate-10pm

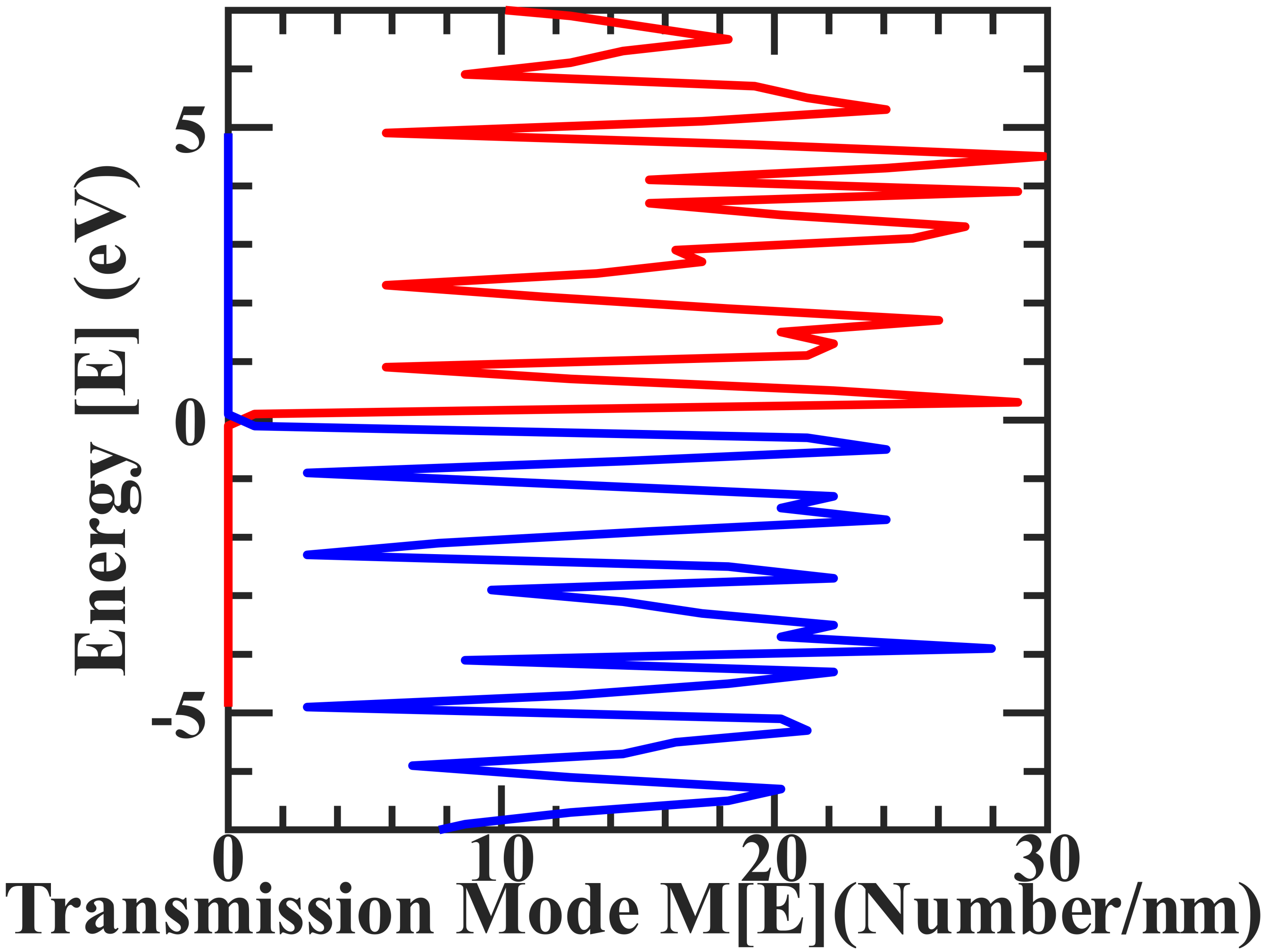

Figure 43. Density of Mode M(E) corrugate-15pm

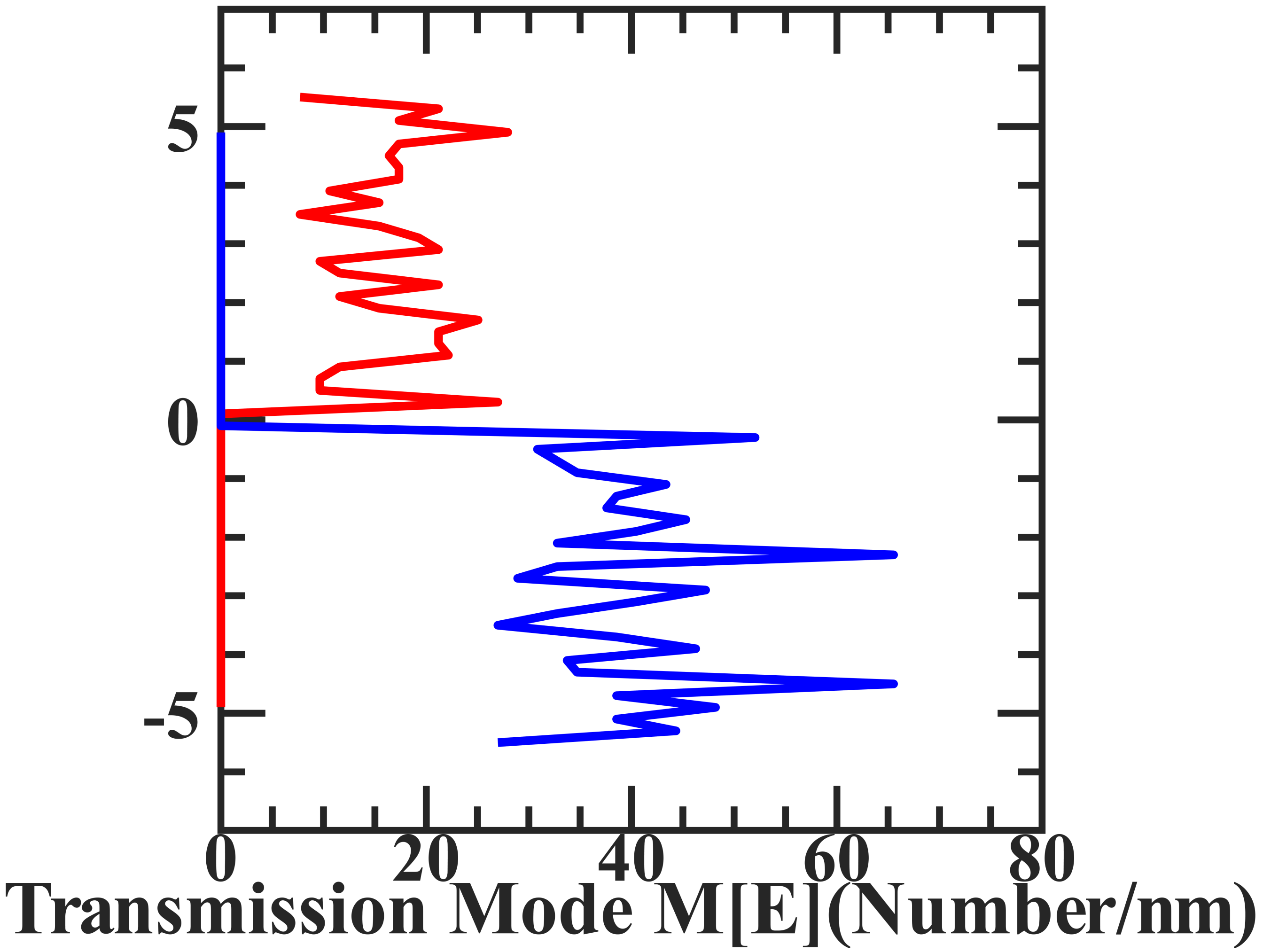

Figure 44. Density of Mode M(E) corrugate-20pm

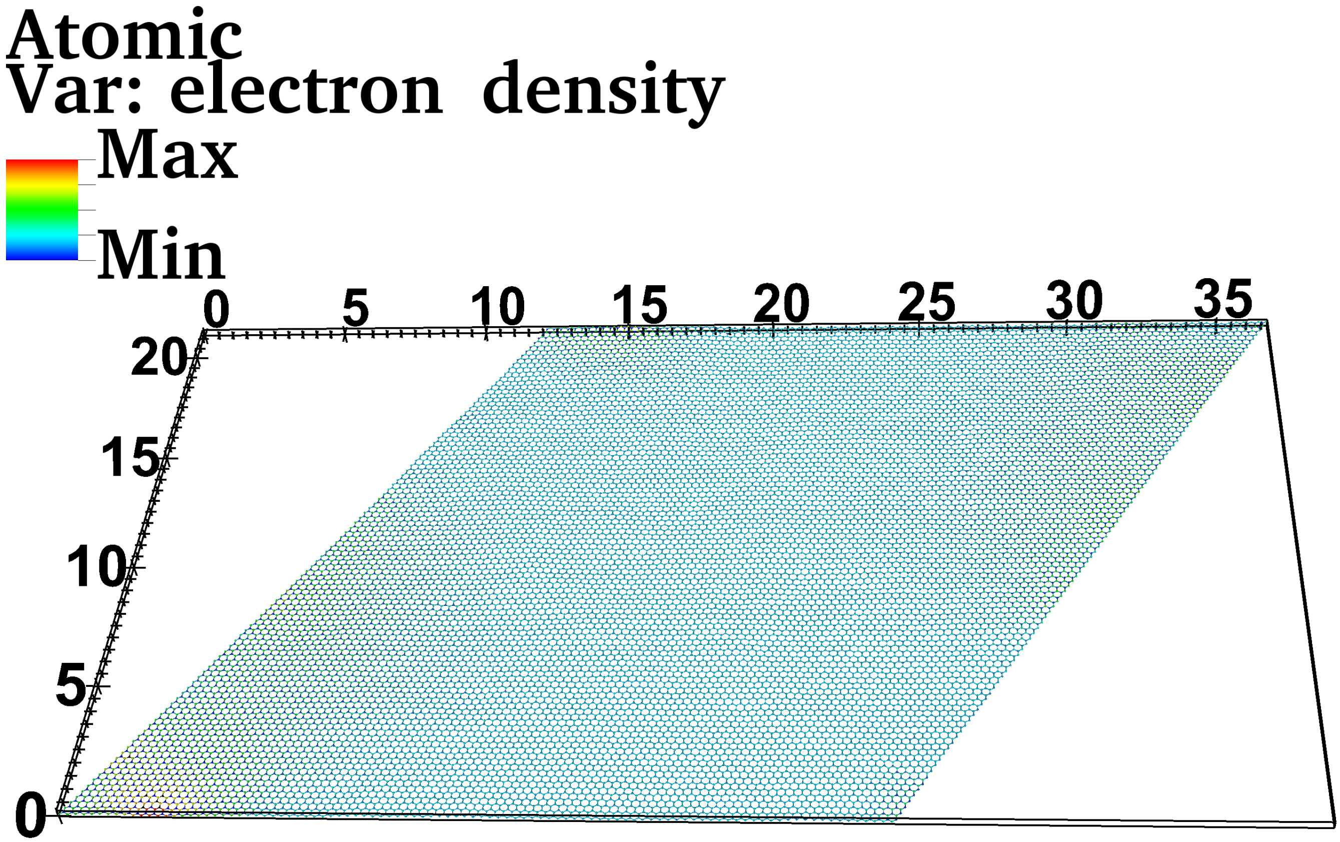

Figure 45. Electron density corrugate-5pm

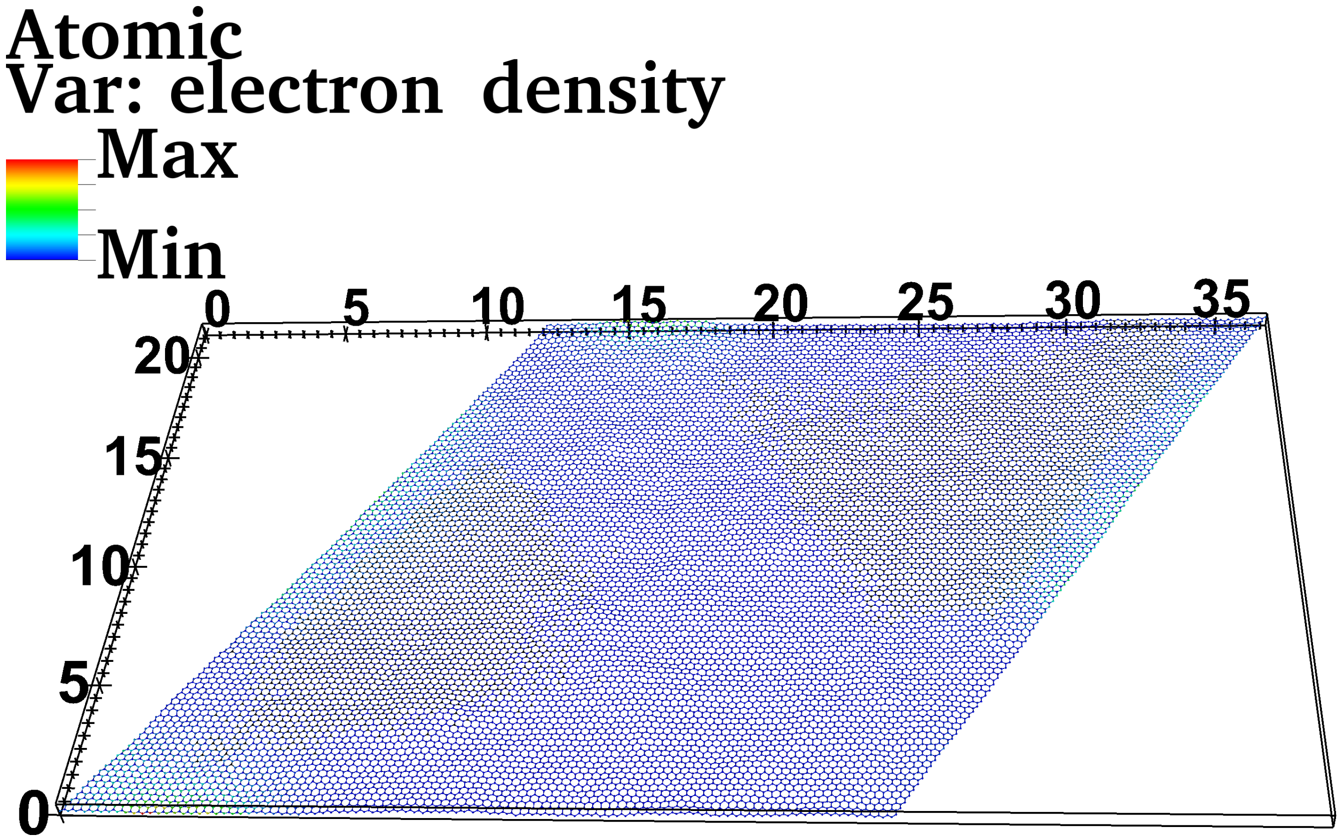

Figure 46. Electron density corrugate-10pm

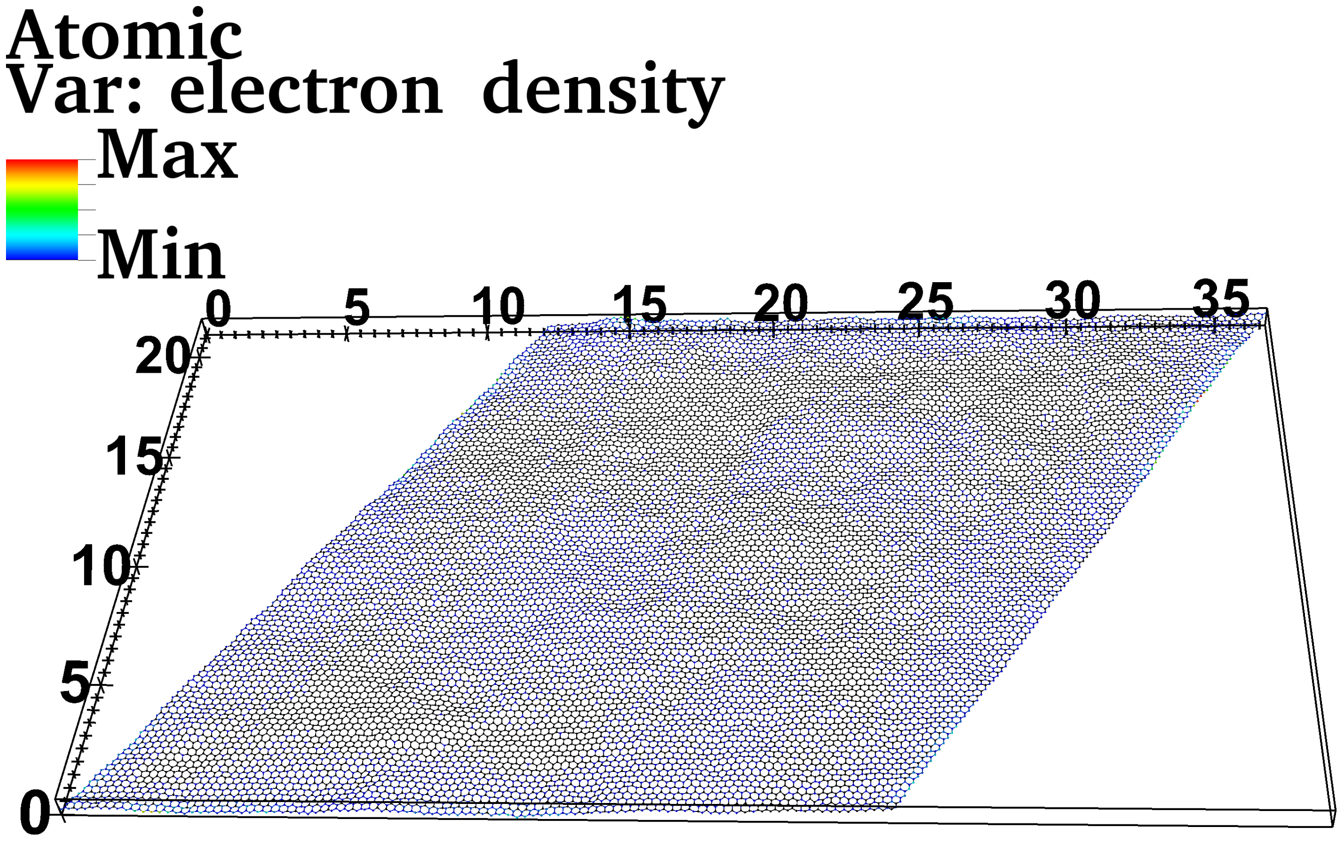

Figure 47. Electron density corrugate-15pm

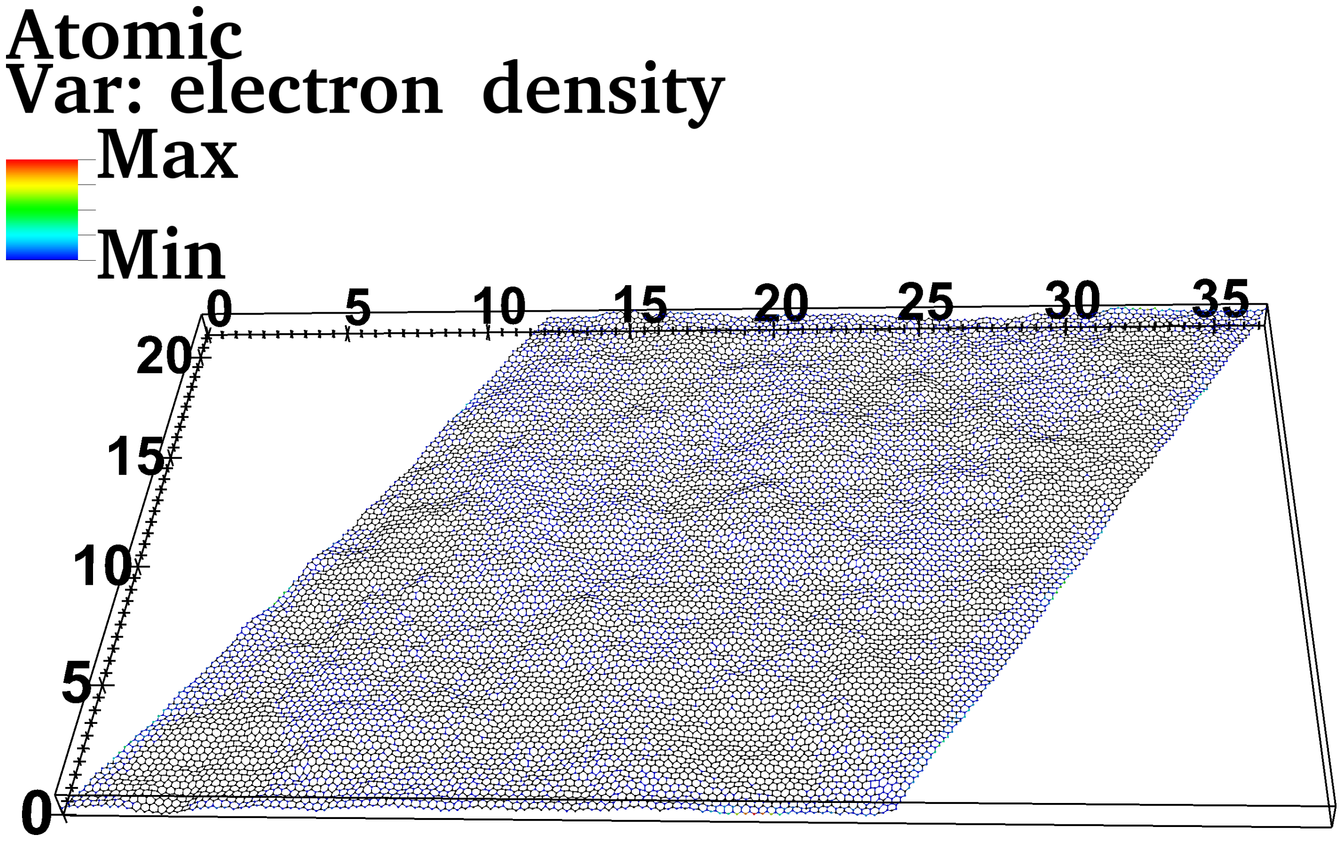

Figure 48. Electron density corrugate-20pm

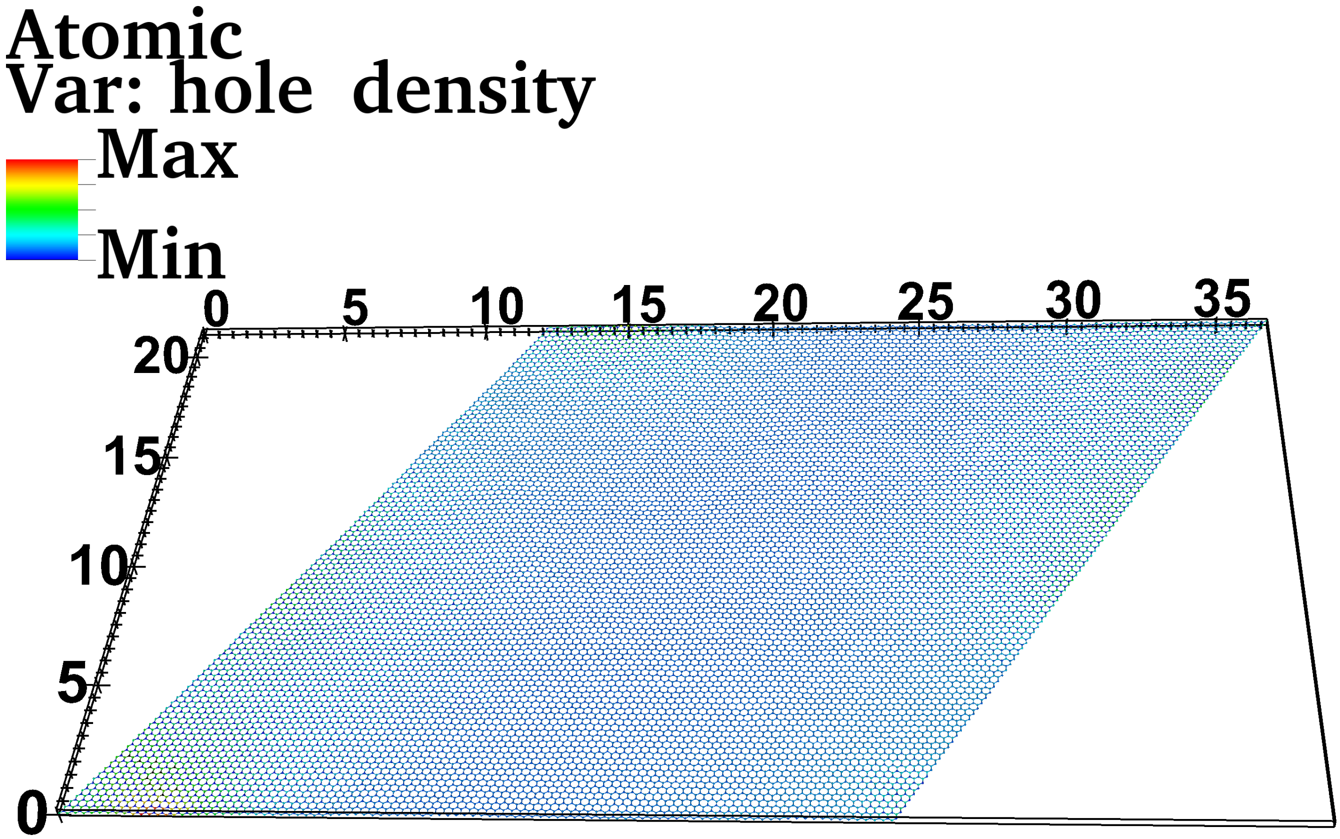

Figure 49. Hole density corrugate-5pm

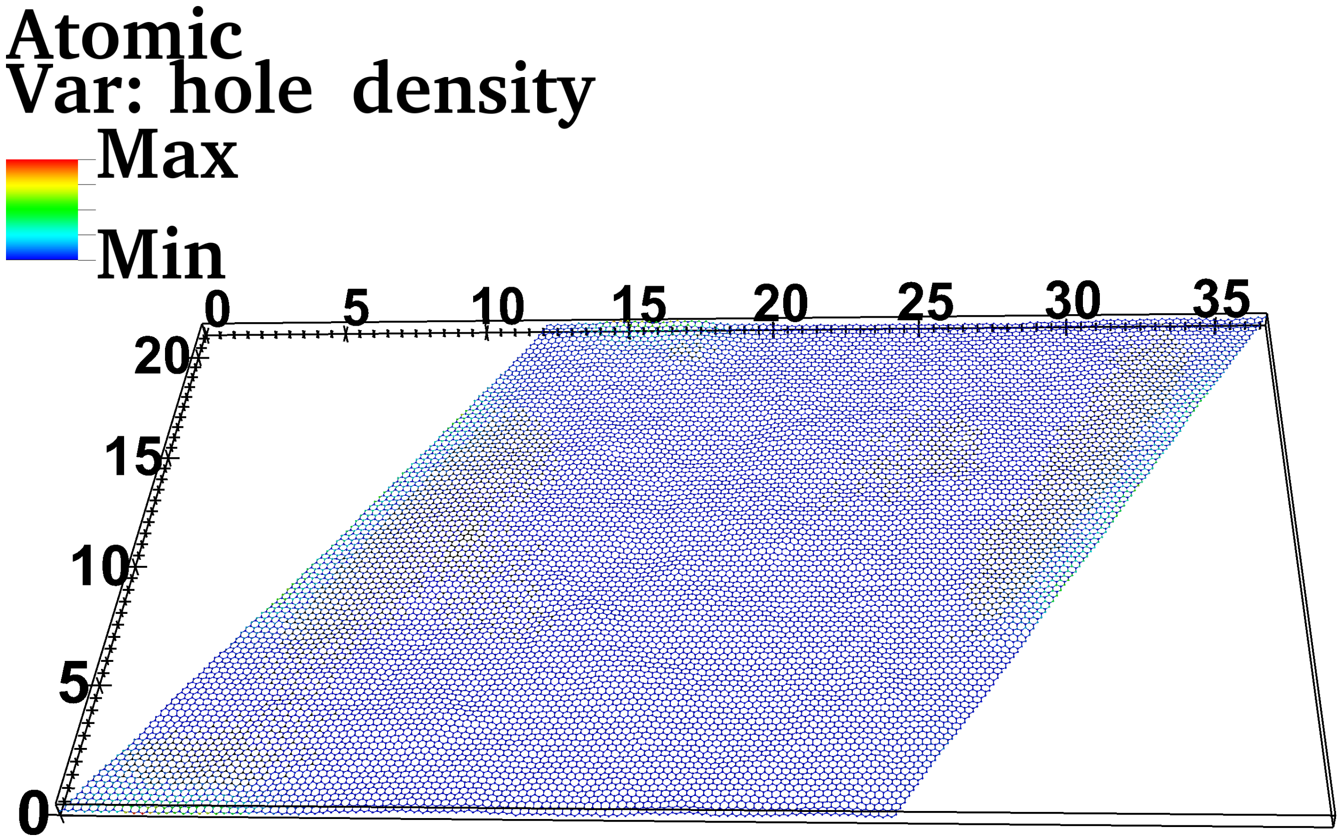

Figure 50. Hole density corrugate-10pm

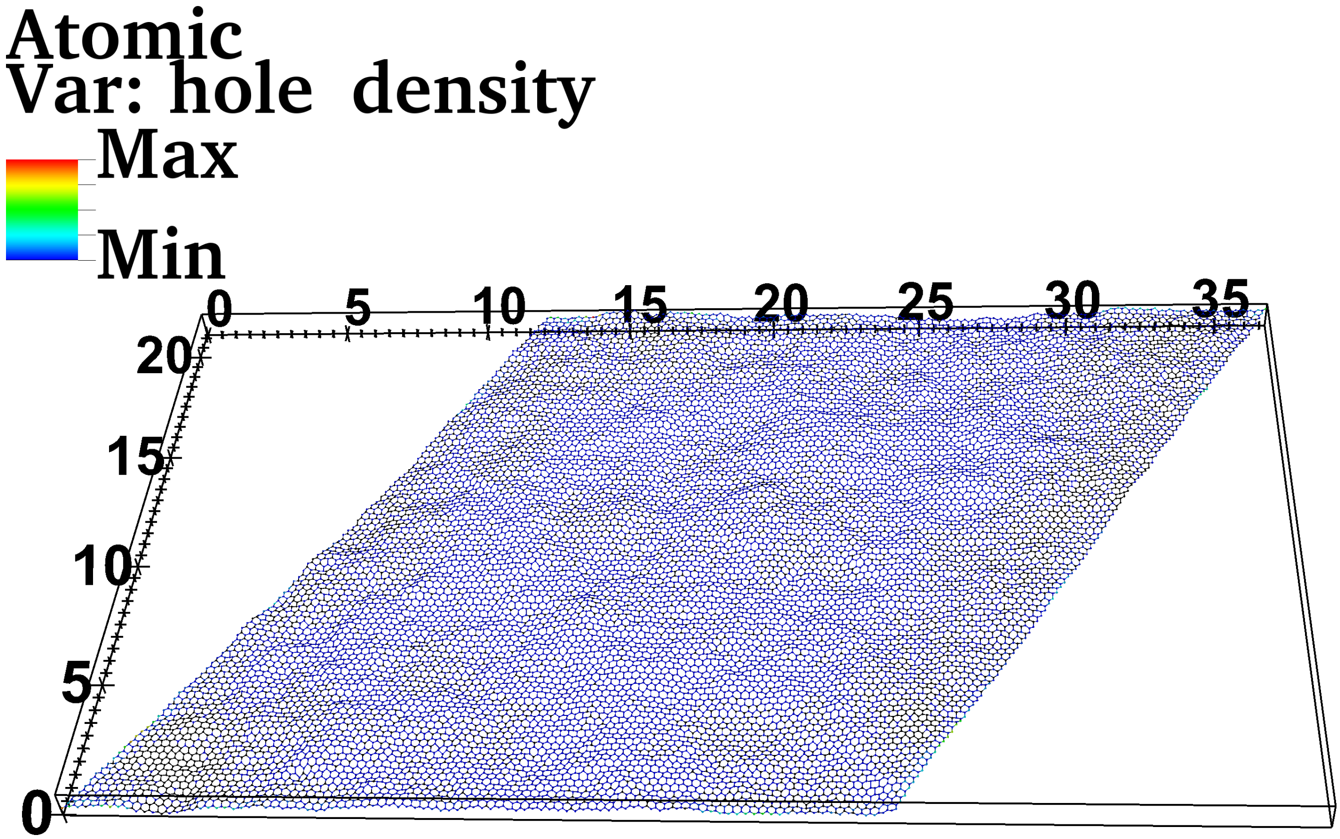

Figure 51. Hole density corrugate-20pm

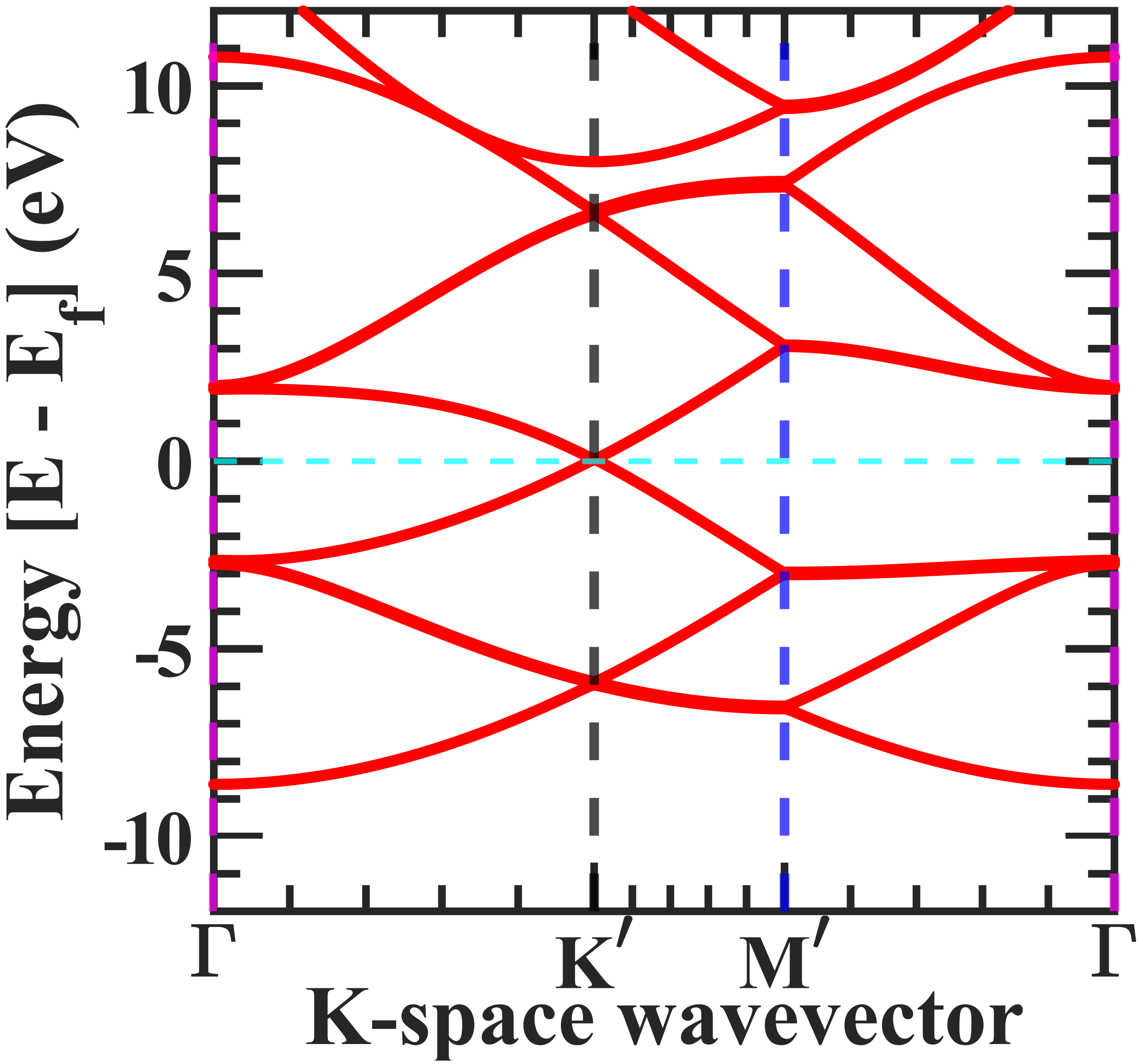

Figure 52. Band structure corrugate-5pm

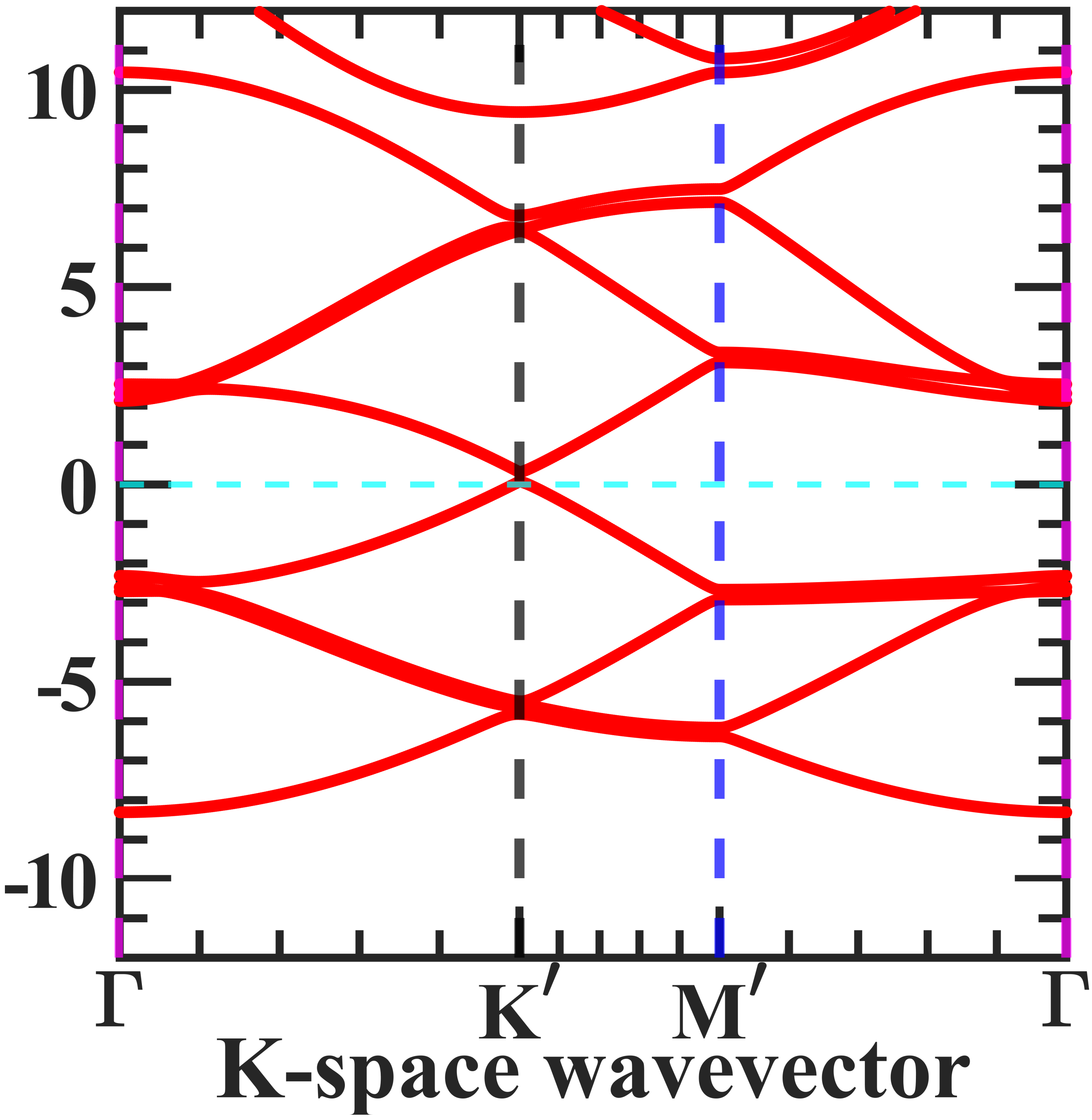

Figure 53. Band structure corrugate-10pm

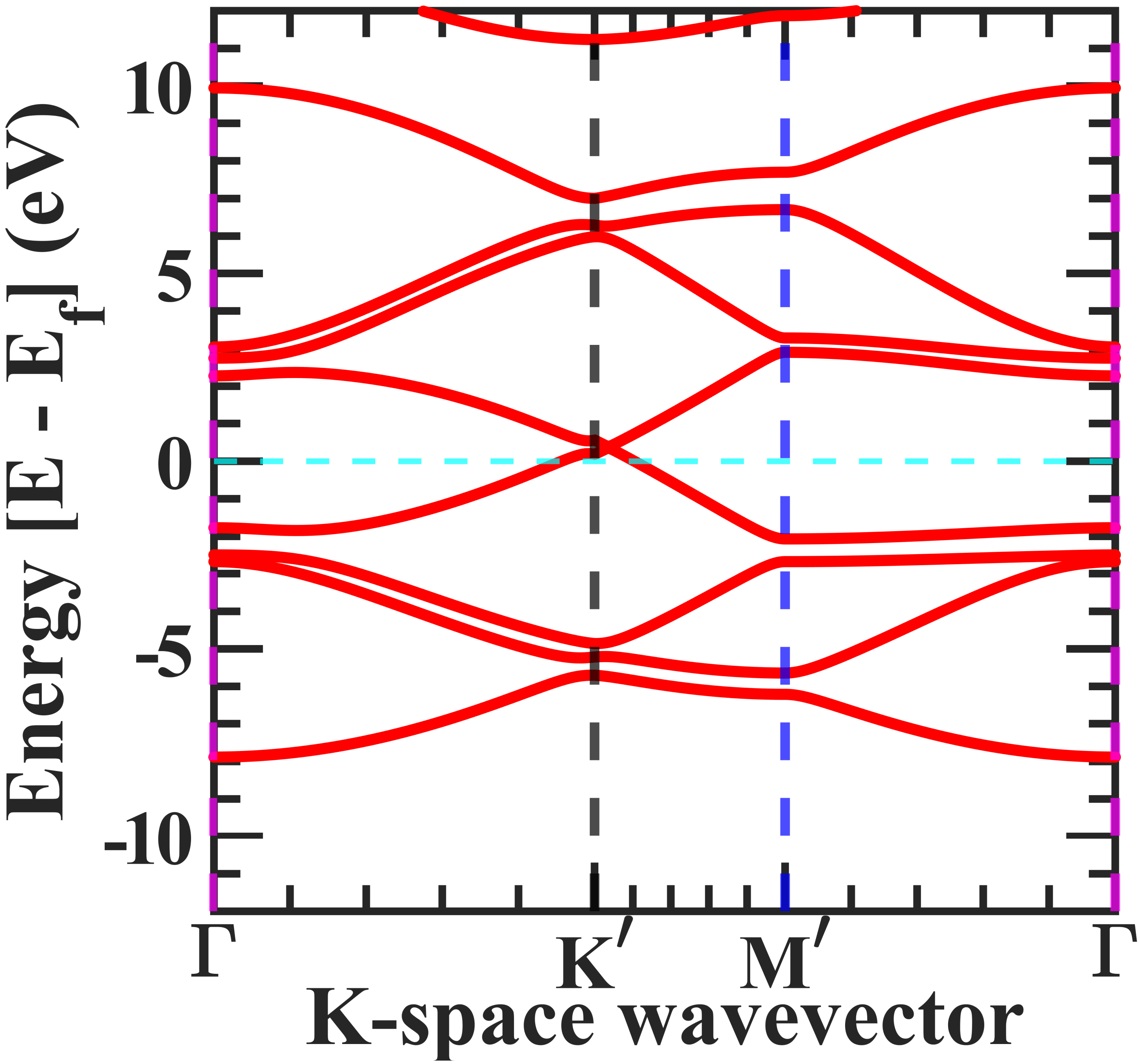

Figure 54. Band structure corrugate-15pm

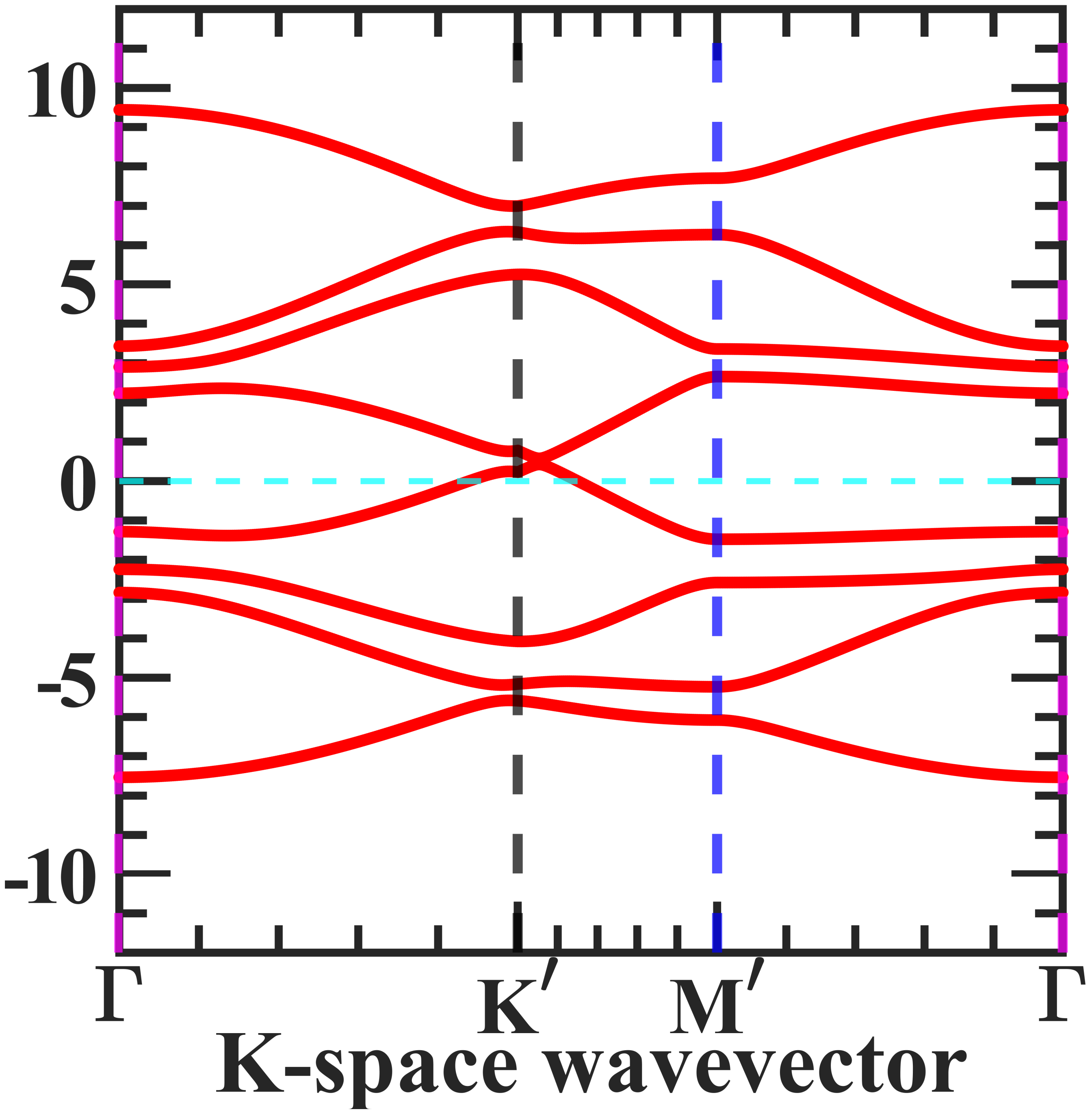

Figure 55. Band structure corrugate-20pm

10x10 graphene orthorhombic supercell

Next, We have also simulated a two-dimensional orthorhombic device structure to investigate whether a four atom per unit supercell will influence similarly or have different characteristics. This geometrical structure can be further tailored to make an armchair and zigzag nanoribbon configuration based upon confinement direction, boundary condition, and edge topology. The fig. 56 correspond to orthorhombic flat 10x10 graphene supercell. fig. 57 to fig. 60 correspond to the corrugate device structure with roughness varying from 5 pm to 20 pm, mimicking the roughness profile of h-BN and SiO_2 substrate. Similarly, fig. 61 to fig. 65 represents the electronic density of mode M(E) in the x-y direction, fig. 66 to fig. 70 represents the electronic density, fig. 71 to fig. 75 represents the hole density and fig. 76 to fig. 80 represents the electronic bandstructure of the corresponding device structure. In all, the corrugate structure correlation is kept constant at 10nm length. In the simulated device, the orthorhombic unit cell has four atoms per cell, and a total of 400 atoms are simulated. The P-D tight-binding

model contains three orbitals, namely carbon P_z, and carbon-hydrogen passivated D_{yz}, D_{xz} orbitals. Therefore total degree of freedom in hamiltonian is 1200 variable-sized orbitals. The *P* and *X* are high symmetric points corresponding to the folded reduced BZ-zone of graphene orthorhombic supercell.

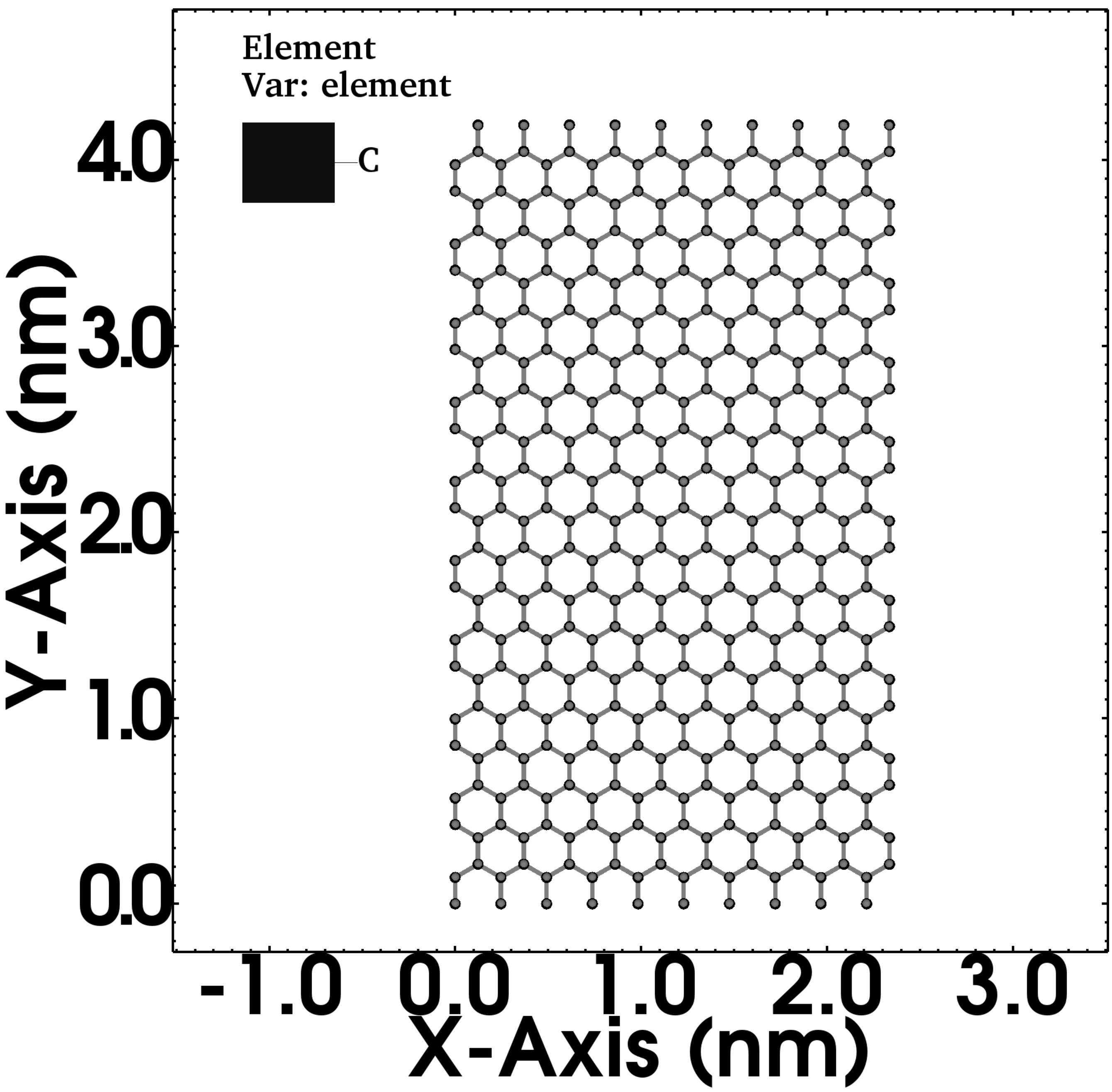

Figure 56. structure-flat

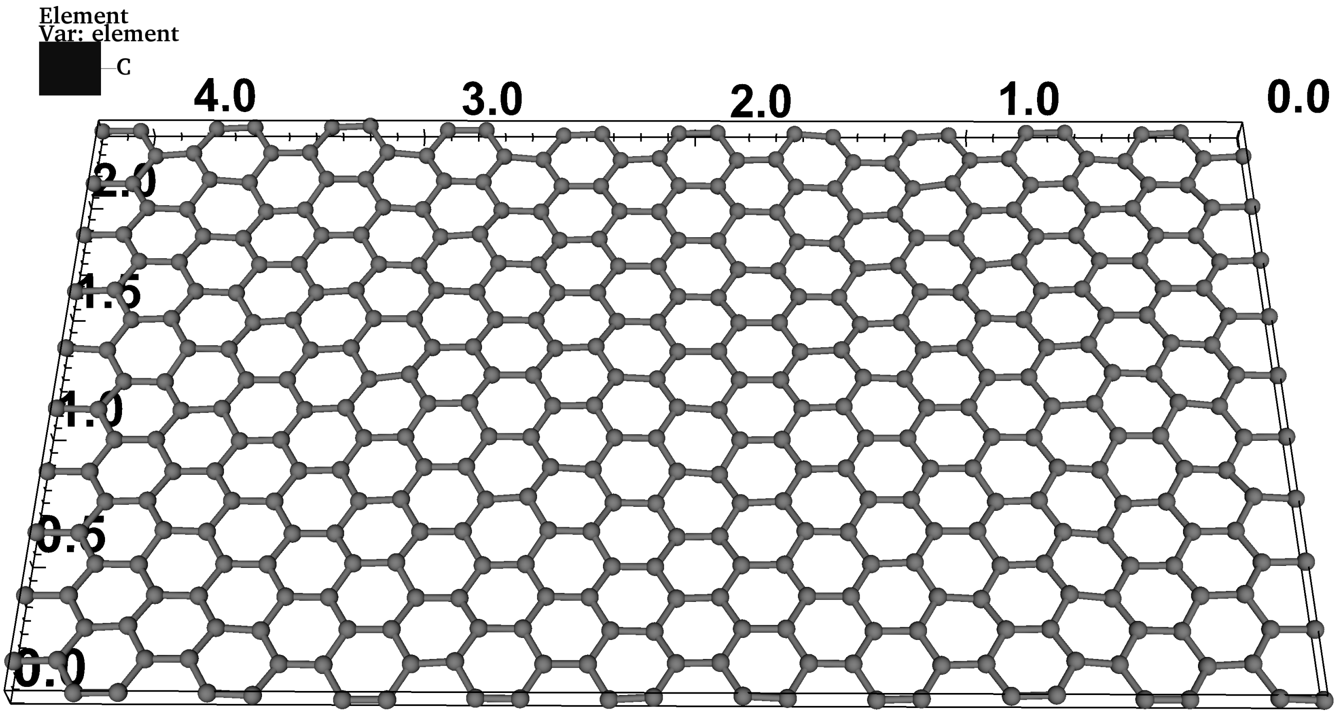

Figure 57. corrugate-5pm

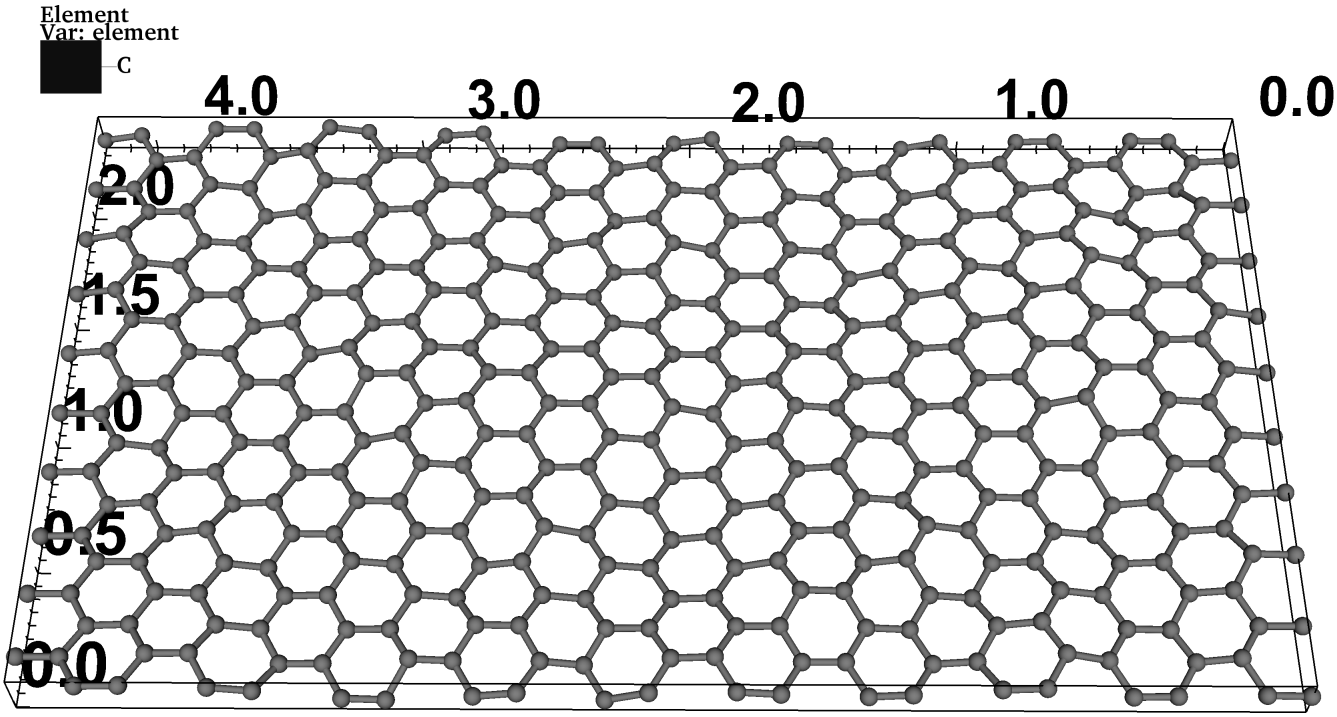

Figure 58. corrugate-10pm

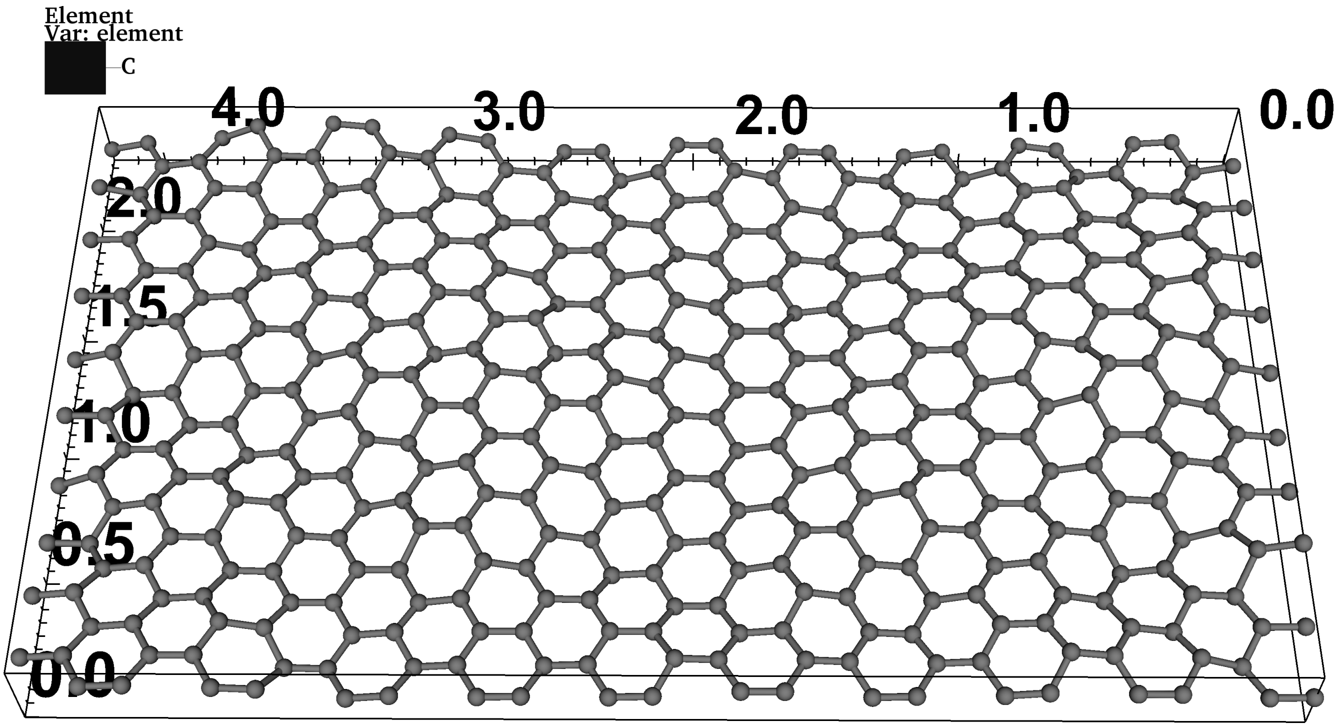

Figure 59. corrugate-15pm

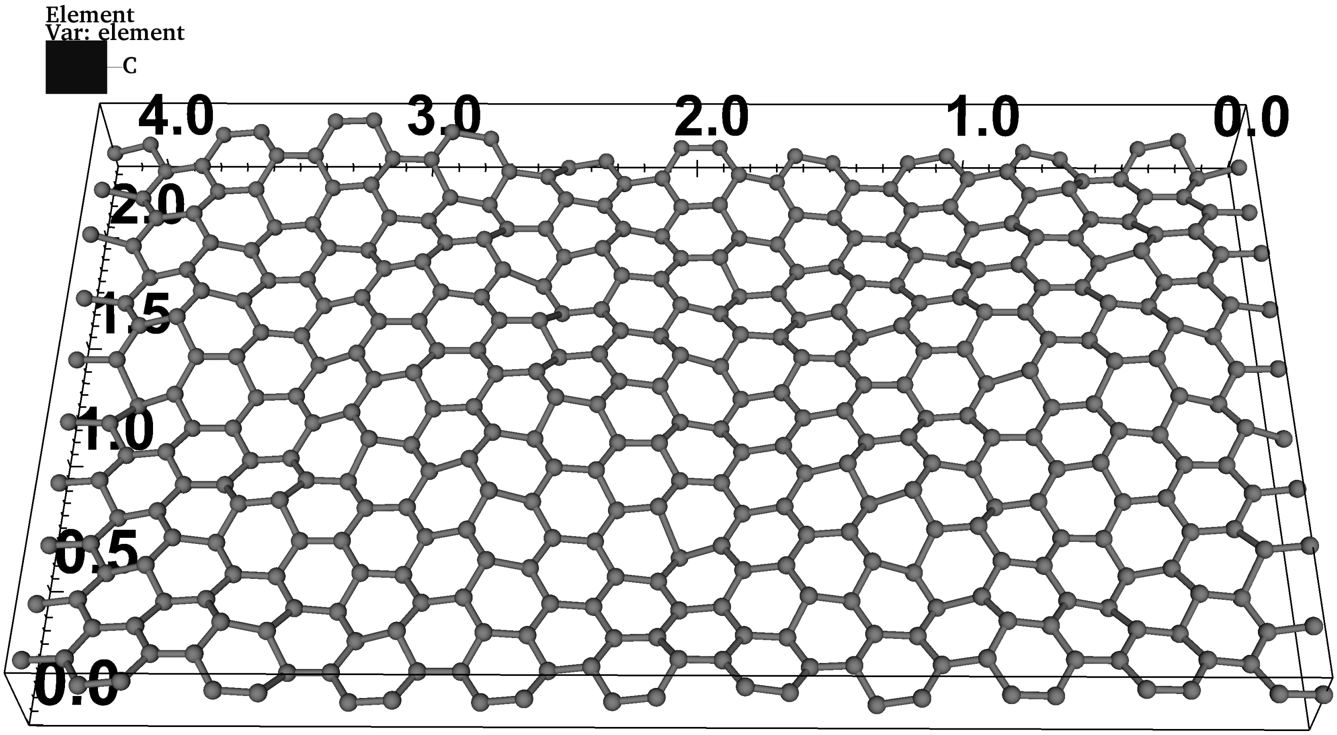

Figure 60. corrugate-20pm

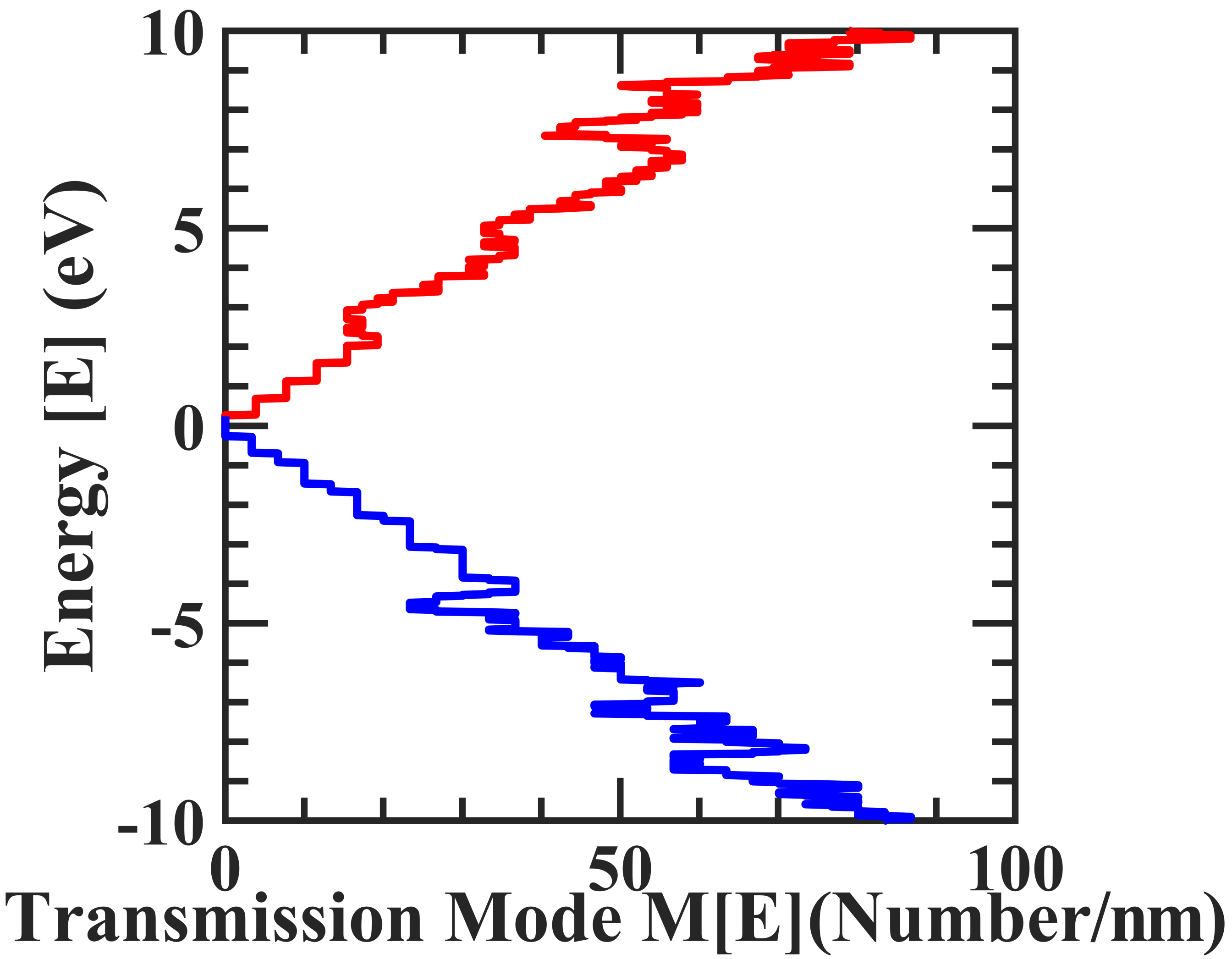

Figure 61. Density of Mode M(E) flat

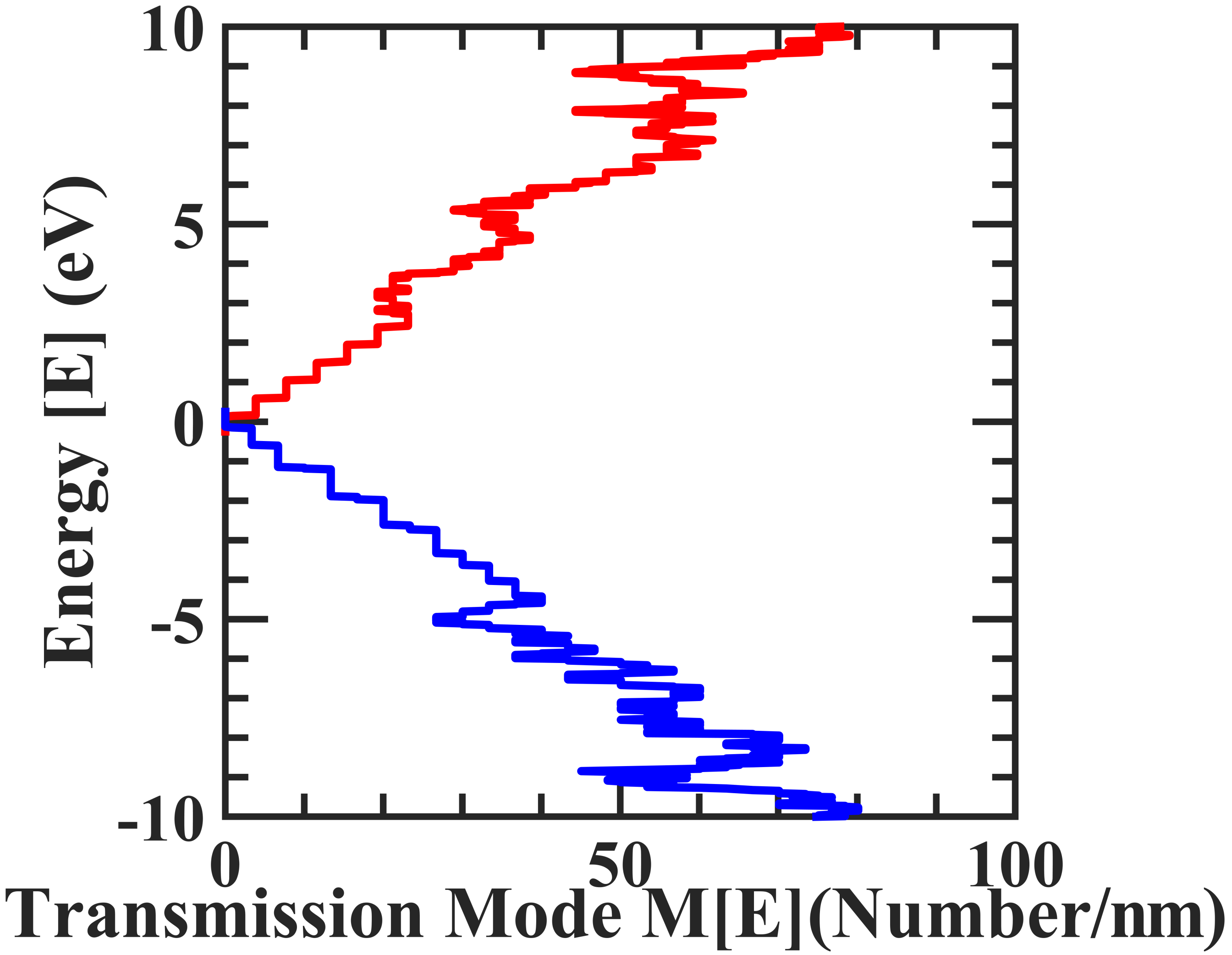

Figure 62. Density of Mode M(E) corrugate-5pm

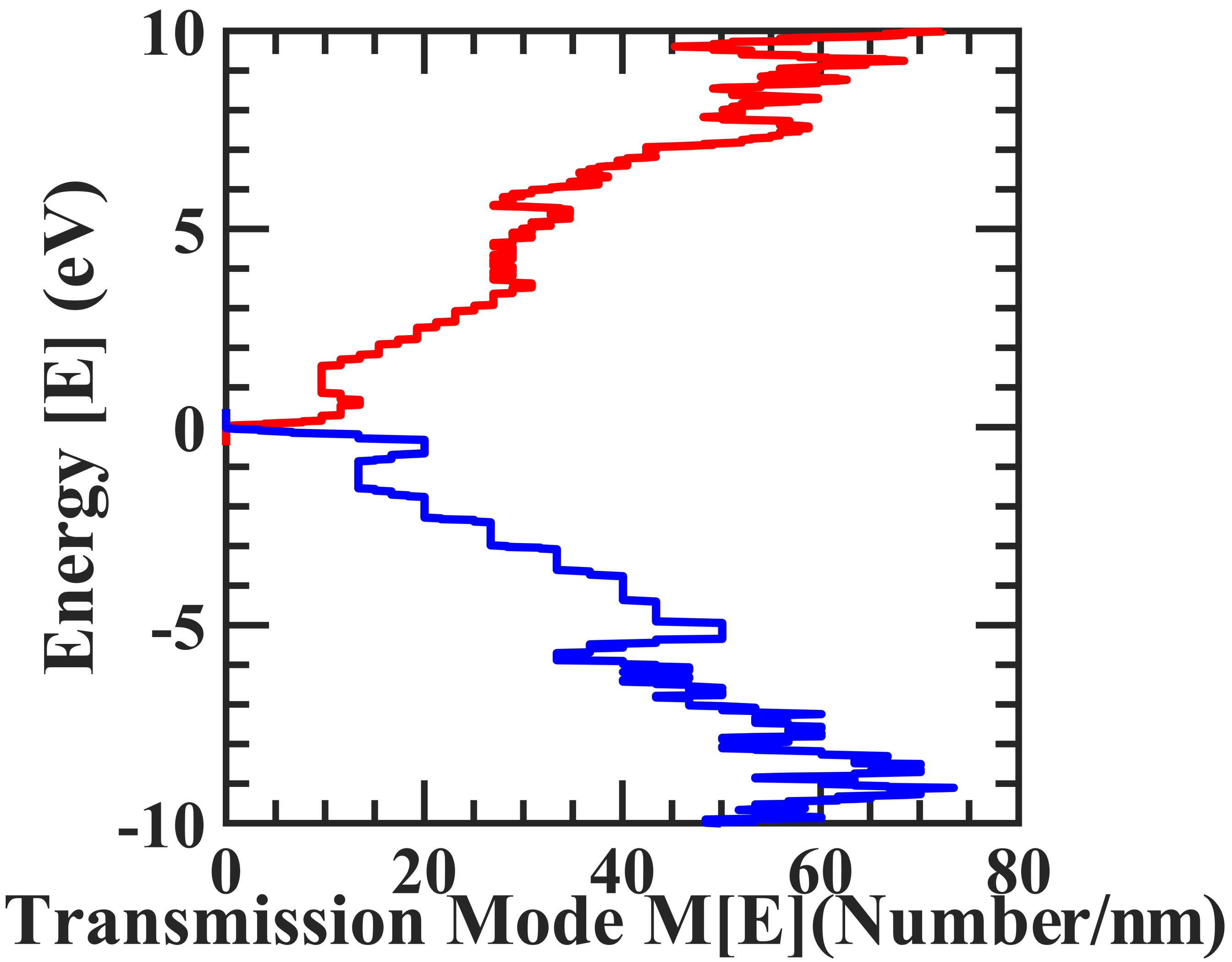

Figure 63. Density of Mode M(E) corrugate-10pm

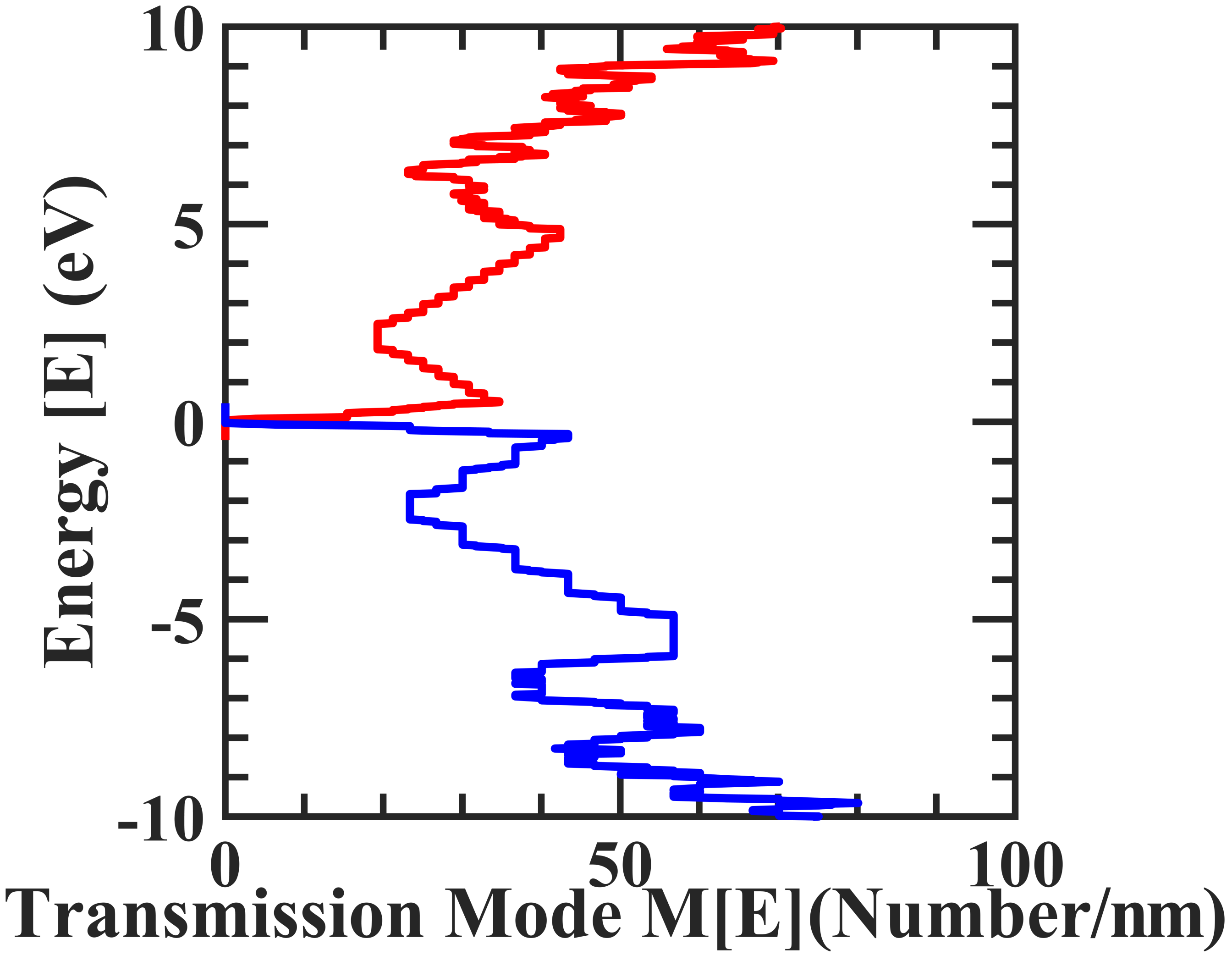

Figure 64. Density of Mode M(E) corrugate-15pm

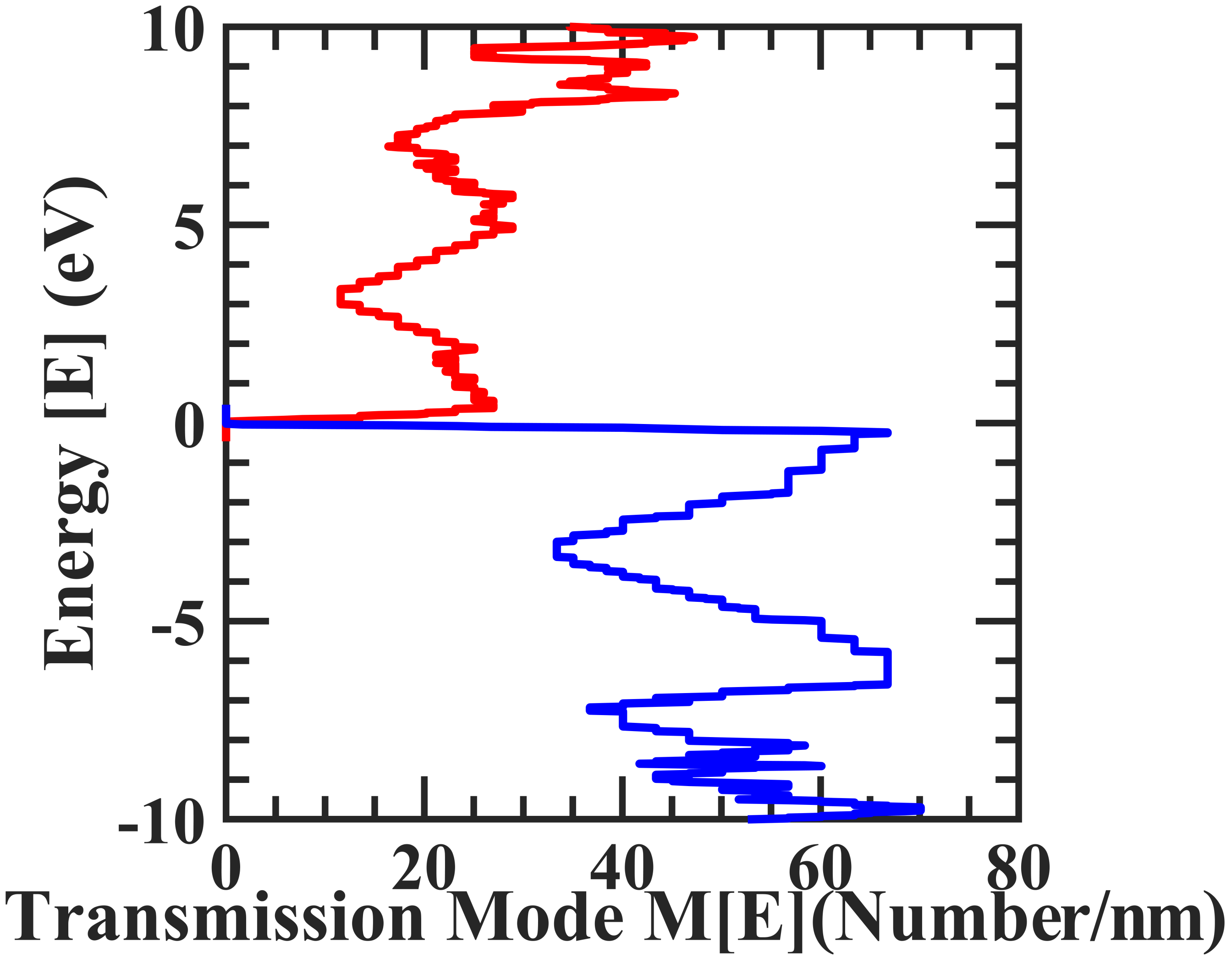

Figure 65. Density of Mode M(E) corrugate-20pm

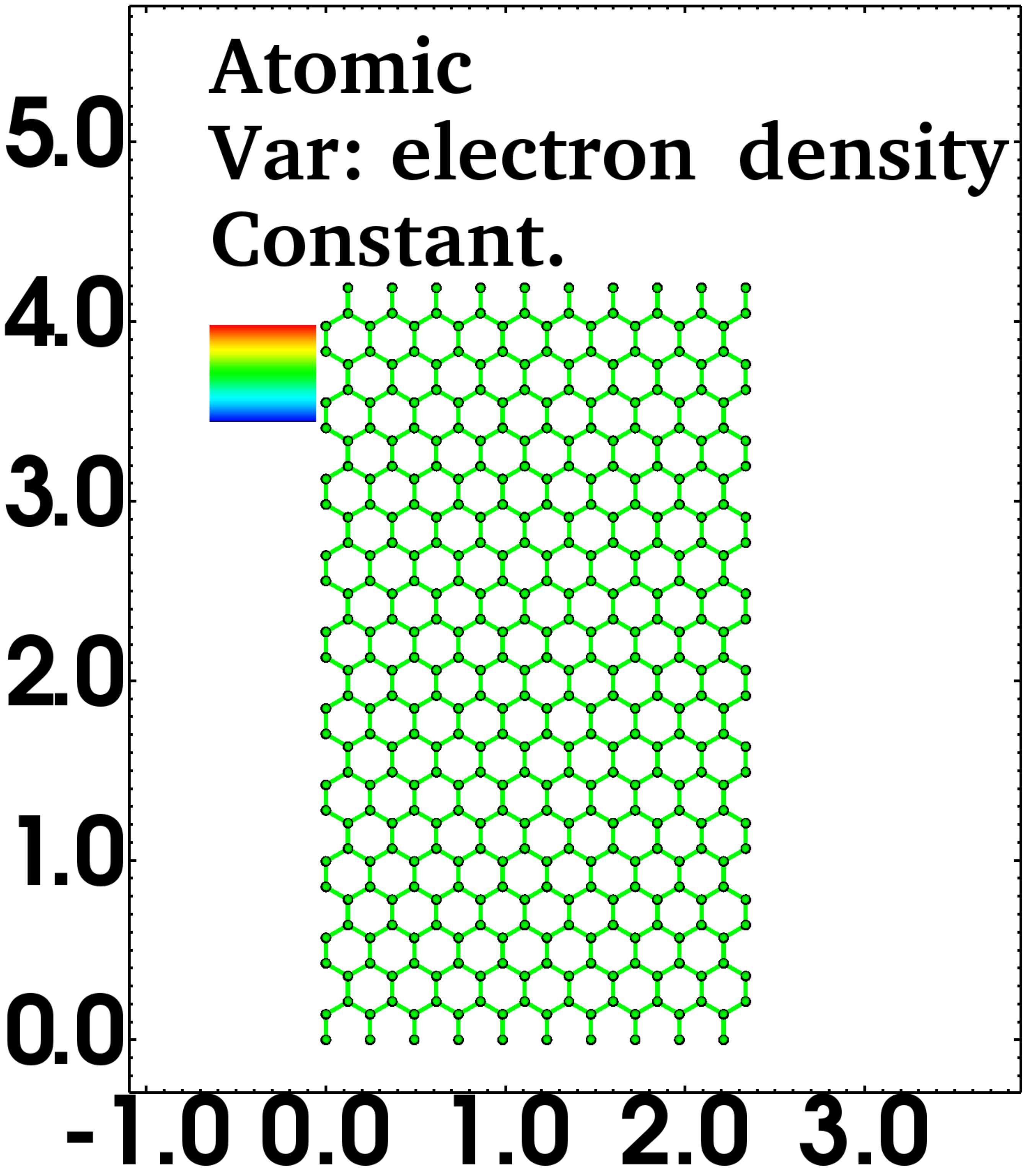

Figure 66. Electron density flat

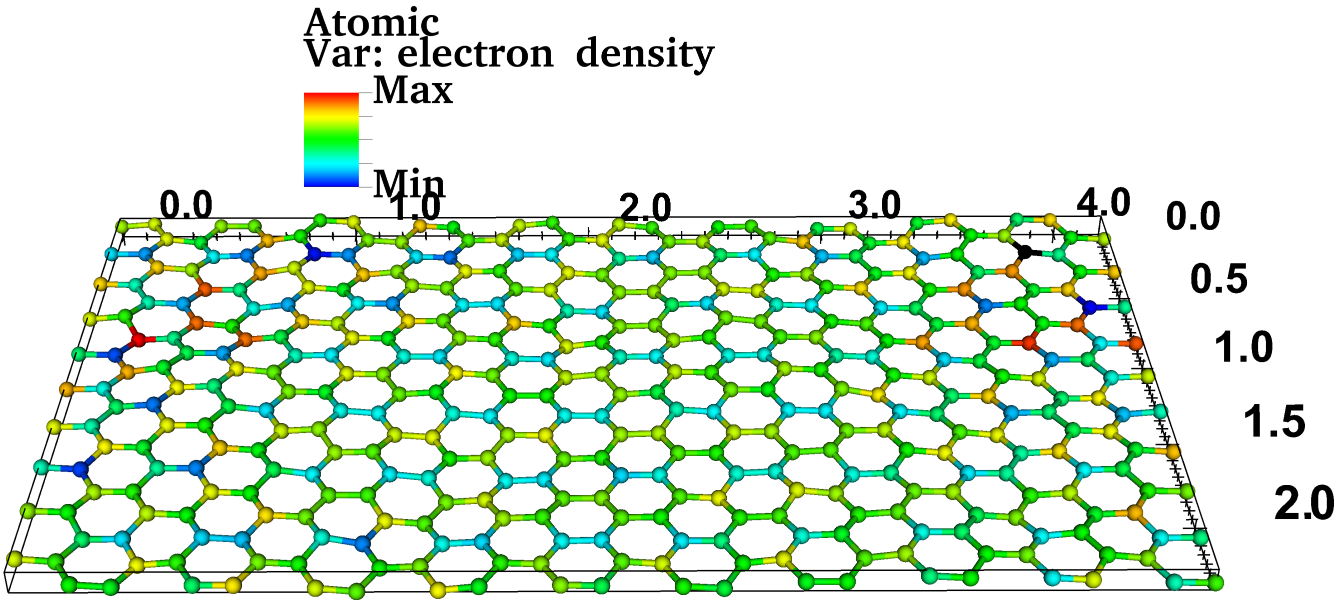

Figure 67. Electron density corrugate-5pm

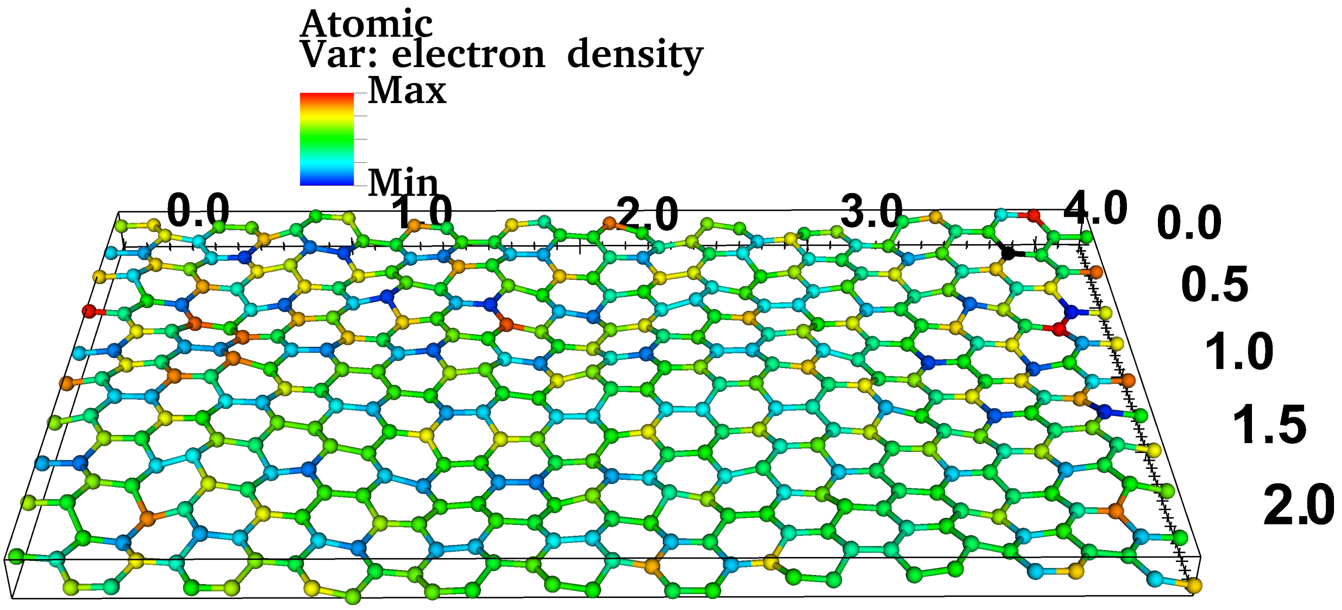

Figure 68. Electron density corrugate-10pm

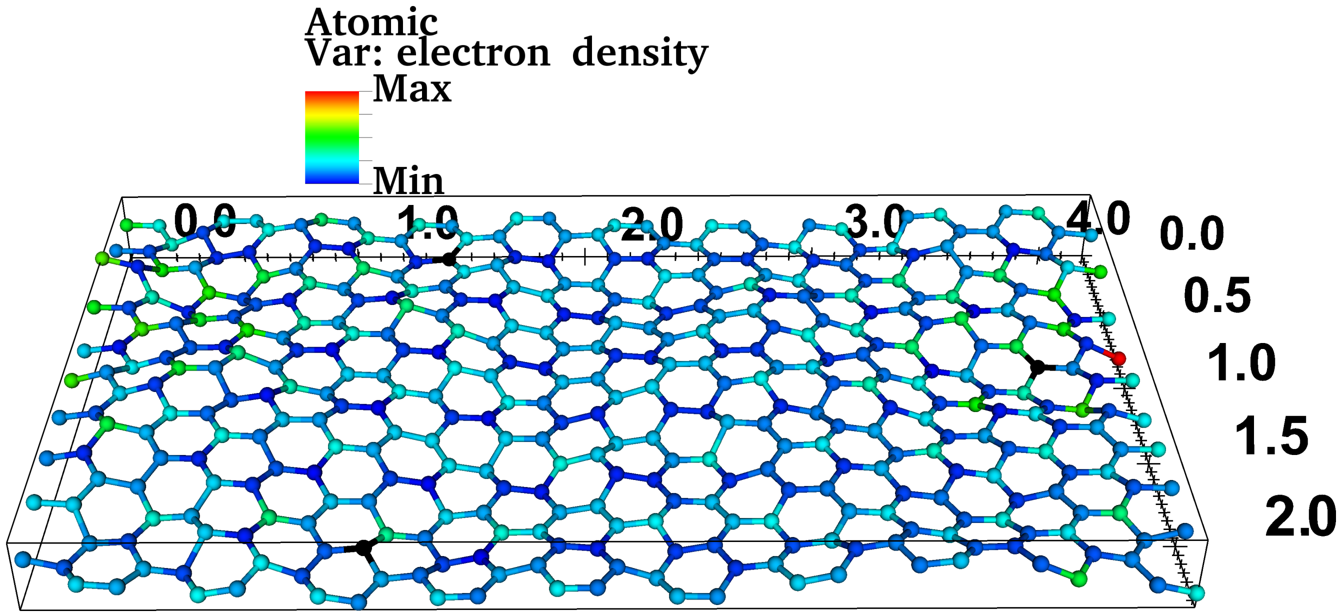

Figure 69. Electron density corrugate-15pm

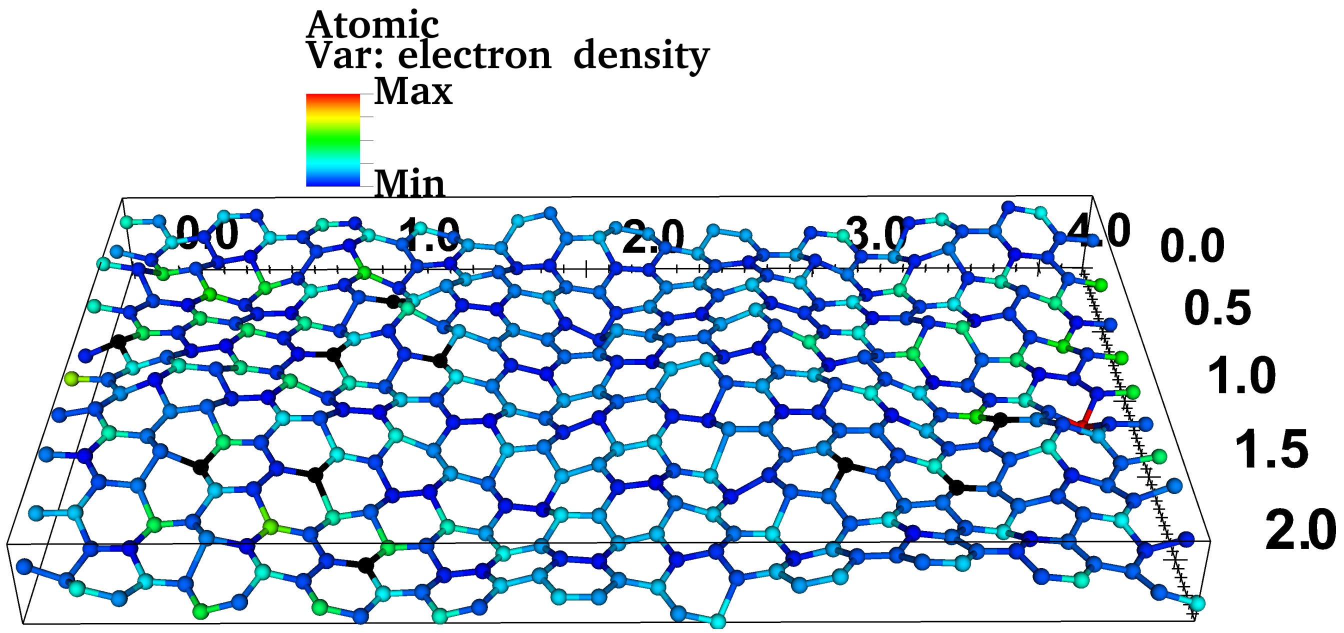

Figure 70. Electron density corrugate-20pm

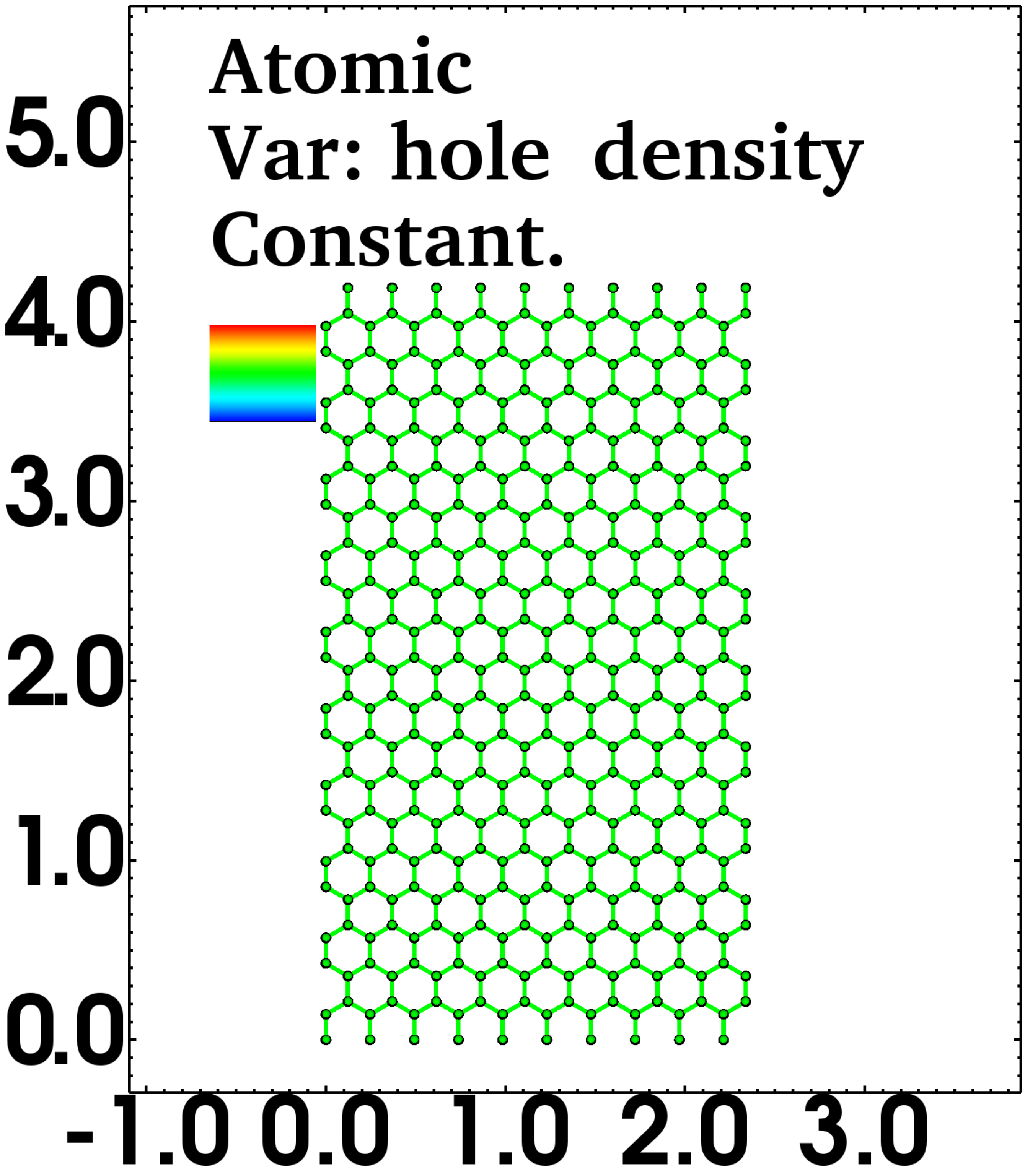

Figure 71. Hole density flat

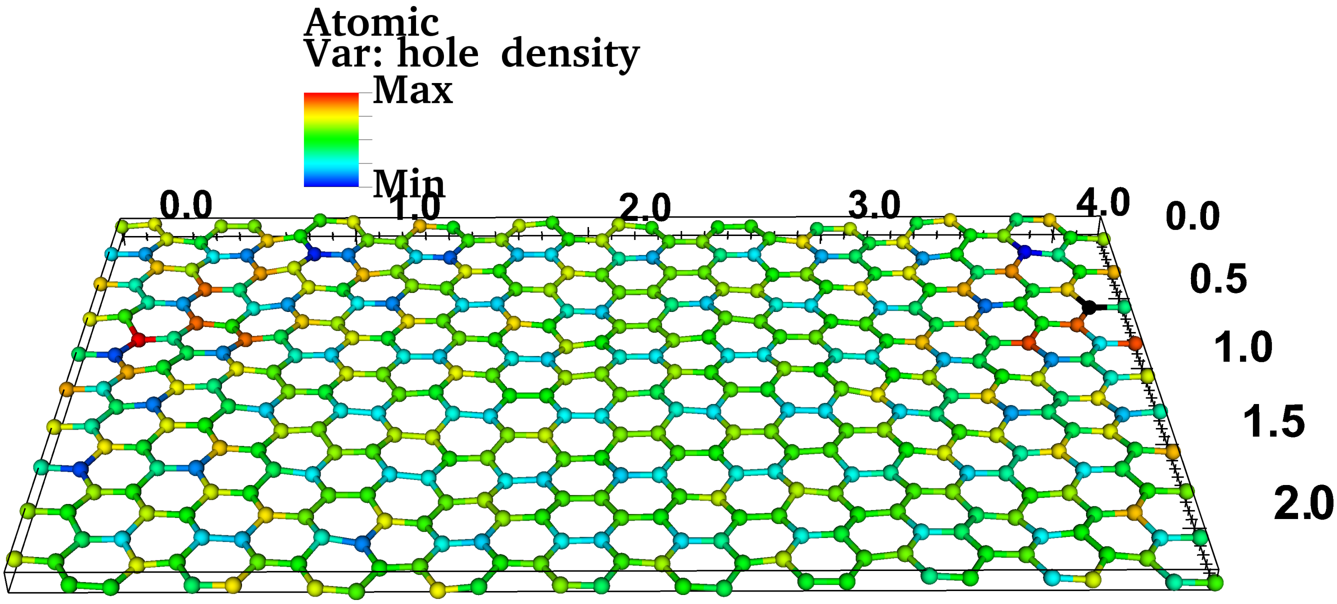

Figure 72. Hole density corrugate-5pm

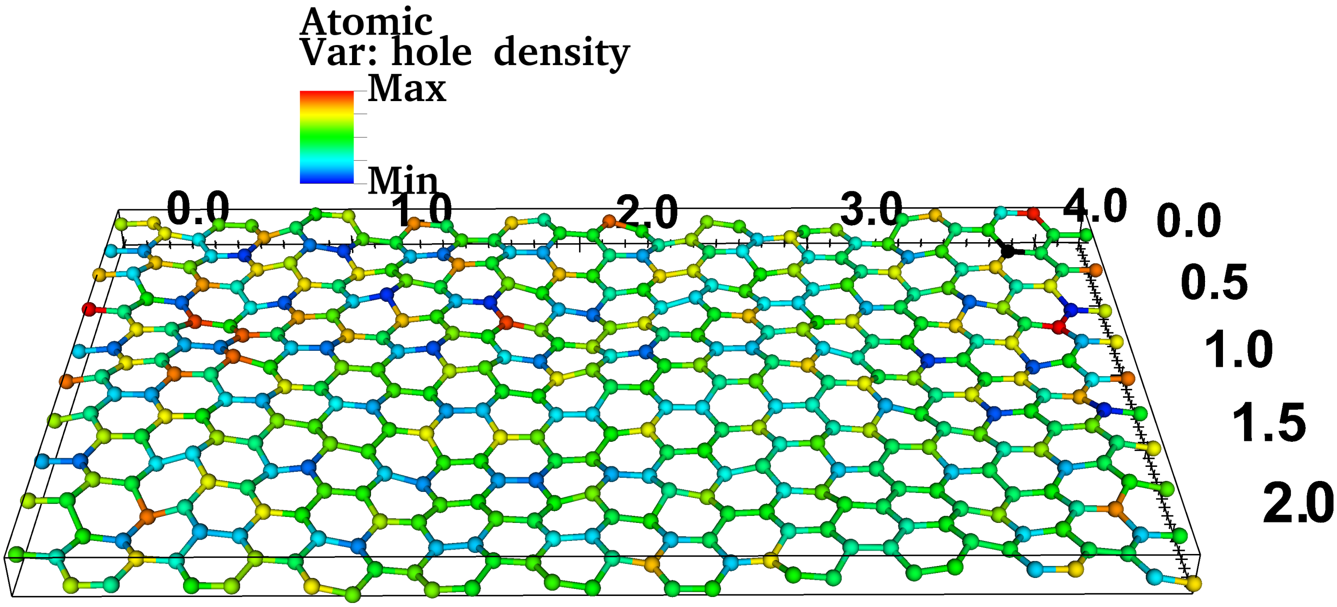

Figure 73. Hole density corrugate-10pm

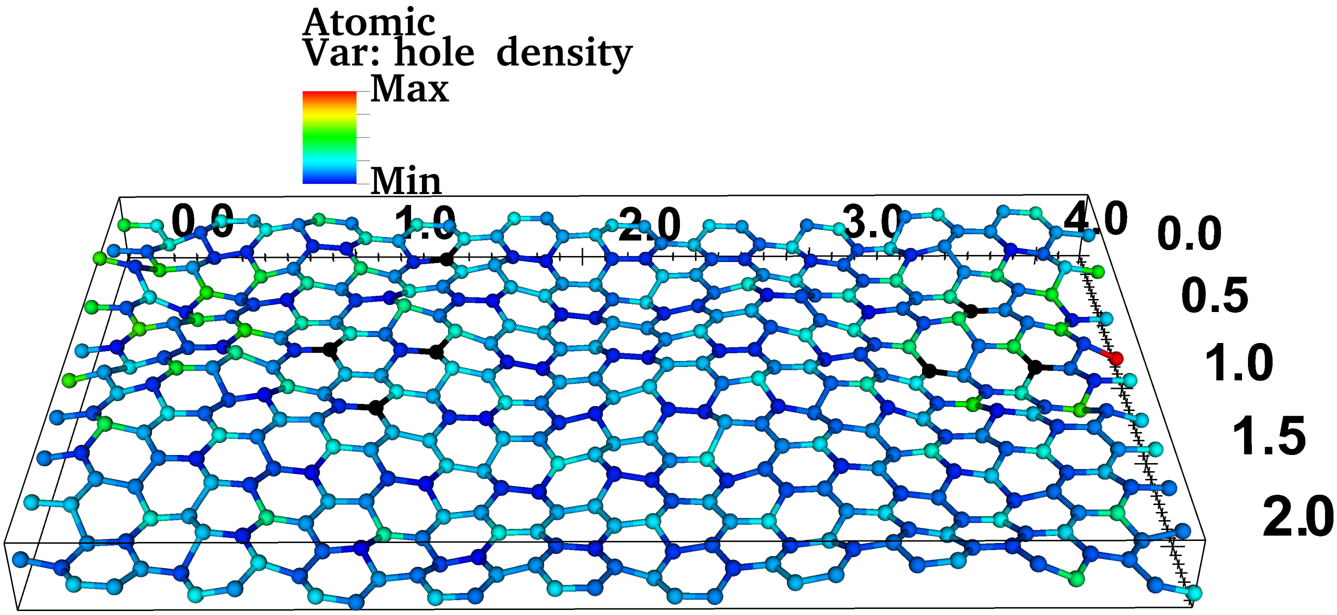

Figure 74. Hole density corrugate-15pm

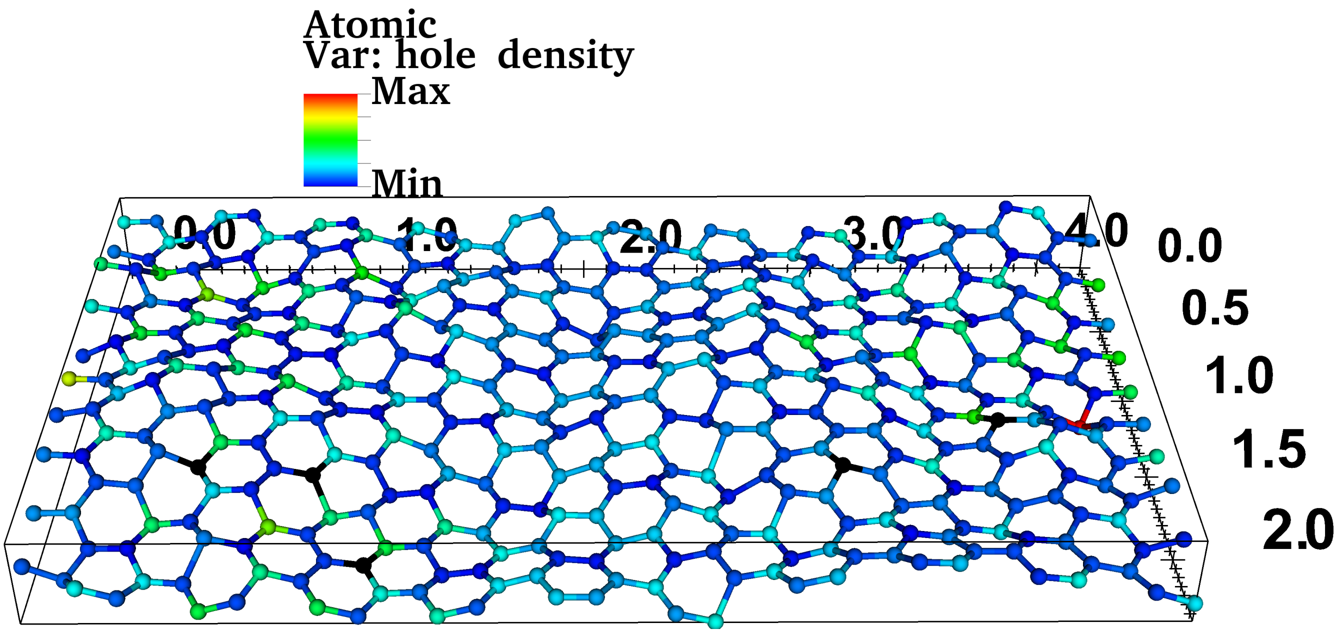

Figure 75. Hole density corrugate-20pm

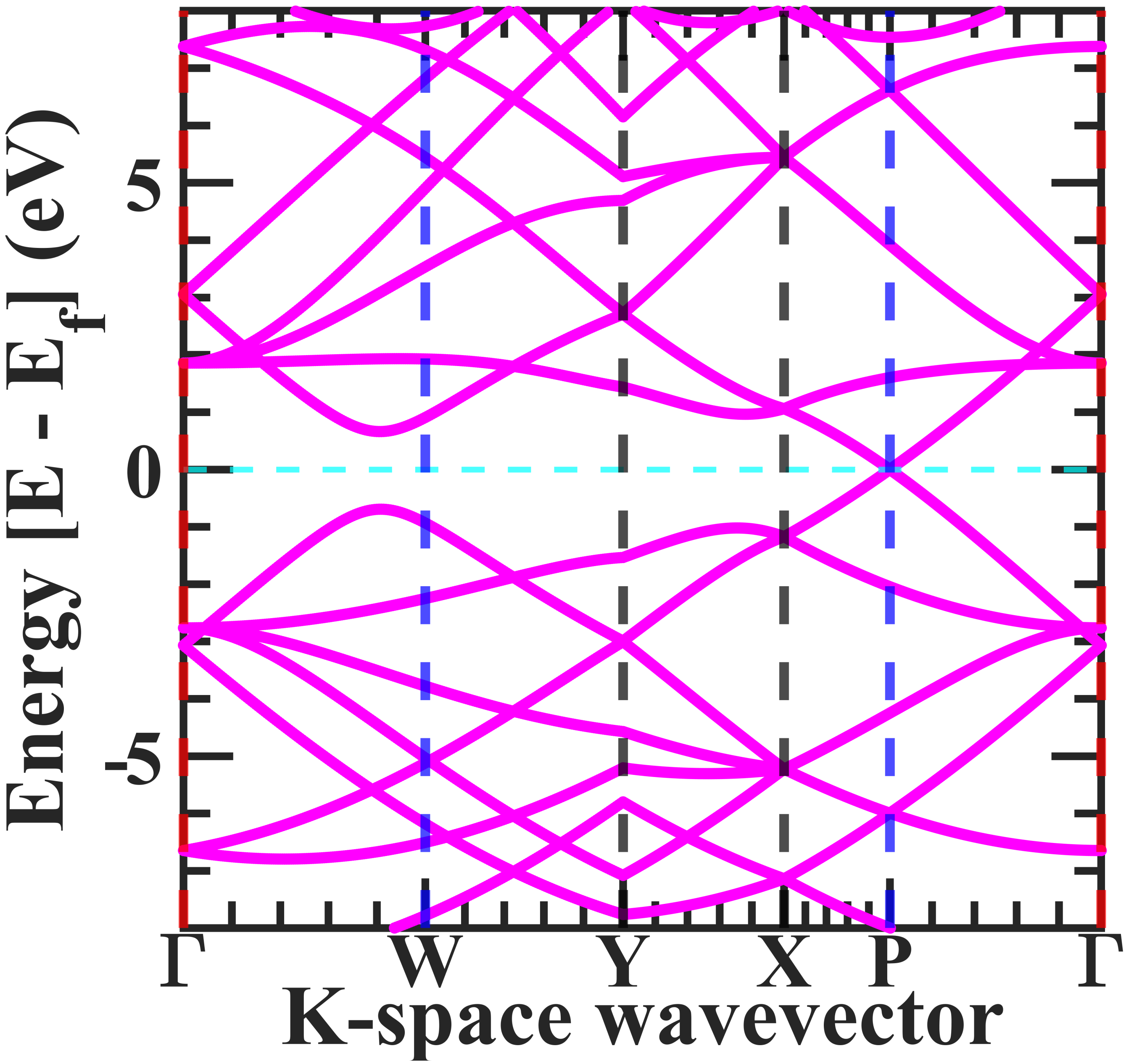

Figure 76. Band structure flat

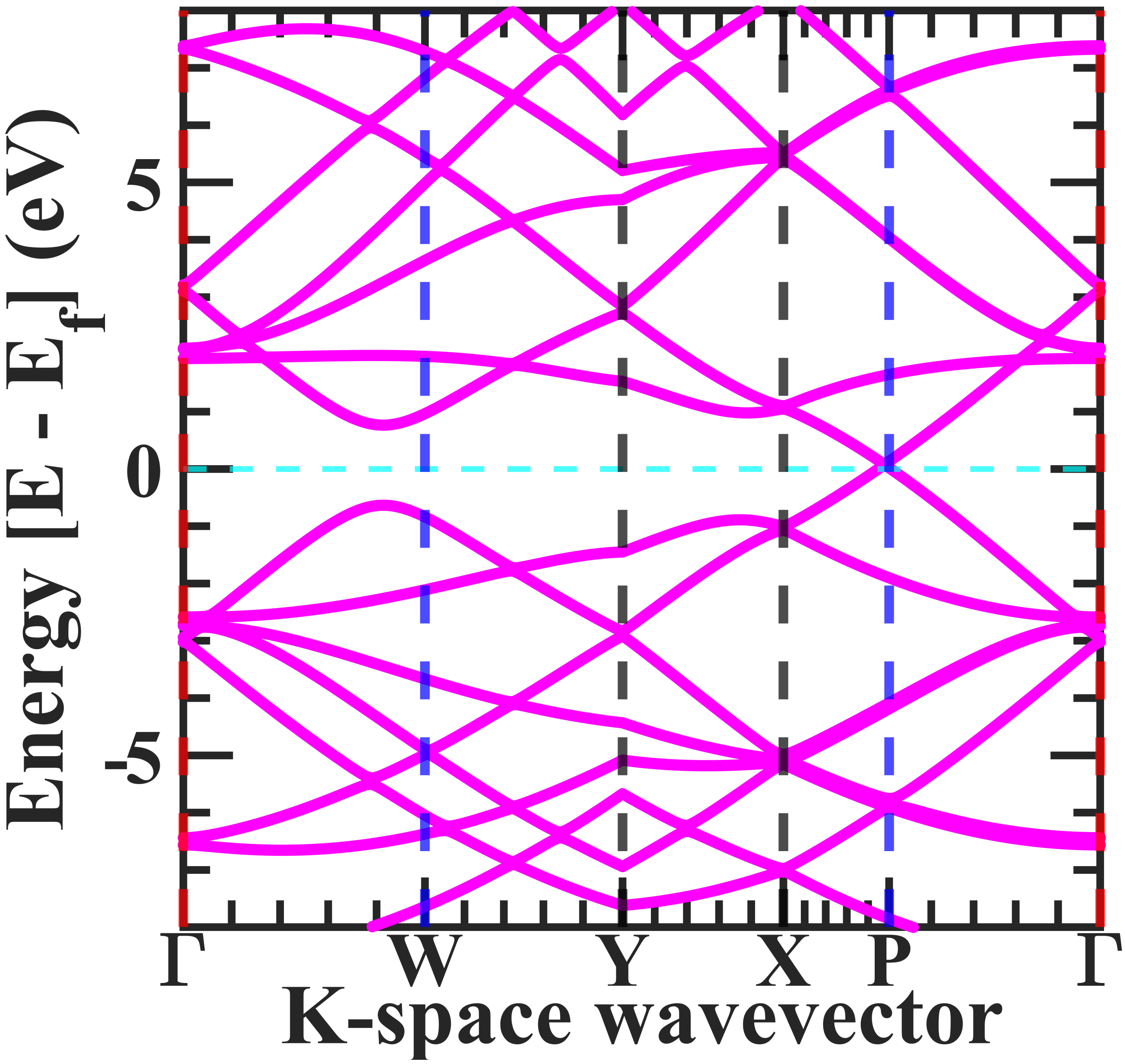

Figure 77. Band structure corrugate-5pm

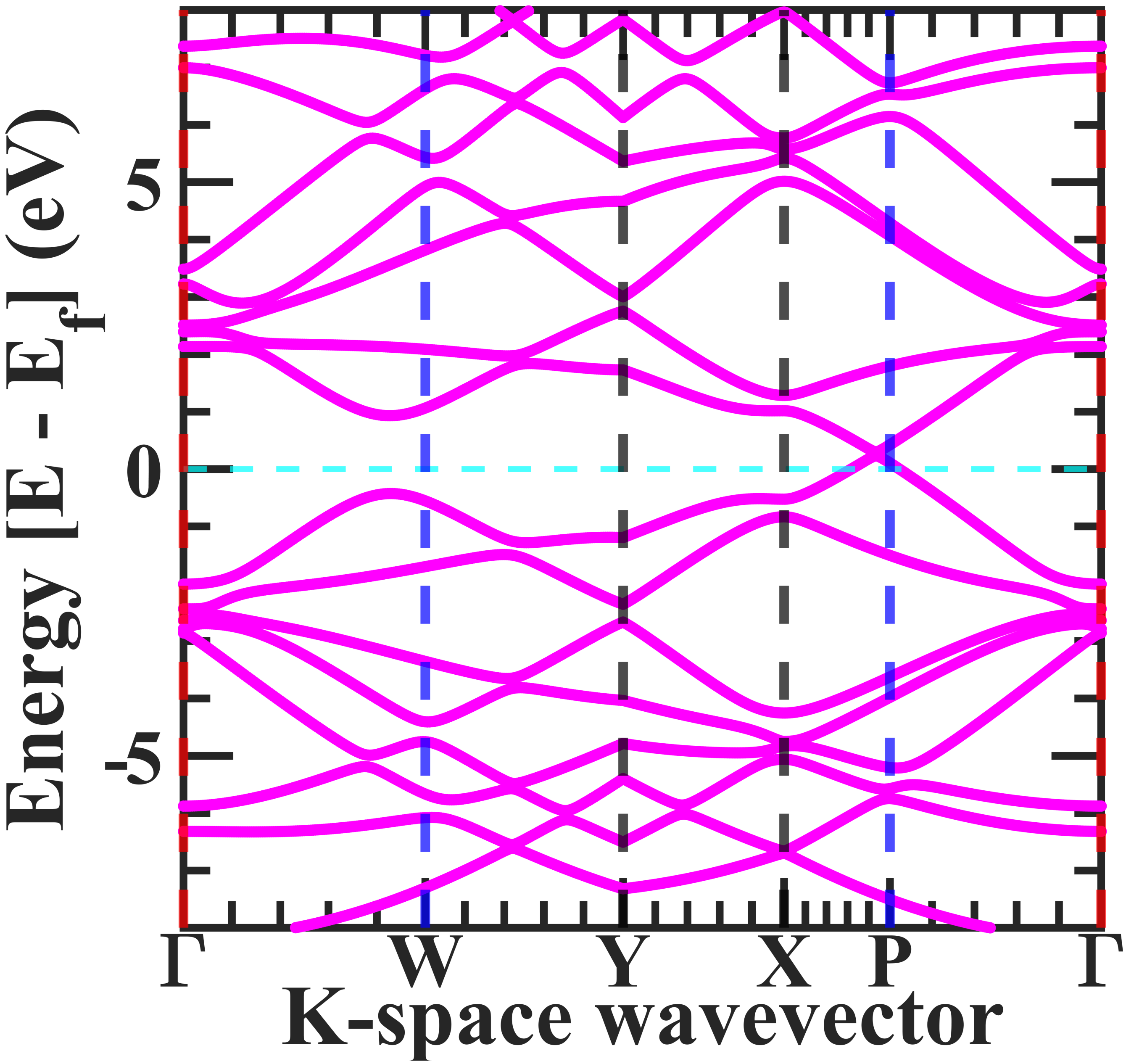

Figure 78. Band structure corrugate-10pm

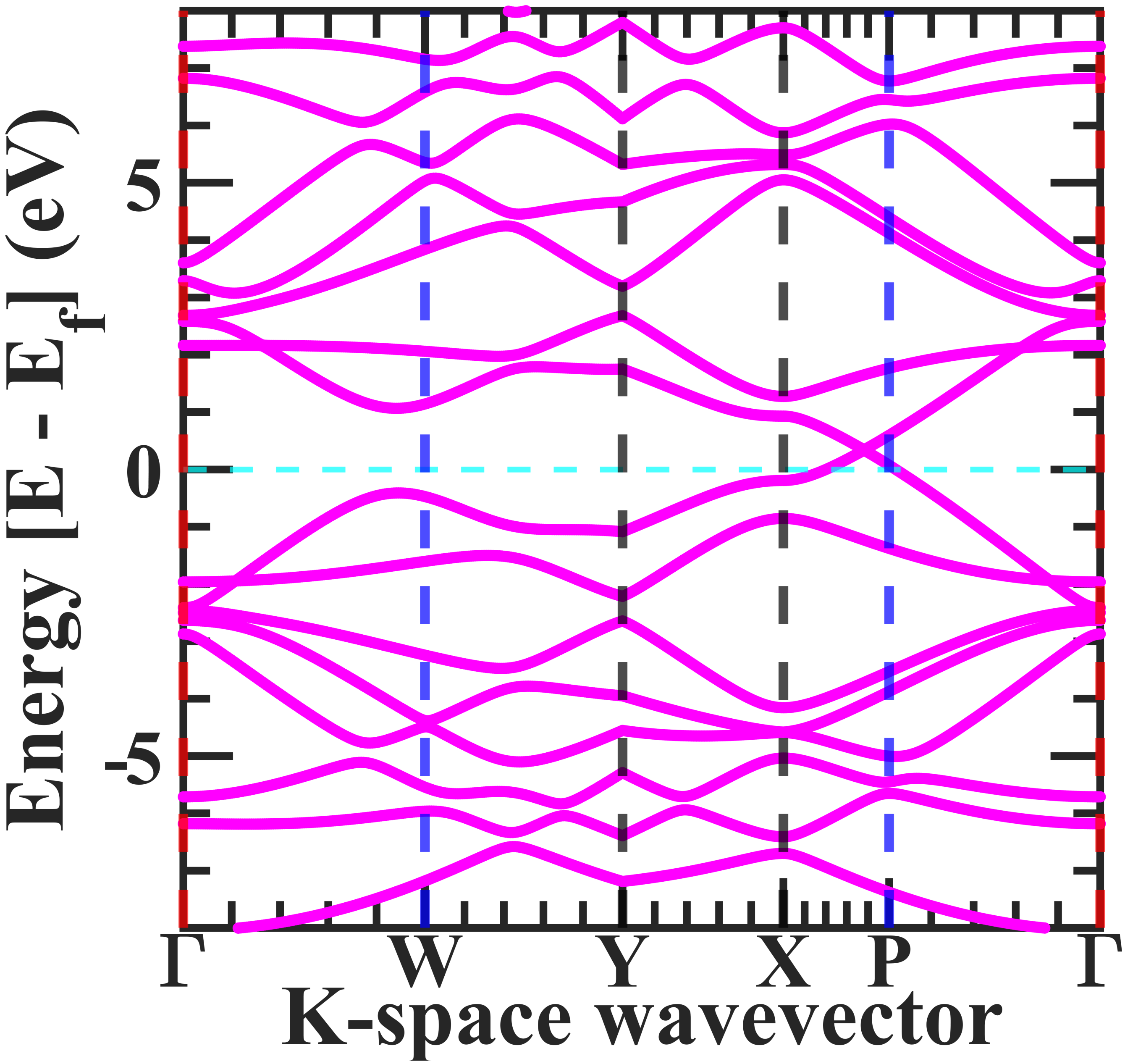

Figure 79. Band structure corrugate-15pm

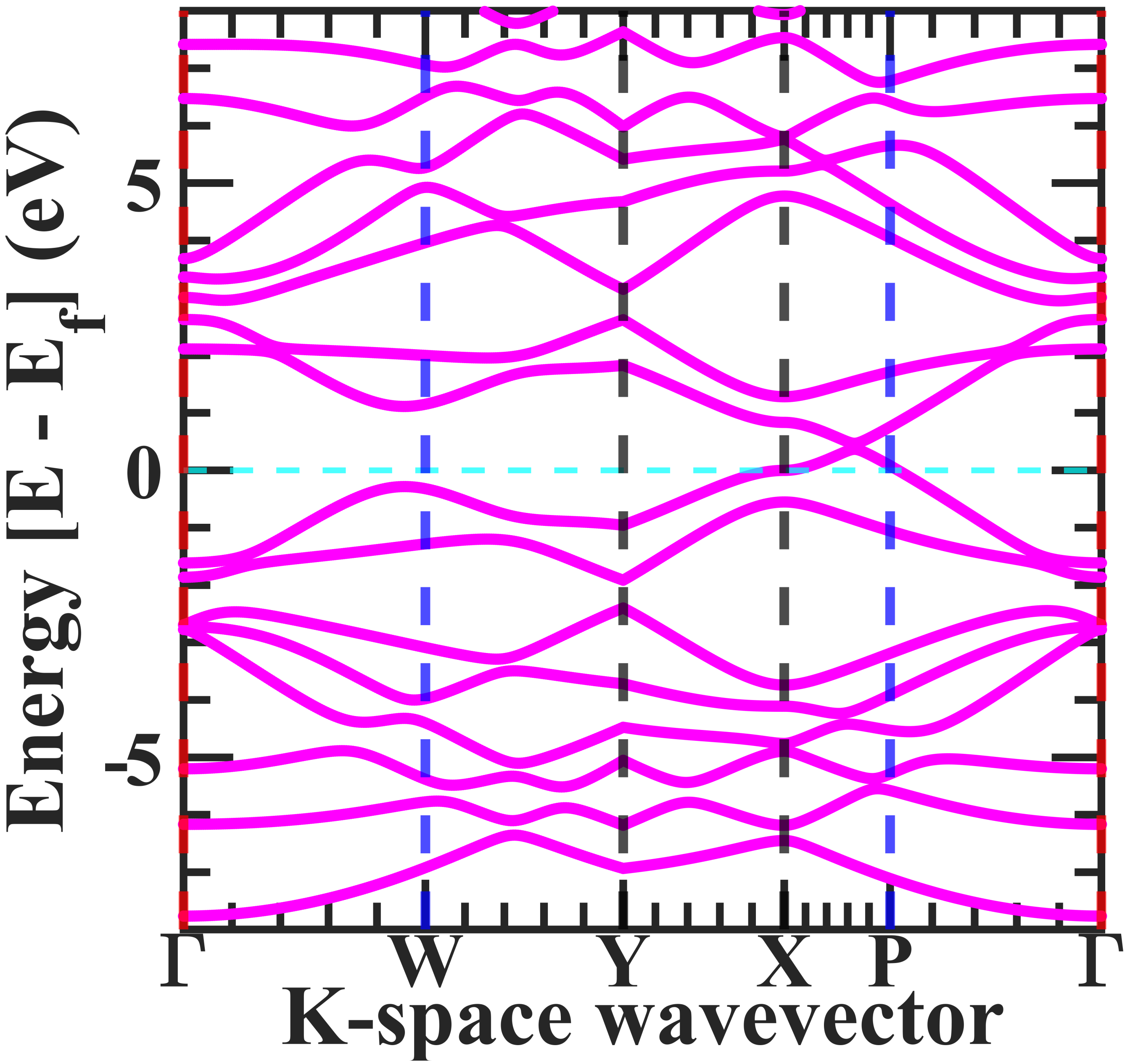

Figure 80. Band structure corrugate-20pm

In-plane Deformation Roughness Effect Effect

10x10 graphene supercell

The fig. 81 to fig. 82 correspond to 10x10 graphene supercell in-plane deformation device structure with roughness varying from 5 pm to 10 pm, mimicking the in-plane deformation profile of h-BN and SiO_2 substrate. Similarly, fig. 83 to fig. 84 represents the corresponding density of state, fig. 85 to fig. 86 represents the electronic density of mode M(E) in the x-y direction, fig. 87 to fig. 88 represents the electronic density, fig. 89 to fig. 90 represents the hole density and fig. 91 to fig. 92 represents the electronic bandstructure of the corresponding in-plane deformation device structure. In all, the in-plane deformation structure correlation is kept constant at 10nm length. In the simulated device, the primitive unit cell has two atoms per cell, and a total of 200 atoms are simulated by a finite element mesh of 800 point Density of Mode size. The P-D tight-binding model contains three orbitals, namely carbon P_z, and carbon-hydrogen passivated D_{yz}, D_{xz} orbitals. Therefore total degree of free density of Mode in hamiltonian is 600 variable-sized. The K' and M' are high symmetric point that corresponds to the folded reduced BZ-zone of graphene supercell.

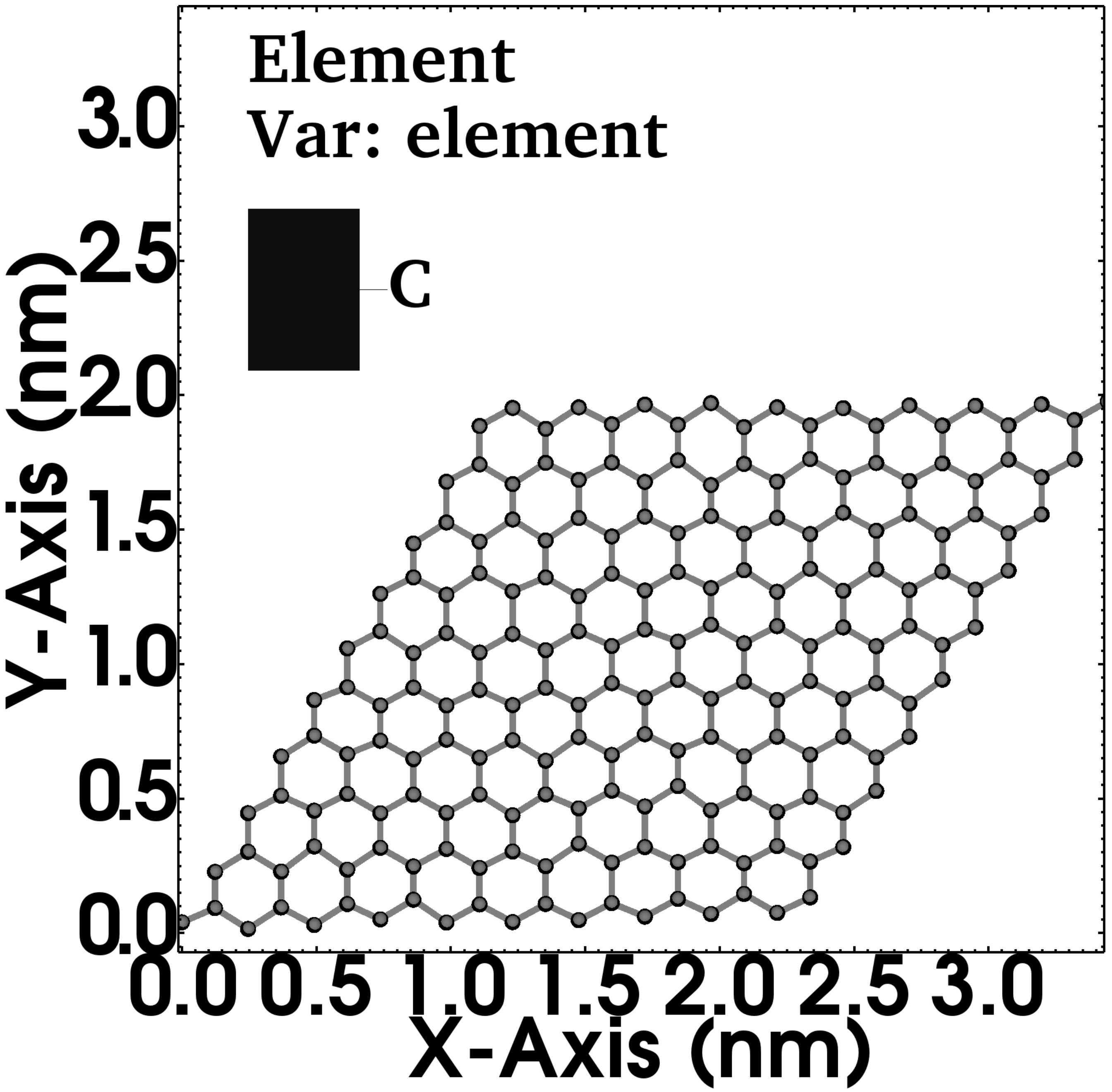

Figure 81. In-plane deformation-5pm

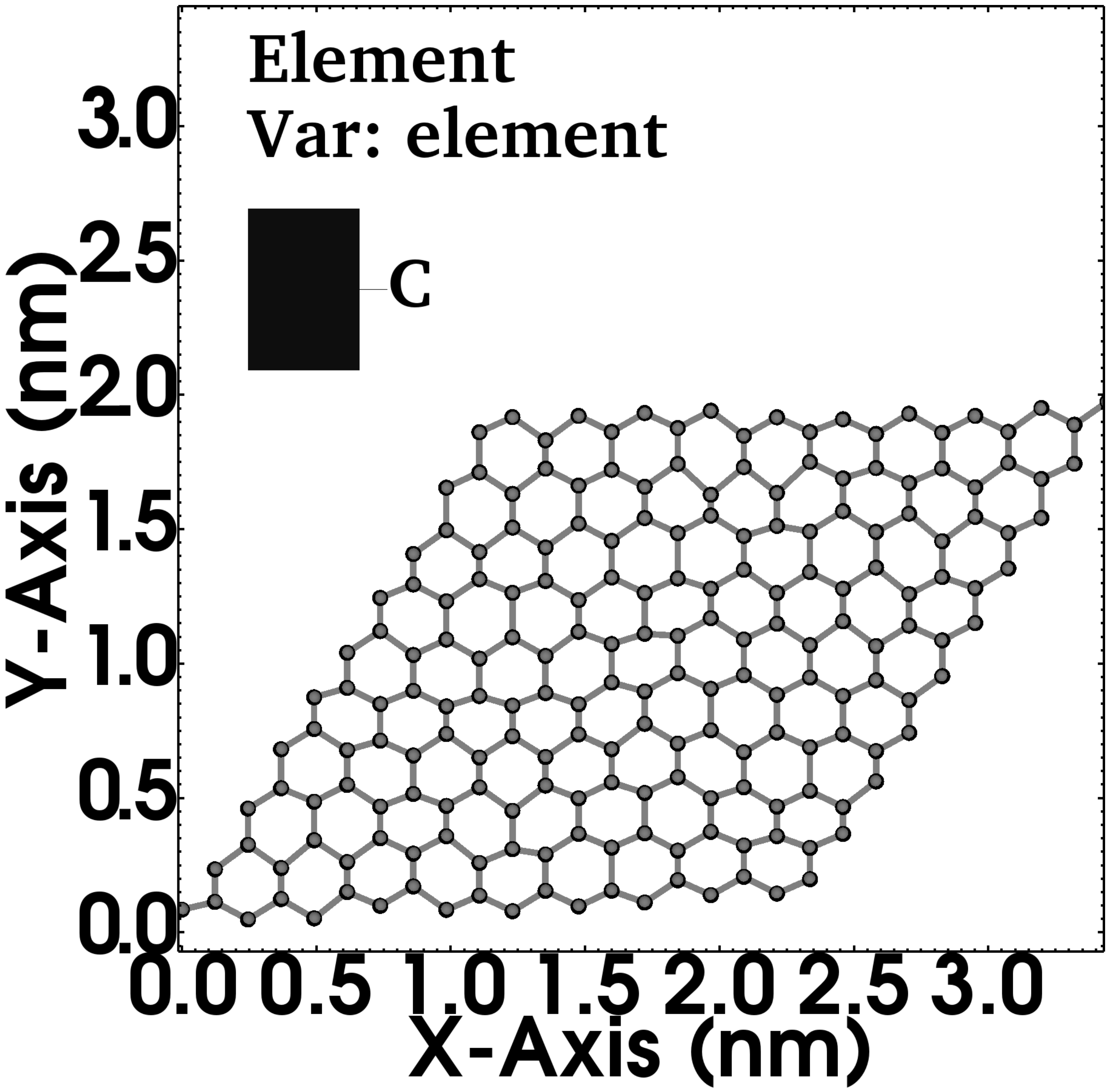

Figure 82. In-plane deformation-10pm

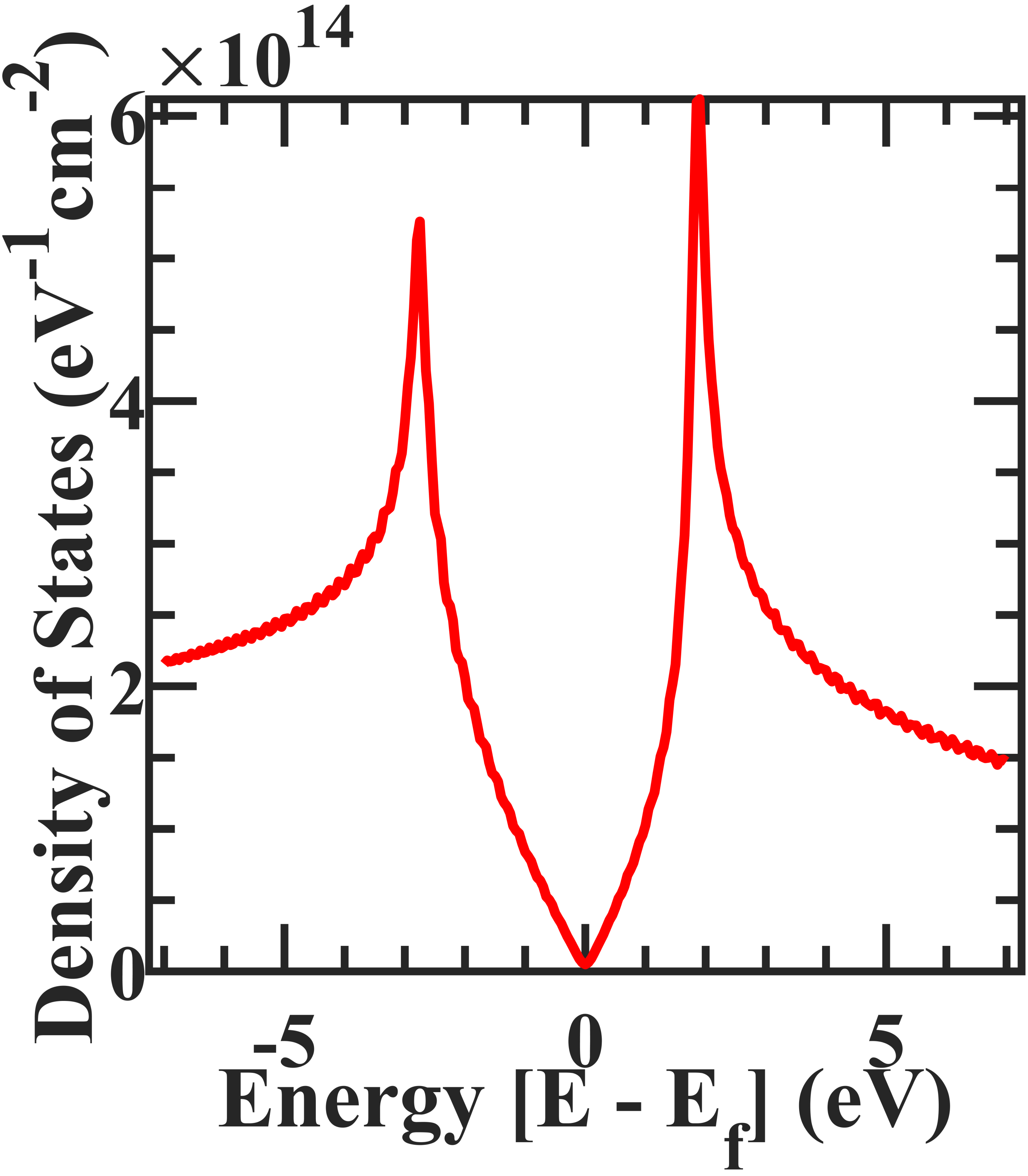

Figure 83. Density of state In-plane deformation-5pm

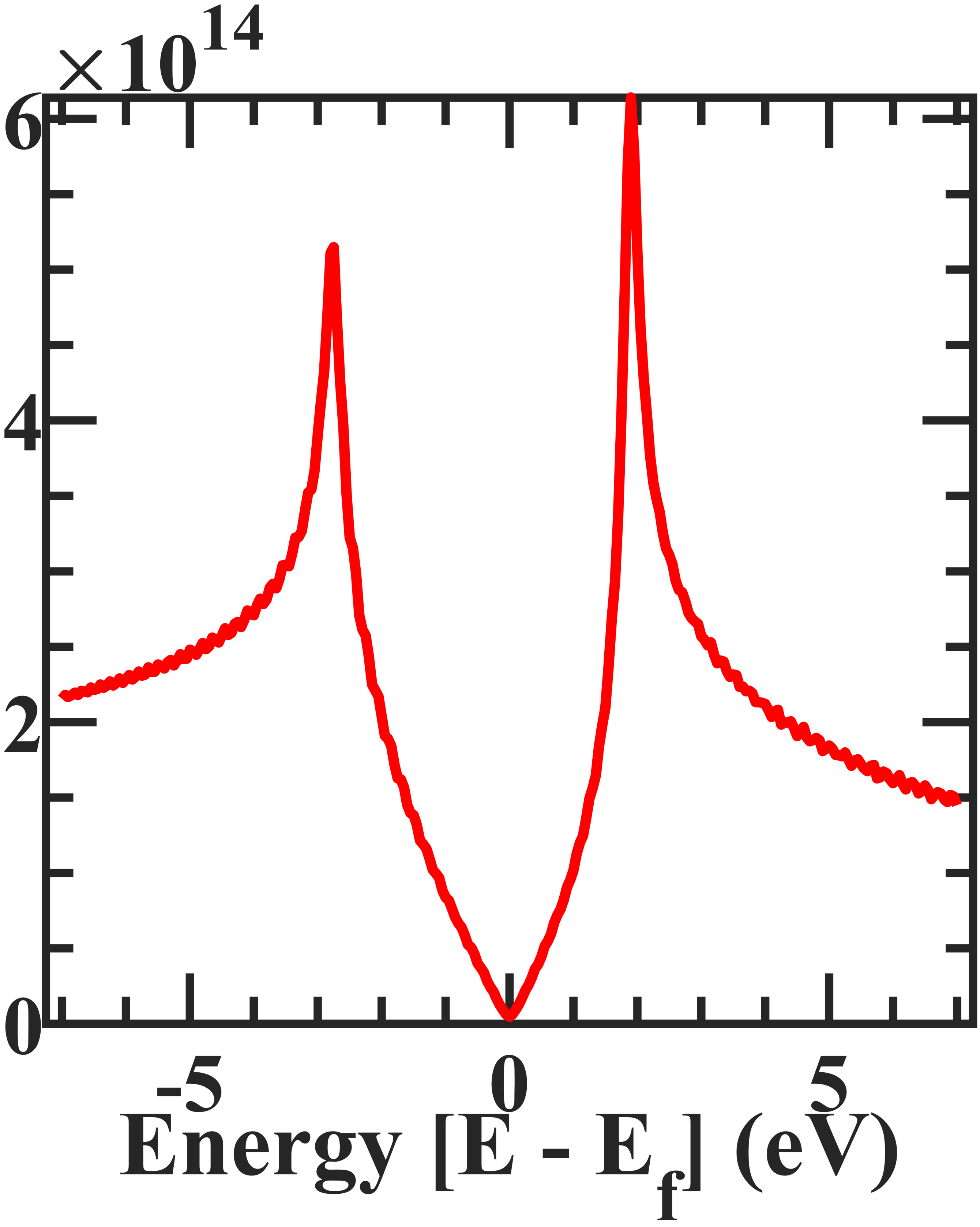

Figure 84. Density of state In-plane deformation-10pm

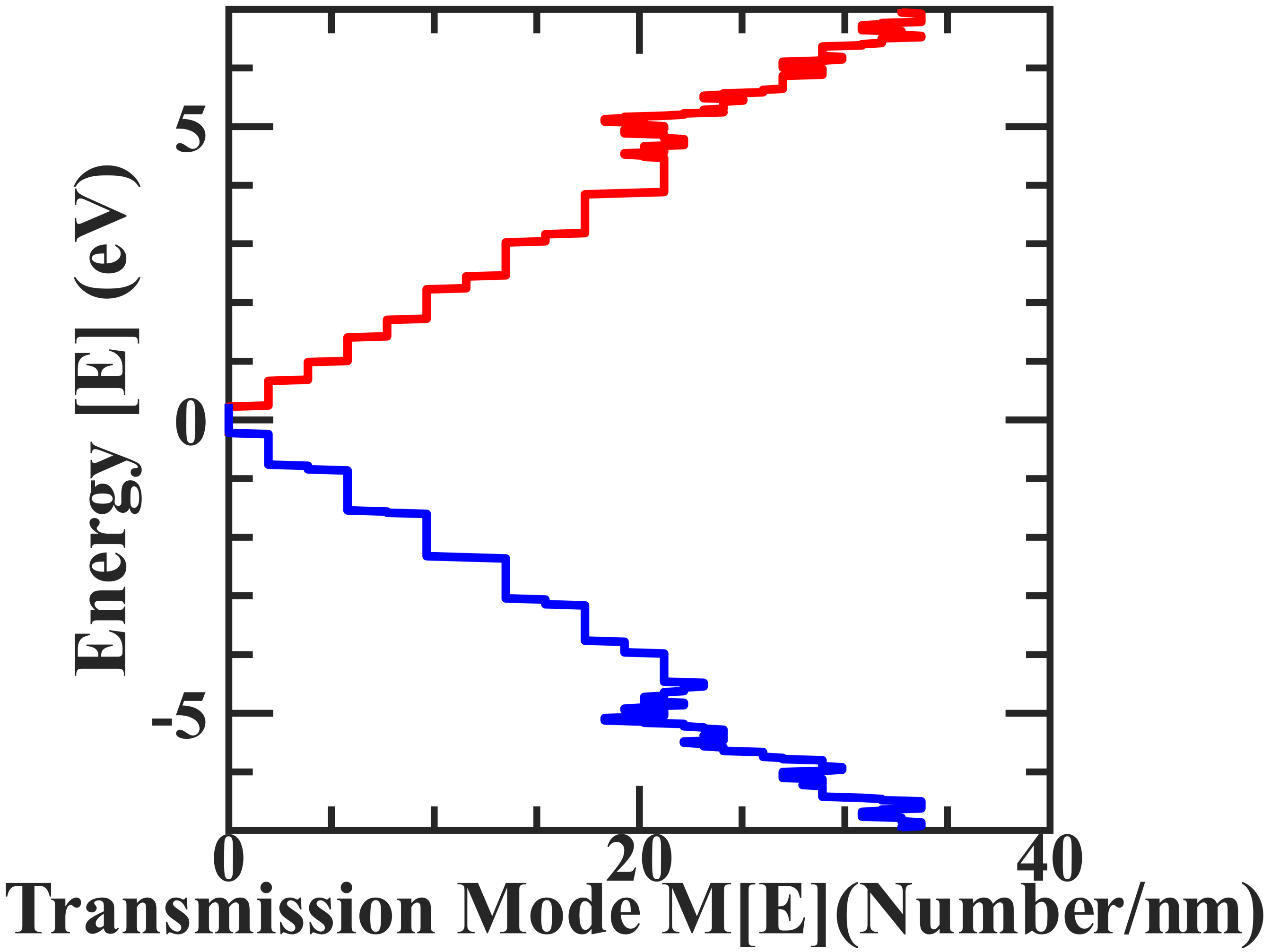

Figure 85. Density of Mode M(E) In-plane deformation-5pm

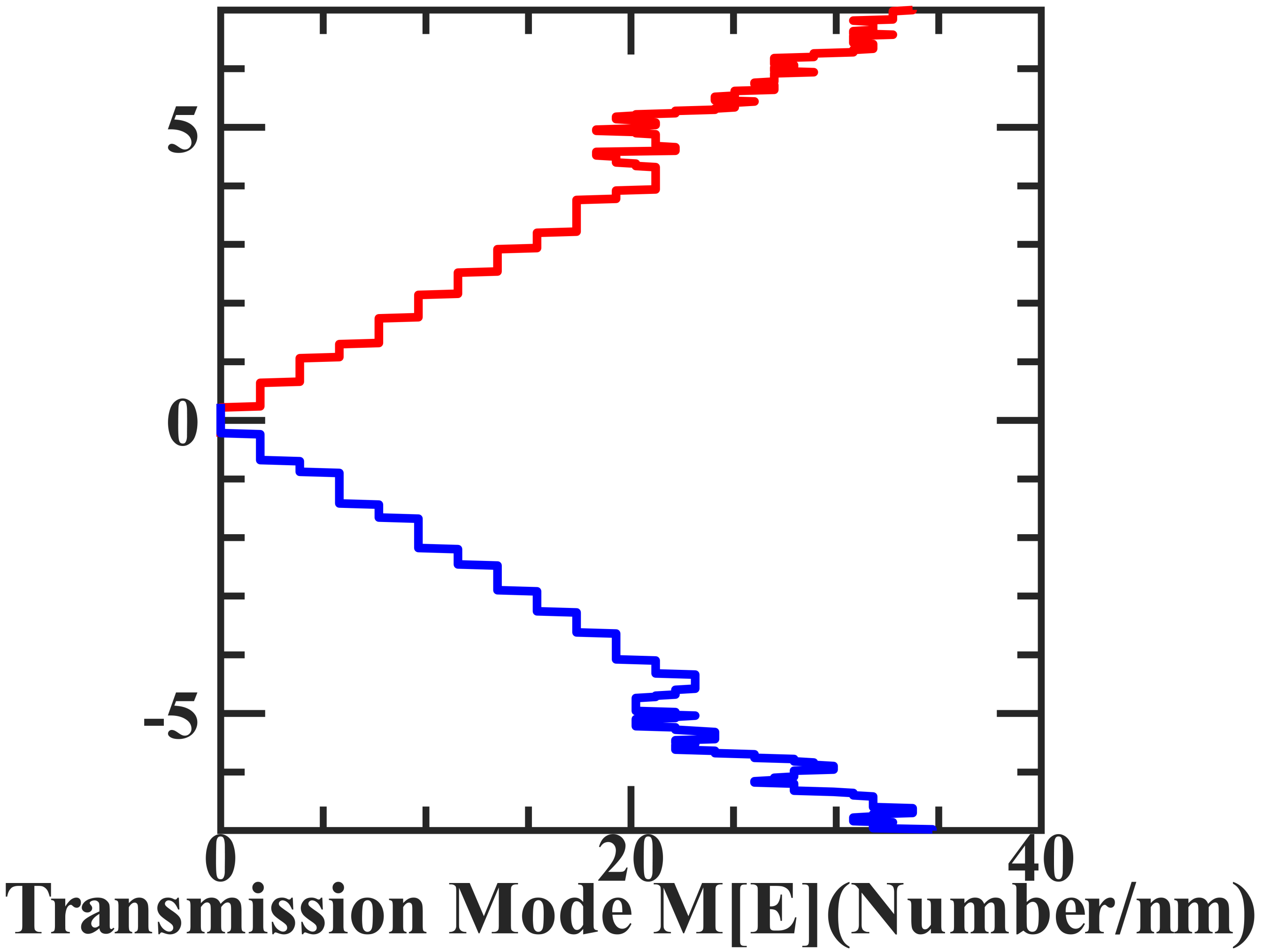

Figure 86. Density of Mode M(E) In-plane deformation-10pm

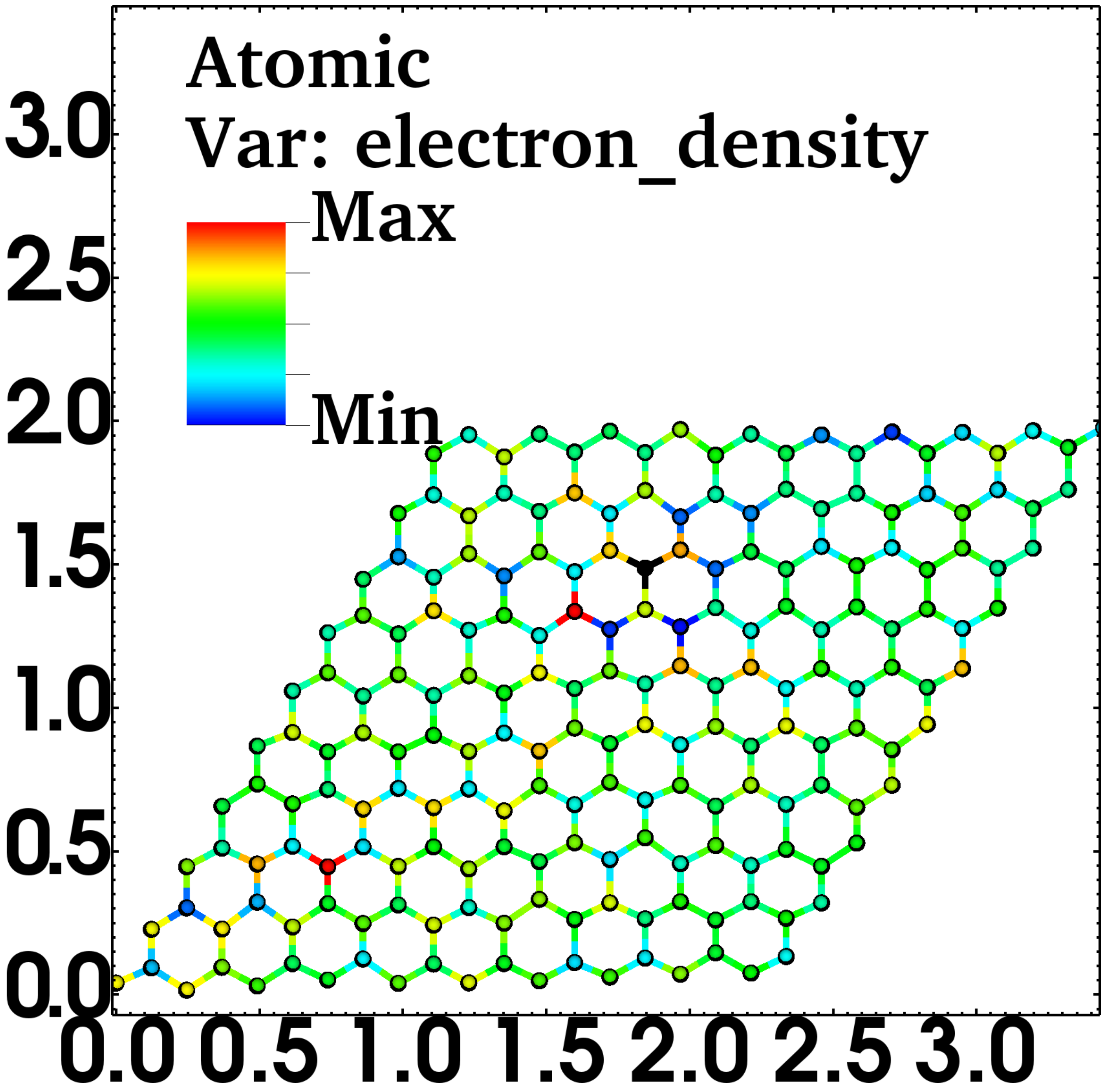

Figure 87. Electron density In-plane deformation-5pm

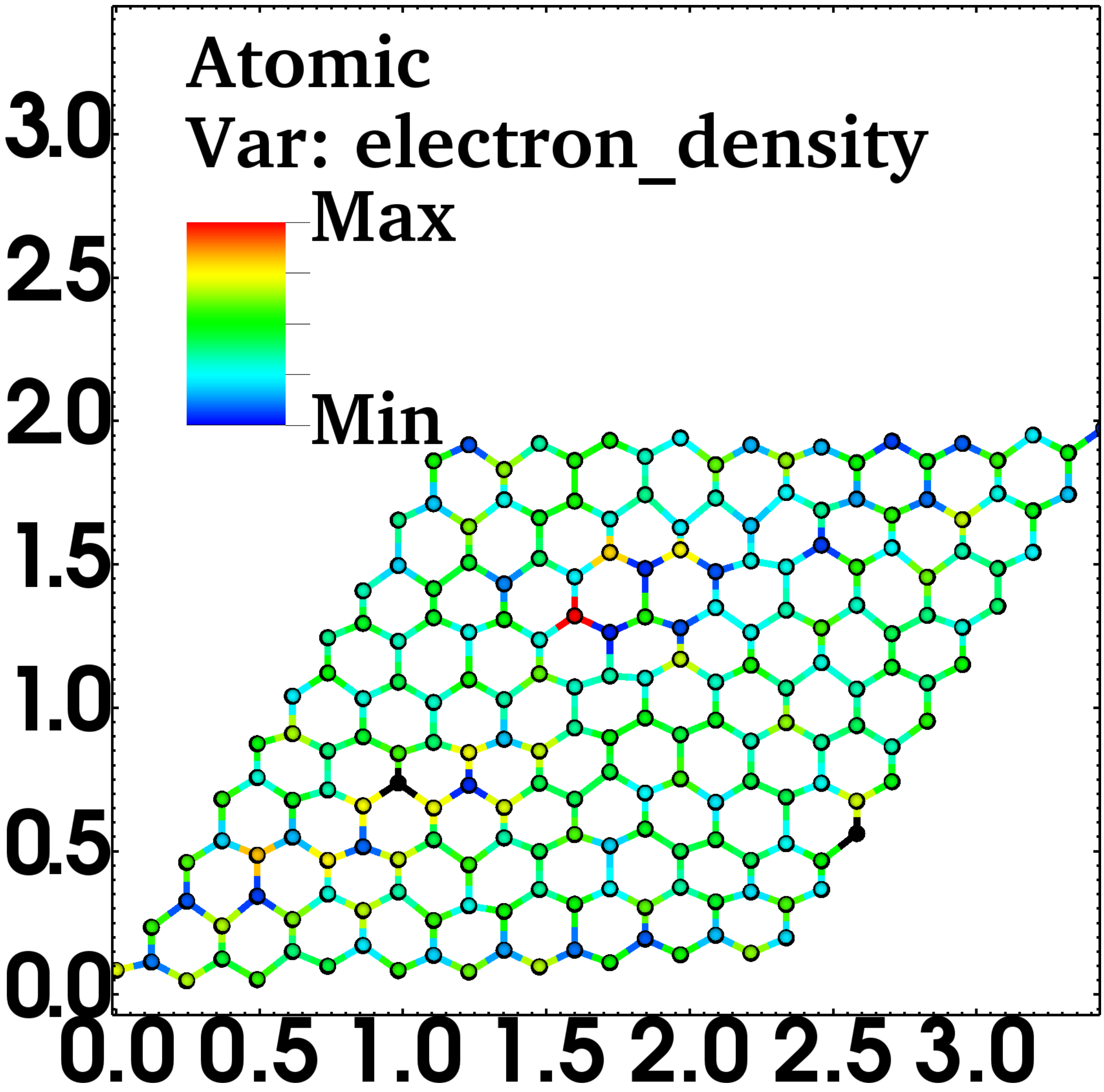

Figure 88. Electron density In-plane deformation-10pm

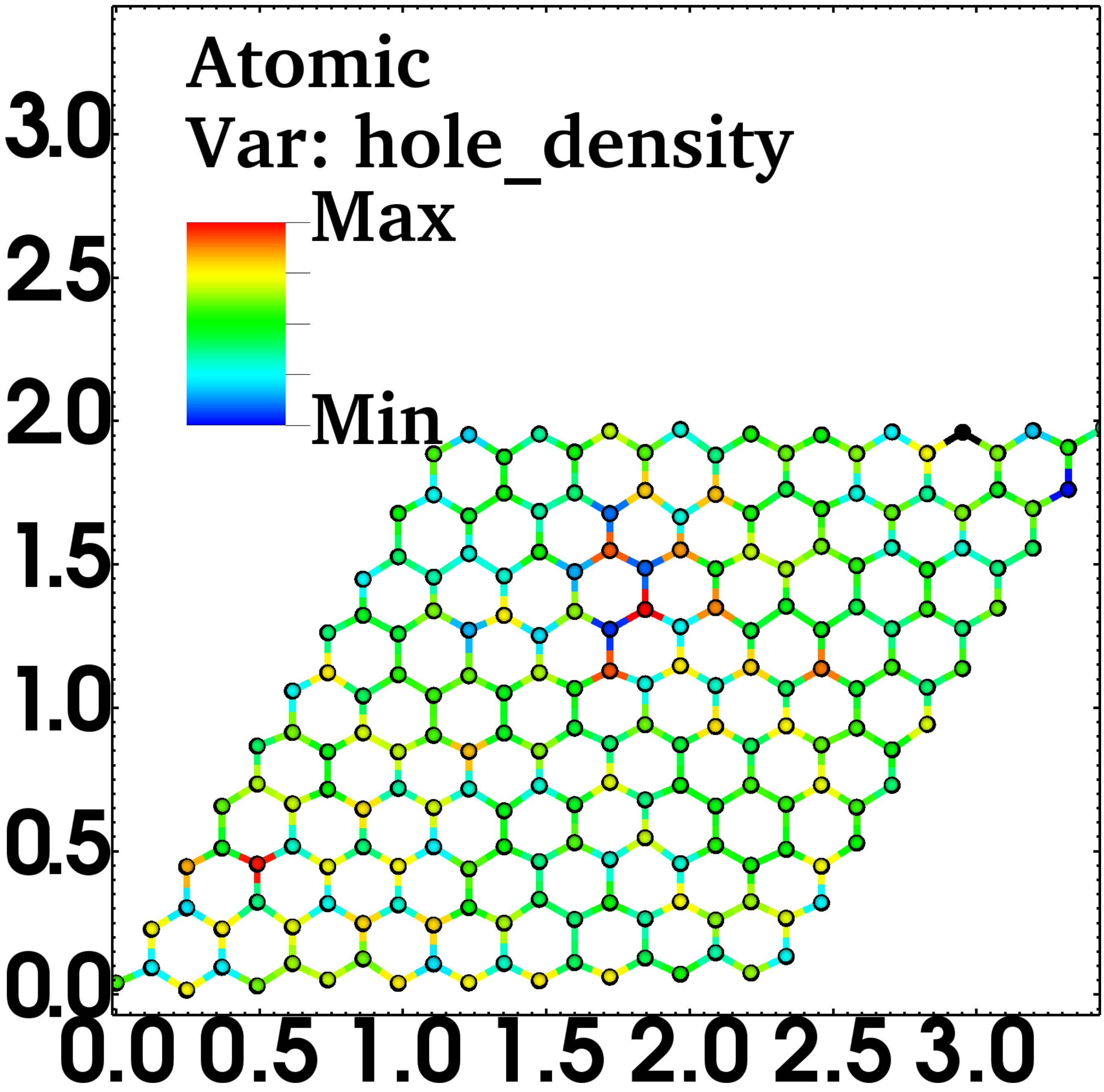

Figure 89. Hole density In-plane deformation-5pm

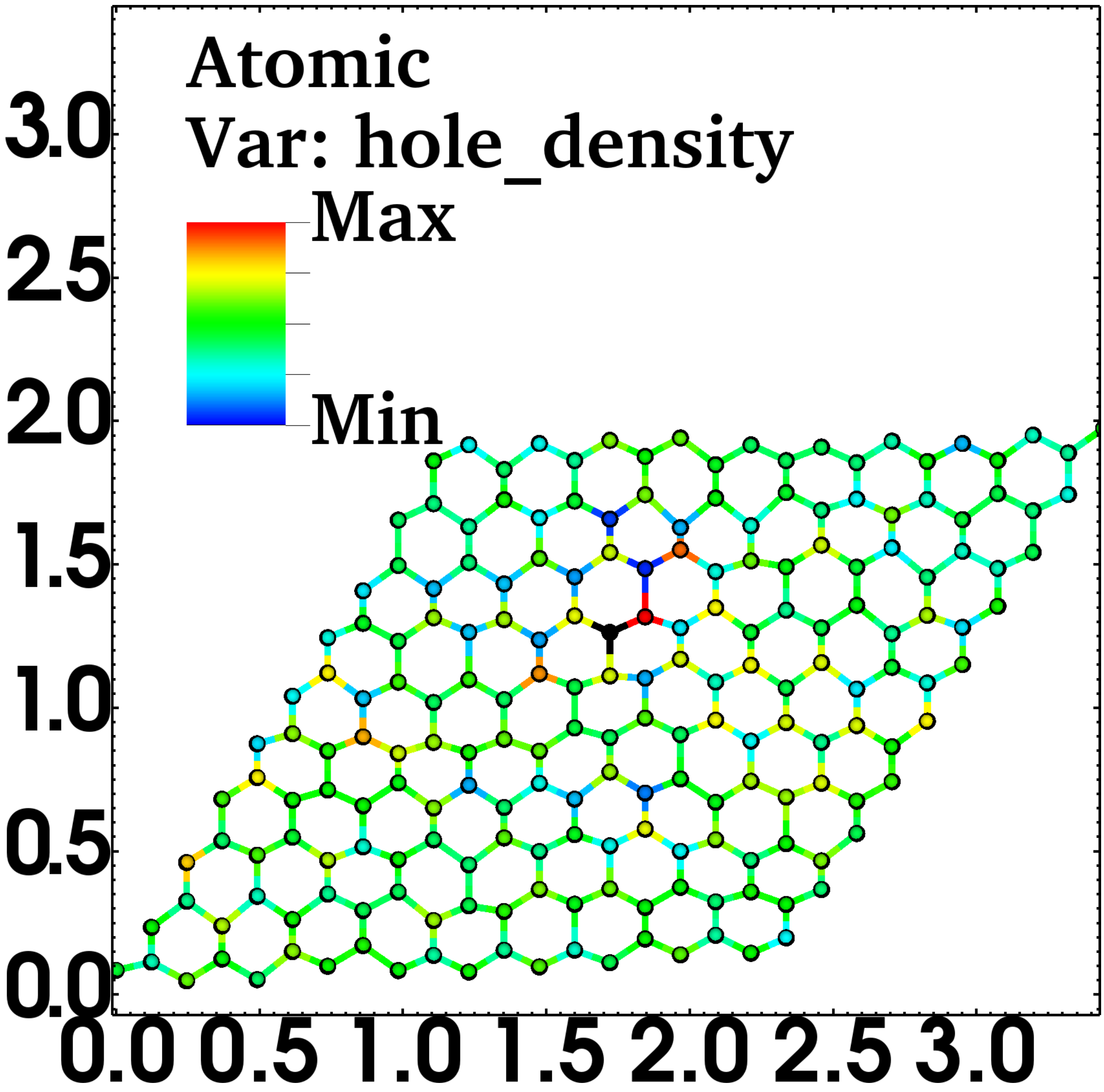

Figure 90. Hole density In-plane deformation-10pm

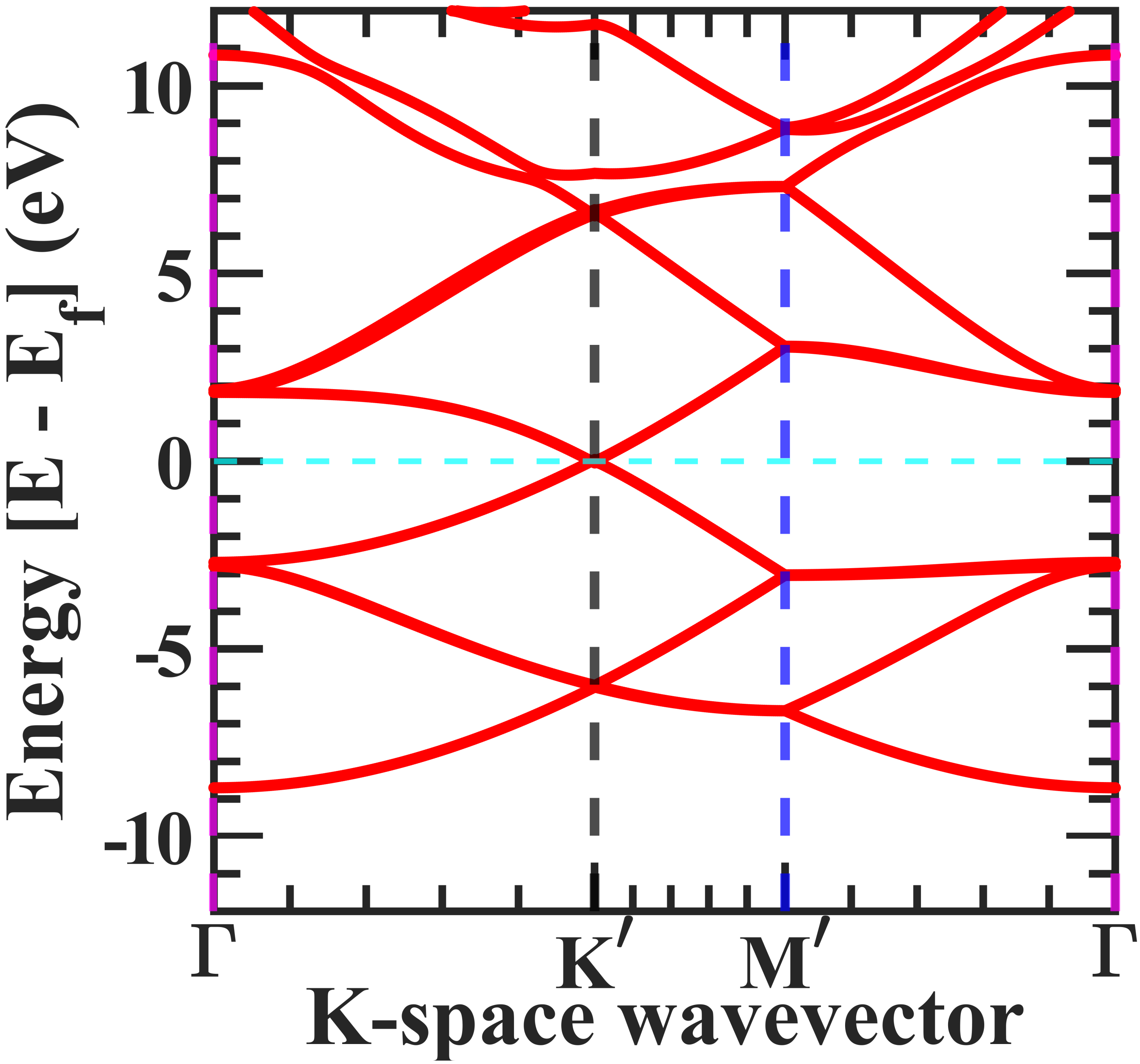

Figure 91. Band structure In-plane deformation-5pm

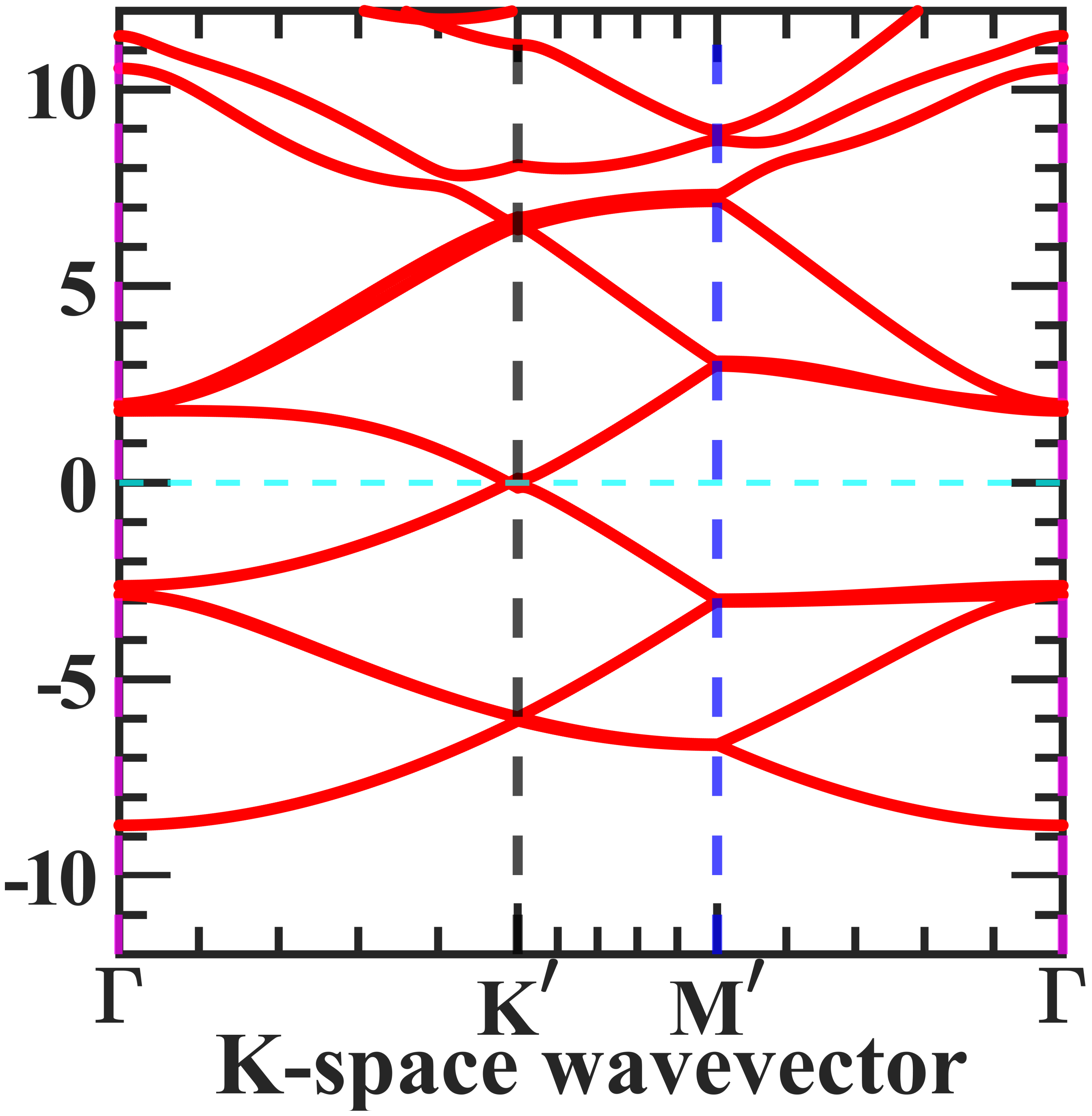

Figure 92. Band structure In-plane deformation-10pm

Impurity Effect

20x20 Nitrogen doped graphene supercell

In the Figure fig. 93 correspond to 20x20 graphene supercell flat structure with substitutional Nitrogen impurity, fig. 94 corresponding density of state, fig. 95 electronic band structure, fig. 96 density of mode, fig. 97 self-consistent Poisson potential due to Nitrogen impurity, fig. 98 electronic density, fig. 99 hole density and fig. 100 to fig. 106 represents spatially resolved electronic orbital state probability amplitude $|\psi|^2$ for first seven eigenvalues of stationary solution of Schrödinger wave-equation from $|\psi_0|^2$ to $|\psi_6|^2$ in the corresponding graphene supercell. In the simulated device, the primitive unit cell has two atoms per cell, and a total of 800 atoms are simulated by a finite element mesh of 3200 point Density of Mode size. The P-D tight-binding model contains three orbitals, namely carbon P_z, and carbon-hydrogen passivated D_{yz}, D_{xz} orbitals. Therefore total degree of free density of Mode in hamiltonian is 2400 variable-sized. The K' and M' are high symmetric point

that corresponds to the folded reduced BZ-zone of graphene supercell.

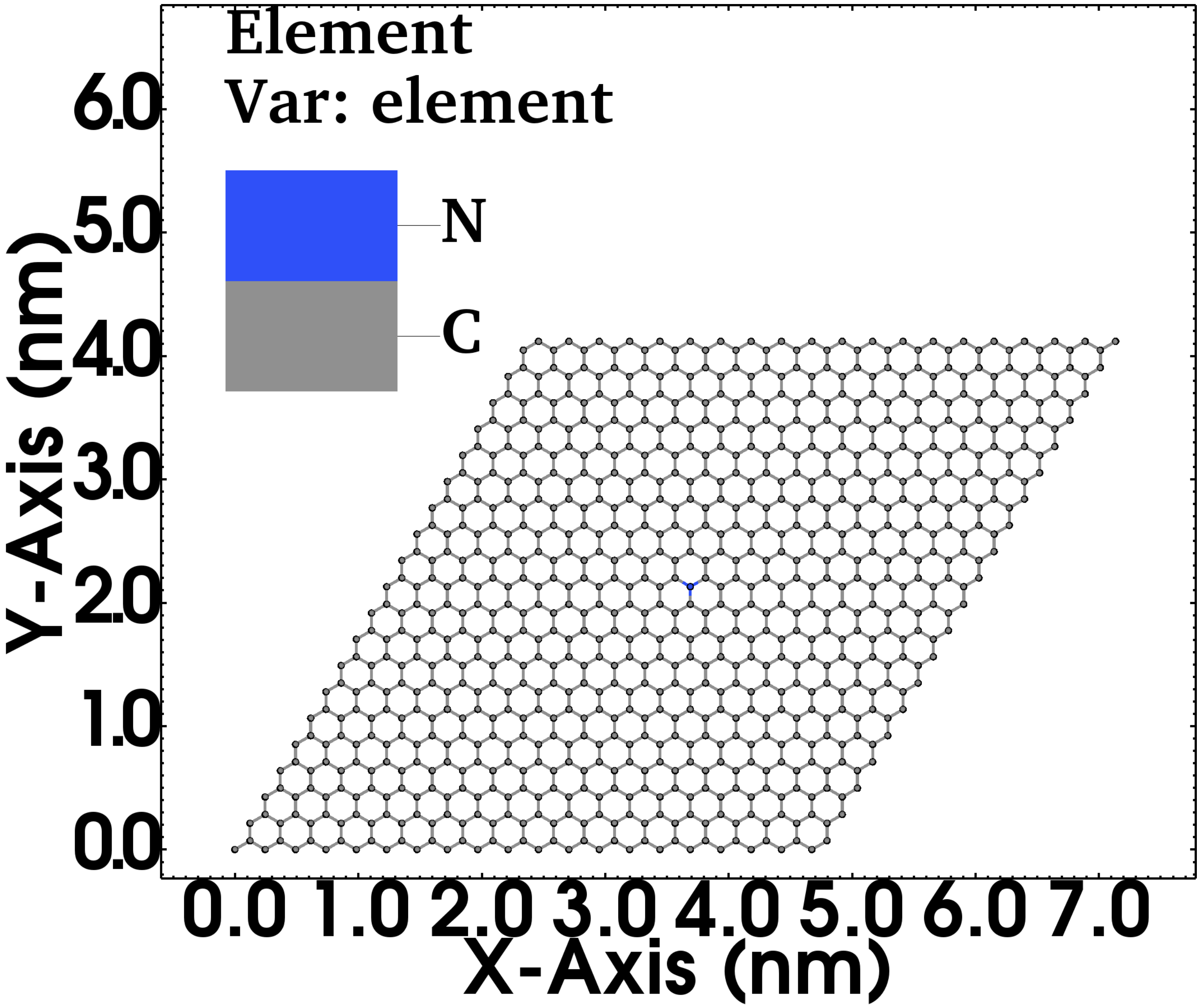

Figure 93. Nitrogen atom substitute 20x20 graphene supercell structure

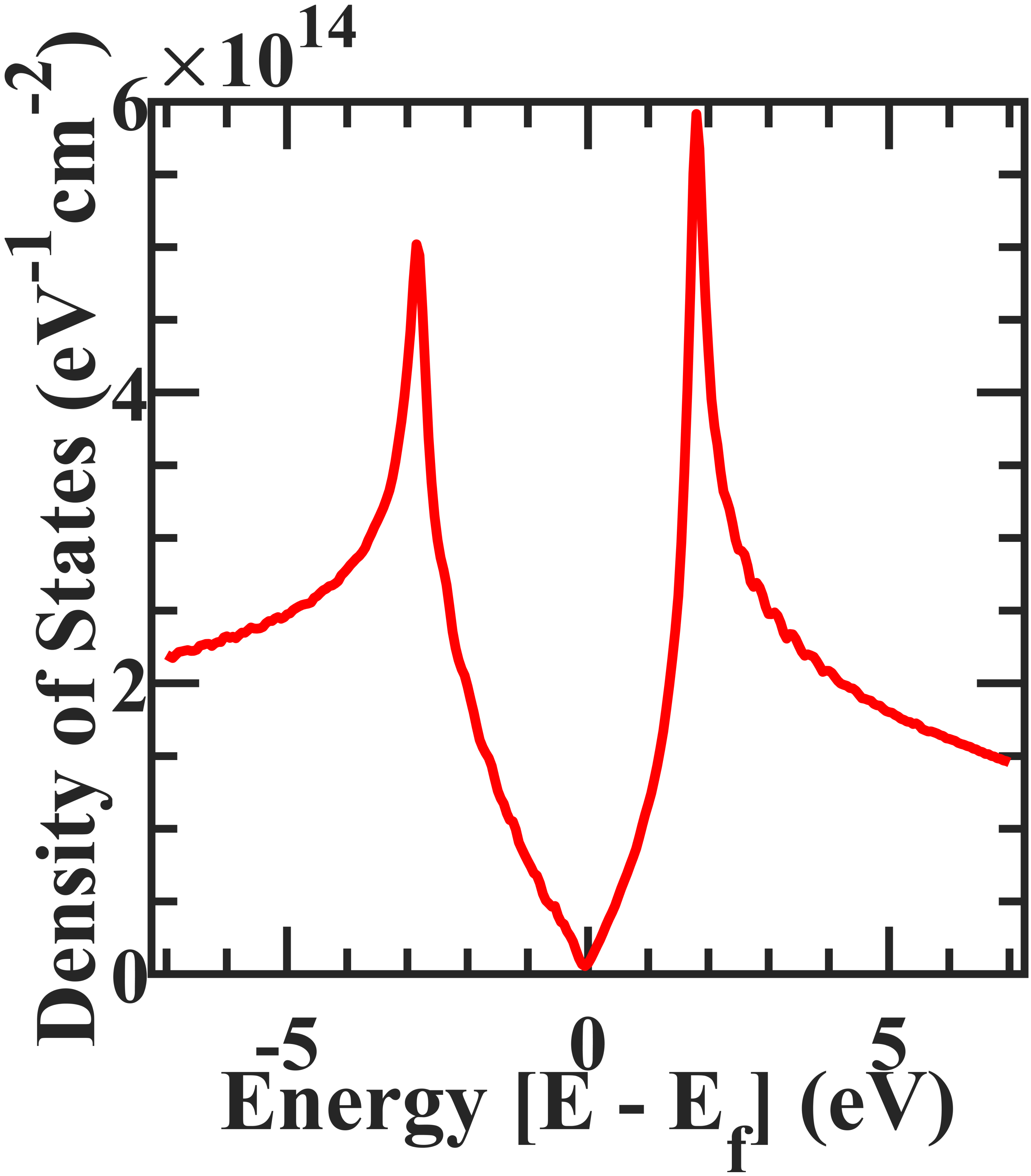

Figure 94. Density of state Nitrogen atom impurity 20x20 graphene supercell

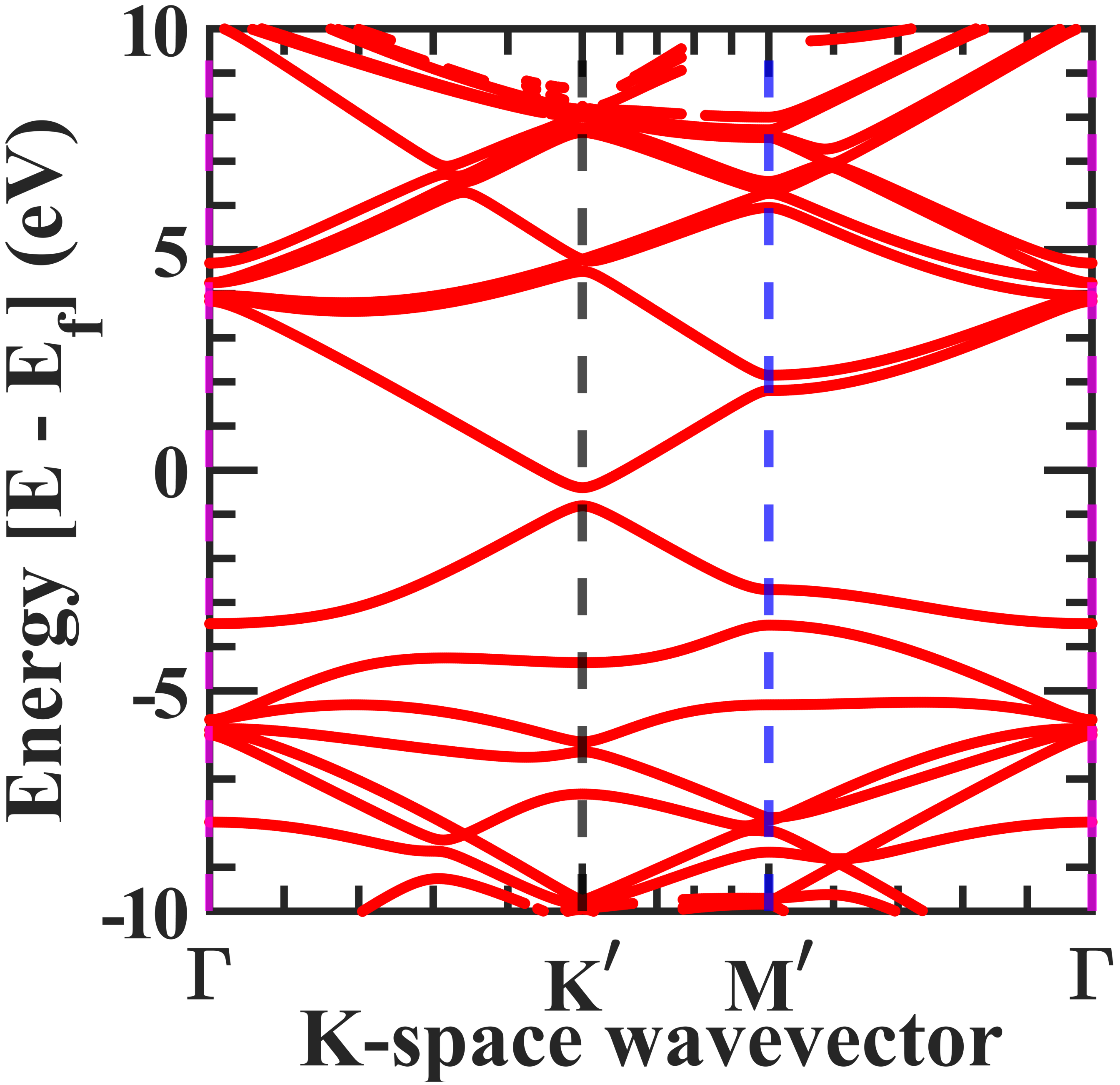

Figure 95. Band structure Nitrogen atom impurity 20x20 graphene supercell

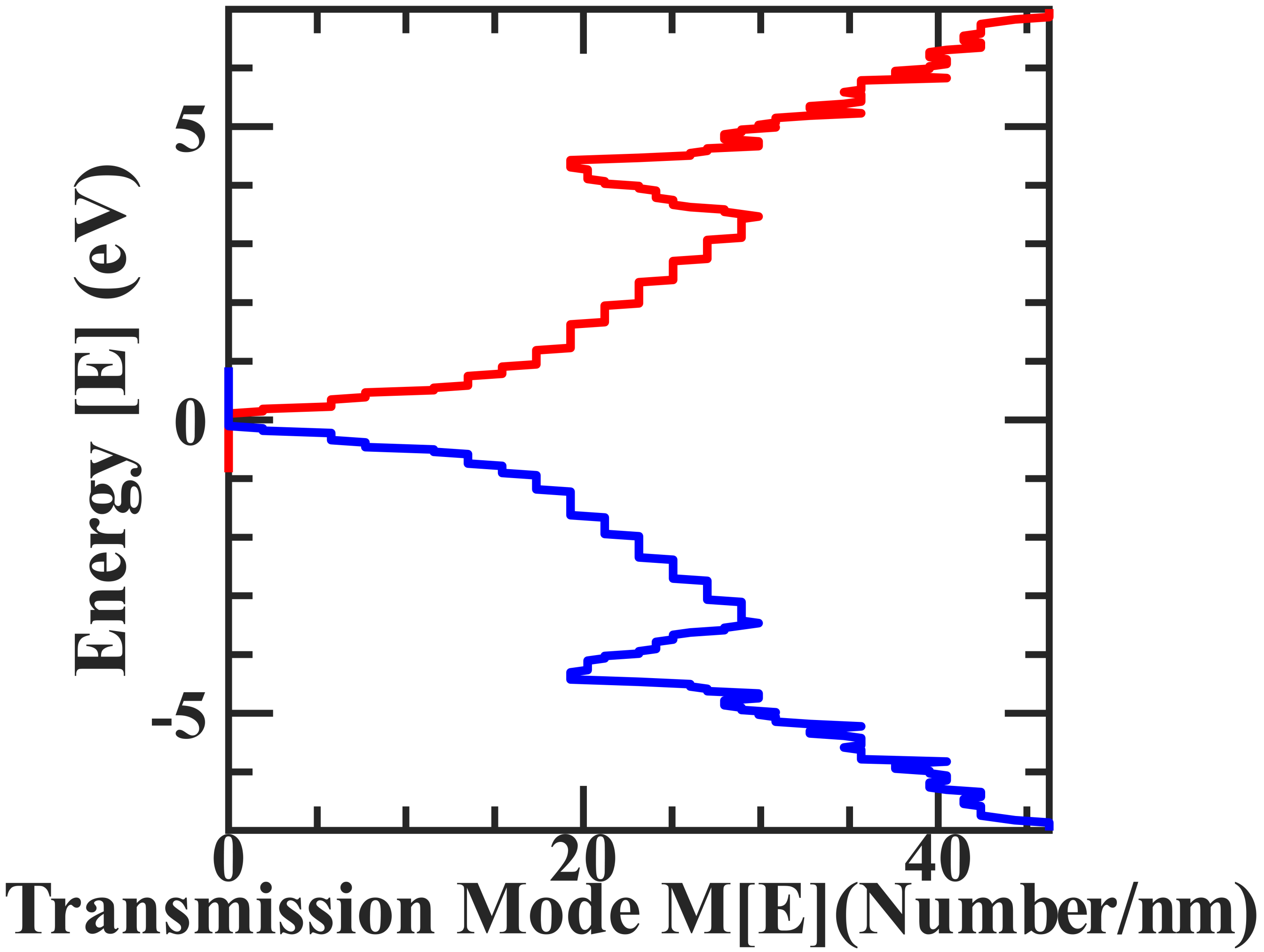

Figure 96. Density of mode Nitrogen impurity 20x20 graphene supercell

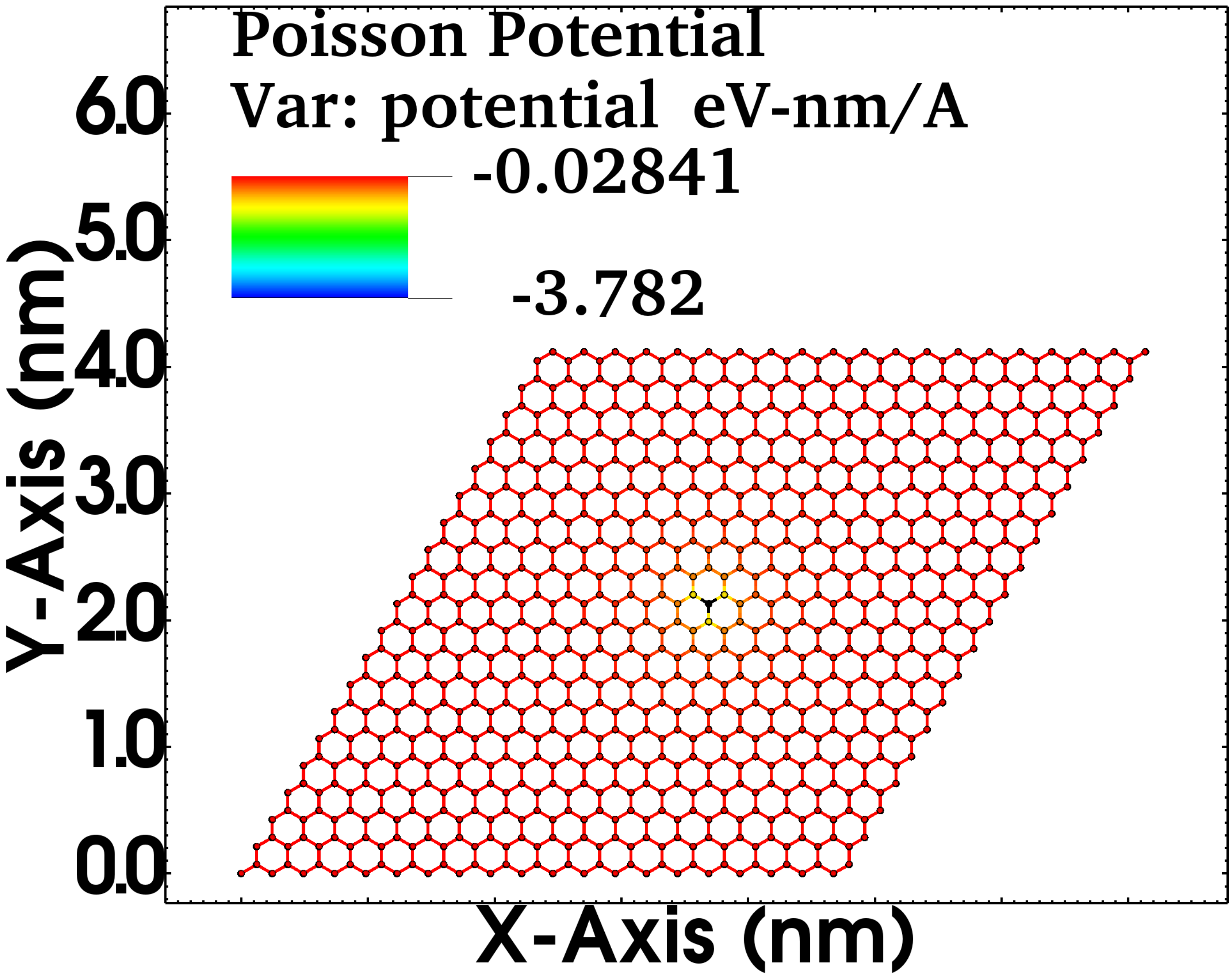

Figure 97. Self-consistent Poisson potential due to Nitrogen impurity 20x20 graphene supercell

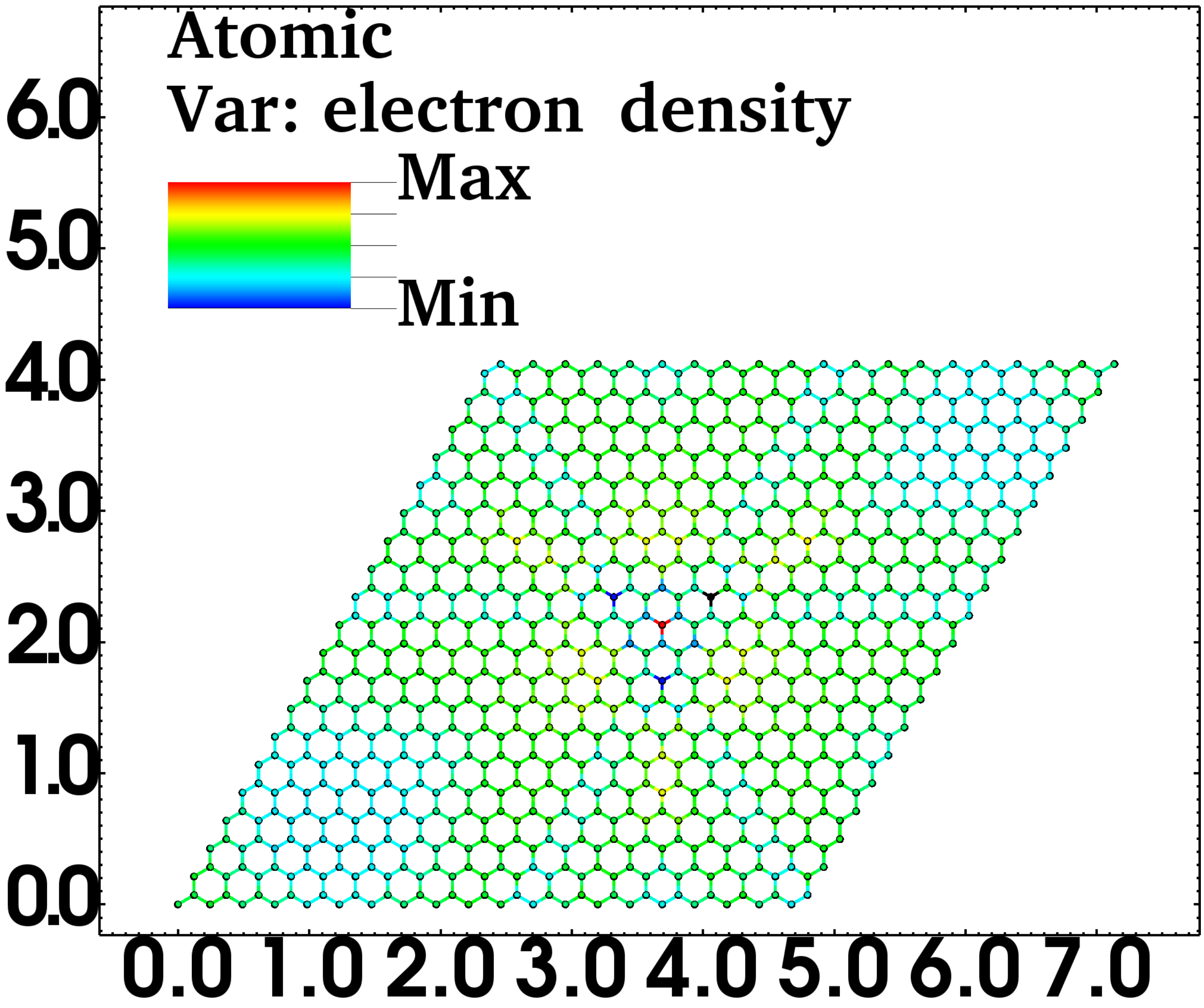

Figure 98. Electron density Nitrogen impurity 20x20 graphene supercell

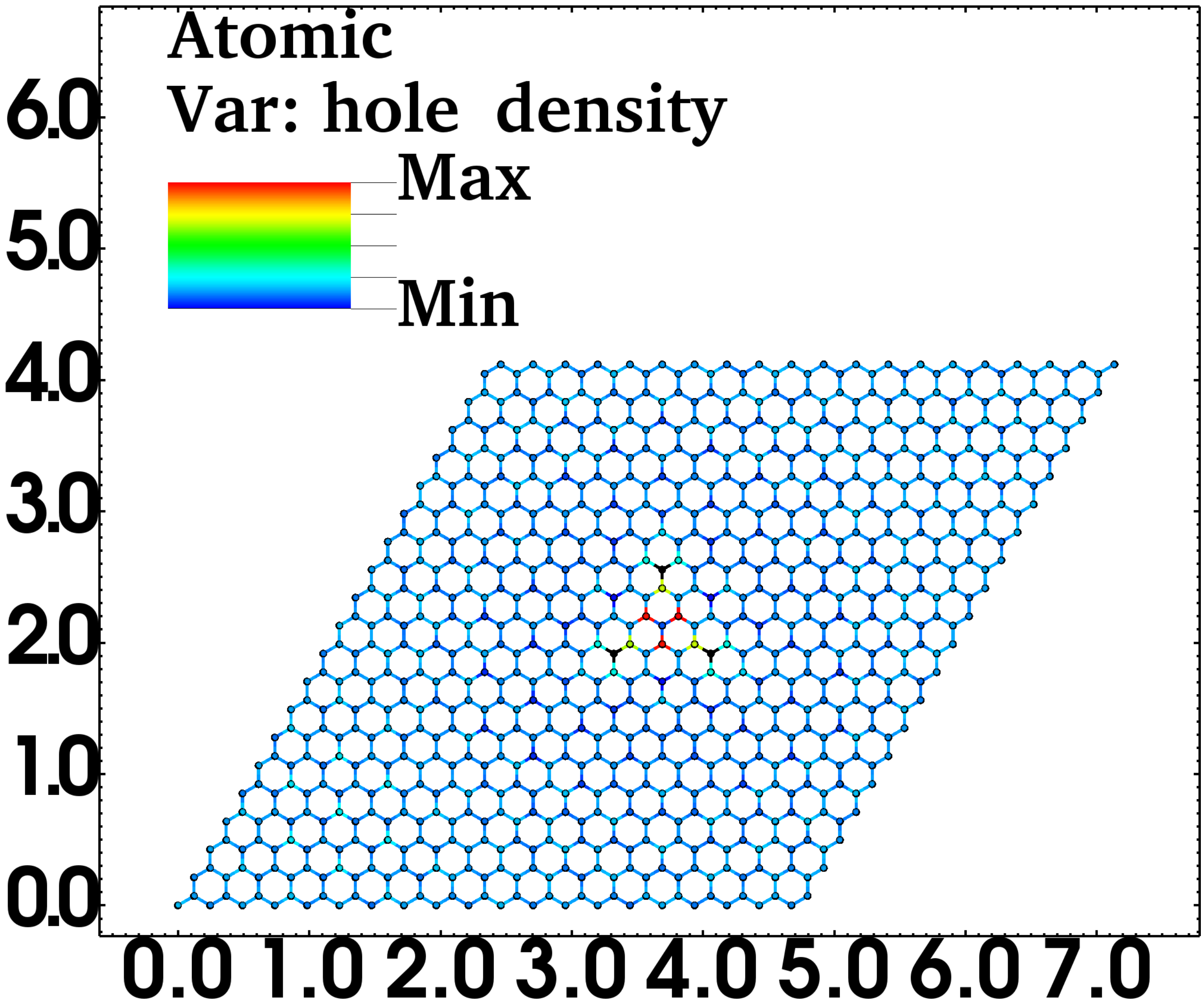

Figure 99. Hole density Nitrogen impurity 20x20 graphene supercell

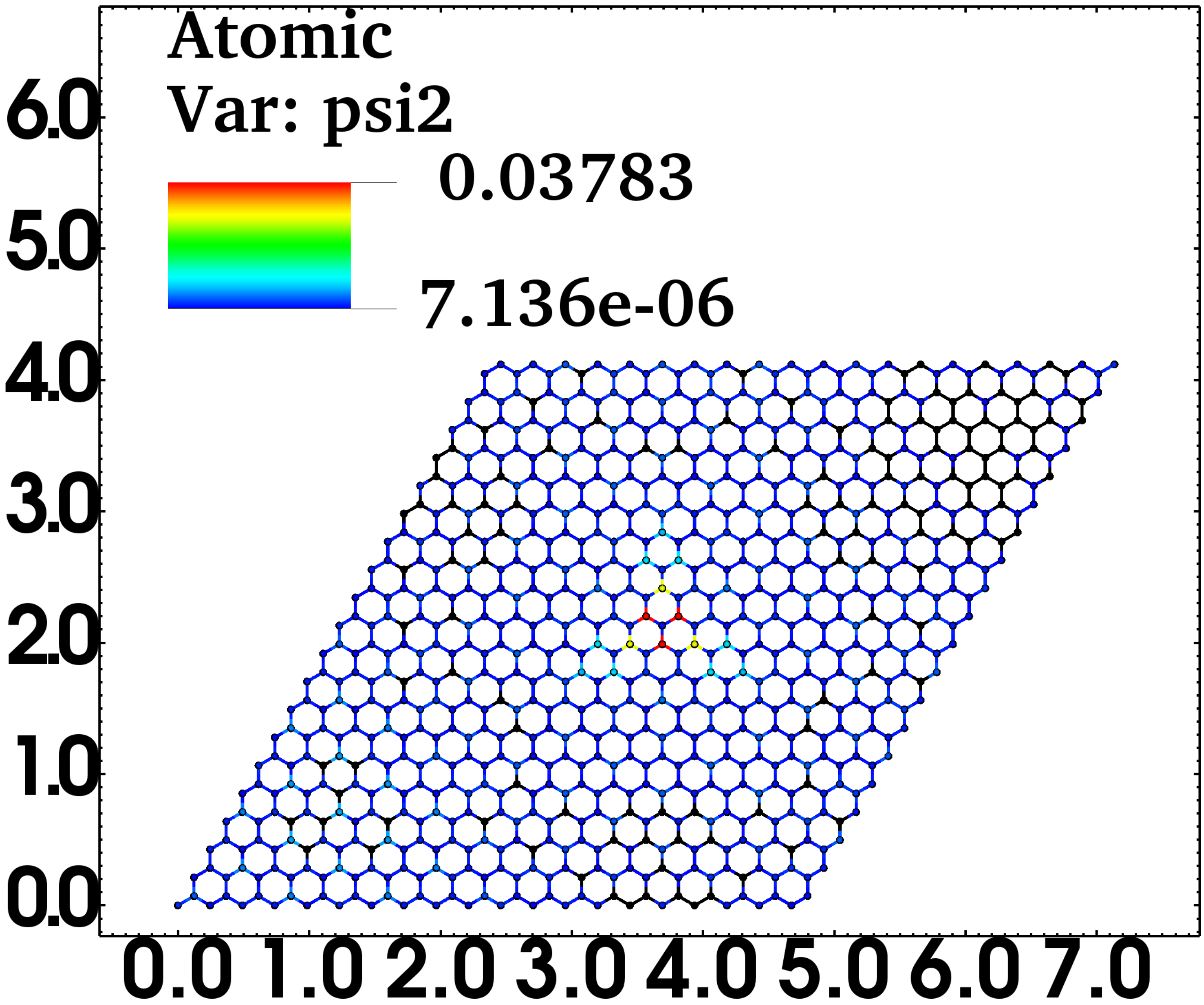

Figure 100. Spatially resolved electronic state $|\psi_0|^2$ Nitrogen impurity 20x20 graphene supercell

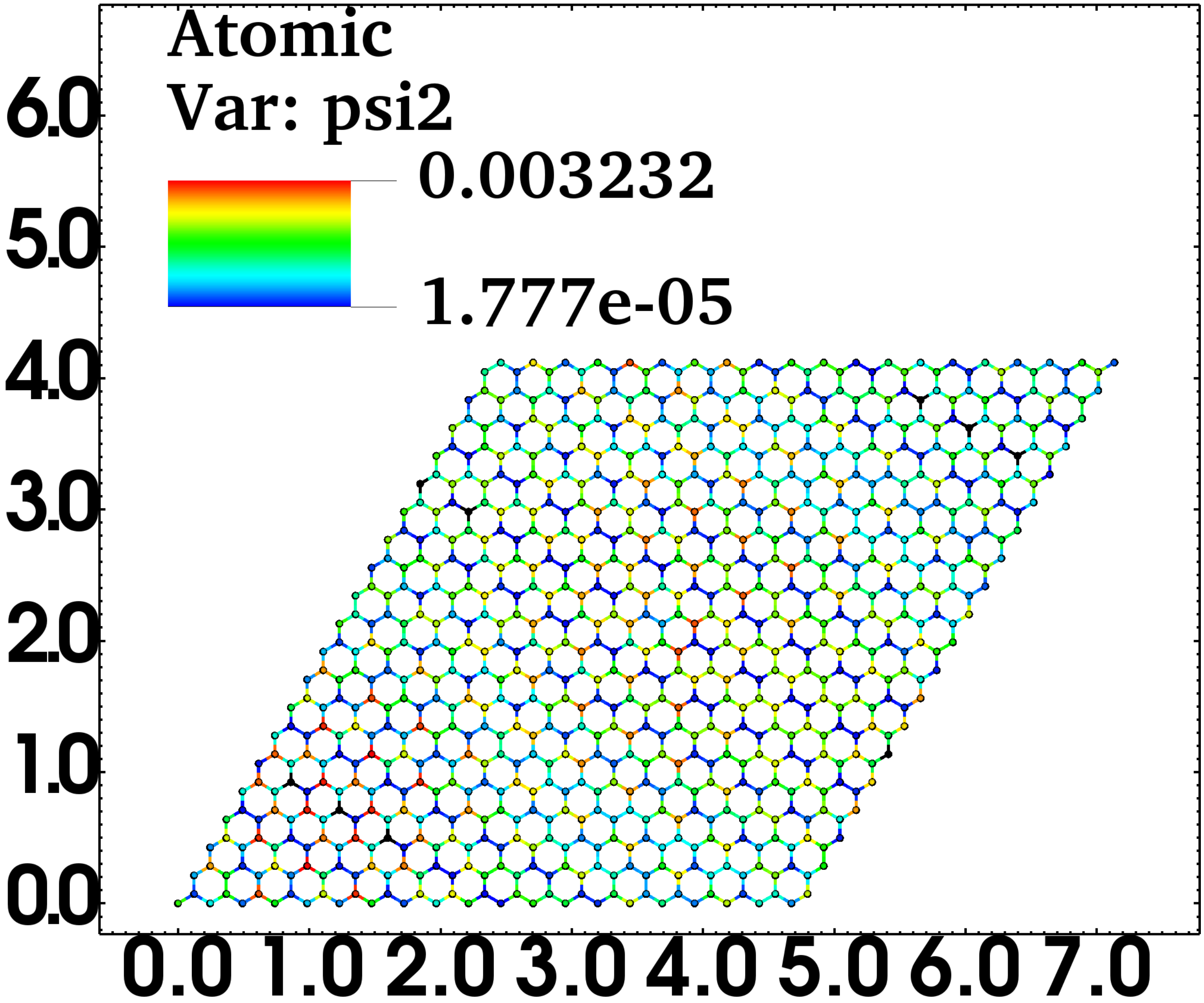

Figure 101. Spatially resolved electronic state $|\psi_1|^2$ Nitrogen impurity 20x20 graphene supercell

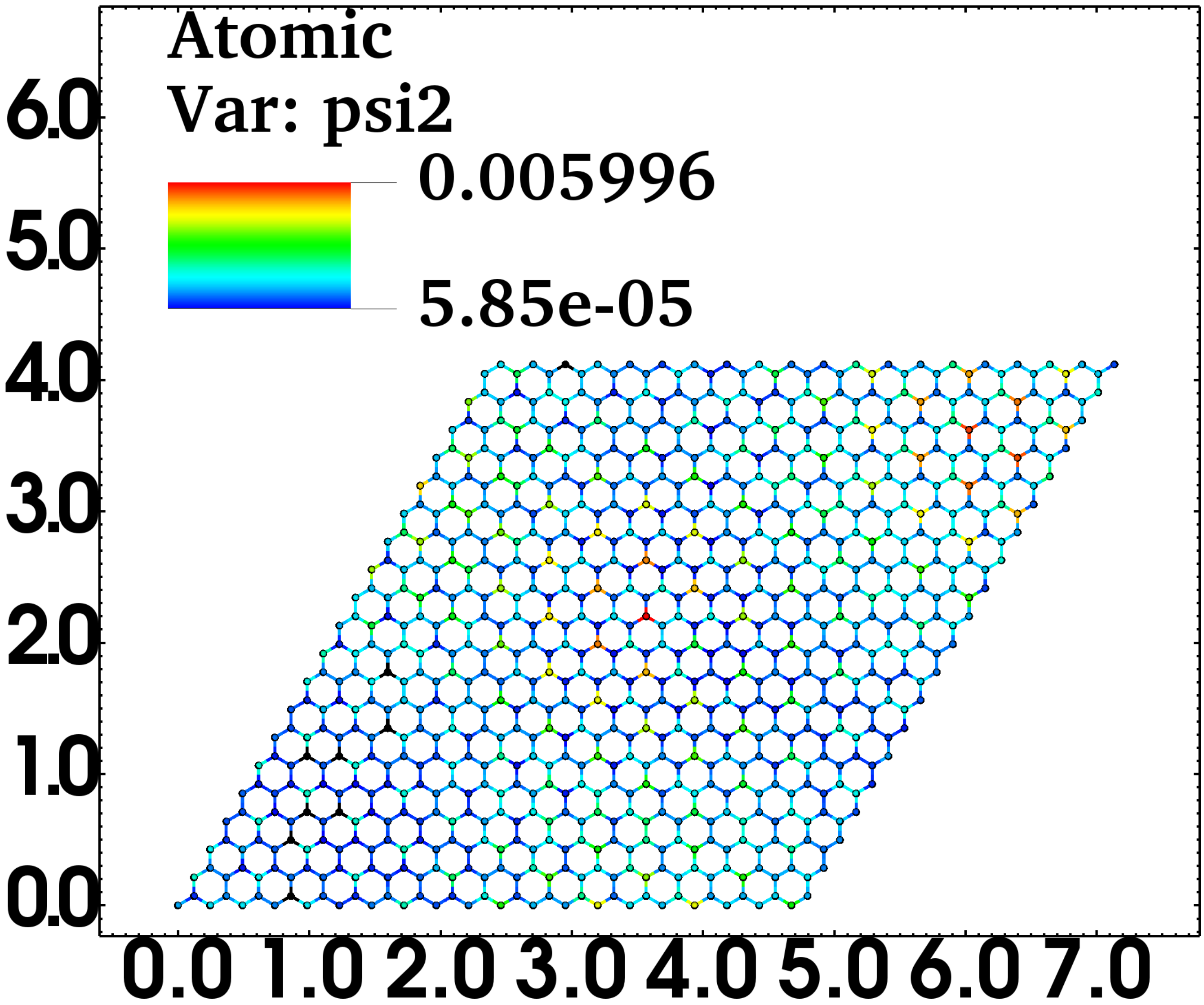

Figure 102. Spatially resolved electronic state $|\psi_2|^2$ Nitrogen impurity 20x20 graphene supercell

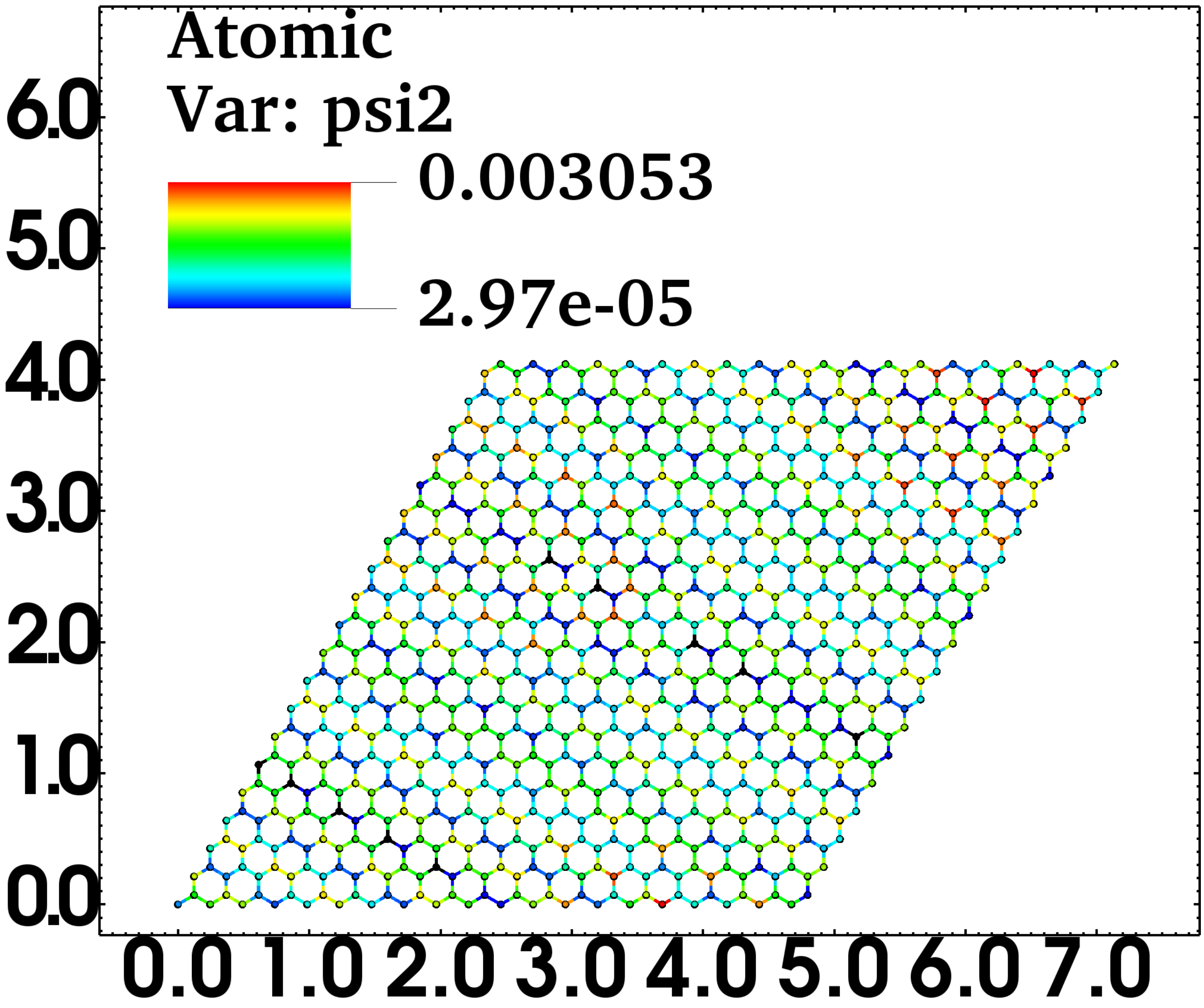

Figure 103. Spatially resolved electronic state $|\psi_3|^2$ Nitrogen impurity 20x20 graphene supercell

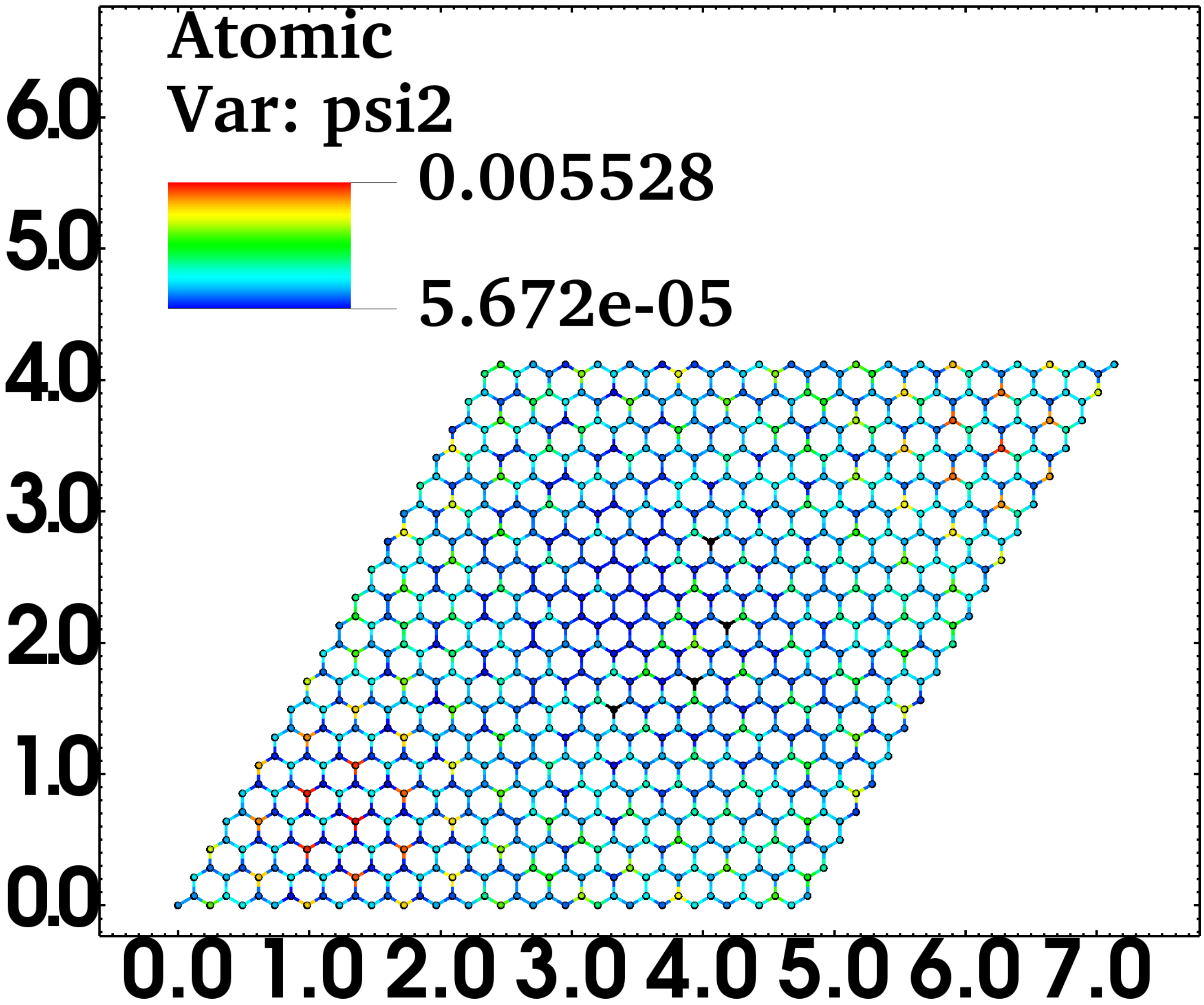

Figure 104. Spatially resolved electronic state $|\psi_4|^2$ Nitrogen impurity 20x20 graphene supercell

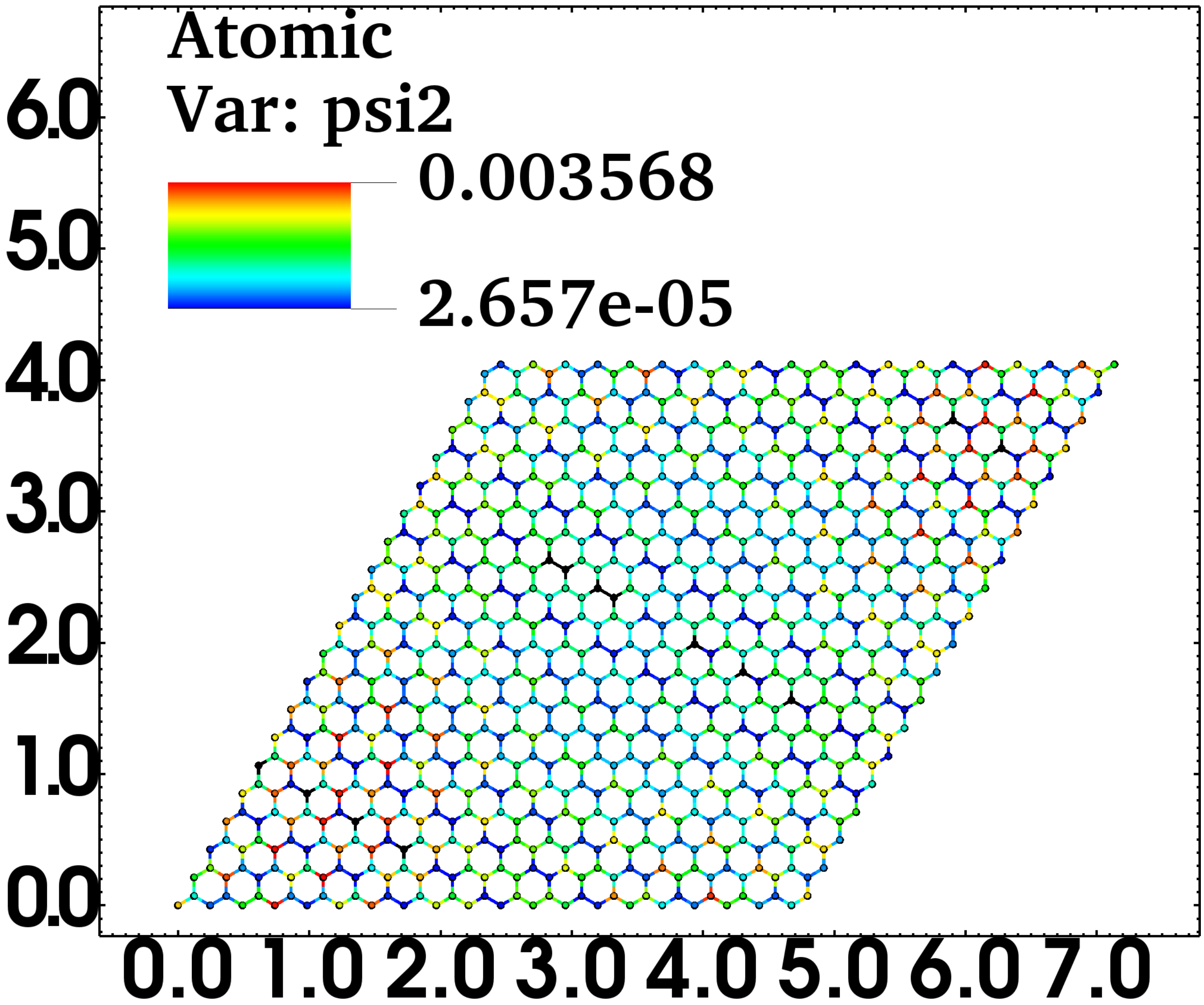

Figure 105. Spatially resolved electronic state $|\psi_5|^2$ Nitrogen impurity 20x20 graphene supercell

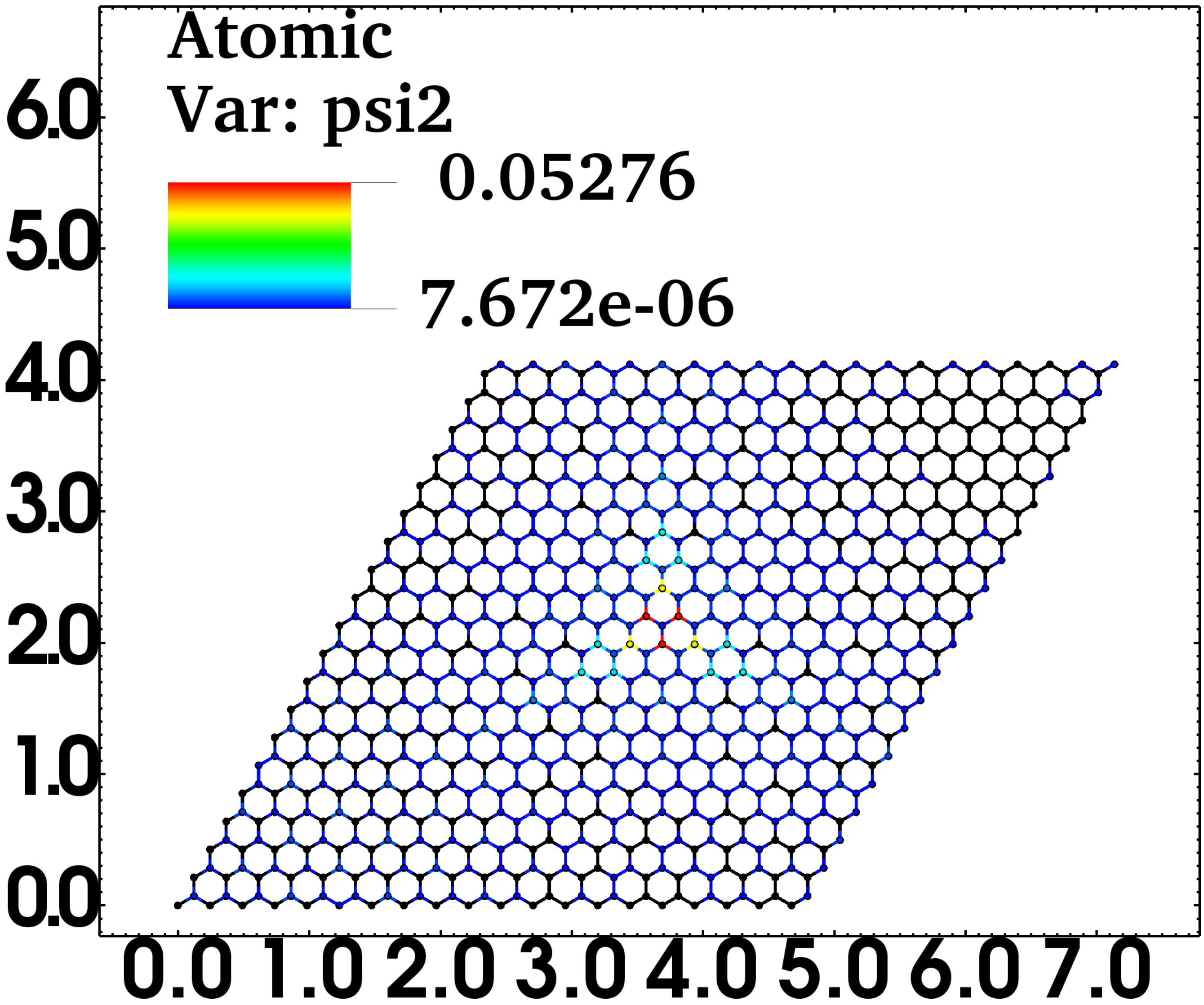

Figure 106. Spatially resolved electronic state $|\psi_6|^2$ Nitrogen impurity 20x20 graphene supercell

50x50 Nitrogen doped graphene supercell

In the Figure fig. 107 correspond to 50x50 graphene supercell flat structure with substitutional Nitrogen impurity, fig. 108 corresponding density of state, fig. 109 electronic band structure, fig. 110 density of mode, fig. 111 self-consistent Poisson potential due to Nitrogen impurity, fig. 112 electronic density, fig. 113 hole density and fig. 114 to fig. 120 represents spatially resolved electronic orbital state probability amplitude $|\psi|^2$ for first seven eigenvalues of stationary solution of Schrödinger wave-equation from $|\psi_0|^2$ to $|\psi_6|^2$ in the corresponding graphene supercell. In the simulated device, the primitive unit cell has two atoms per cell, and a total of 5000 atoms are simulated by a finite element mesh of 20000 point Density of Mode size. The P-D tight-binding model contains three orbitals, namely carbon P_z, and carbon-hydrogen passivated D_{yz}, D_{xz} orbitals. Therefore total degree of free density of Mode in hamiltonian is 15000 variable-sized. The K' and M' are high symmetric point that corresponds to the folded reduced BZ-zone of graphene supercell.

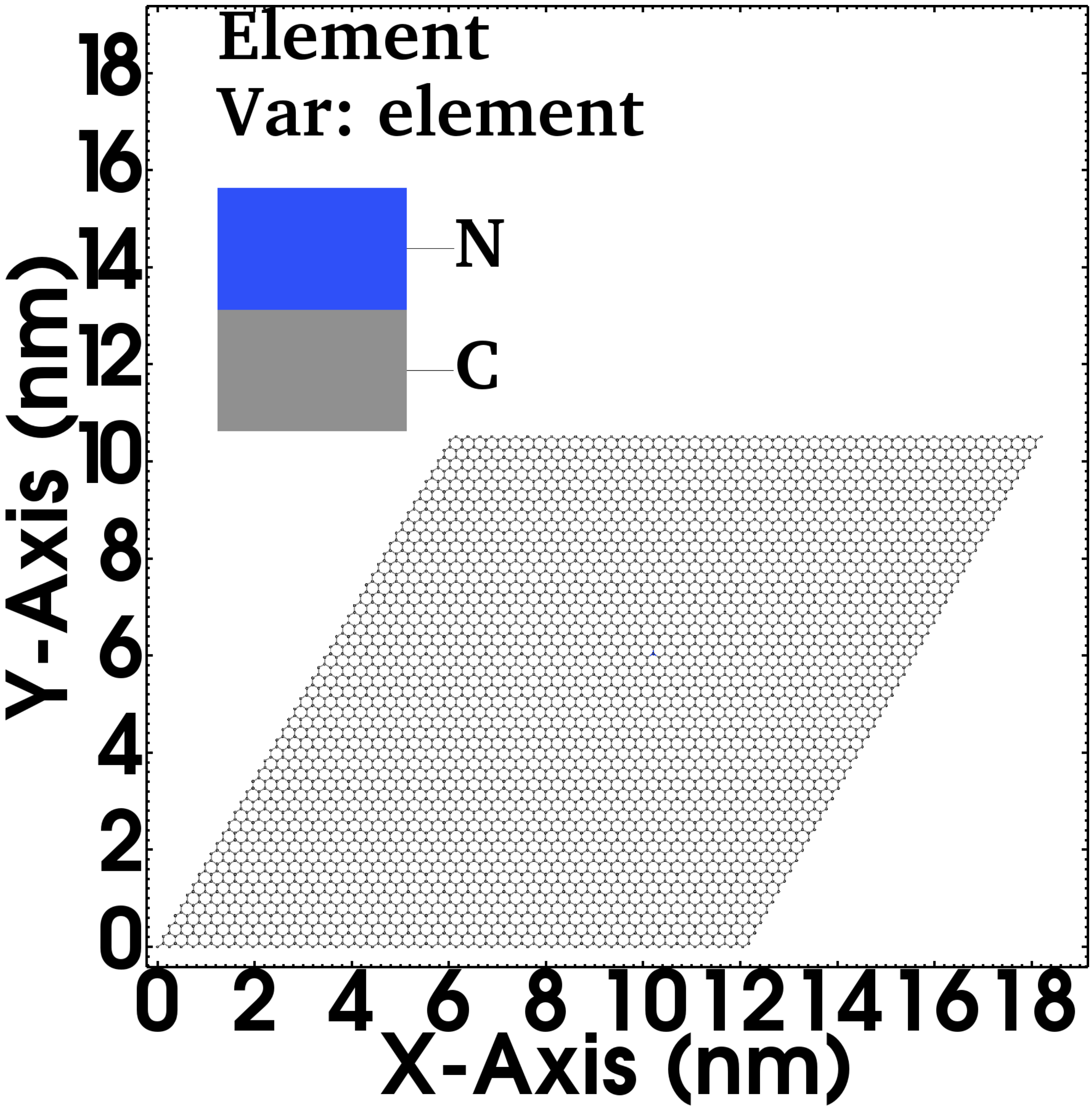

Figure 107. Nitrogen atom substitute structure 50x50 graphene supercell

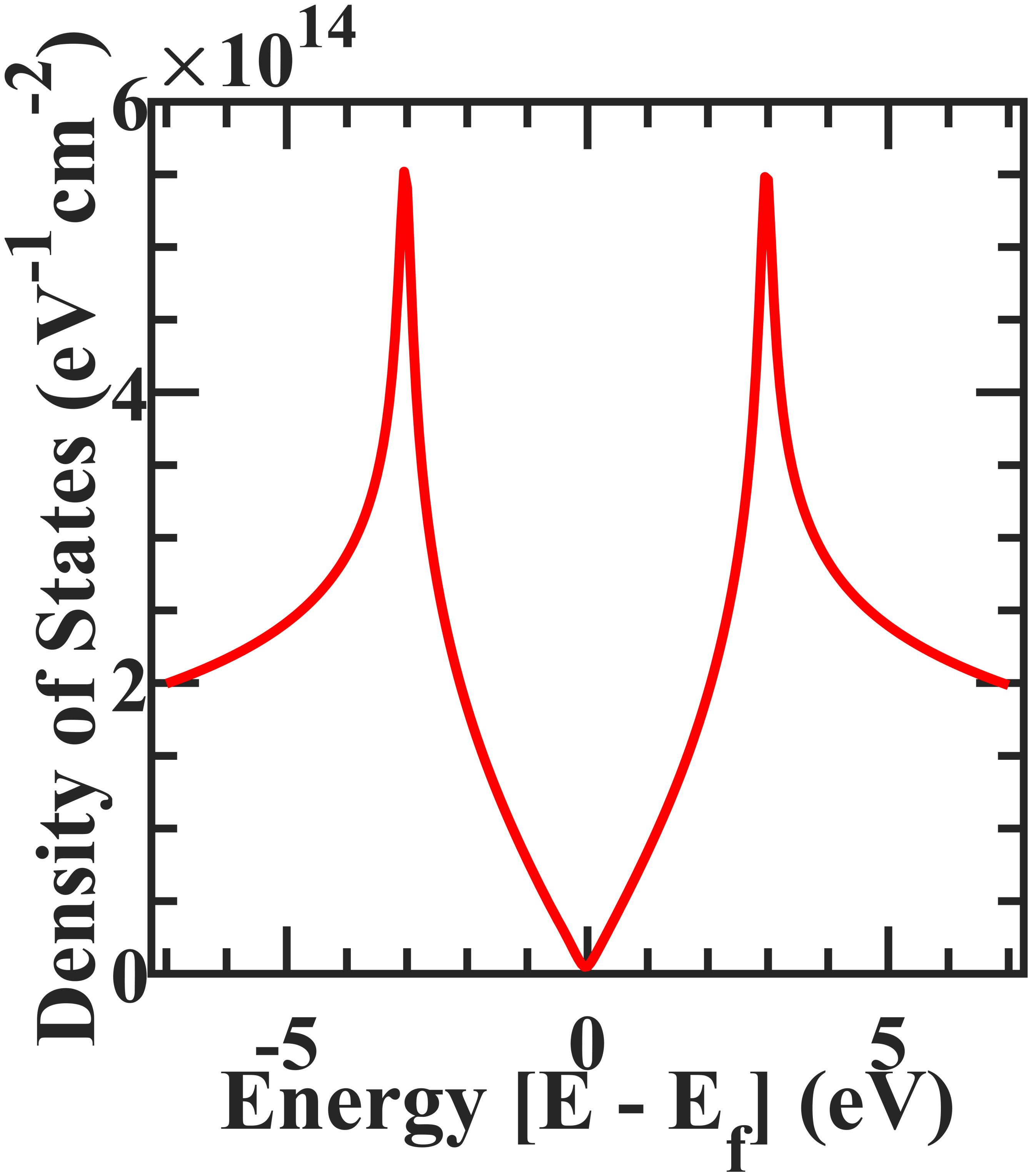

Figure 108. Density of state Nitrogen impurity 50x50 graphene supercell

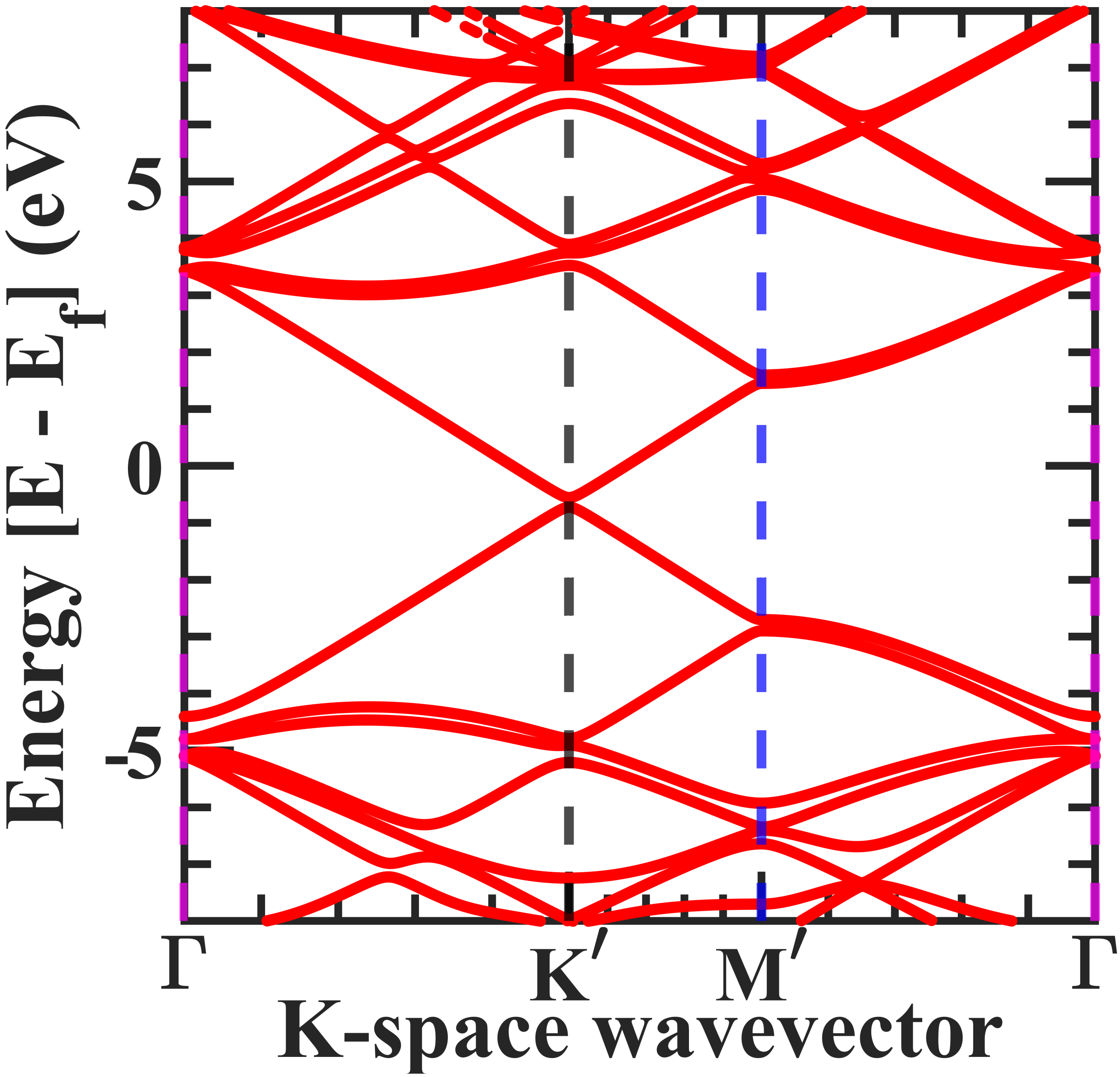

Figure 109. Band structure Nitrogen impurity 50x50 graphene supercell

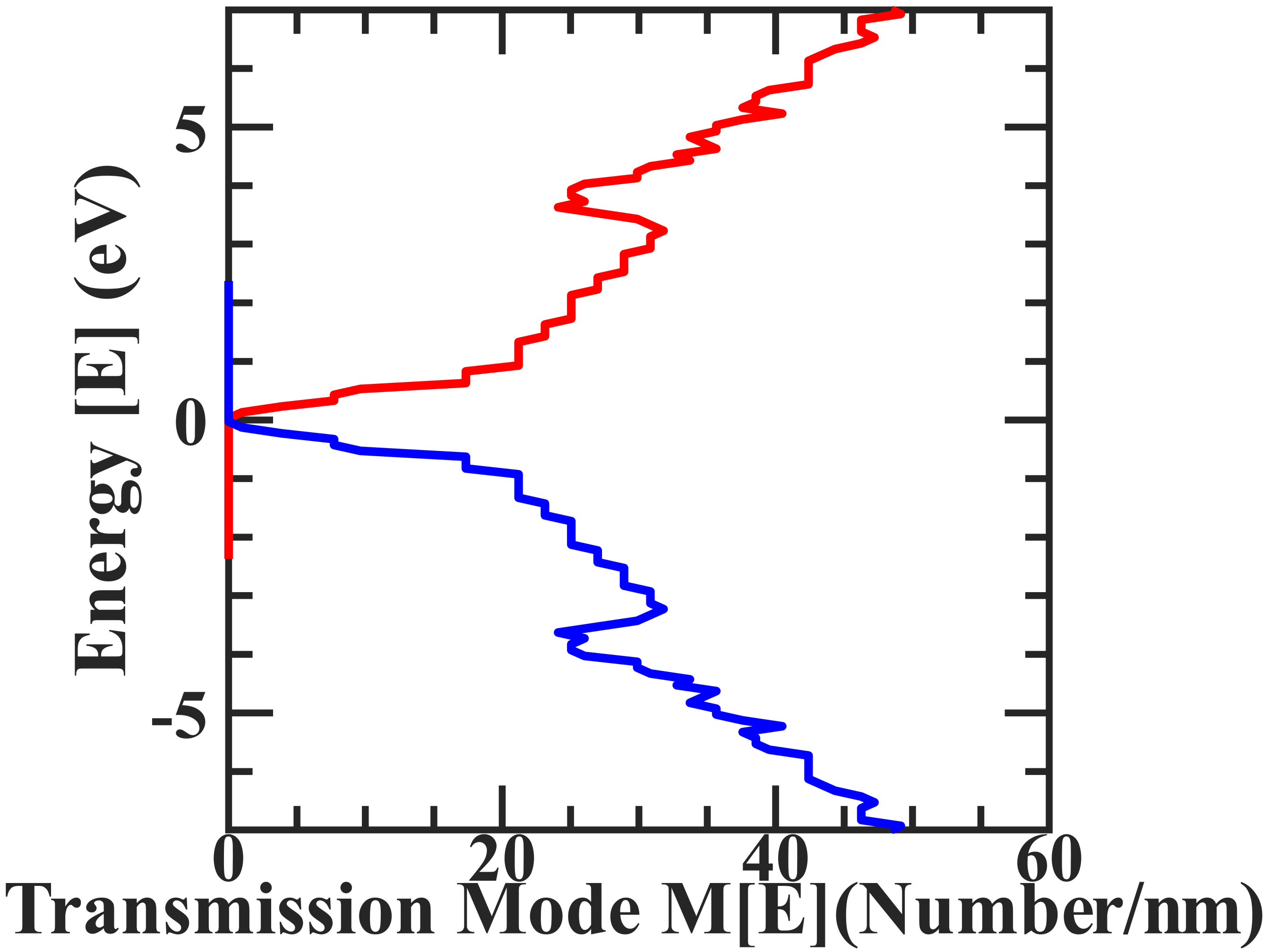

Figure 110. Density of mode Nitrogen impurity 50x50 graphene supercell

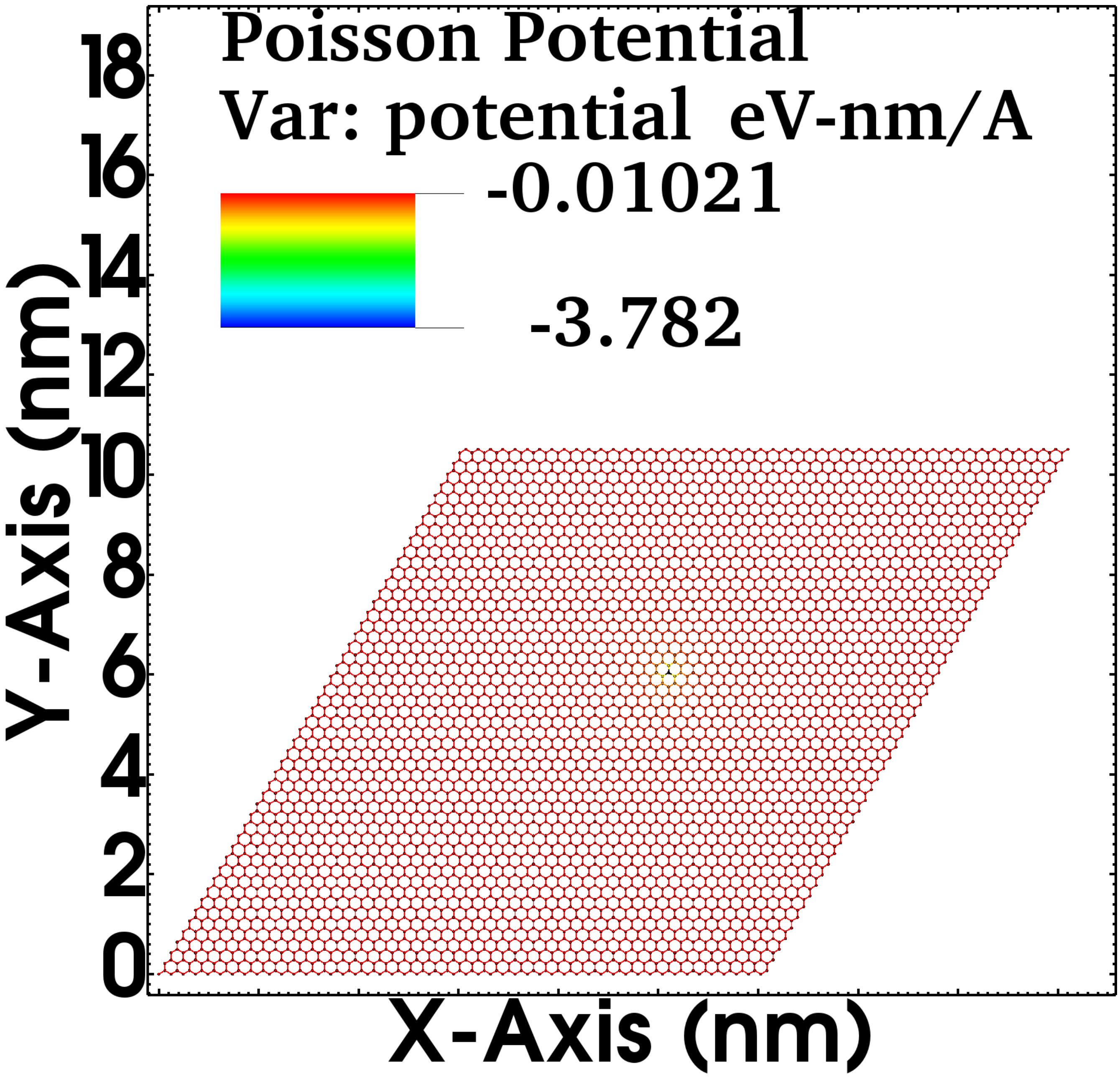

Figure 111. Self-consistent Poisson potential due to Nitrogen impurity 50x50 graphene supercell

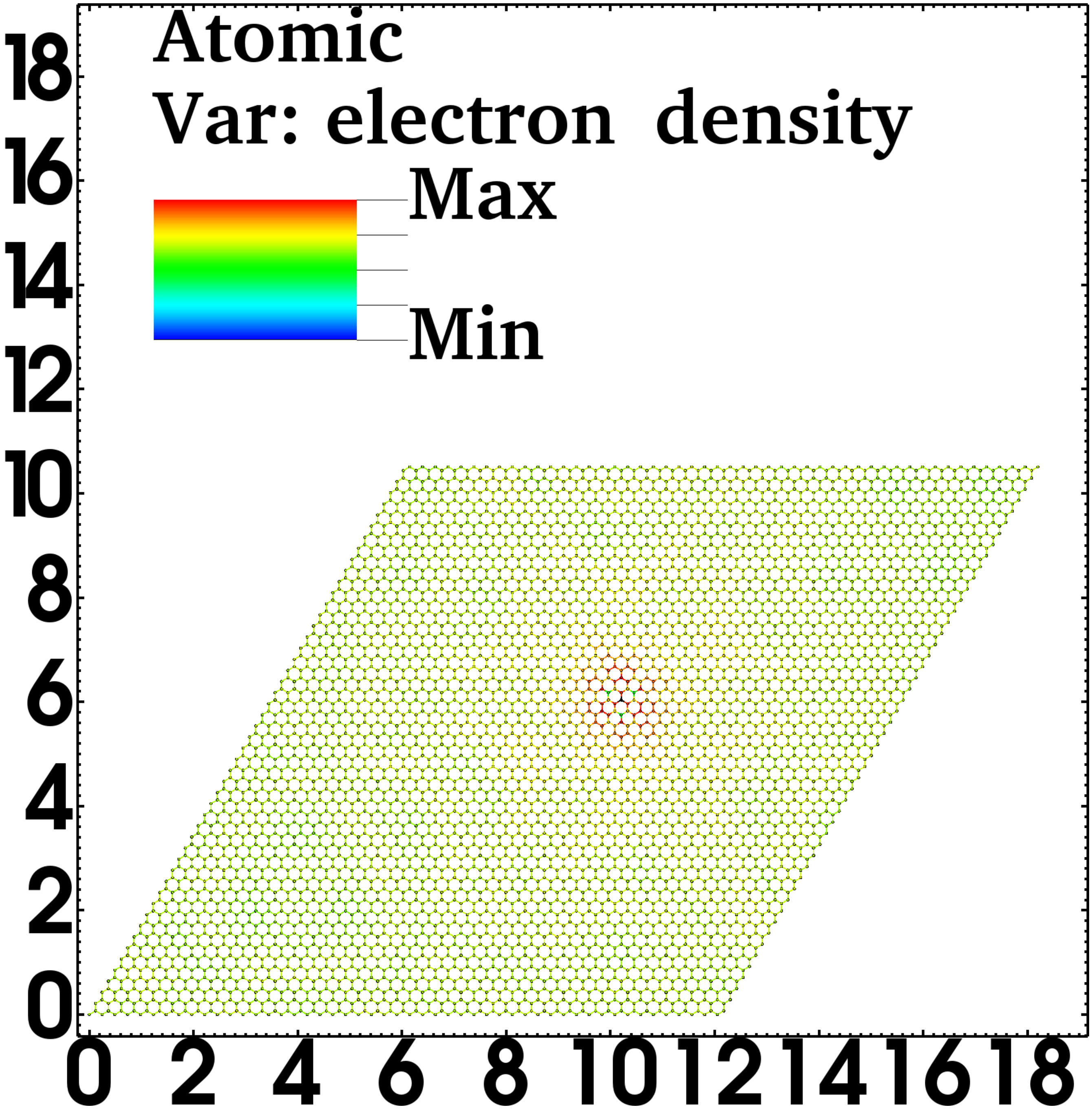

Figure 112. Electron density Nitrogen impurity 50x50 graphene supercell

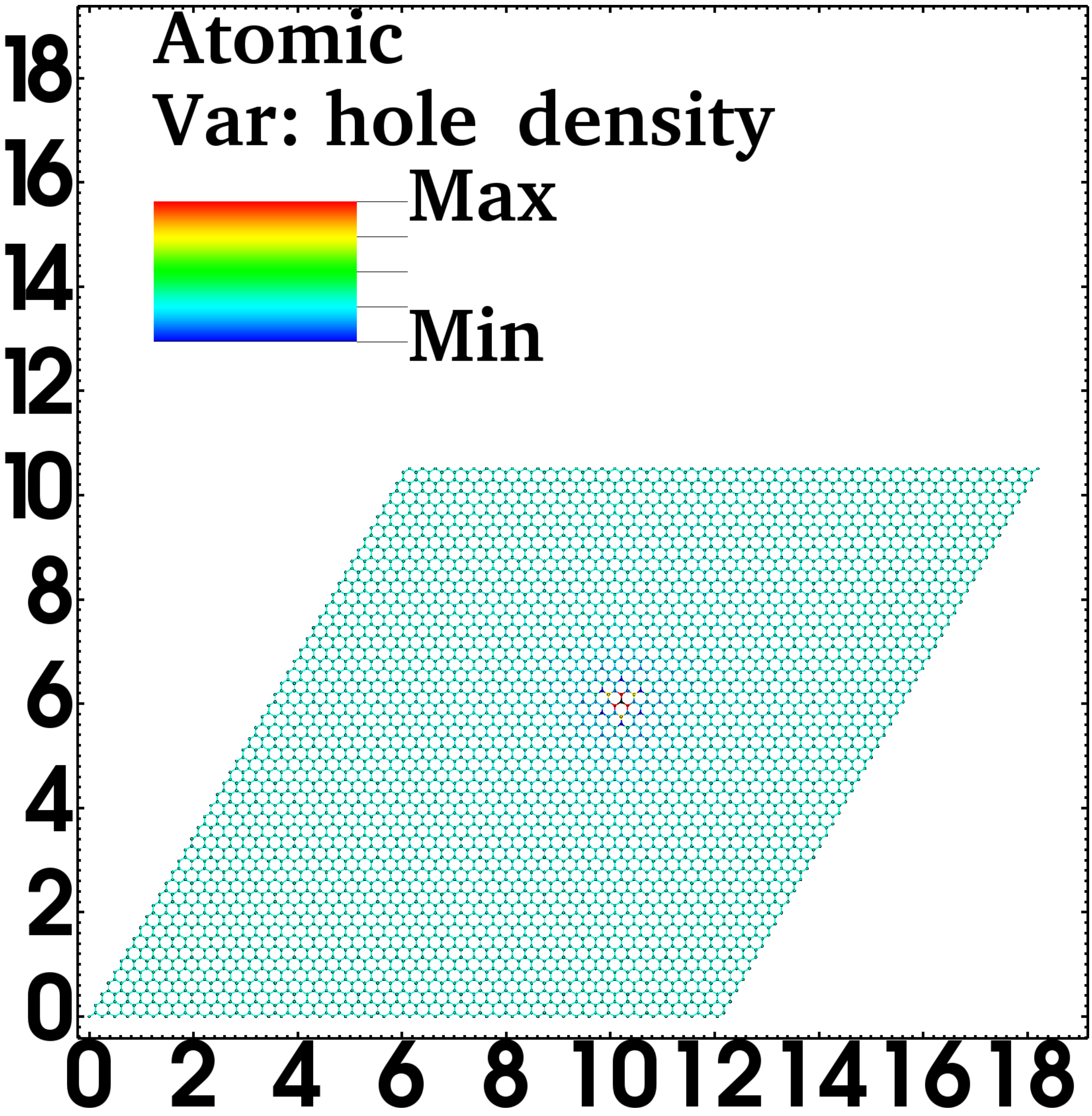

Figure 113. Hole density Nitrogen impurity 50x50 graphene supercell

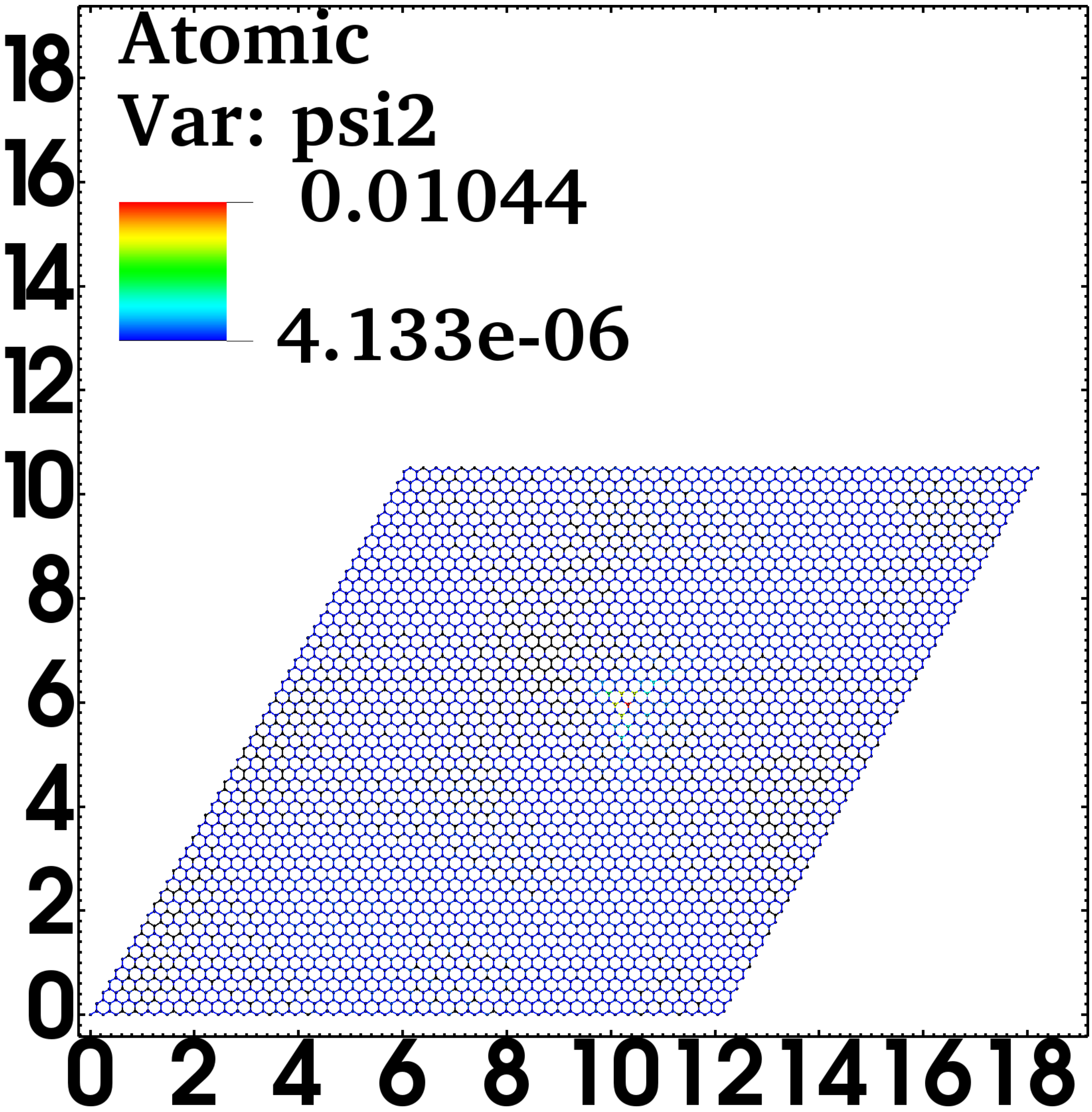

Figure 114. Spatially resolved electronic state $|\psi_0|^2$ Nitrogen impurity 50x50 graphene supercell

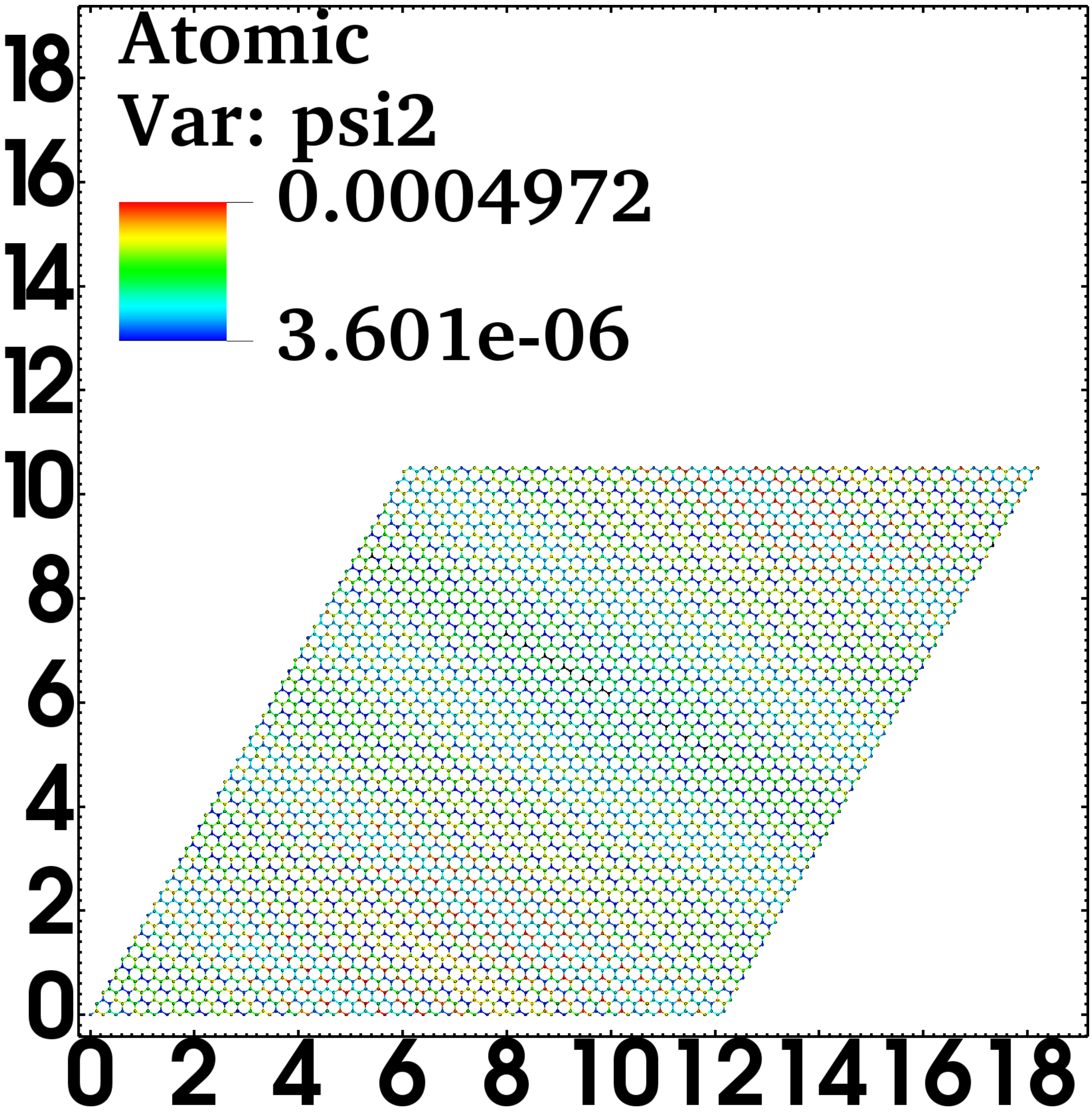

Figure 115. Spatially resolved electronic state $|\psi_1|^2$ Nitrogen impurity 50x50 graphene supercell

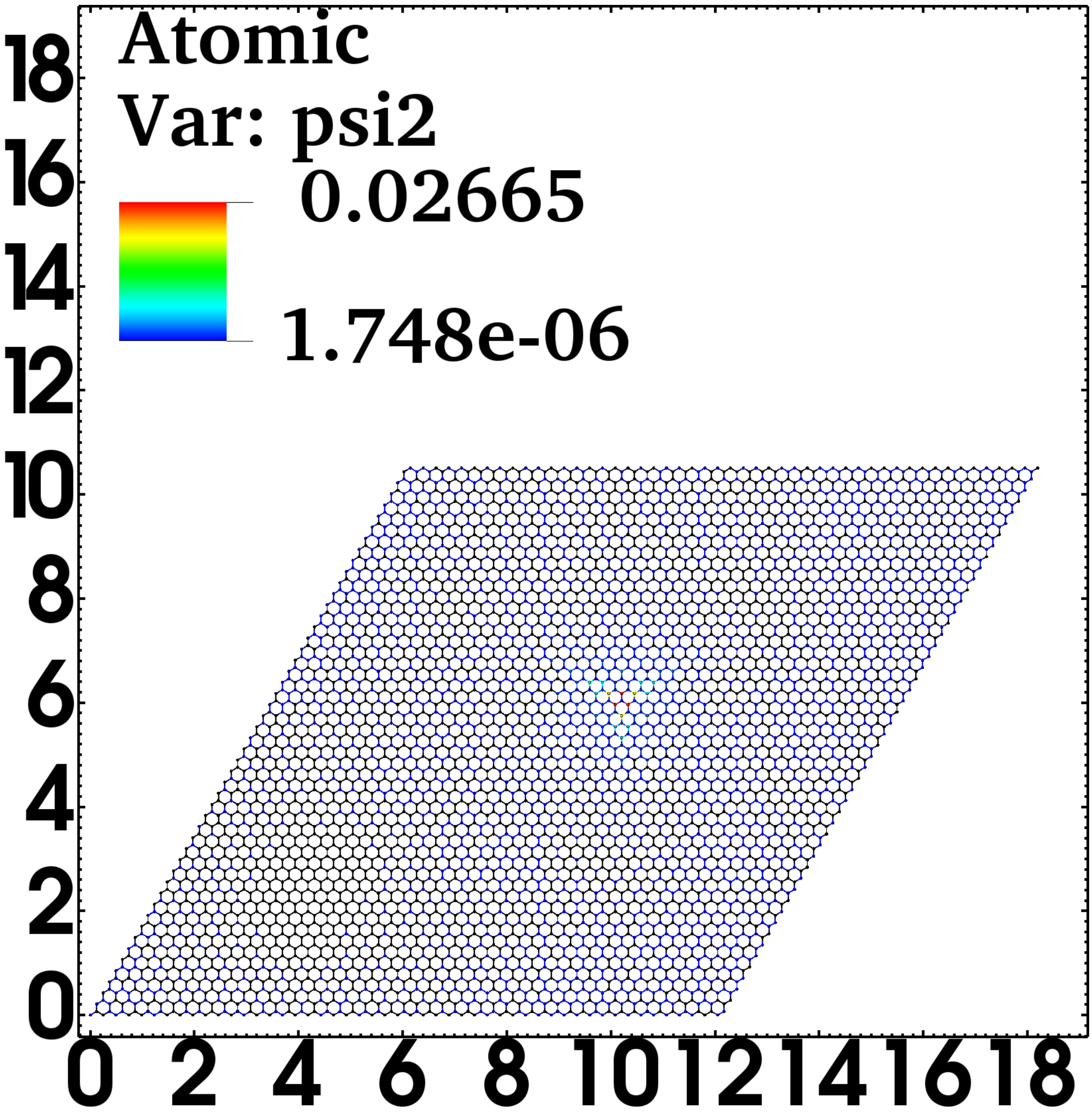

Figure 116. Spatially resolved electronic state $|\psi_2|^2$ Nitrogen impurity 50x50 graphene supercell

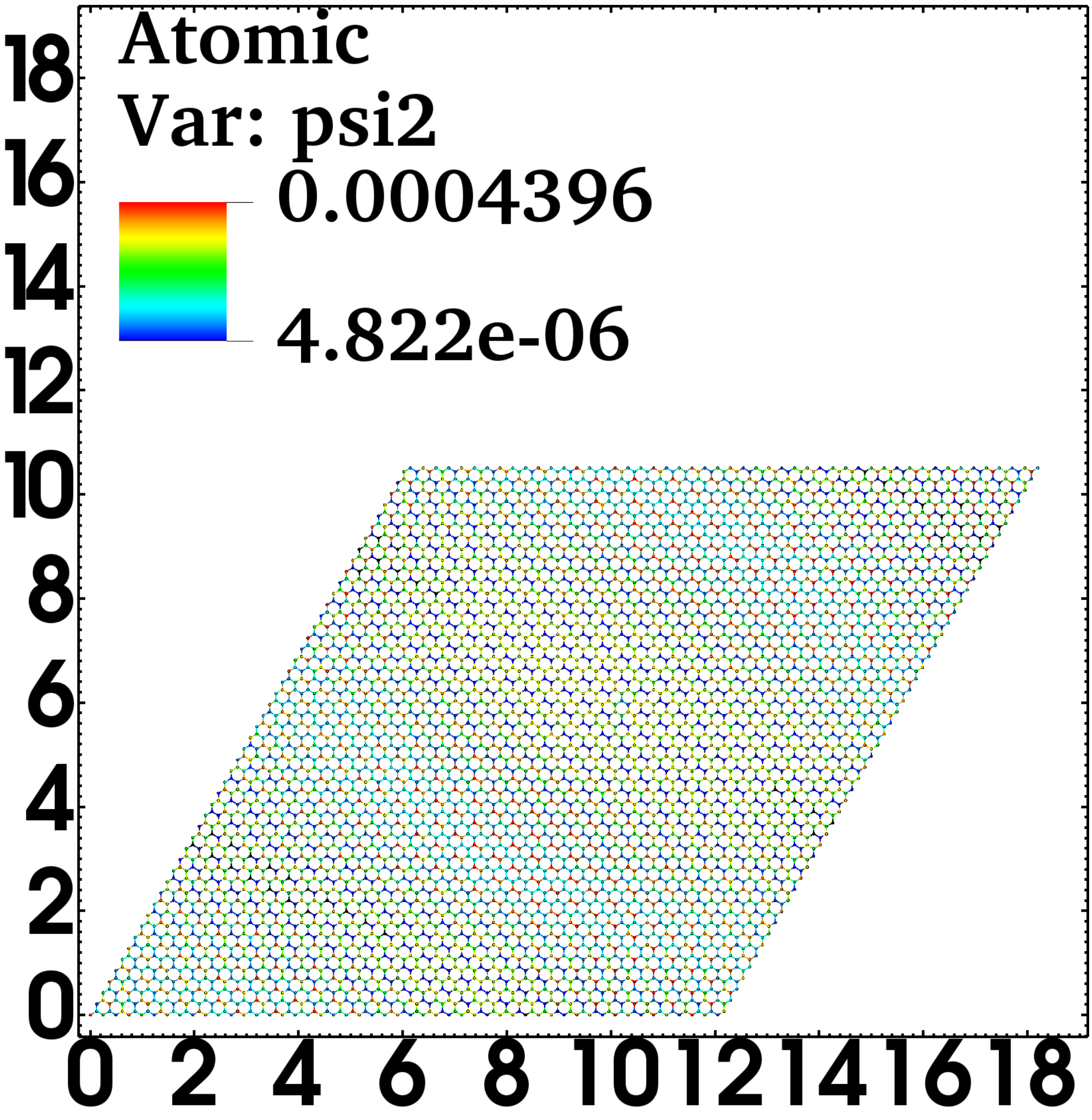

Figure 117. Spatially resolved electronic state $|\psi_3|^2$ Nitrogen impurity 50x50 graphene supercell

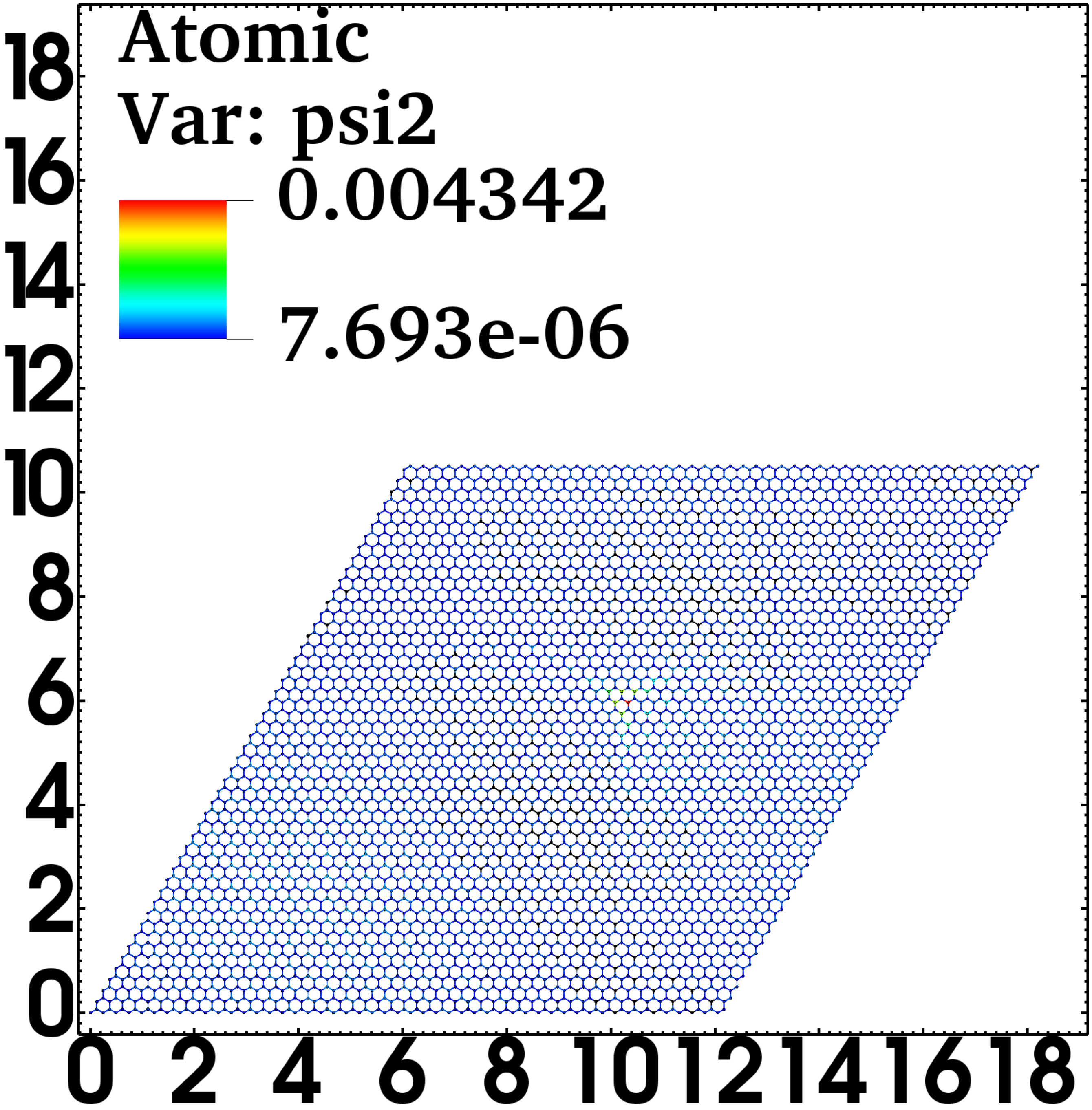

Figure 118. Spatially resolved electronic state $|\psi_4|^2$ Nitrogen impurity 50x50 graphene supercell

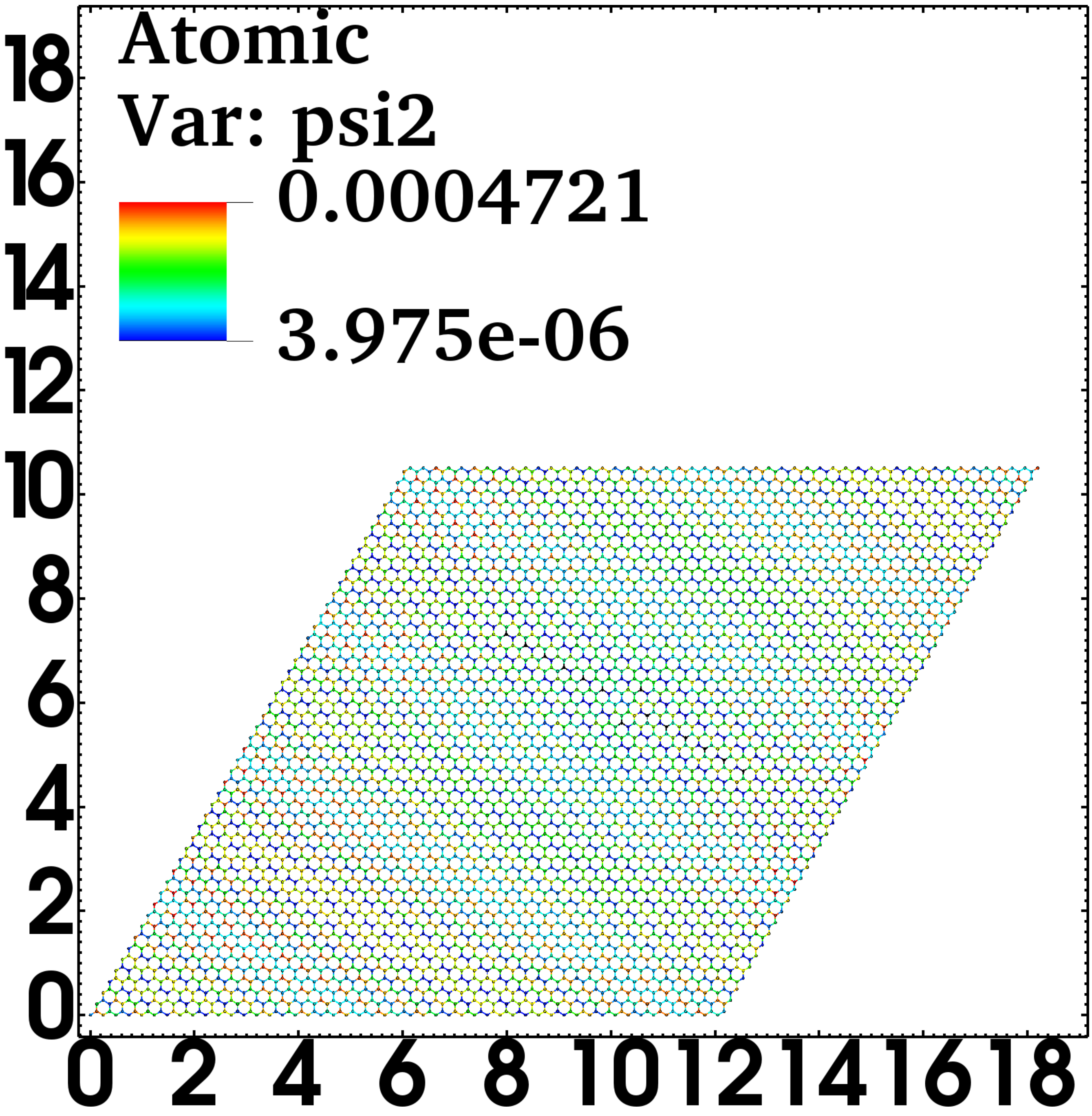

Figure 119. Spatially resolved electronic state $|\psi_5|^2$ Nitrogen impurity 50x50 graphene supercell

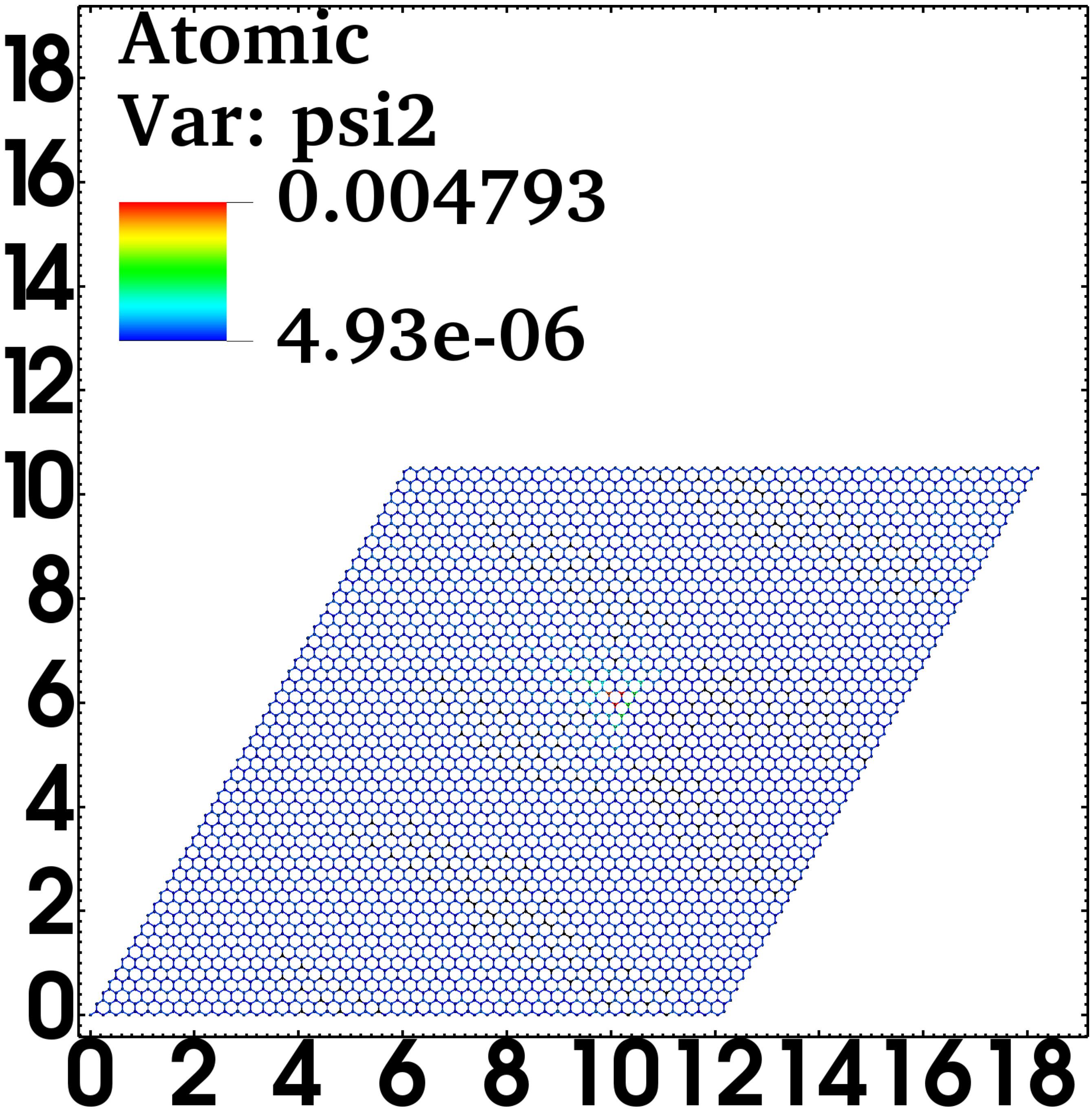

Figure 120. Spatially resolved electronic state $|\psi_6|^2$ Nitrogen impurity 50x50 graphene supercell

100x100 substitutional Nitrogen impurities doped corrugated graphene supercell

Next, we have doped the corrugated graphene sheet of 100x100 supercell with substitutional Nitrogen impurities with varying corrugation from 5 pm to 20 pm in the fig. 121 to fig. 132 investigate the combined effect of impurities atom and out of plane corrugation. We have substituted the 12 carbon atoms with the Nitrogen at the atomic location numbers 2972, 3565, 7129, 7418, 8127, 9101, 9178, 11032, 11692, 11707, 16757, 17155 in a graphene sheet constituting 20000 atoms carved out of 25nm x 40 nm flake.

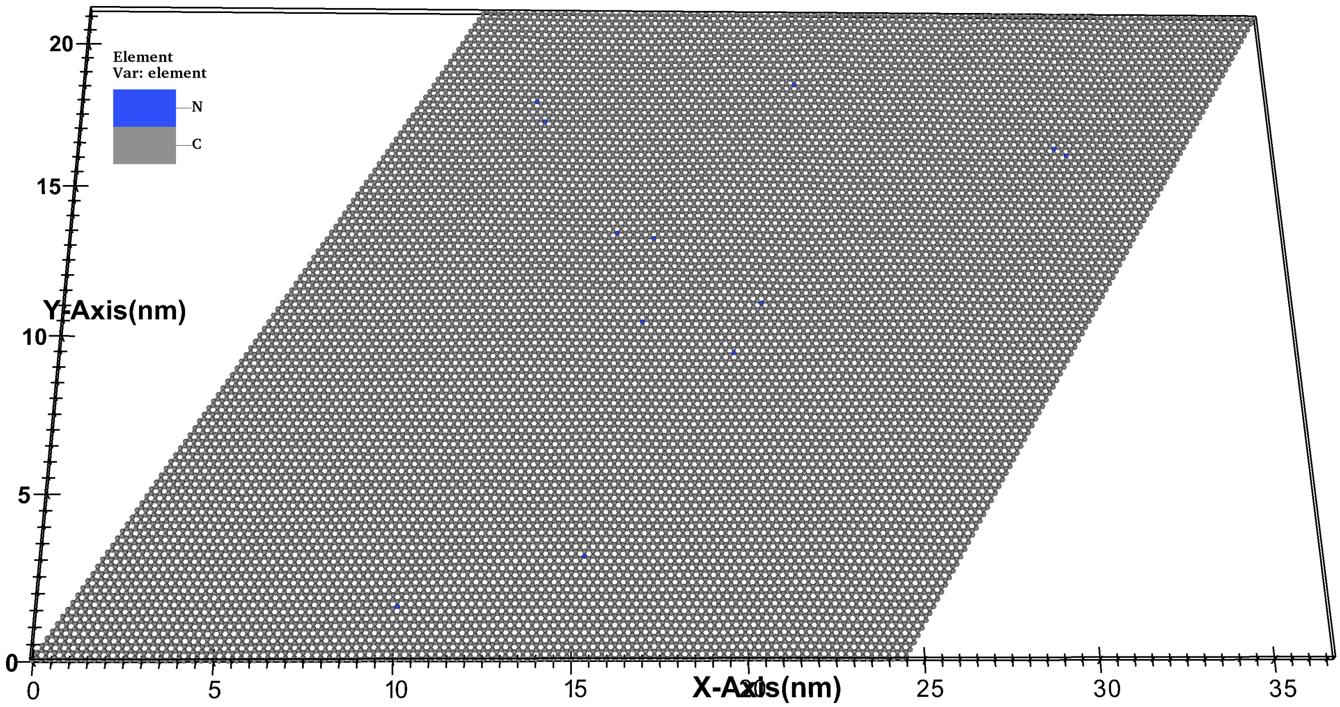

Figure 121. 12 substitutional Nitrogen impurities on corrugation-5pm

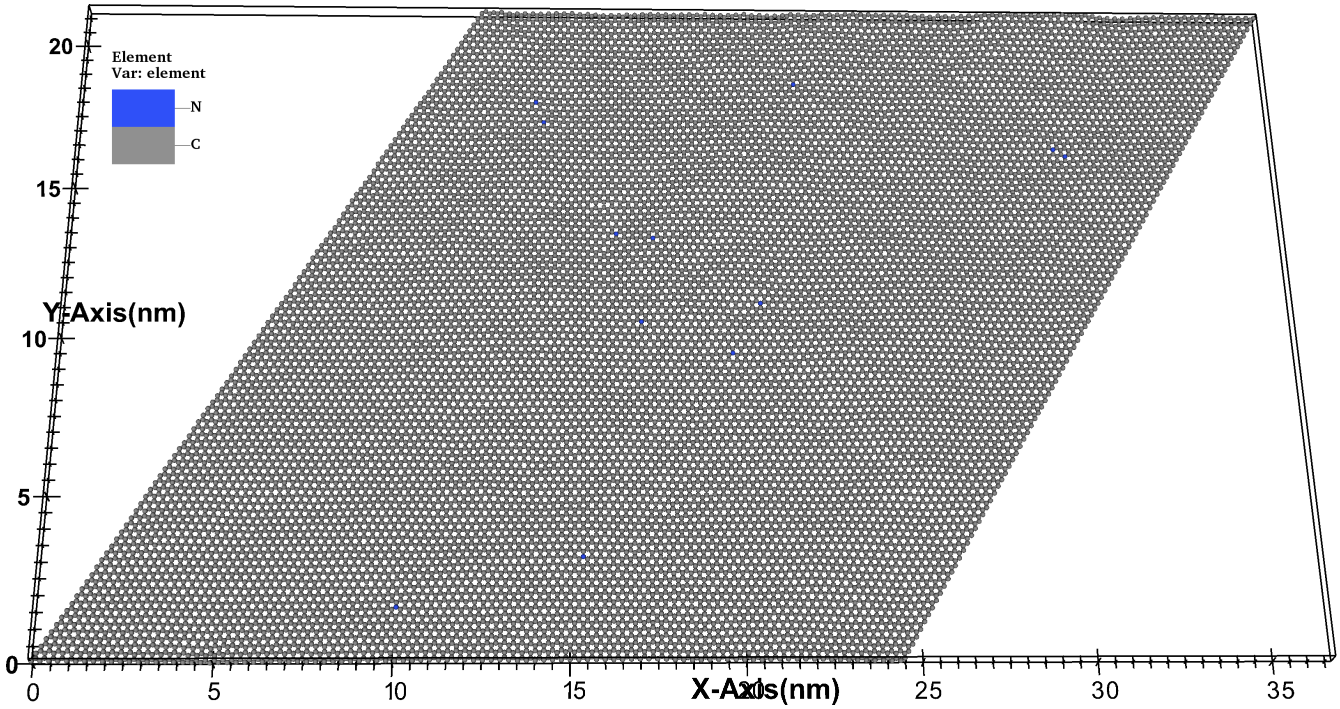

Figure 122. 12 substitutional Nitrogen impurities on corrugation-10pm

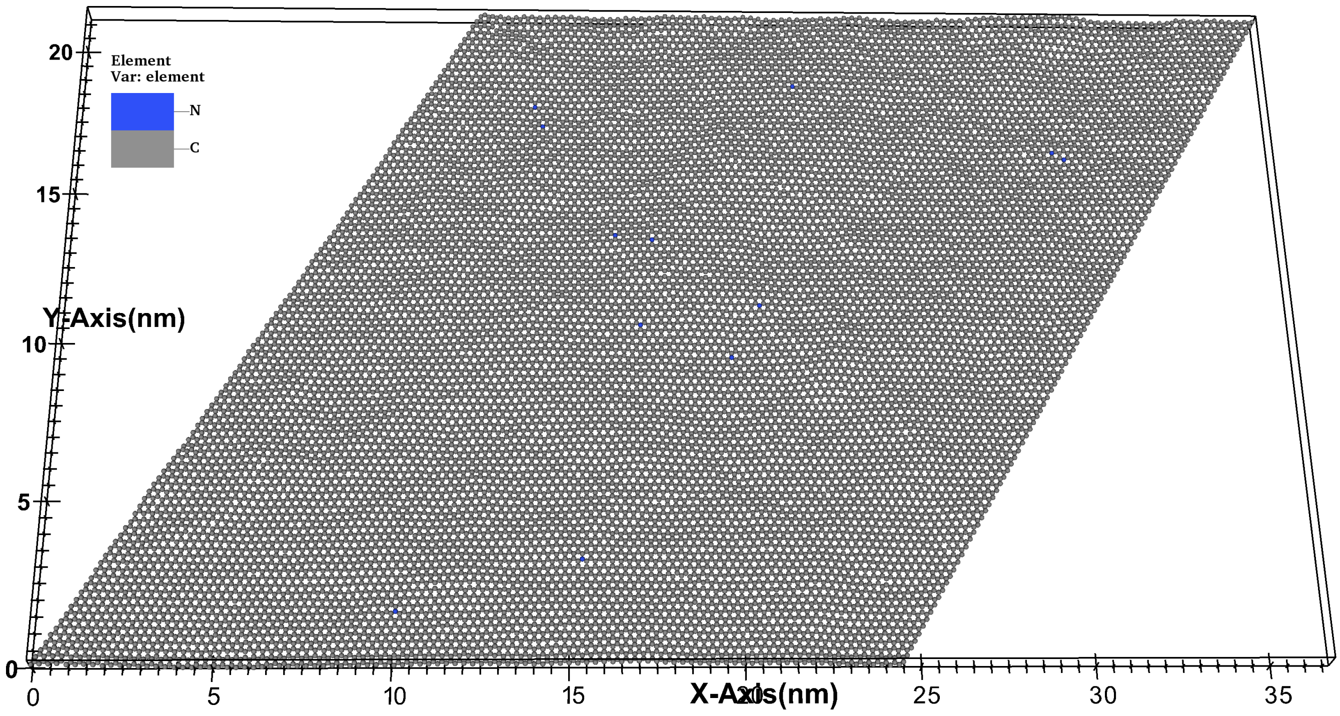

Figure 123. 12 substitutional Nitrogen impurities on corrugation-15pm

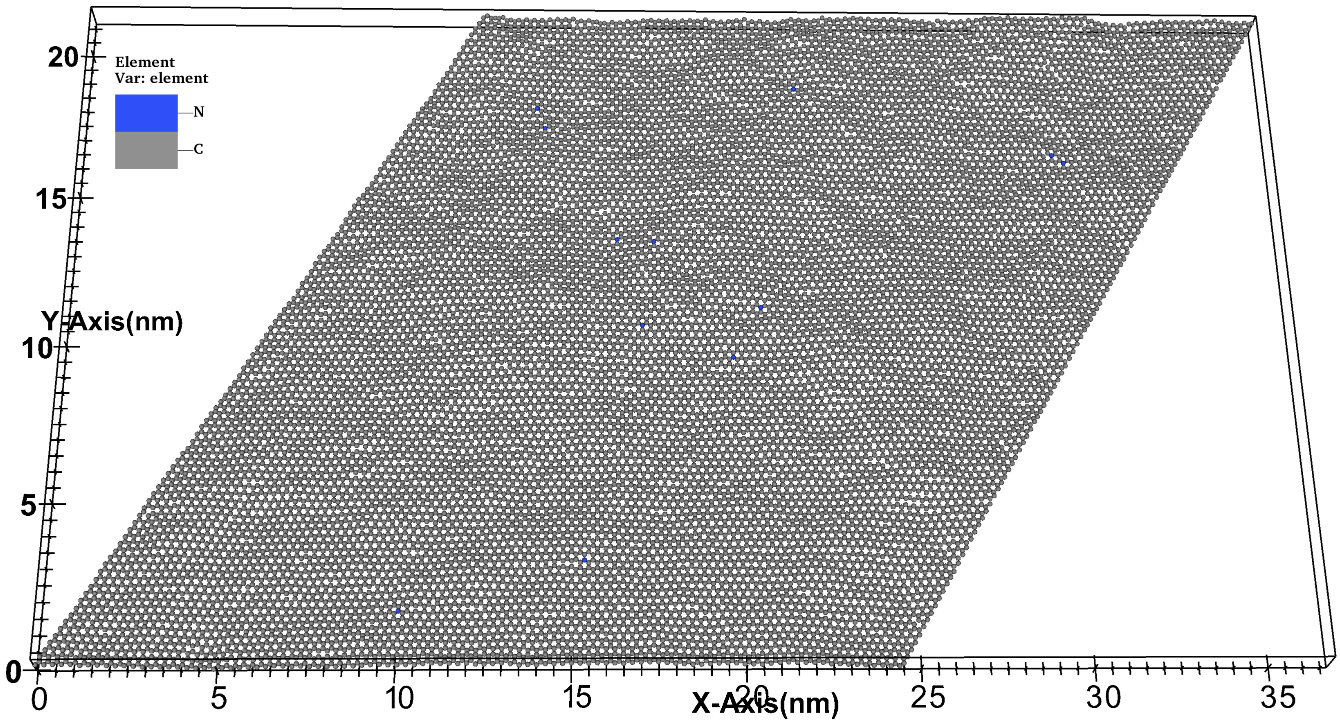

Figure 124. 12 substitutional Nitrogen impurities on corrugation-20pm

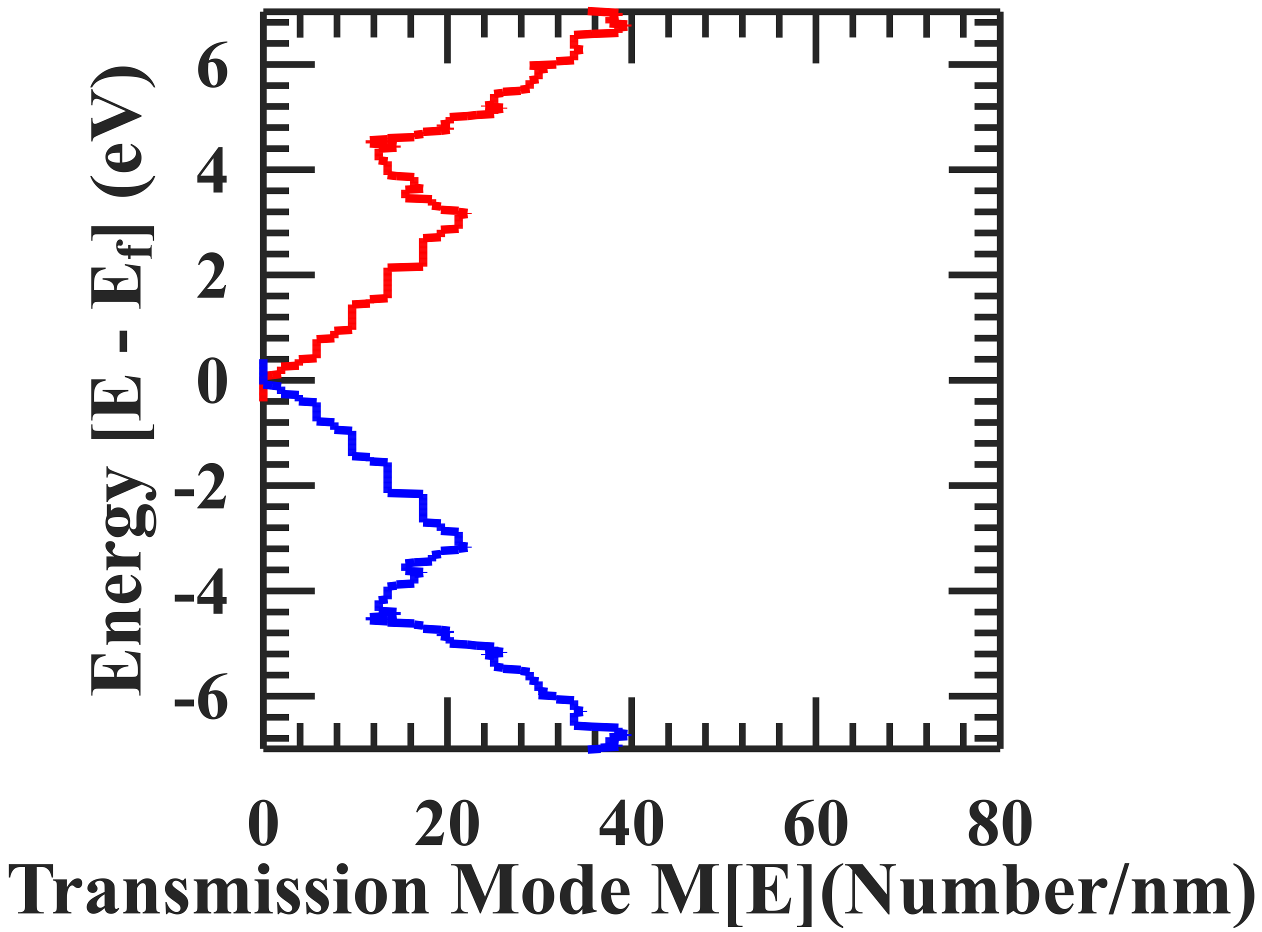

Figure 125. DOM M(E) in substitutional Nitrogen impurities on corrugation-5pm

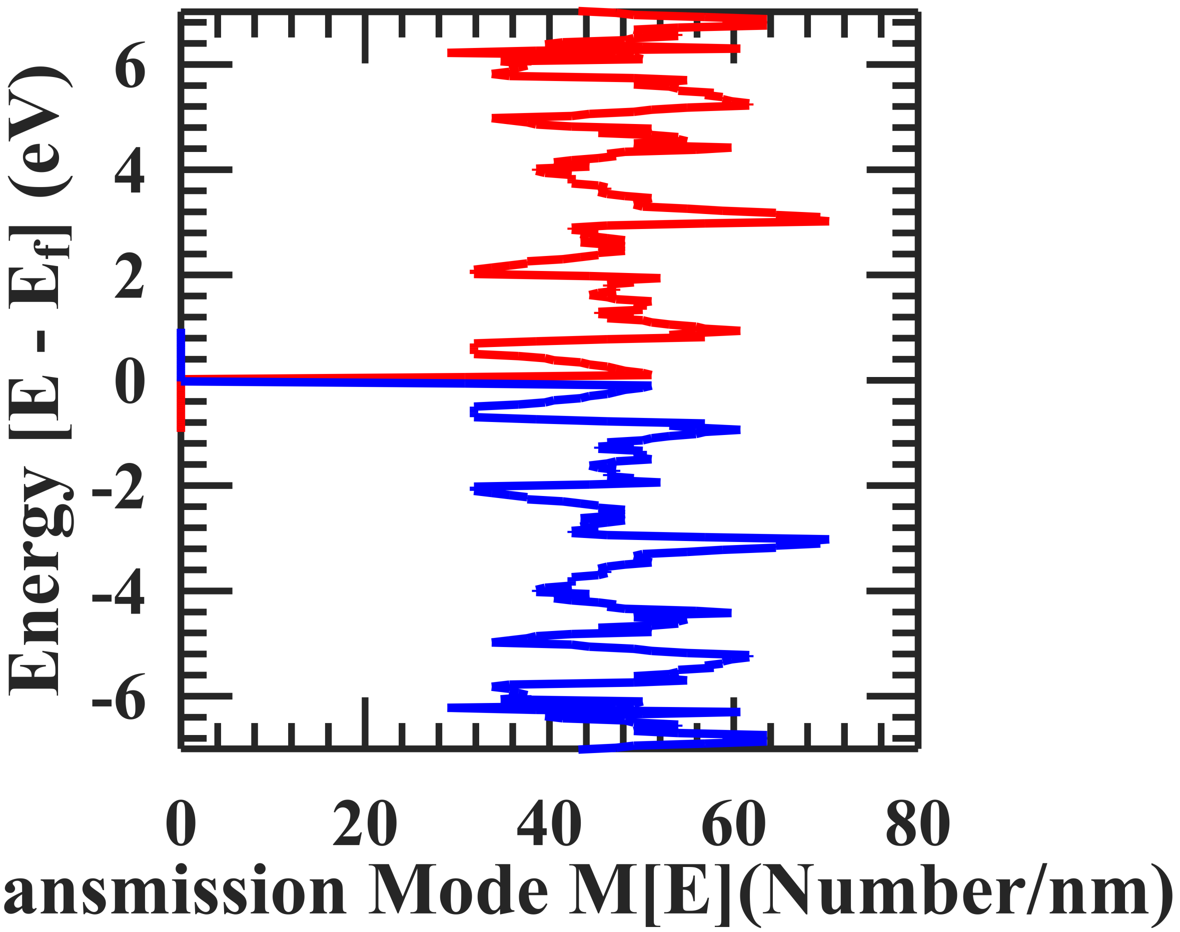

Figure 126. DOM M(E) in substitutional Nitrogen impurities on corrugation-10pm

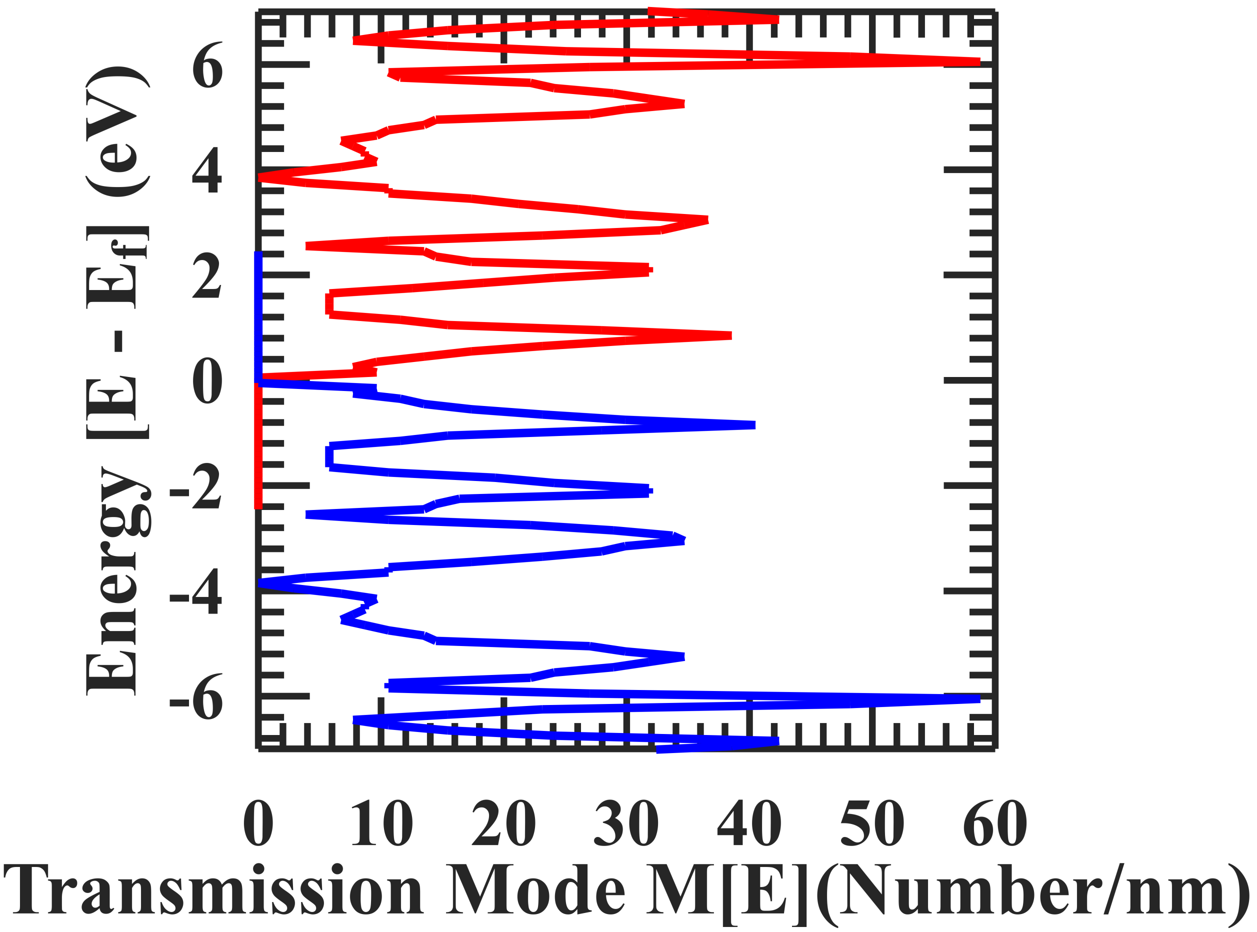

Figure 127. DOM M(E) in substitutional Nitrogen impurities on corrugation-15pm

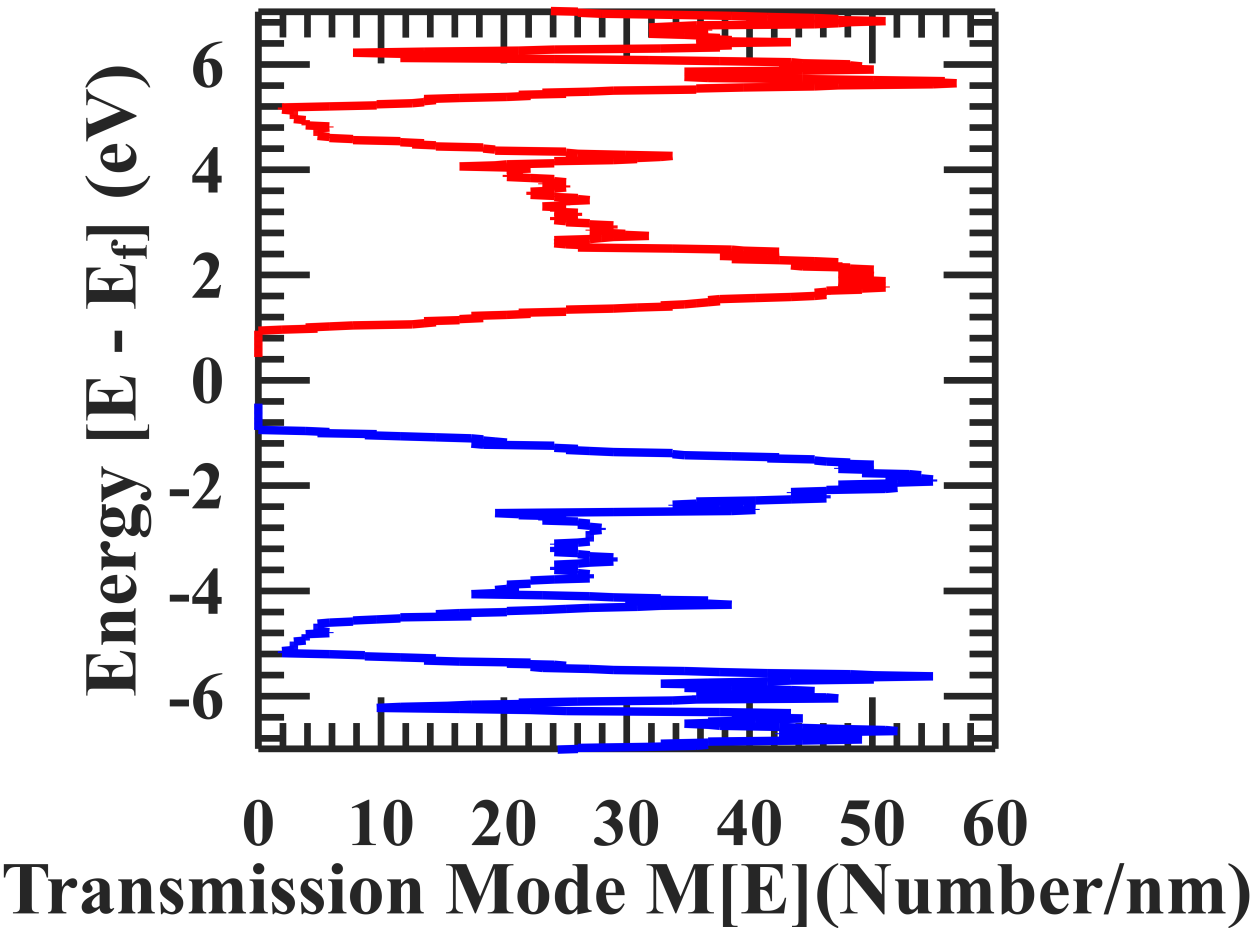

Figure 128. DOM M(E) in substitutional Nitrogen impurities on corrugation-20pm

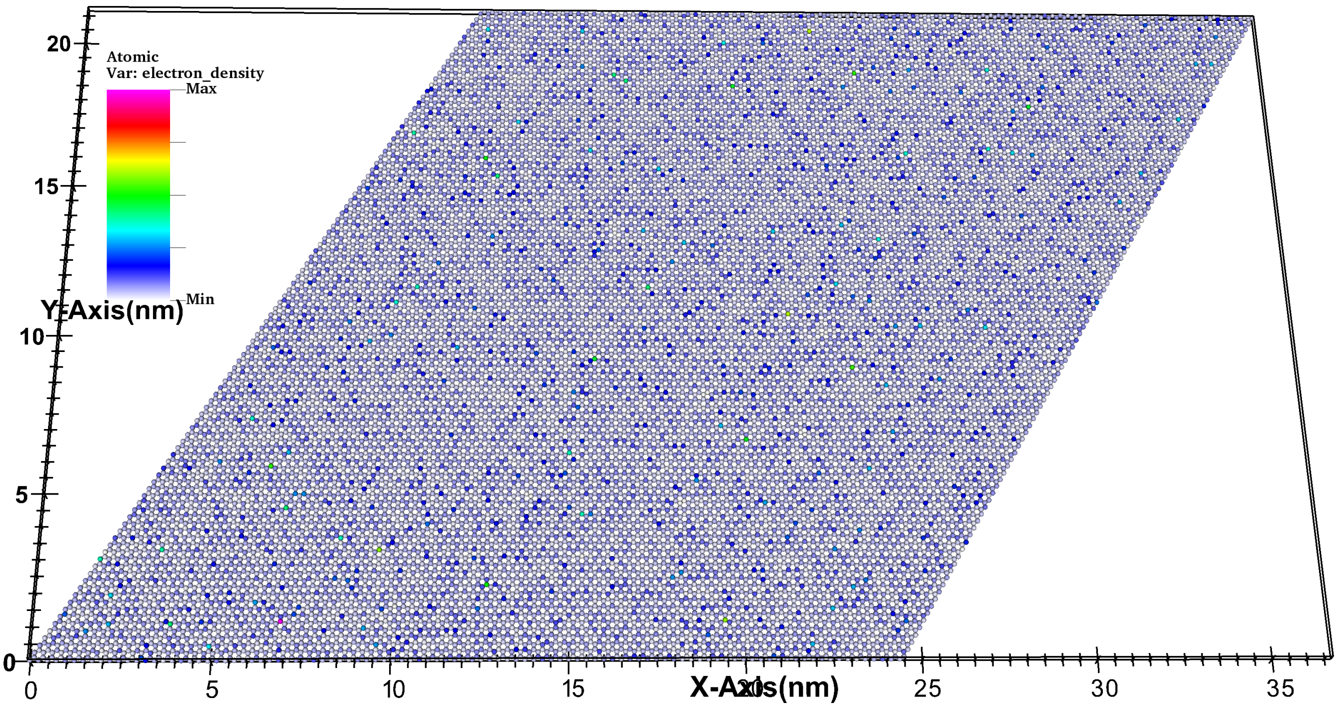

Figure 129. Electronic density of 12 substitutional Nitrogen impurities on corrugation-5pm

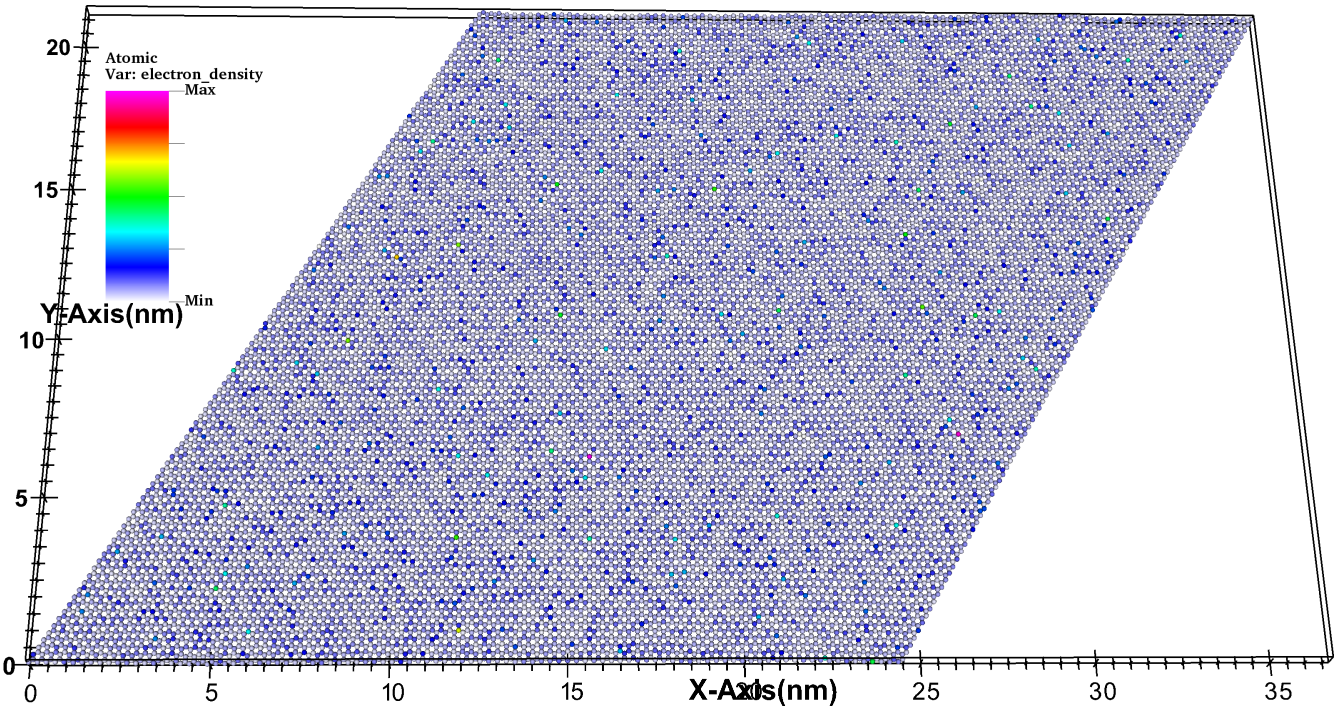

Figure 130. Electronic density of 12 substitutional Nitrogen impurities on corrugation-10pm

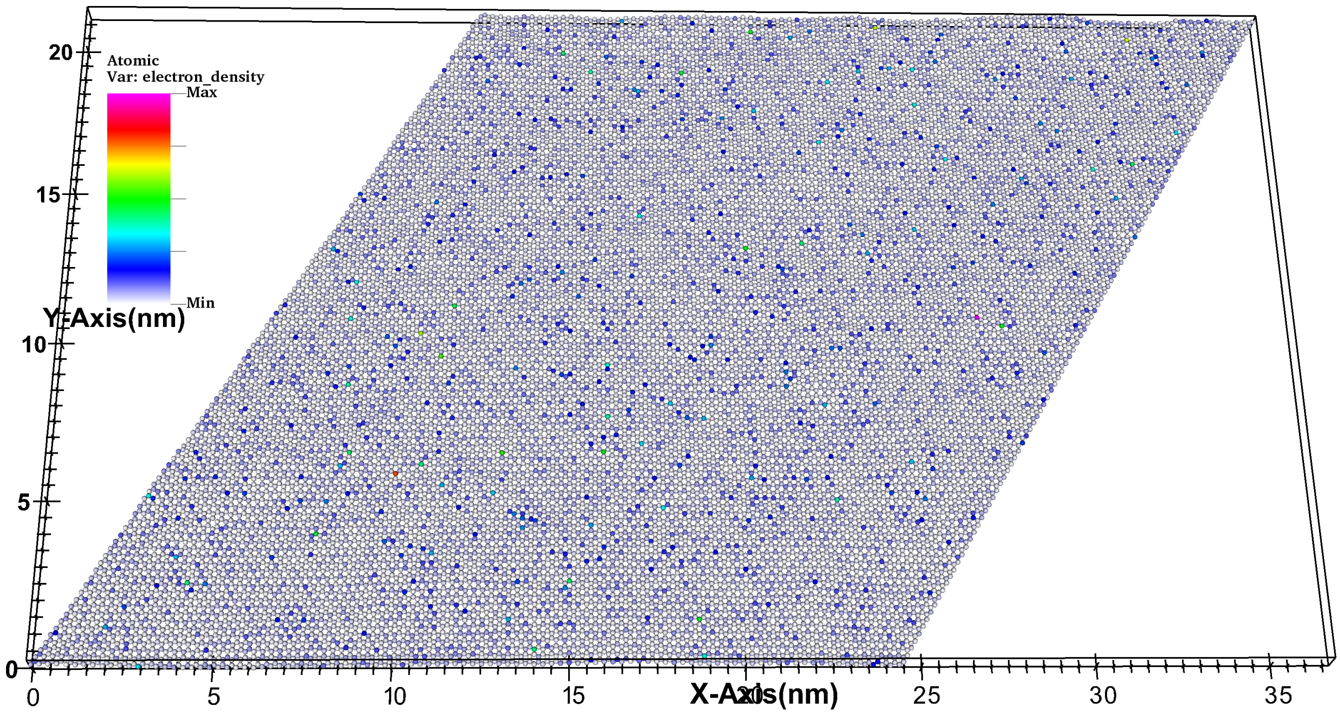

Figure 131. Electronic density of 12 substitutional Nitrogen impurities on corrugation-15pm

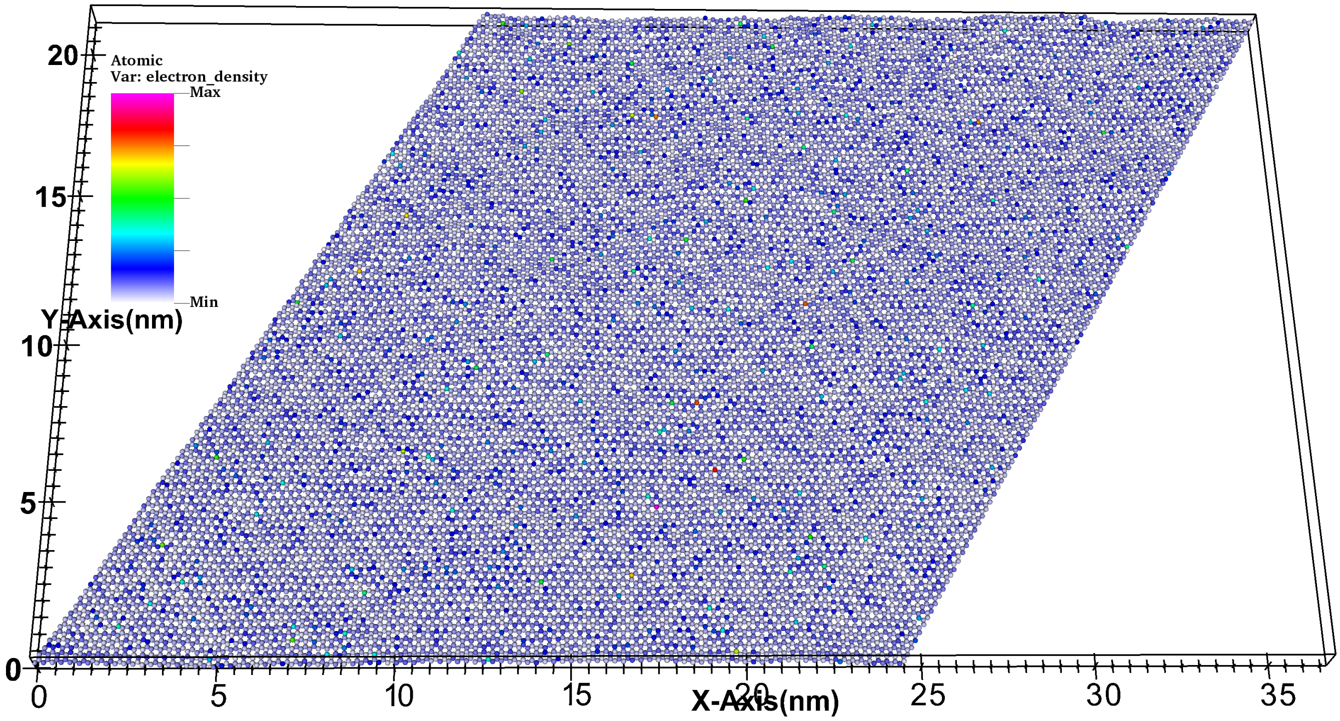

Figure 132. Electronic density of 12 substitutional Nitrogen impurities on corrugation-20pm

20x20 Phosphorus doped graphene supercell

In the Figure fig. 133 correspond to 20x20 graphene supercell flat structure with substitutional Phosphorus impurity, fig. 134 corresponding density of state, fig. 135 electronic band structure, fig. 136 density of mode, fig. 137 self-consistent Poisson potential due to Phosphorus impurity, fig. 138 electronic density, and fig. 139 to fig. 145 represents spatially resolved electronic orbital state probability amplitude $|\psi|^2$ for first seven eigenvalues of stationary solution of Schrödinger wave-equation from

$|\psi_0|^2$ to $|\psi_6|^2$ in the corresponding graphene supercell. In the simulated device, the primitive unit cell has two atoms per cell, and a total of 800 atoms are simulated by a finite element mesh of 3200 point Density of Mode size. The P-D tight-binding model contains three orbitals, namely carbon P_z, and carbon-hydrogen passivated D_{yz}, D_{xz} orbitals. Therefore total degree of free Density of Mode in hamiltonian is 2400 variable-sized. The K' and M' are high symmetric point that corresponds to the folded reduced BZ-zone of graphene supercell.

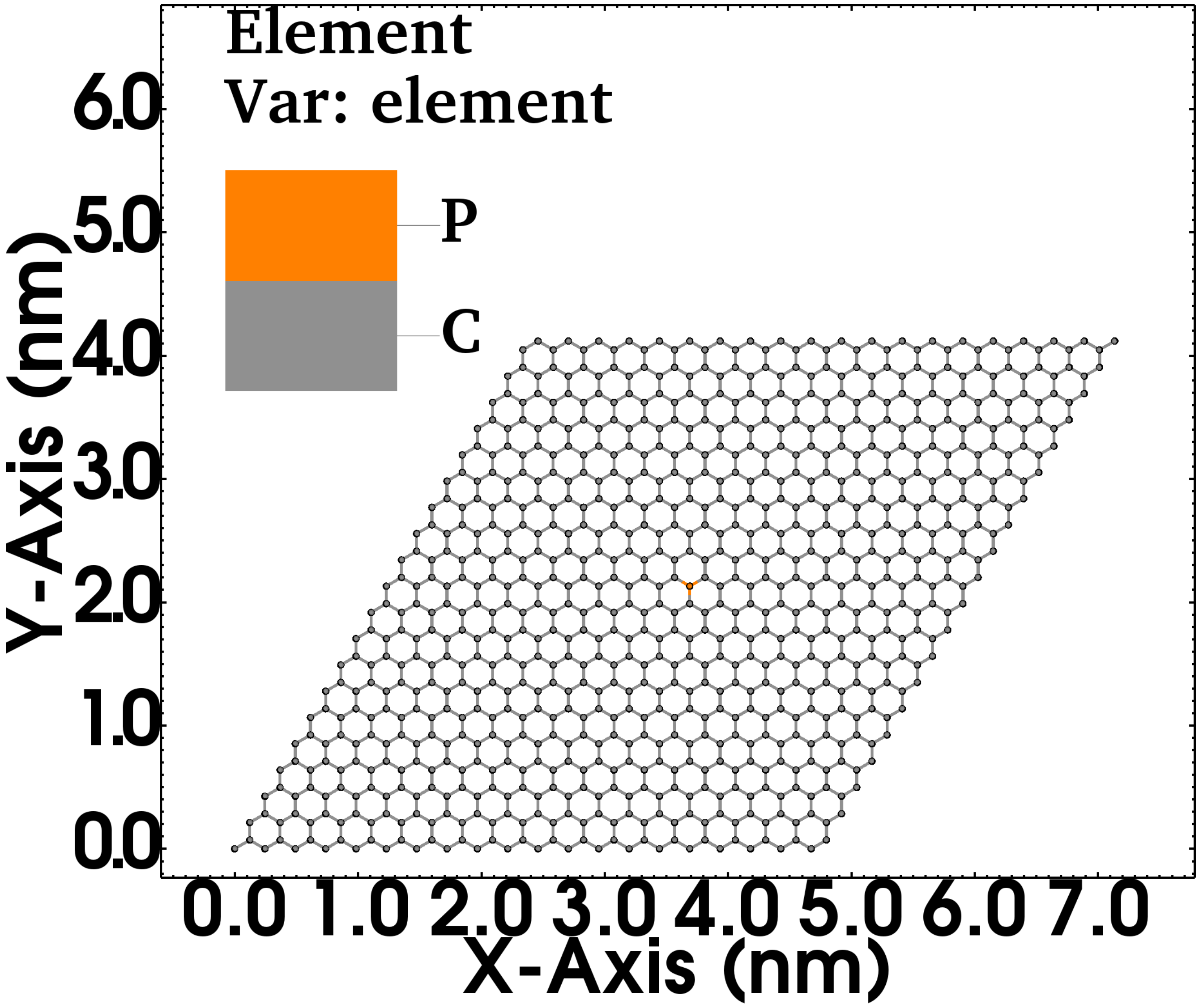

Figure 133. Phosphorus atom substitute 20x20 graphene supercell structure

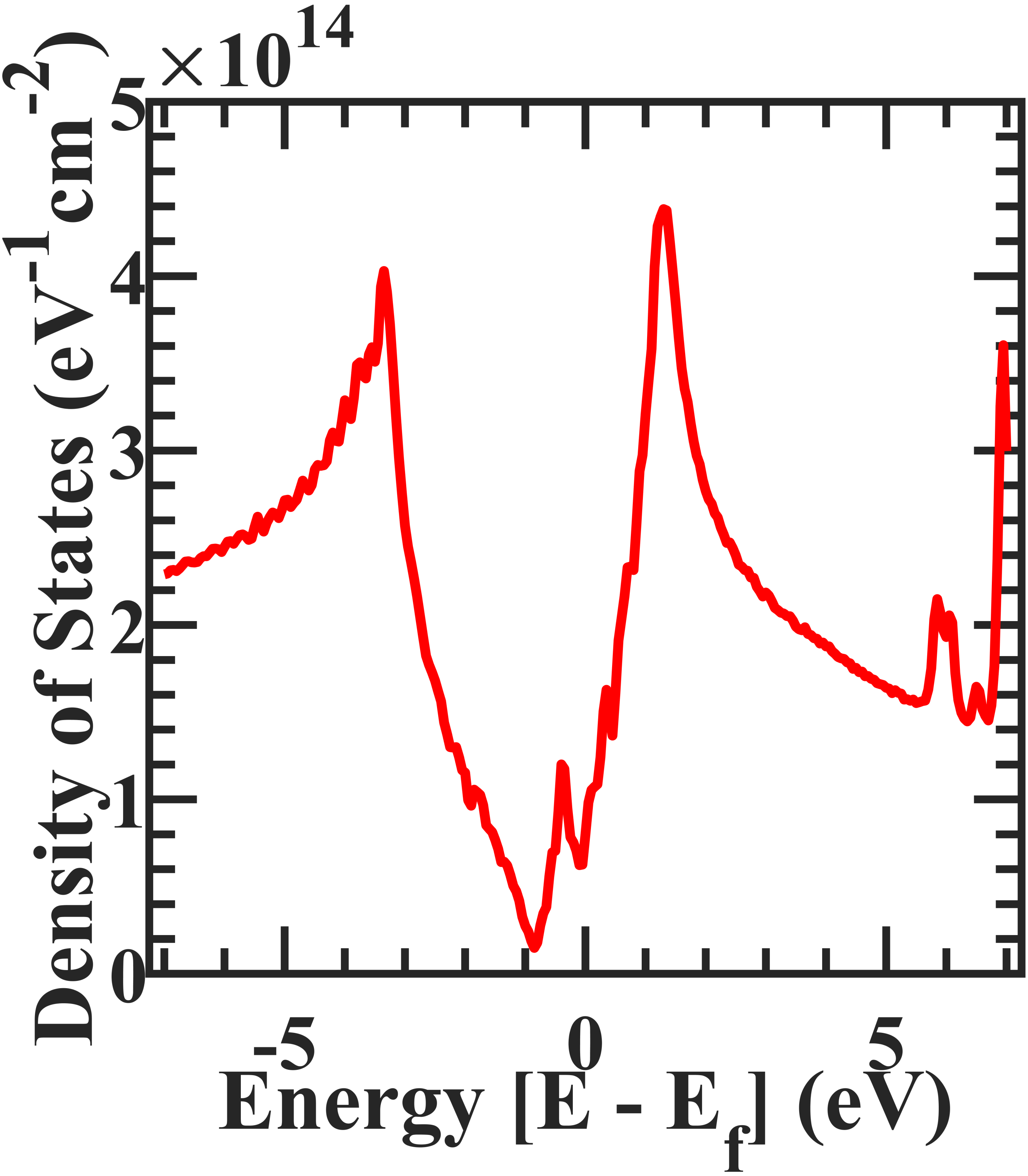

Figure 134. Density of state Phosphorus atom impurity 20x20 graphene supercell

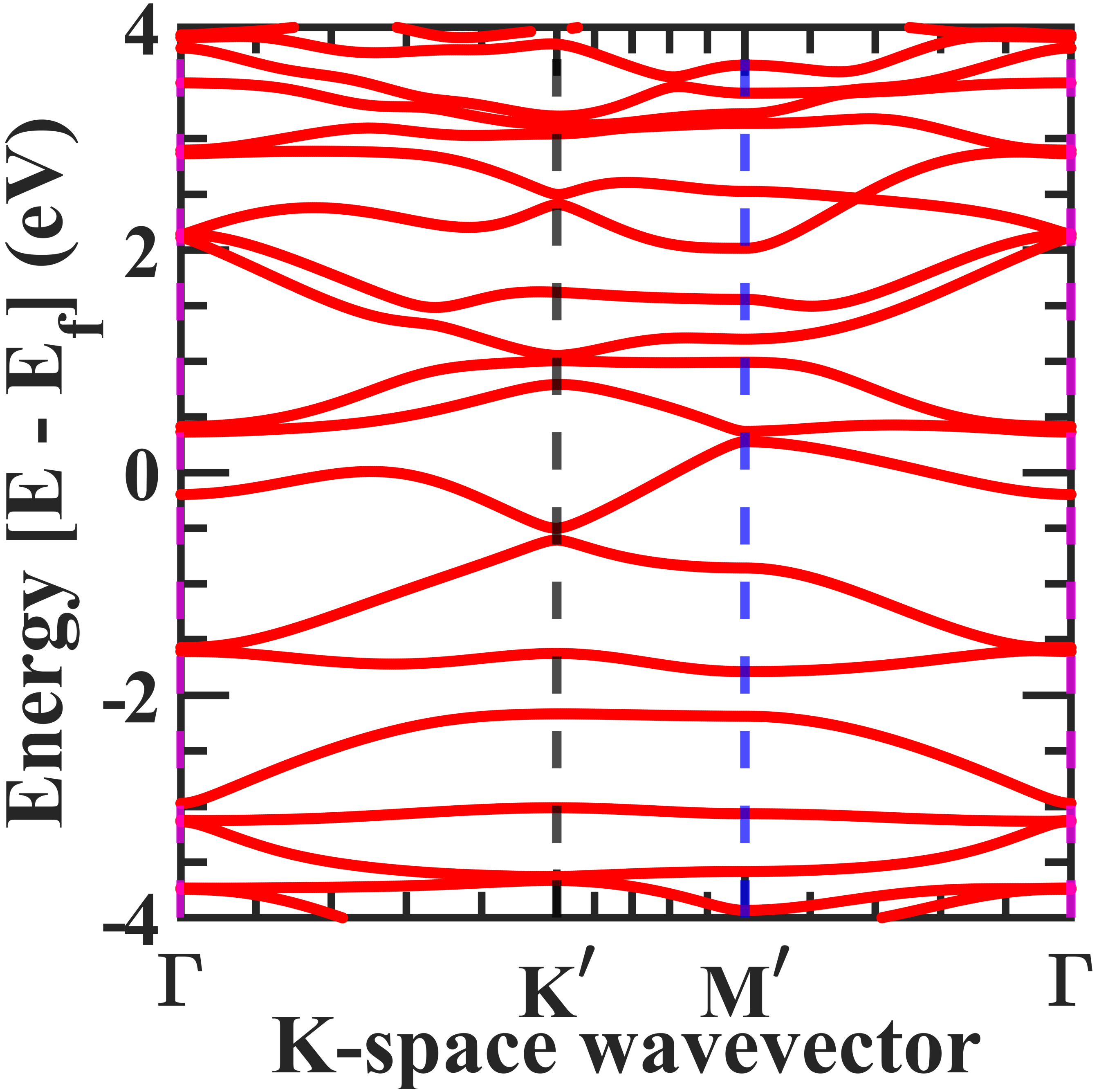

Figure 135. Band structure Phosphorus atom impurity 20x20 graphene supercell

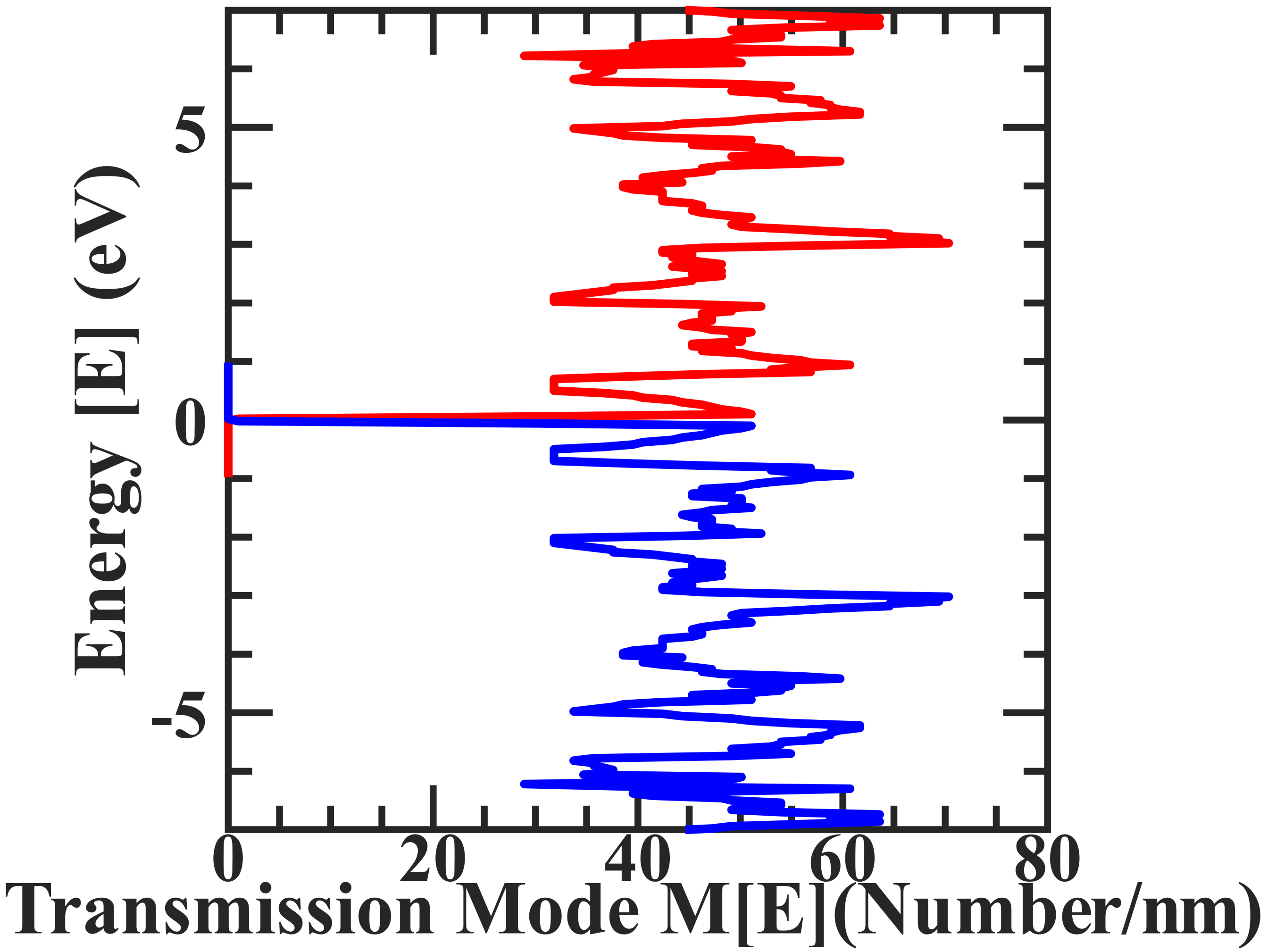

Figure 136. Density of mode Phosphorus atom impurity 20x20 graphene supercell

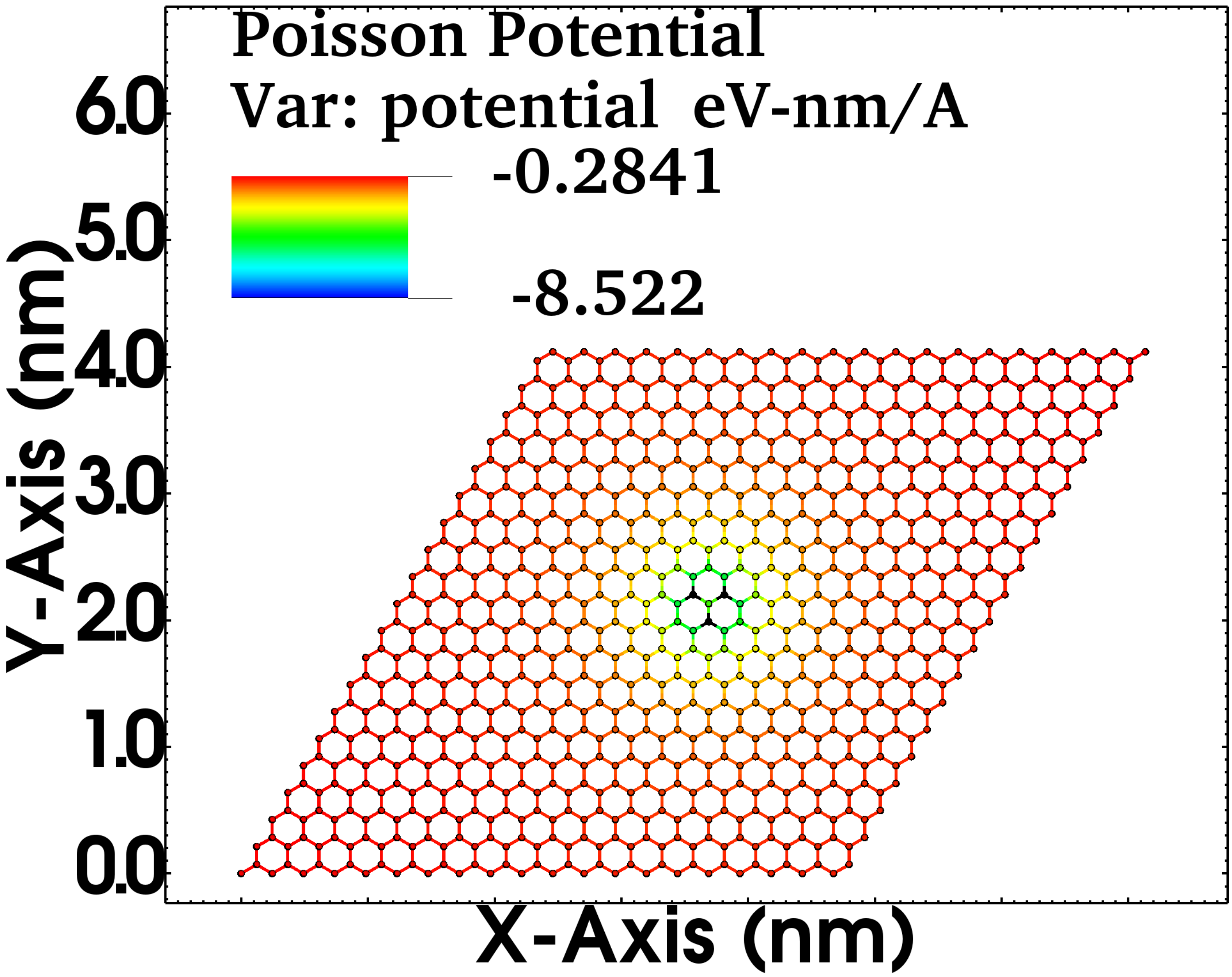

Figure 137. Self-consistent Poisson potential due to Phosphorus atom impurity 20x20 graphene supercell

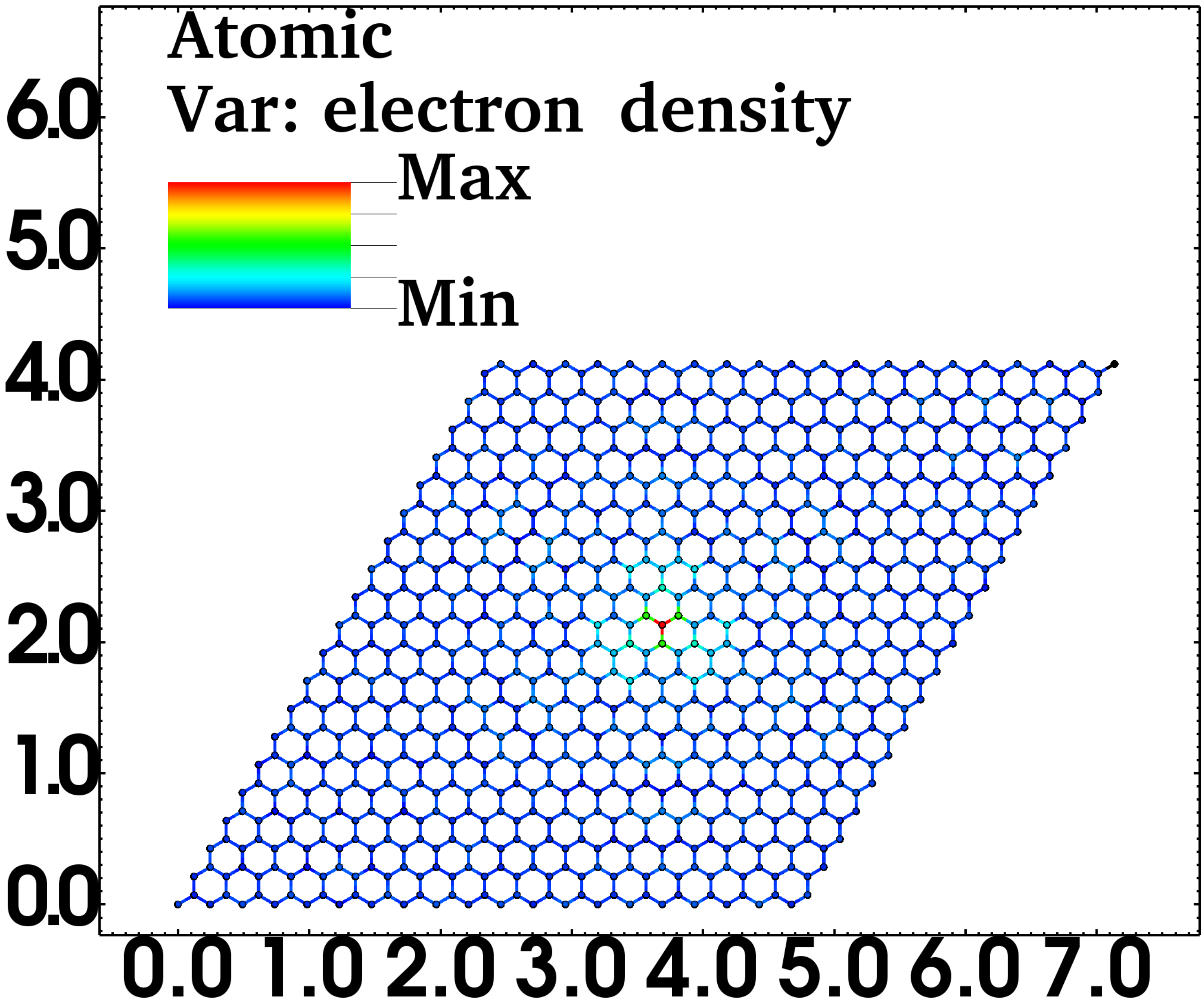

Figure 138. Electron density Phosphorus atom impurity 20x20 graphene supercell

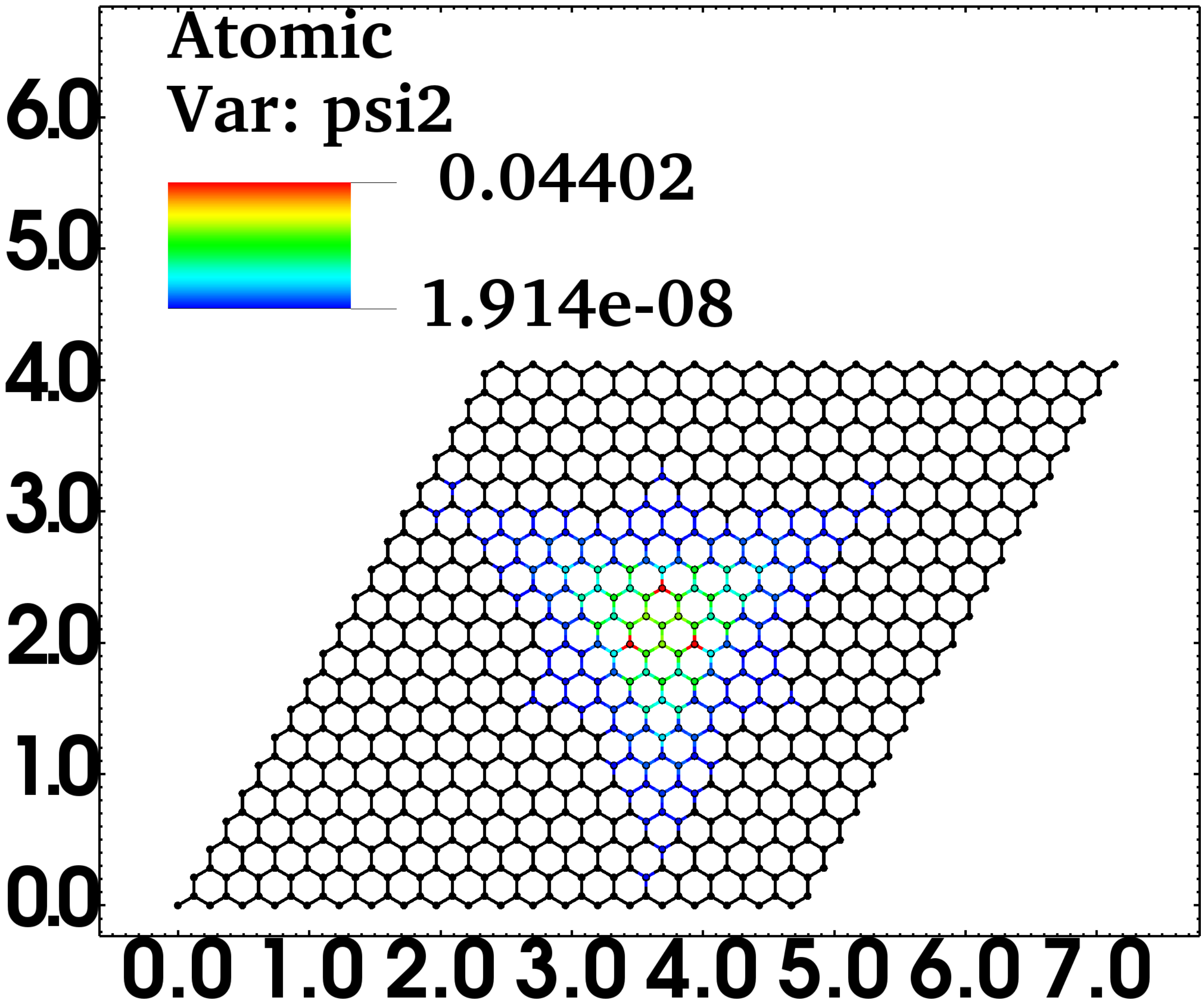

Figure 139. Spatially resolved electronic state $|\psi_0|^2$ Phosphorus atom impurity 20x20 graphene supercell

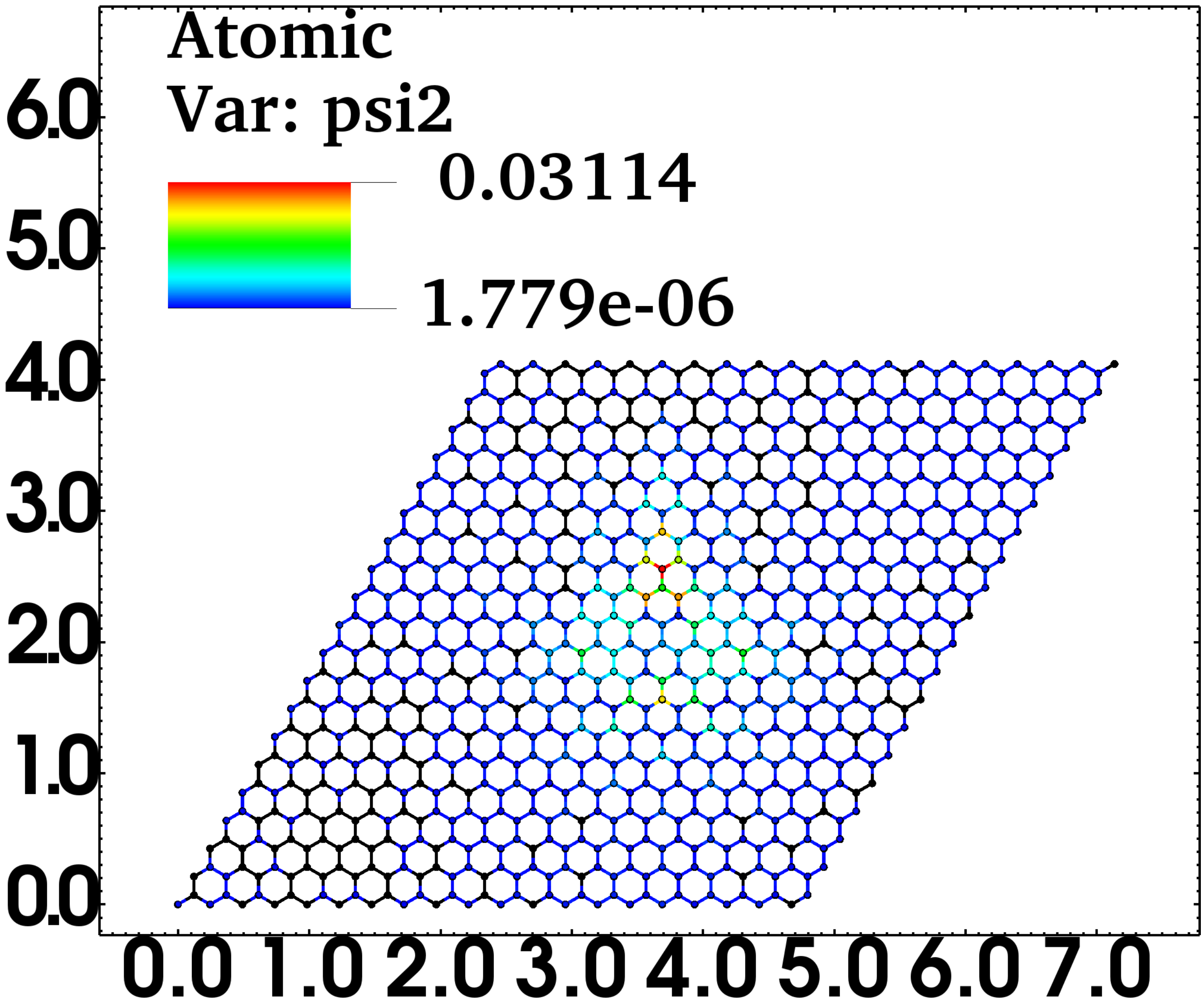

Figure 140. Spatially resolved electronic state $|\psi_1|^2$ Phosphorus atom impurity 20x20 graphene supercell

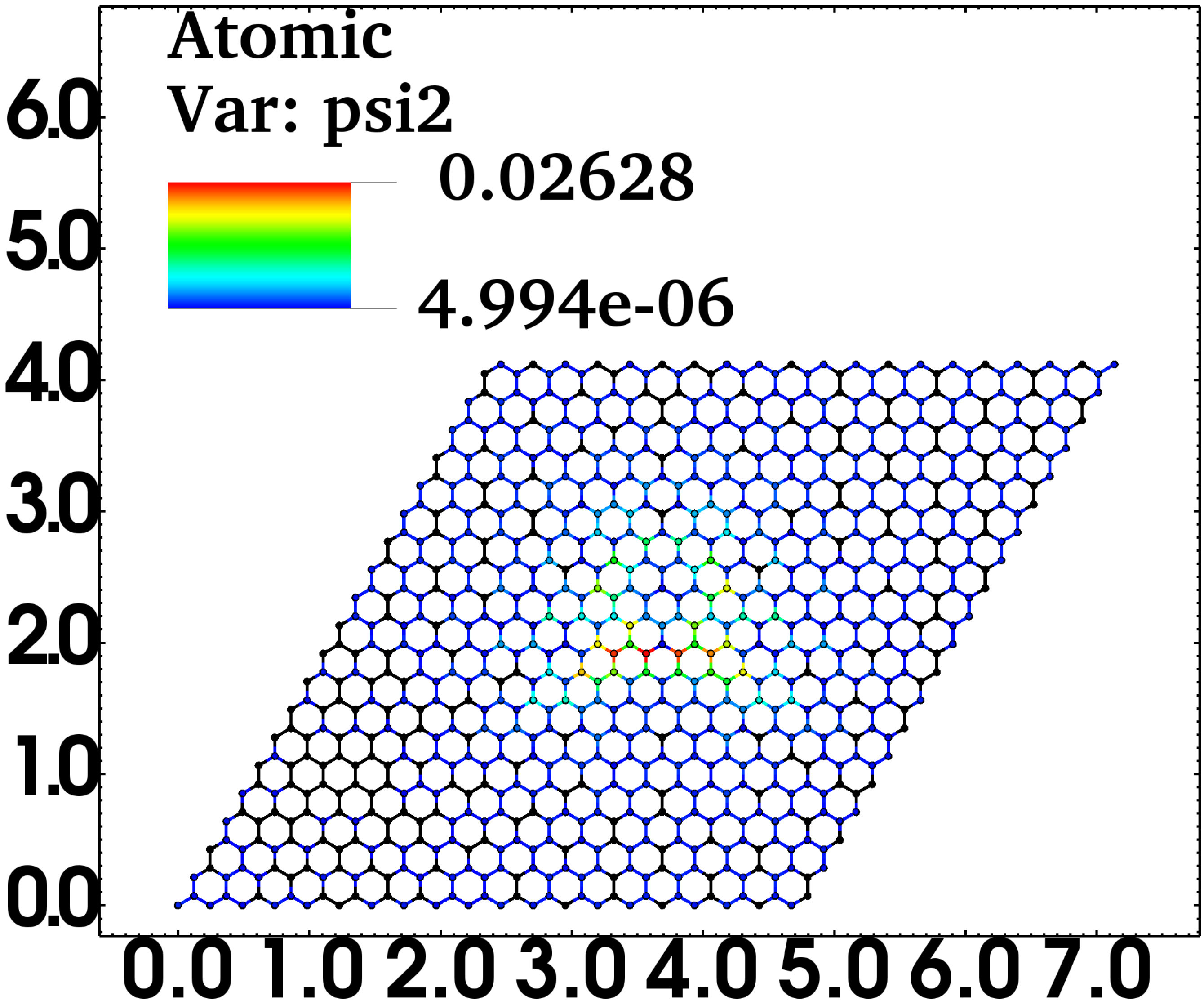

Figure 141. Spatially resolved electronic state $|\psi_2|^2$ Phosphorus atom impurity 20x20 graphene supercell

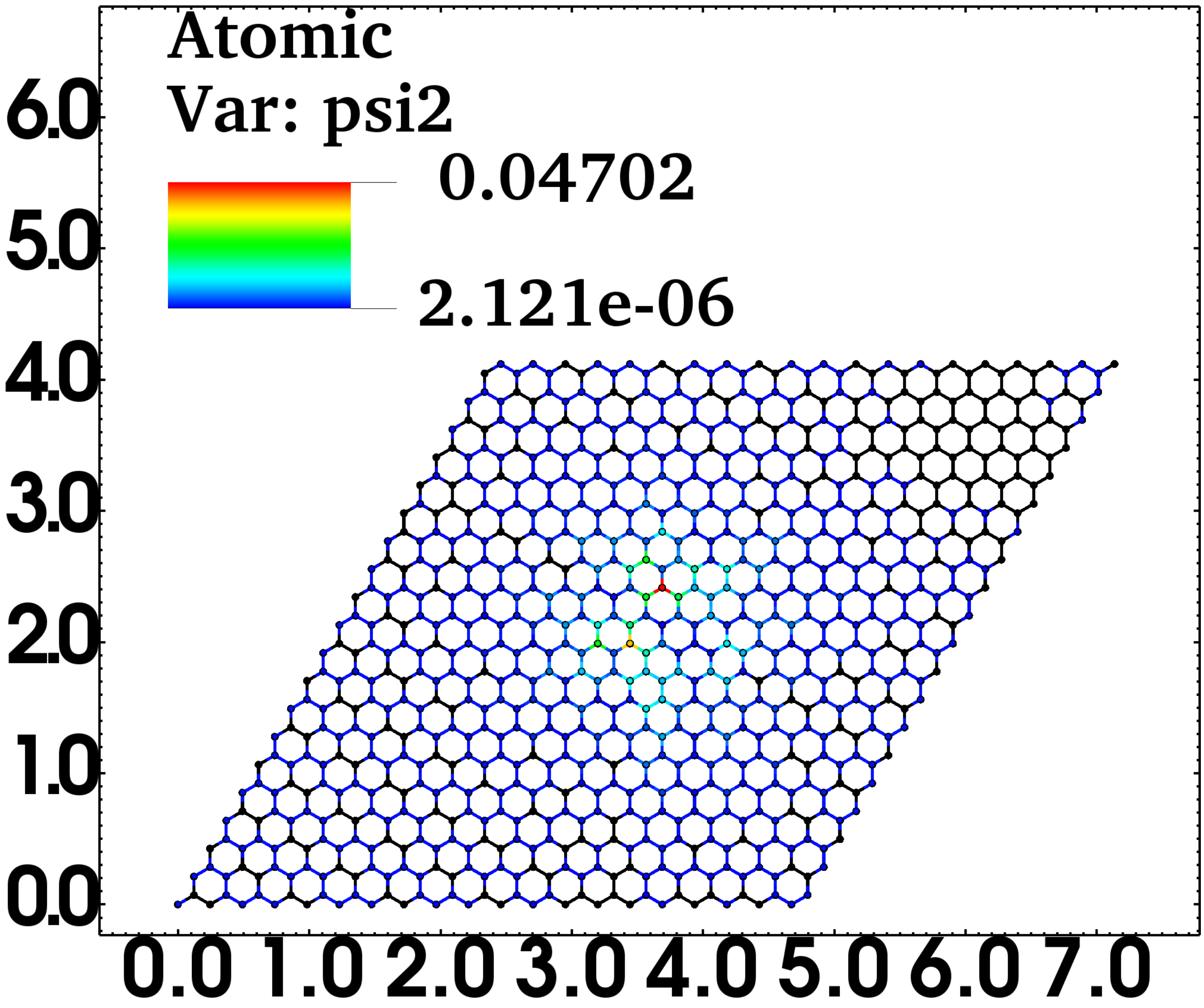

Figure 142. Spatially resolved electronic state $|\psi_3|^2$ Phosphorus atom impurity 20x20 graphene supercell

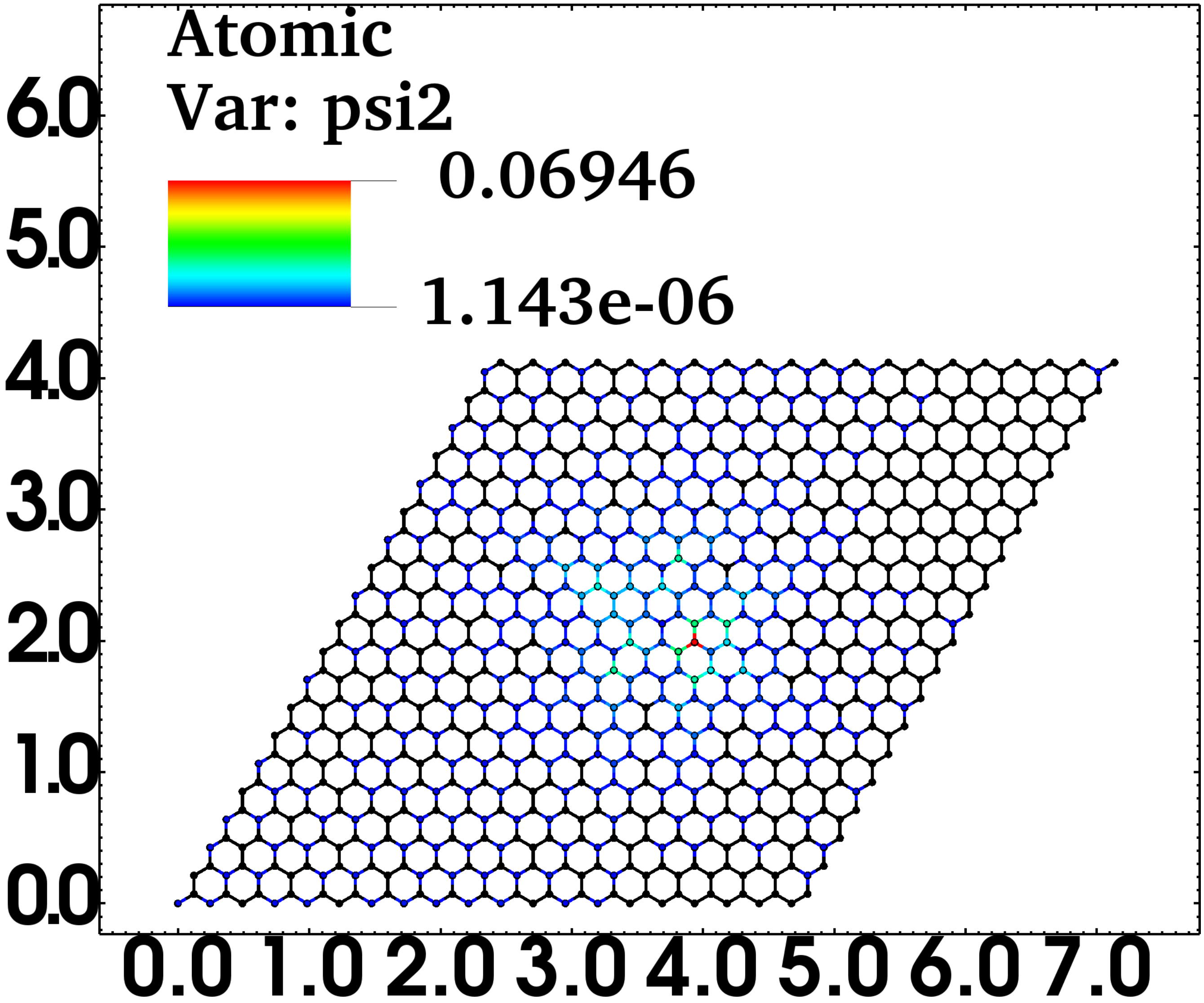

Figure 143. Spatially resolved electronic state $|\psi_4|^2$ Phosphorus atom impurity 20x20 graphene supercell

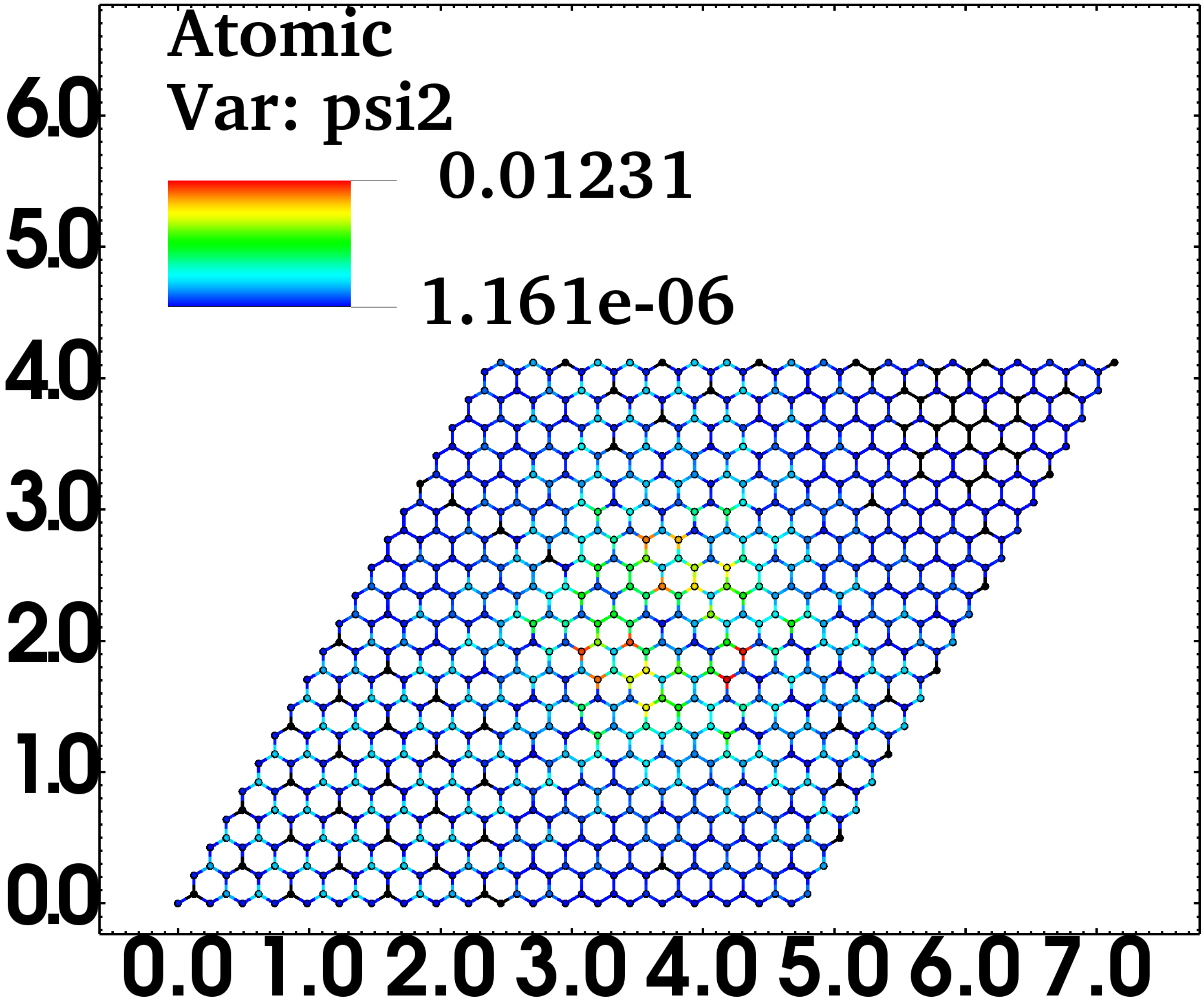

Figure 144. Spatially resolved electronic state $|\psi_5|^2$ Phosphorus atom impurity 20x20 graphene supercell

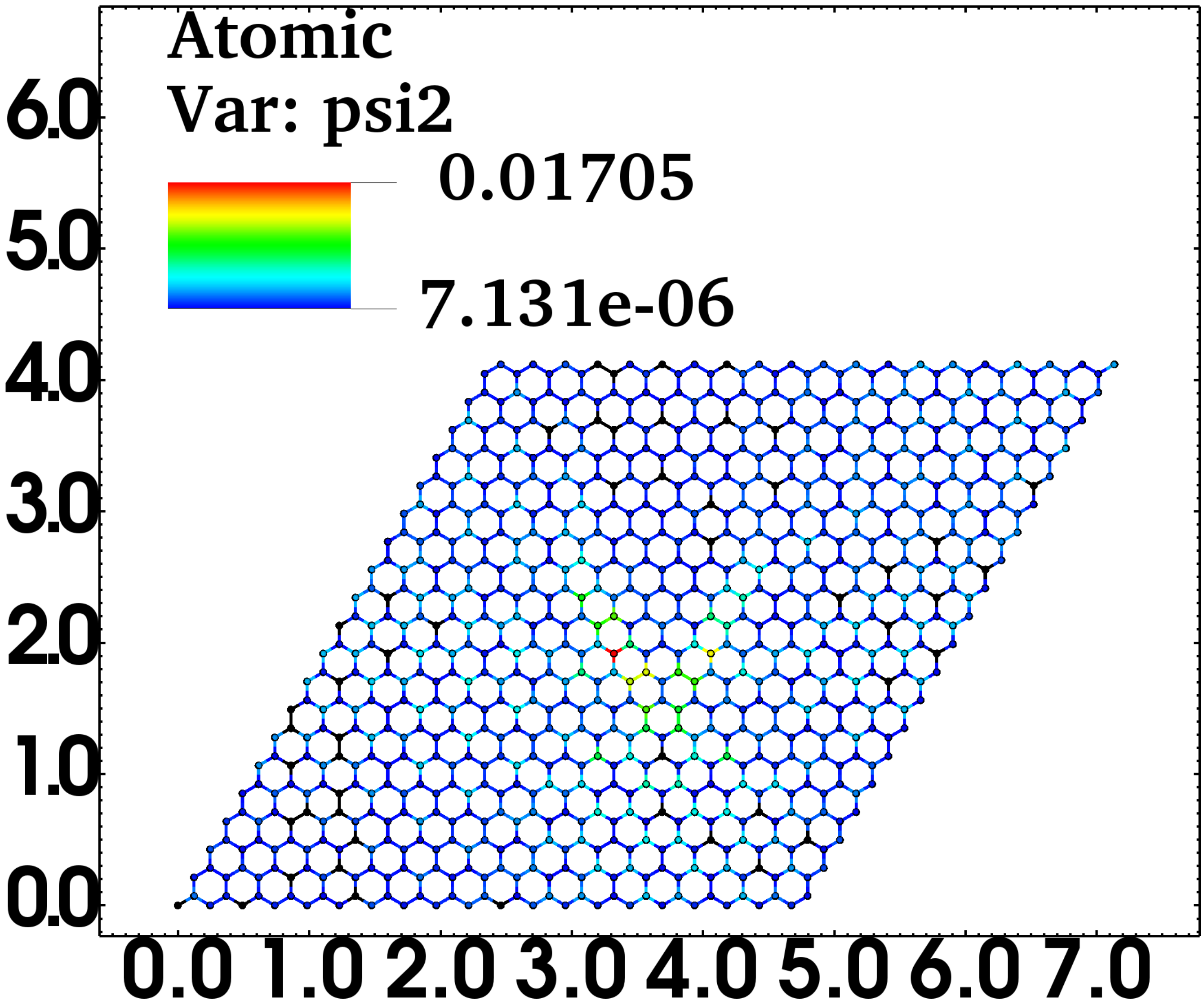

Figure 145. Spatially resolved electronic state $|\psi_6|^2$ Phosphorus atom impurity 20x20 graphene supercell

50x50 Phosphorus doped graphene supercell

In the Figure fig. 146 correspond to 50x50 graphene supercell flat structure with substitutional Phosphorus impurity, fig. 147 corresponding density of state, fig. 148 electronic band structure, fig. 149 density of mode, fig. 150 self-consistent Poisson potential due to Phosphorus impurity, fig. 151 electronic density, and fig. 152 to fig. 158 represents spatially resolved electronic orbital state probability amplitude $|\psi|^2$ for first seven eigenvalues of stationary solution of Schrödinger wave-equation from $|\psi_0|^2$ to $|\psi_6|^2$ in the corresponding graphene supercell. In the simulated device, the primitive unit cell has two atoms per cell, and a total of 5000 atoms are simulated by a finite element mesh of 20000 point Density of Mode size. The P-D tight-binding model contains three orbitals, namely carbon P_z, and carbon-hydrogen passivated D_{yz}, D_{xz} orbitals. Therefore total degree of free density of Mode in hamiltonian is 15000 variable-sized. The K' and M' are high symmetric point that corresponds to the folded reduced BZ-zone of graphene supercell.

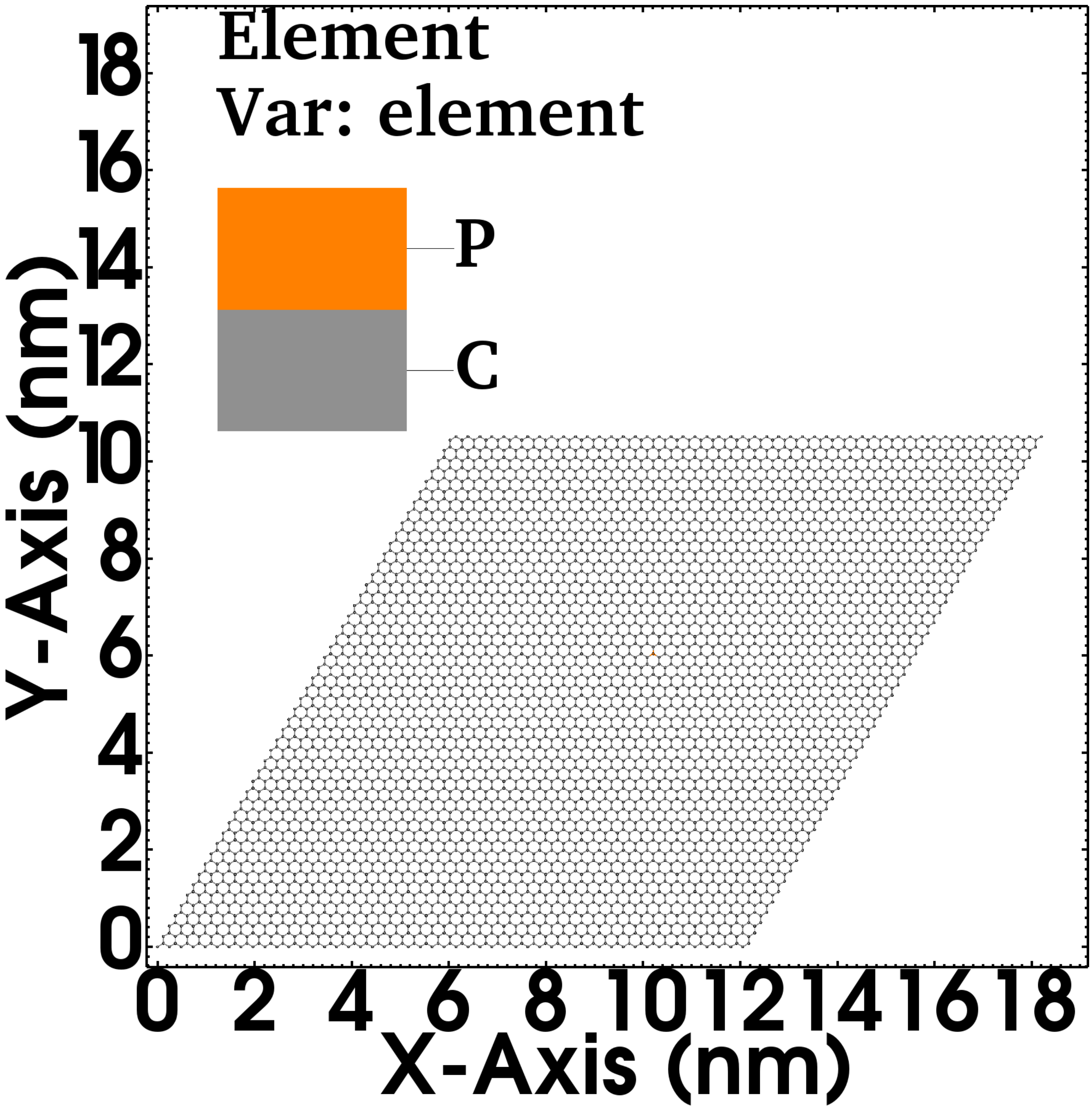

Figure 146. Phosphorus atom substitute 50x50 graphene supercell structure

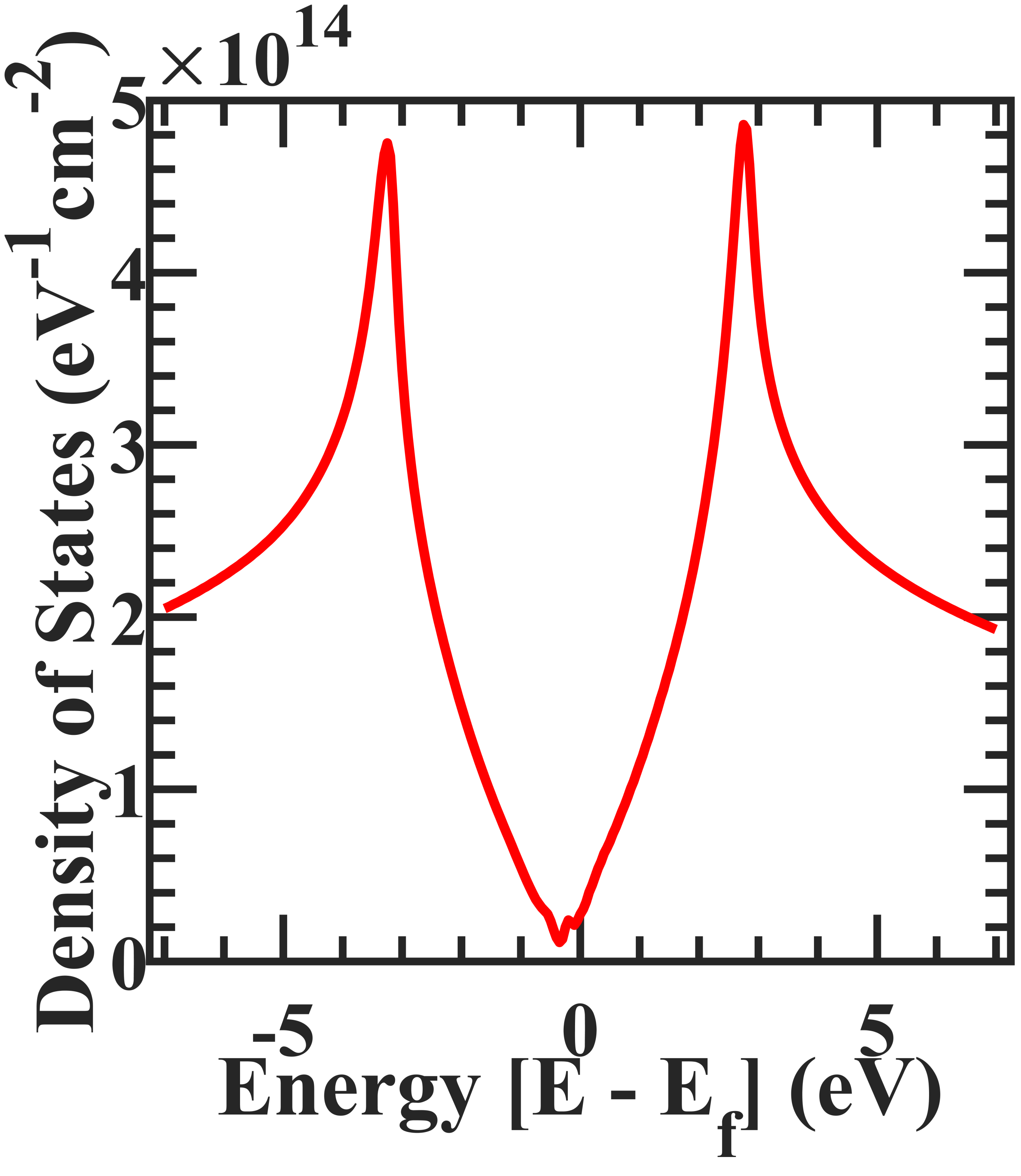

Figure 147. Density of state Phosphorus atom impurity 50x50 graphene supercell

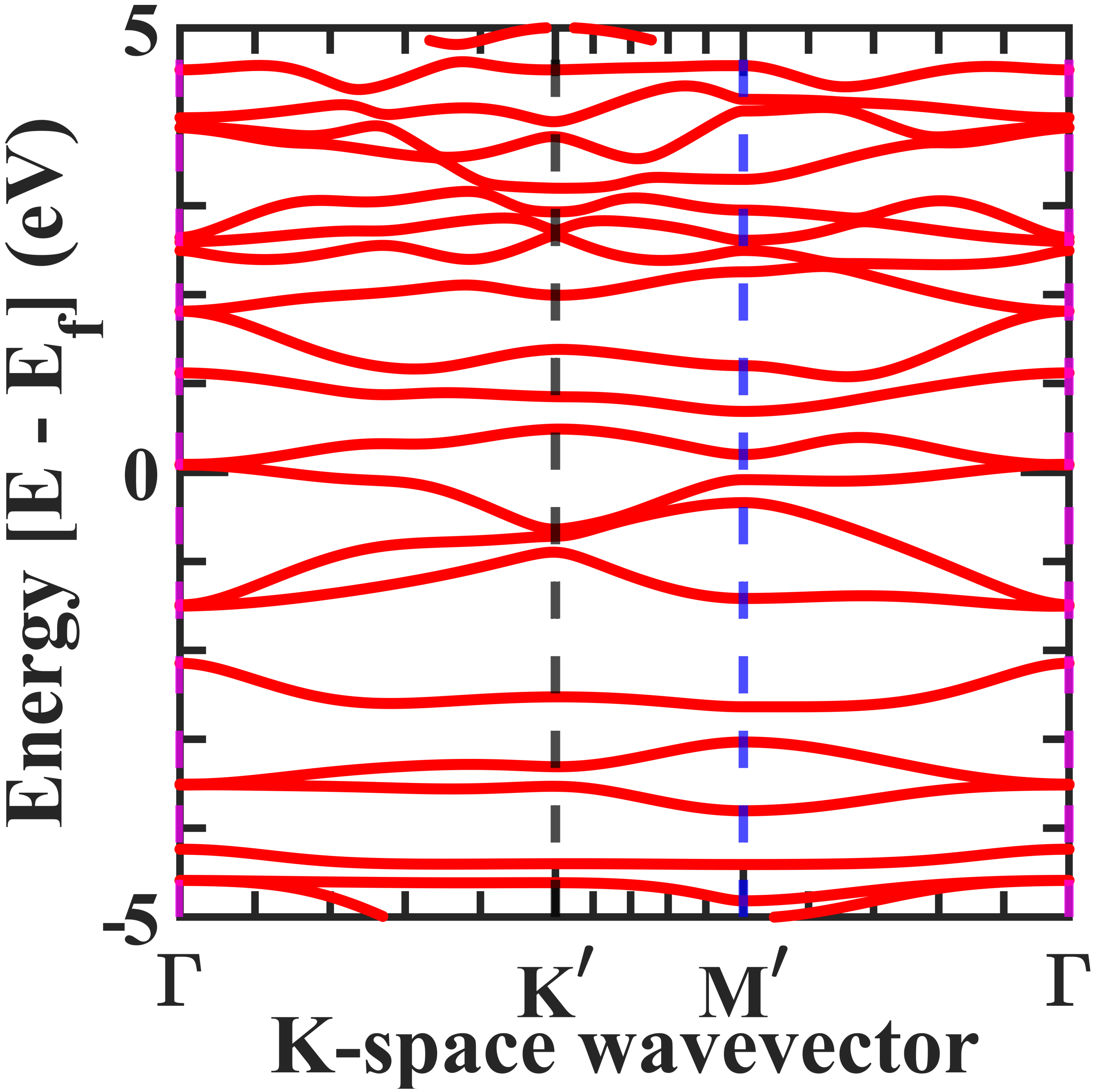

Figure 148. Band structure Phosphorus atom impurity 50x50 graphene supercell

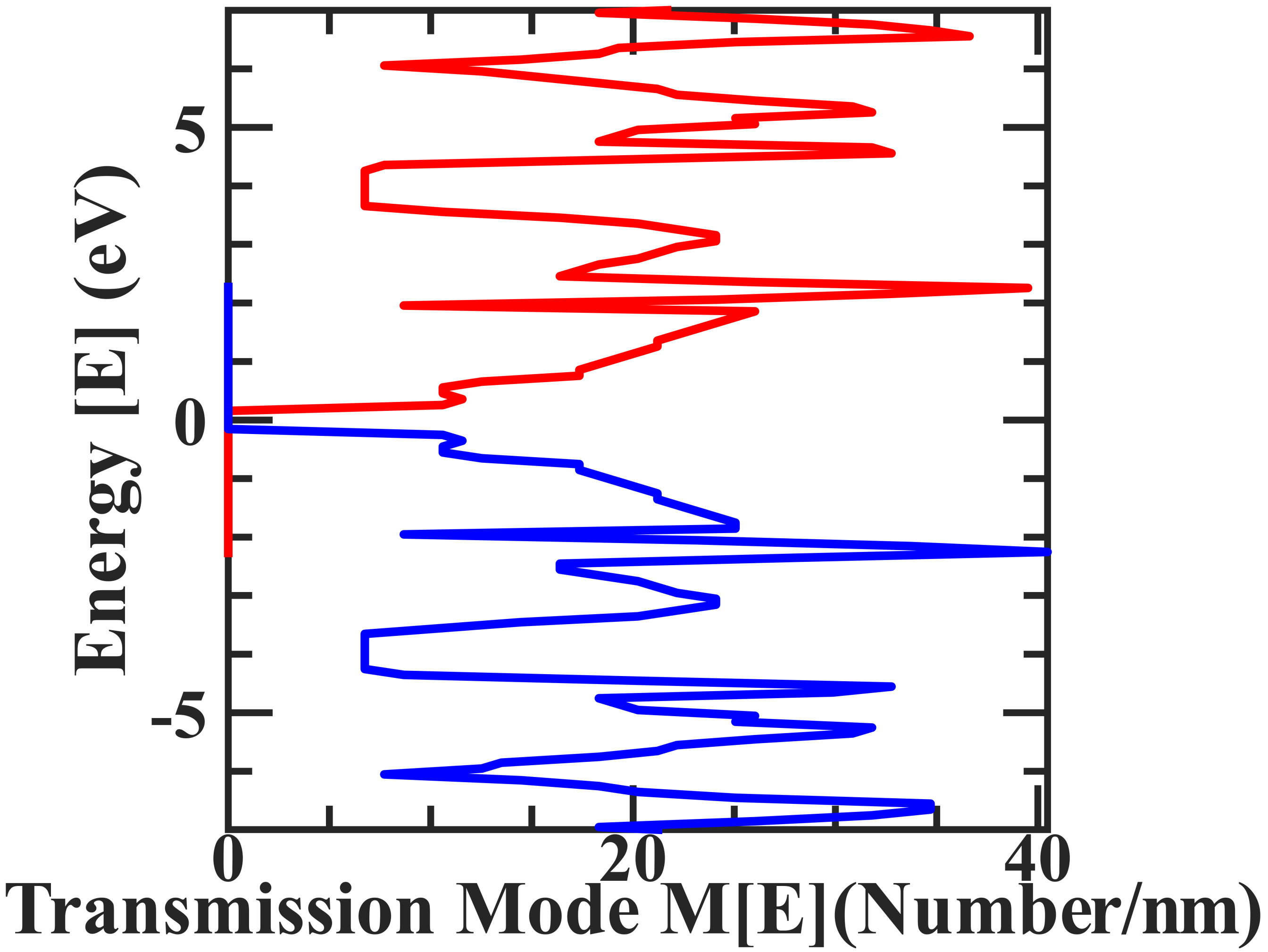

Figure 149. Density of mode Phosphorus atom impurity 50x50 graphene supercell

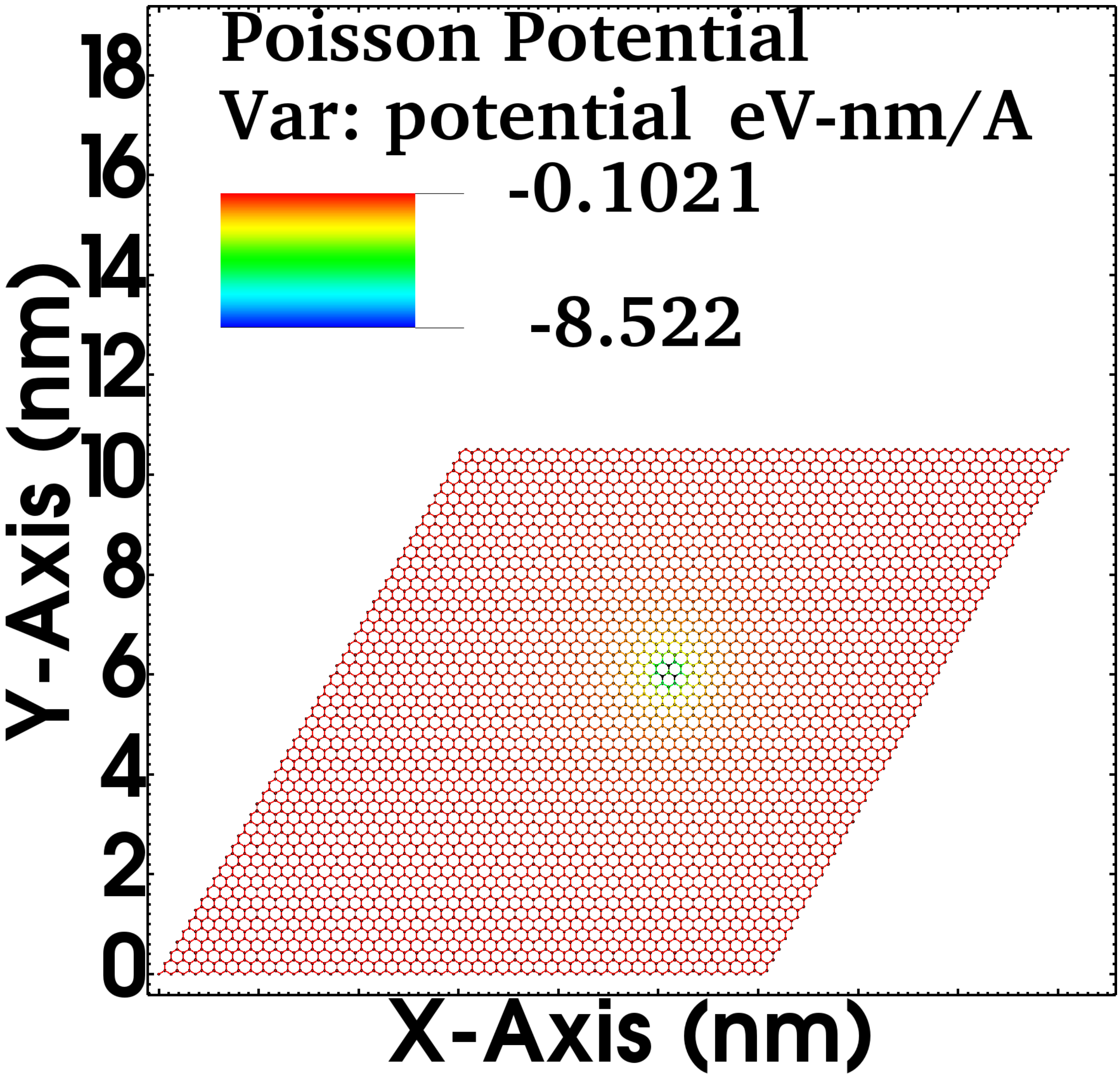

Figure 150. Self-consistent Poisson potential due to Phosphorus atom impurity 50x50 graphene supercell

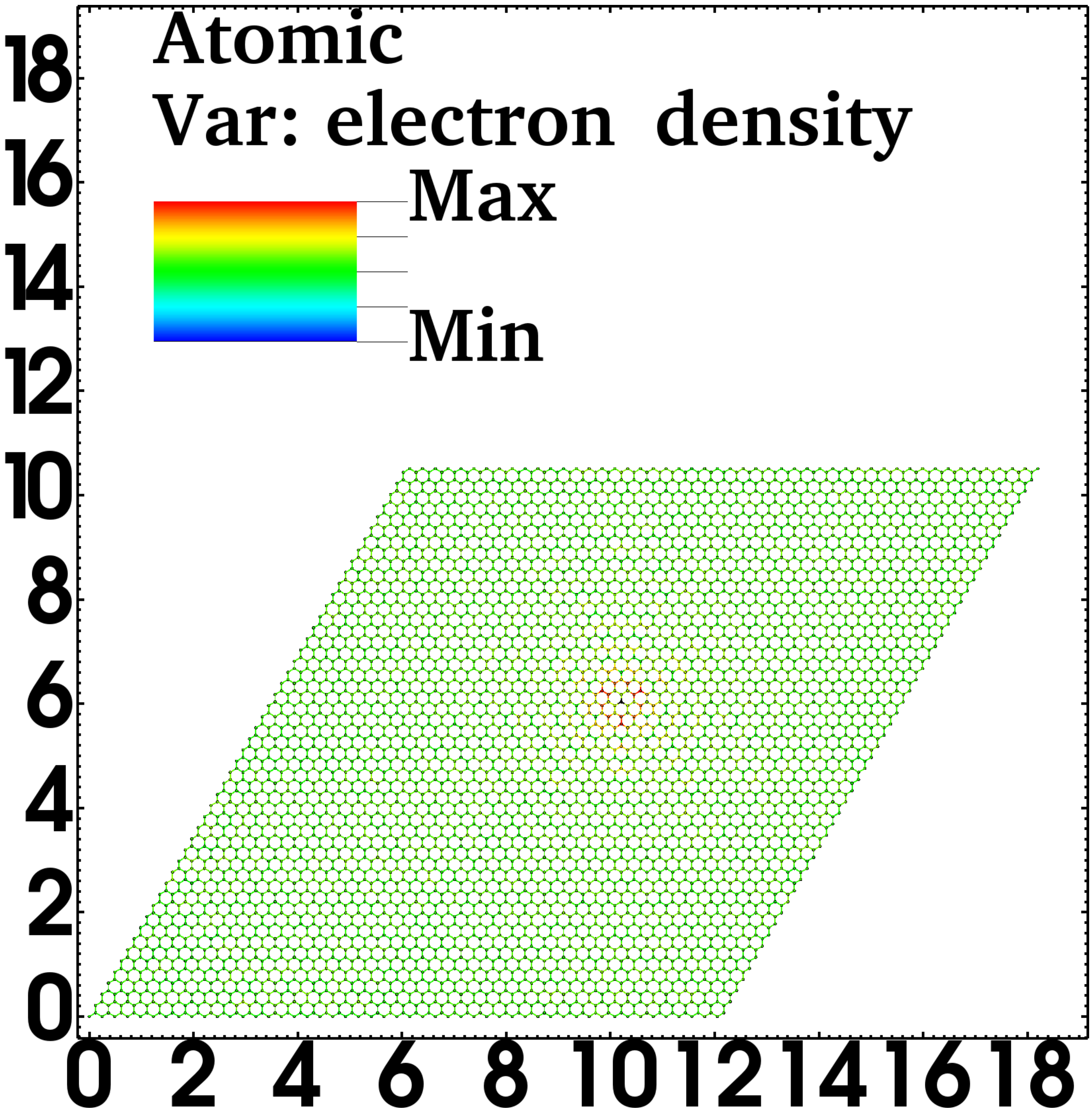

Figure 151. Electron density Phosphorus atom impurity 50x50 graphene supercell

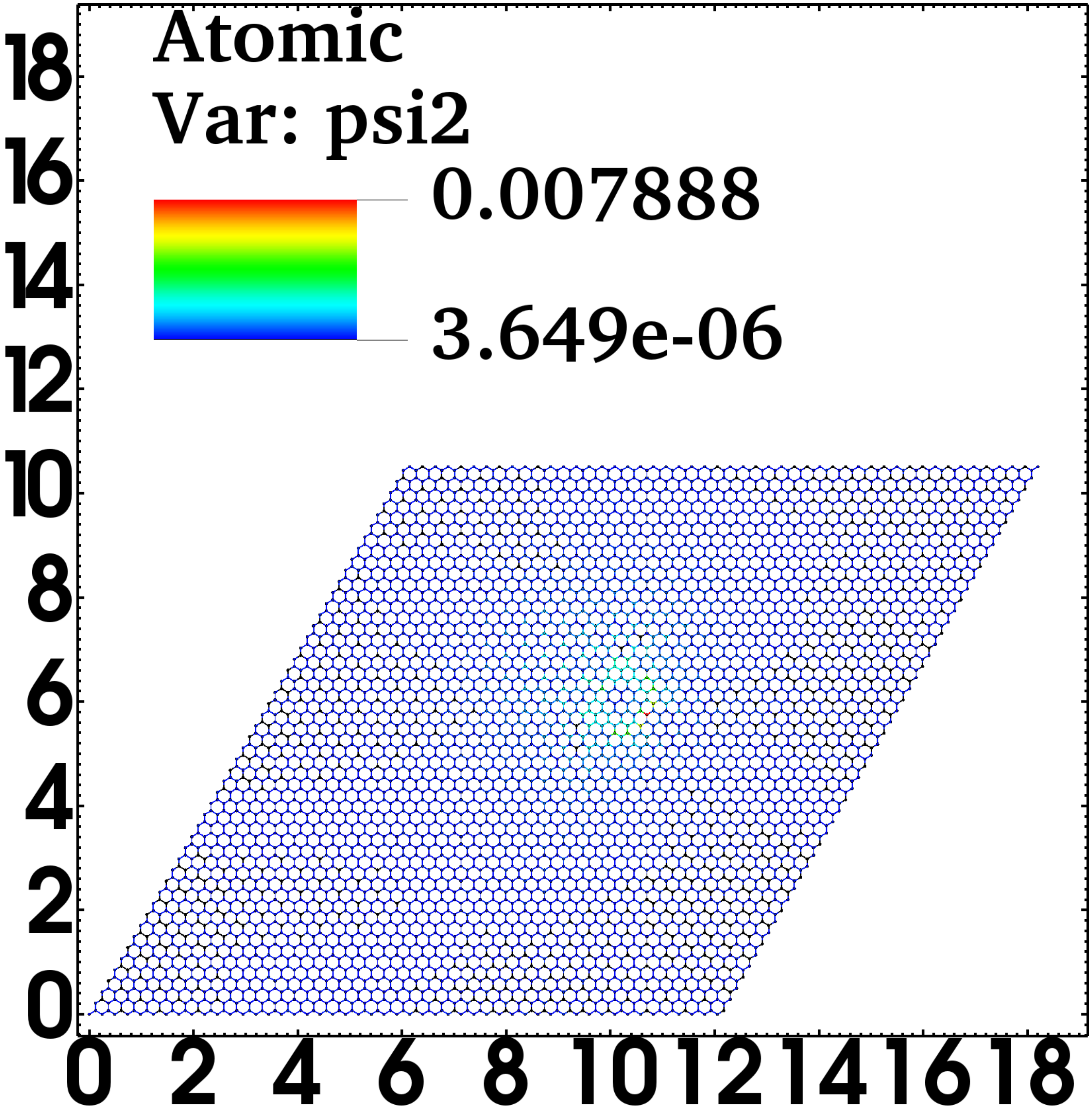

Figure 152. Spatially resolved electronic state $|\psi_0|^2$ Phosphorus atom impurity 50x50 graphene supercell

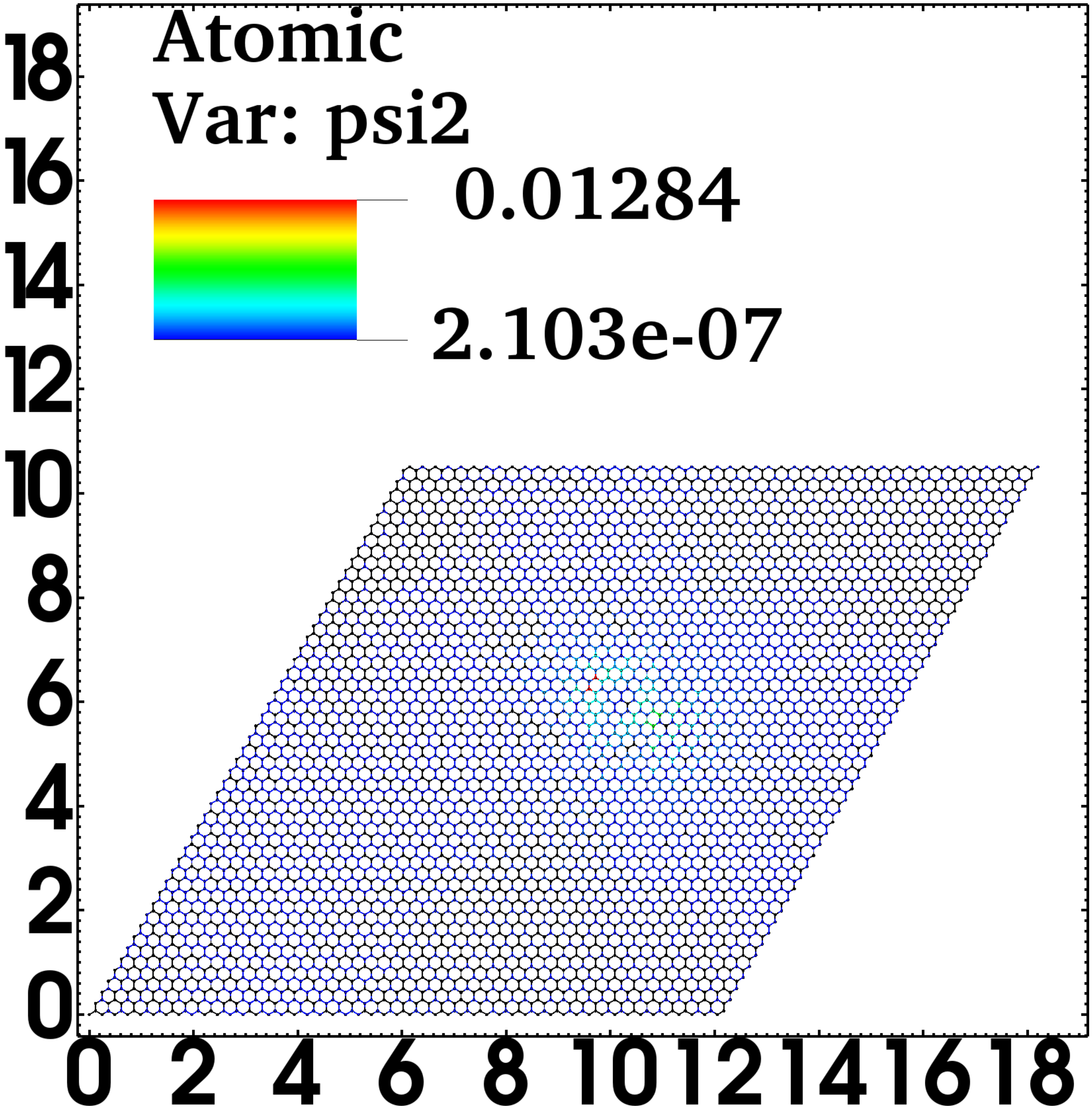

Figure 153. Spatially resolved electronic state $|\psi_1|^2$ Phosphorus atom impurity 50x50 graphene supercell

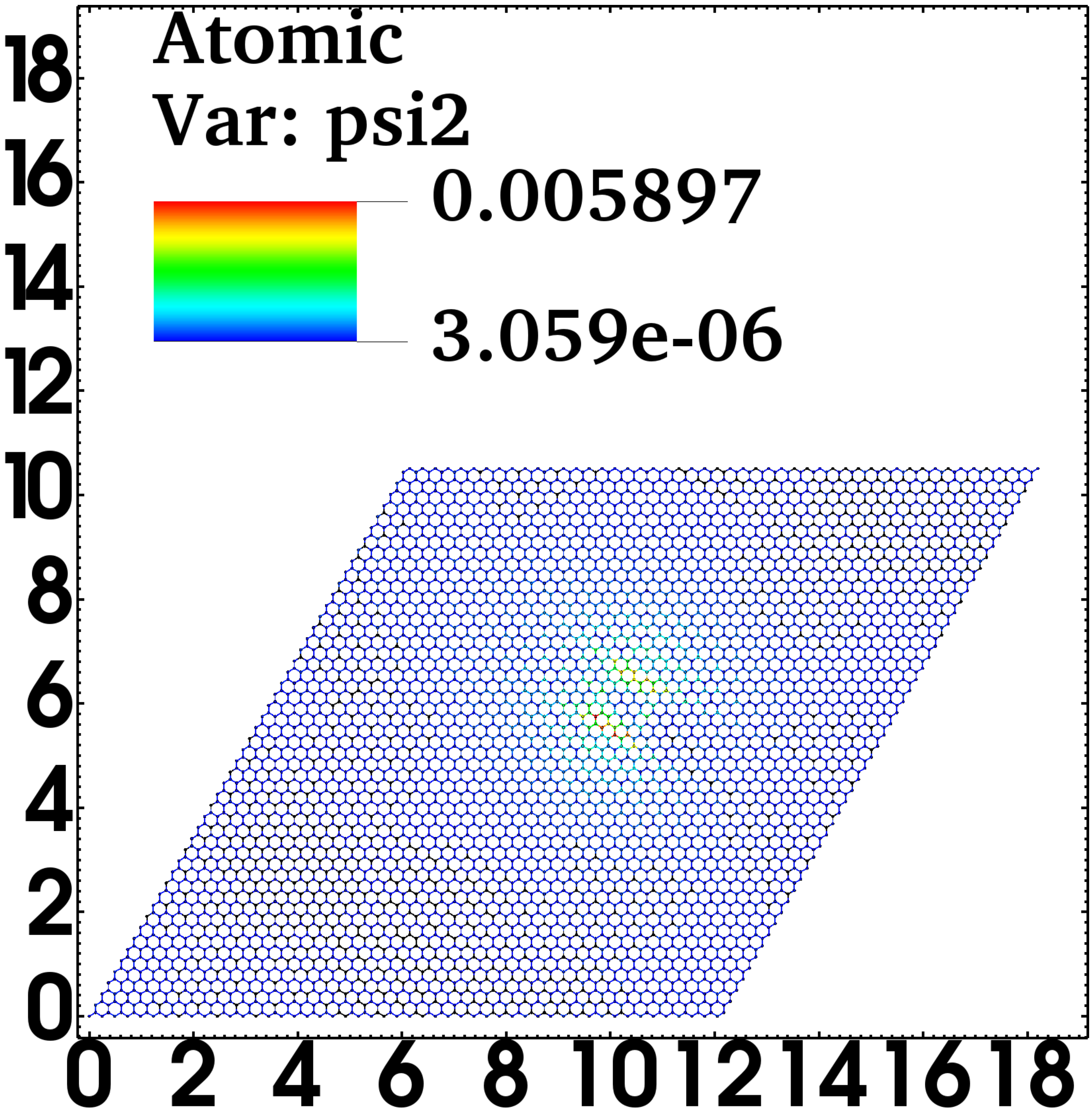

Figure 154. Spatially resolved electronic state $|\psi_2|^2$ Phosphorus atom impurity 50x50 graphene supercell

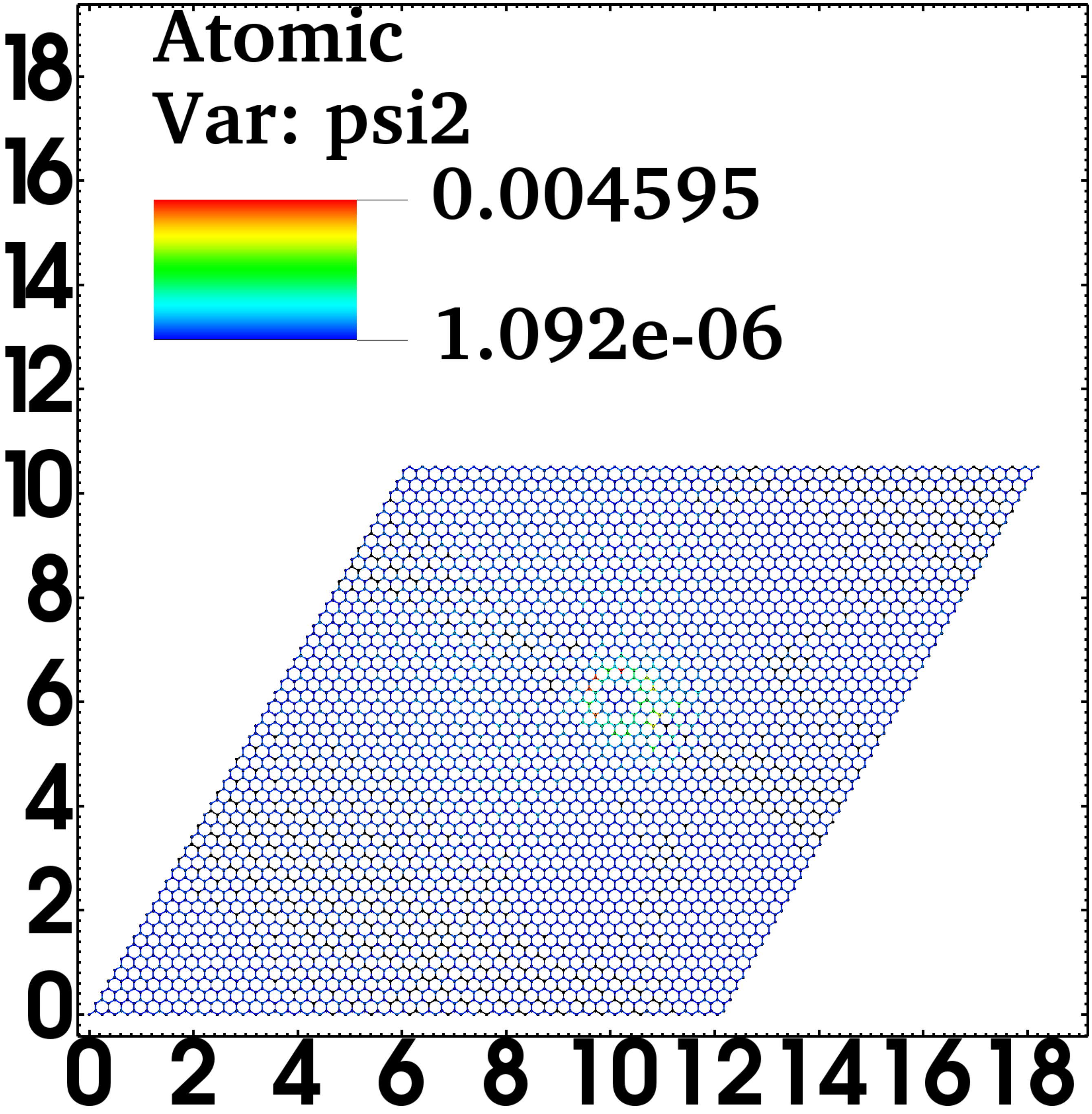

Figure 155. Spatially resolved electronic state $|\psi_3|^2$ Phosphorus atom impurity 50x50 graphene supercell

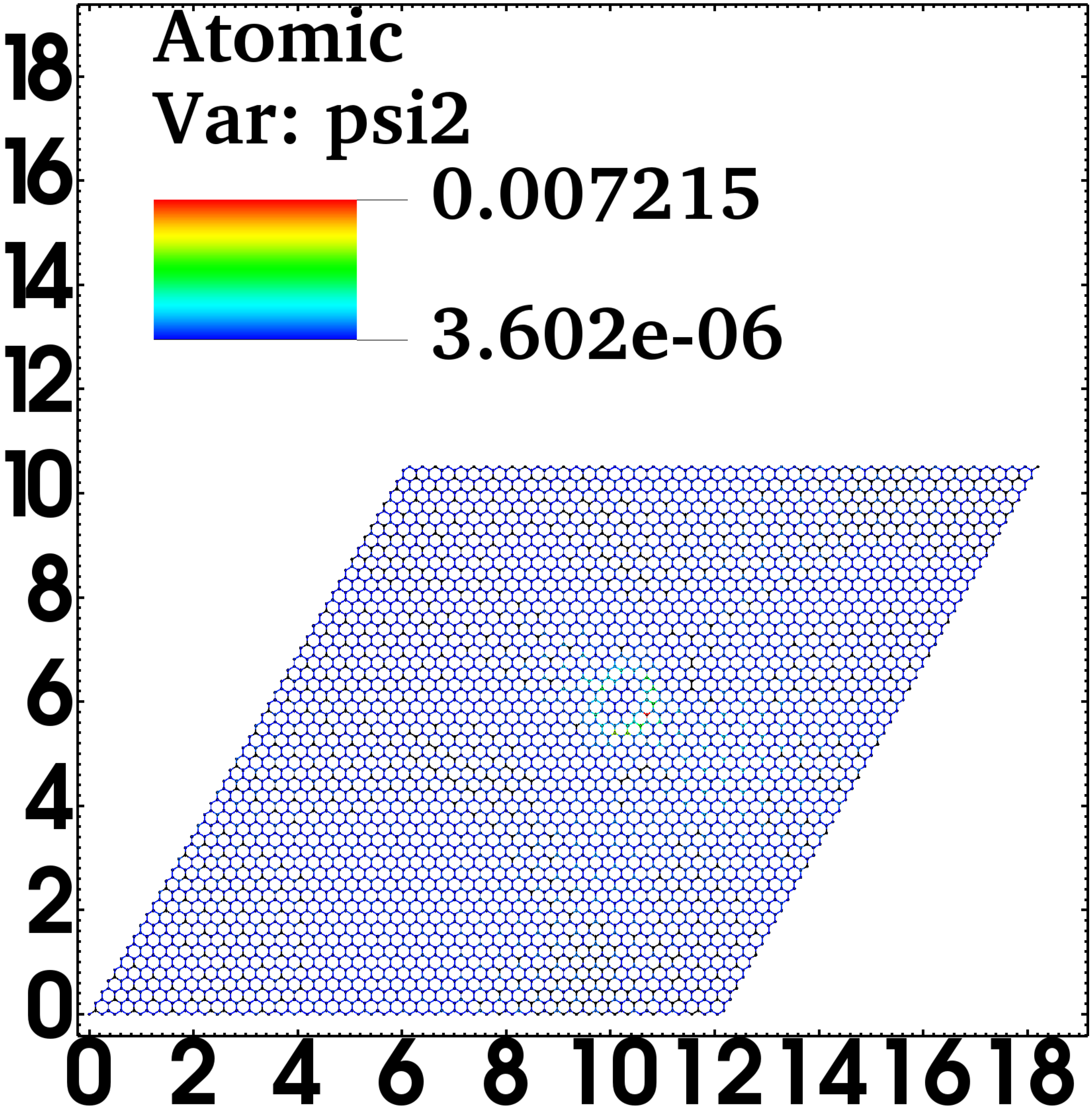

Figure 156. Spatially resolved electronic state $|\psi_4|^2$ Phosphorus atom impurity 50x50 graphene supercell

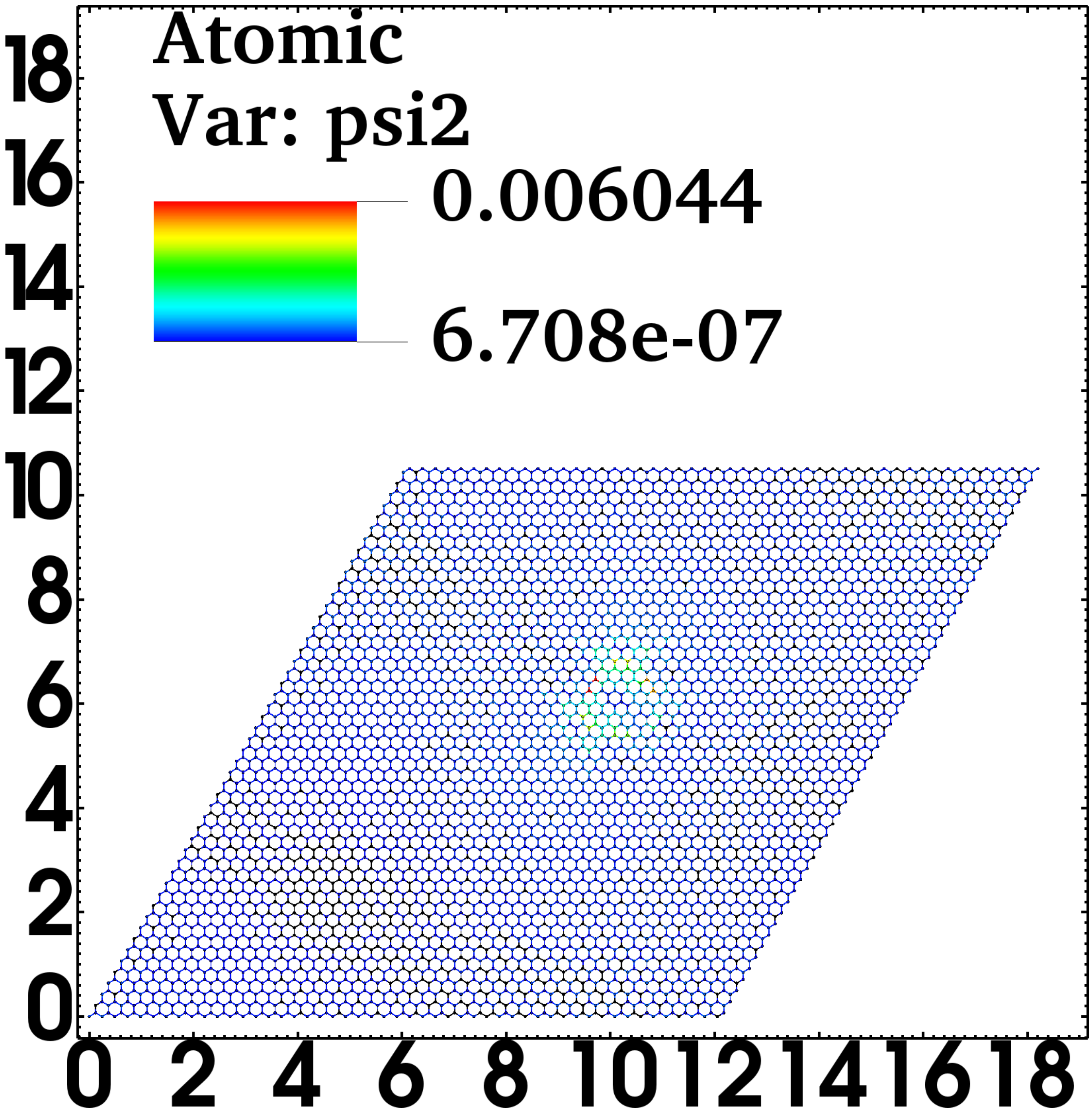

Figure 157. Spatially resolved electronic state $|\psi_5|^2$ Phosphorus atom impurity 50x50 graphene supercell

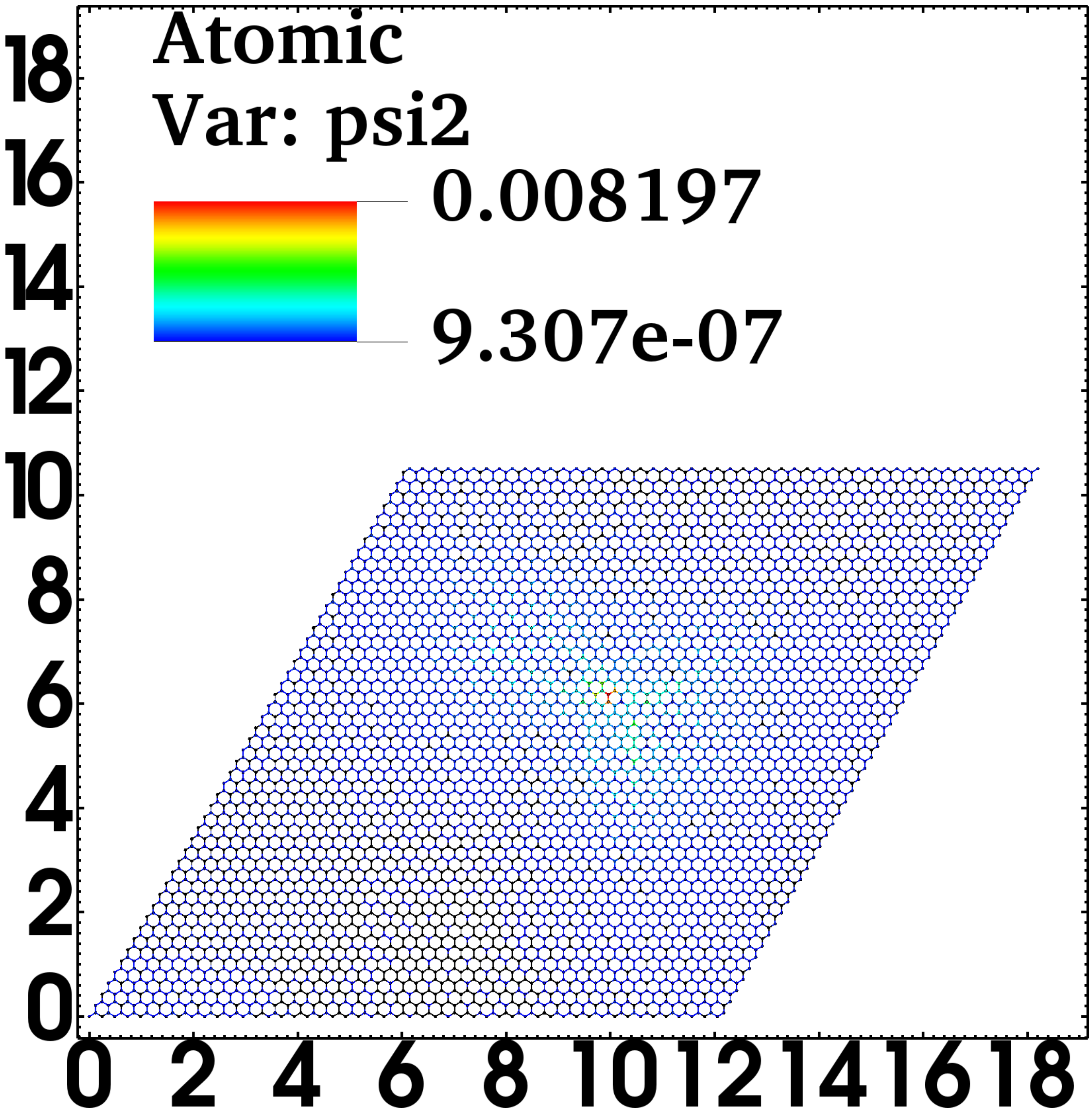

Figure 158. Spatially resolved electronic state $|\psi_6|^2$ Phosphorus atom impurity 50x50 graphene supercell

References

1. G. Baym and L. P. Kadanoff, "Conservation Laws and Correlation Functions," *Physical Review*, vol. 124, no. 2, pp. 287–299, Oct. 1961. [Online]. Available: https://link.aps.org/doi/10.1103/PhysRev.124.287
2. L. V. Keldysh, "Diagram technique for nonequilibrium processes," *Zh. Eksp. Teor. Fiz.*, vol. 47, pp. 1515–1527, 1964.
3. R. Lake and S. Datta, "Nonequilibrium Green's-function method applied to double-barrier resonant-tunneling diodes," *Physical Review B*, vol. 45, no. 12, pp. 6670–6685, Mar. 1992. [Online]. Available: https://link.aps.org/doi/10.1103/PhysRevB.45.6670

4. R. Lake, G. Klimeck, R. C. Bowen, and D. Jovanovic, “Single and multiband modeling of quantum electron transport through layered semiconductor devices,” *Journal of Applied Physics*, vol. 81, no. 12, pp. 7845–7869, Jun. 1997. [Online]. Available: https://aip.scitation.org/doi/10.1063/1.365394

5. A. Wacker, “Semiconductor superlattices: a model system for nonlinear transport,” *Physics Reports*, vol. 357, no. 1, pp. 1–111, Jan. 2002. [Online]. Available: http://www.sciencedirect.com/science/article/pii/S0370157301000291

6. P. Danielewicz, “Quantum theory of nonequilibrium processes, i,” *Annals of Physics*, vol. 152, no. 2, pp. 239–304, 1984. [Online]. Available: https://www.sciencedirect.com/science/article/pii/0003491684900927

7. ——, “Quantum theory of nonequilibrium processes ii. application to nuclear collisions,” *Annals of Physics*, vol. 152, no. 2, pp. 305–326, 1984. [Online]. Available: https://www.sciencedirect.com/science/article/pii/0003491684900939

8. R. Gebauer and R. Car, “Current in open quantum systems,” *Phys. Rev. Lett.*, vol. 93, p. 160404, Oct 2004. [Online]. Available: https://link.aps.org/doi/10.1103/PhysRevLett.93.160404

9. K. Burke, R. Car, and R. Gebauer, “Functional theory of the electrical conductivity of molecular devices,” *Phys. Rev. Lett.*, vol. 94, p. 146803, Apr 2005. [Online]. Available: https://link.aps.org/doi/10.1103/PhysRevLett.94.146803

10. T. Frederiksen, M. Paulsson, M. Brandbyge, and A.-P. Jauho, “Inelastic transport theory from first principles: Methodology and application to nanoscale devices,” *Phys. Rev. B*, vol. 75, p. 205413, May 2007. [Online]. Available: https://link.aps.org/doi/10.1103/PhysRevB.75.205413

11. R. Landauer, “Spatial variation of currents and fields due to localized scatterers in metallic conduction,” *IBM Journal of Research and Development*, vol. 1, no. 3, pp. 223–231, 1957.

12. S. Datta, *Electronic transport in mesoscopic systems*. Cambridge university press, 1997.

13. ——, “Nanoscale device modeling: the Green’s function method,” *Superlattices and Microstructures*, vol. 28, no. 4, pp. 253–278, Oct. 2000. [Online]. Available: http://www.sciencedirect.com/science/article/pii/S0749603600909200

14. M. Lundstrom, *Fundamentals of Carrier Transport*. Cambridge, England, UK: Cambridge University Press, Oct 2000.

15. M. Anantram, M. S. Lundstrom, and D. E. Nikonov, “Modeling of nanoscale devices,” *Proceedings of the IEEE*, vol. 96, no. 9, pp. 1511–1550, 2008.

16. D. Weinmann, *Quantum transport in nanostructures*. Wirtschaftsverl. NW, Verlag für Neue Wiss., 1994.

17. C. W. J. Beenakker and H. van Houten, “Quantum Transport in Semiconductor Nanostructures,” in *Solid State Physics*, ser. Semiconductor Heterostructures and Nanostructures, H. Ehrenreich and D. Turnbull, Eds. Academic Press, Jan. 1991, vol. 44, pp. 1–228. [Online]. Available: http://www.sciencedirect.com/science/article/pii/S0081194708600910

18. T. Kubis, *Quantum Transport in Semiconductor Nanostructures*, ser. Selected topics of semiconductor physics and technology. Walter Schottky Institut, Technische Universität München, 2009.

19. W. Fichtner, *Quantum Transport for Nanostructures*. Integrated Systems Laboratory, ETH Zürich, Sep. 2006. [Online]. Available: https://nanohub.org/resources/1792

20. M. R. Hirsbrunner, T. M. Philip, B. Basa, Y. Kim, M. J. Park, and M. J. Gilbert, “A review of modeling interacting transient phenomena with non-equilibrium Green functions,” *Reports on Progress in Physics*, vol. 82, no. 4, p. 046001, Mar. 2019, publisher: IOP Publishing. [Online]. Available: https://doi.org/10.1088%2F1361-6633%2Faafe5f

21. H. Haug and A.-P. Jauho, *Quantum Kinetics in Transport and Optics of Semiconductors*, 2nd ed., ser. Springer Series in Solid-State Sciences. Berlin Heidelberg: Springer-Verlag, 2008. [Online]. Available: https://www.springer.com/gp/book/9783540735618

22. J. Rammer and H. Smith, “Quantum field-theoretical methods in transport theory of metals,” *Reviews of Modern Physics*, vol. 58, no. 2, pp. 323–359, Apr. 1986, publisher: American Physical Society. [Online]. Available: https://link.aps.org/doi/10.1103/RevModPhys.58.323

23. P. C. Martin, “Theory of Many-Particle Systems. I,” *Physical Review*, vol. 115, no. 6, pp. 1342–1373, 1959.

24. L. P. Kadanoff, “Theory of Many-Particle Systems. II. Superconductivity,” *Physical Review*, vol. 124, no. 3, pp. 670–697, 1961.

25. R. P. Feynman, “Space-time approach to non-relativistic quantum mechanics,” *Rev. Mod. Phys.*, vol. 20, pp. 367–387, Apr 1948. [Online]. Available: https://link.aps.org/doi/10.1103/RevModPhys.20.367

26. J. Schwinger, “On gauge invariance and vacuum polarization,” *Phys. Rev.*, vol. 82, pp. 664–679, Jun 1951. [Online]. Available: https://link.aps.org/doi/10.1103/PhysRev.82.664

27. R. D. Mattuck, *A guide to Feynman diagrams in the many-body problem*, 2nd ed., ser. Advanced book program. New York, NY: McGraw-Hill, 1976.

28. G. C. Wick, "The evaluation of the collision matrix," *Phys. Rev.*, vol. 80, pp. 268–272, Oct 1950. [Online]. Available: https://link.aps.org/doi/10.1103/PhysRev.80.268

29. R. Binder and S. W. Koch, "Nonequilibrium semiconductor dynamics," *Progress in Quantum Electronics*, vol. 19, no. 4, pp. 307–462, Jan. 1995. [Online]. Available: http://www.sciencedirect.com/science/article/pii/007967279500001S

30. S. S. Fanchenko, "Generalized diagram technique of nonequilibrium processes," *Theoretical and Mathematical Physics*, vol. 55, no. 1, pp. 406–409, Apr. 1983. [Online]. Available: https://link.springer.com/article/10.1007/BF01019028

31. A. L. Fetter and J. D. Walecka, *Quantum Theory of Many-particle Systems*. McGraw-Hill, 1971.

32. M. Wagner, "Expansions of nonequilibrium Green's functions," *Physical Review B*, vol. 44, no. 12, pp. 6104–6117, Sep. 1991, publisher: American Physical Society. [Online]. Available: https://link.aps.org/doi/10.1103/PhysRevB.44.6104

33. W. Schafer and M. Wegener, *Semiconductor Optics and Transport Phenomena*. Springer-Verlag Berlin Heidelberg, 2002. [Online]. Available: https://www.springer.com/gp/book/9783540616146

34. A. Svizhenko, M. P. Anantram, T. R. Govindan, B. Biegel, and R. Venugopal, "Two-dimensional quantum mechanical modeling of nanotransistors," *Journal of Applied Physics*, vol. 91, no. 4, pp. 2343–2354, Jan. 2002, publisher: American Institute of Physics. [Online]. Available: https://aip.scitation.org/doi/10.1063/1.1432117

35. F. J. Dyson, "The s matrix in quantum electrodynamics," *Phys. Rev.*, vol. 75, pp. 1736–1755, Jun 1949. [Online]. Available: https://link.aps.org/doi/10.1103/PhysRev.75.1736

36. J. Schwinger, "On the Green's functions of quantized fields. I," *Proceedings of the National Academy of Sciences*, vol. 37, no. 7, pp. 452–455, 1951. [Online]. Available: https://www.pnas.org/content/37/7/452

37. ——, "On the Green's functions of quantized fields. II," *Proceedings of the National Academy of Sciences*, vol. 37, no. 7, pp. 455–459, 1951, publisher: National Academy of Sciences. [Online]. Available: https://www.pnas.org/content/37/7/455

38. L. Hedin, "New method for calculating the one-particle green's function with application to the electron-gas problem," *Phys. Rev.*, vol. 139, pp. A796–a823, Aug 1965. [Online]. Available: https://link.aps.org/doi/10.1103/PhysRev.139.A796

39. R. A. Craig, "Perturbation Expansion for Real-Time Green's Functions," *Journal of Mathematical Physics*, vol. 9, no. 4, pp. 605–611, Apr. 1968, publisher: American Institute of Physics. [Online]. Available: https://aip.scitation.org/doi/abs/10.1063/1.1664616

40. P. Danielewicz, "Quantum theory of nonequilibrium processes, I," *Annals of Physics*, vol. 152, no. 2, pp. 239–304, Feb. 1984. [Online]. Available: http://www.sciencedirect.com/science/article/pii/0003491684900927

41. G. D. Mahan, *Many-Particle Physics*, 2nd ed., ser. Physics of Solids and Liquids. Springer US, 1990. [Online]. Available: https://www.springer.com/gp/book/9780306434235

42. S.-C. Lee and A. Wacker, "Nonequilibrium Green's function theory for transport and gain properties of quantum cascade structures," *Physical Review B*, vol. 66, no. 24, p. 245314, Dec. 2002, publisher: American Physical Society. [Online]. Available: https://link.aps.org/doi/10.1103/PhysRevB.66.245314

43. R. Haydock, "The recursive solution of the Schrödinger equation," *Computer Physics Communications*, vol. 20, no. 1, pp. 11–16, Sep. 1980. [Online]. Available: http://www.sciencedirect.com/science/article/pii/0010465580901010

44. F. Teichert, A. Zienert, J. Schuster, and M. Schreiber, "Improved recursive Green's function formalism for quasi one-dimensional systems with realistic defects," *Journal of Computational Physics*, vol. 334, pp. 607–619, Apr. 2017. [Online]. Available: http://www.sciencedirect.com/science/article/pii/S0021999117300347

45. D. J. Thouless and S. Kirkpatrick, "Conductivity of the disordered linear chain," *Journal of Physics C: Solid State Physics*, vol. 14, no. 3, pp. 235–245, Jan. 1981. [Online]. Available: https://doi.org/10.1088%2F0022-3719%2F14%2F3%2F007

46. A. MacKinnon, "The calculation of transport properties and density of states of disordered solids," *Zeitschrift für Physik B Condensed Matter*, vol. 59, no. 4, pp. 385–390, Dec. 1985. [Online]. Available: https://doi.org/10.1007/BF01328846

47. A. Rahman, J. Guo, S. Datta, and M. S. Lundstrom, "Theory of ballistic nanotransistors," *Electron Devices, IEEE Transactions on*, vol. 50, no. 9, pp. 1853–1864, 2003.

48. S. Cauley, M. Luisier, V. Balakrishnan, G. Klimeck, and C. K. Koh, "Distributed non-equilibrium Green's function algorithms for the simulation of nanoelectronic devices with scattering," *Journal of Applied Physics*, vol. 110, no. 4, p. 043713, 2011.

Acknowledgements

This work was funded by the New Energy and Industrial Technology Development Organization of Japan (NEDO) Grant (JPNP16010).

Author contributions

H.N., M.B., and B.B. designed this study. B.B. carried out the NEGF transport calculations, constructed the roughness and impurity model, performed associated calculations, analyzed the data, and wrote the manuscript text. H.N., M.B., and B.B. discussed the results and participated in the manuscript arrangement. All authors reviewed the final submitted manuscript.

Competing interests

The authors declare no competing interests.

Additional information

Correspondence and requests for materials should be addressed to B.B.
Reprints and permissions information is available at www.nature.com/reprints.
